# Highly Stereoselective Synthesis of a Compound Collection Based on the Bicyclic Scaffolds of Natural Products

**DOI:** 10.3390/molecules22050827

**Published:** 2017-05-18

**Authors:** Murali Annamalai, Stanimira Hristeva, Martyna Bielska, Raquel Ortega, Kamal Kumar

**Affiliations:** 1 Max-Planck-Institut für molekulare Physiologie, Otto-Hahn-Straße 11, 44227 Dortmund, Germany; Murali.Annamalai@mpi-dortmund.mpg.de; 2 Medicinal Chemistry, Taros Chemicals GmbH & Co. KG, Emil Figge-Str. 76a, 44227 Dortmund, Germany; shristeva@taros.de (S.H.); mbielska@taros.de (M.B.); rortega@taros.de (R.O.)

**Keywords:** natural products, drug discovery, scaffolds, Aza-heterocycles, European Lead Factory

## Abstract

Despite the great contribution of natural products in the history of successful drug discovery, there are significant limitations that persuade the pharmaceutical industry to evade natural products in drug discovery research. The extreme scarcity as well as structural complexity of natural products renders their practical synthetic access and further modifications extremely challenging. Although other alternative technologies, particularly combinatorial chemistry, were embraced by the pharmaceutical industry to get quick access to a large number of small molecules with simple frameworks that often lack three-dimensional complexity, hardly any success was achieved in the discovery of lead molecules. To acquire chemotypes beholding structural features of natural products, for instance high *sp*^3^ character, the synthesis of compound collections based on core-scaffolds of natural products presents a promising strategy. Here, we report a natural product inspired synthesis of six different chemotypes and their derivatives for drug discovery research. These bicyclic hetero- and carbocyclic scaffolds are highly novel, rich in *sp*^3^ features and with ideal physicochemical properties to display drug likeness. The functional groups on the scaffolds were exploited further to generate corresponding compound collections. Synthesis of two of these collections exemplified with ca. 350 compounds are each also presented. The whole compound library is being exposed to various biological screenings within the European Lead Factory consortium.

## 1. Introduction

**Figure 1:**
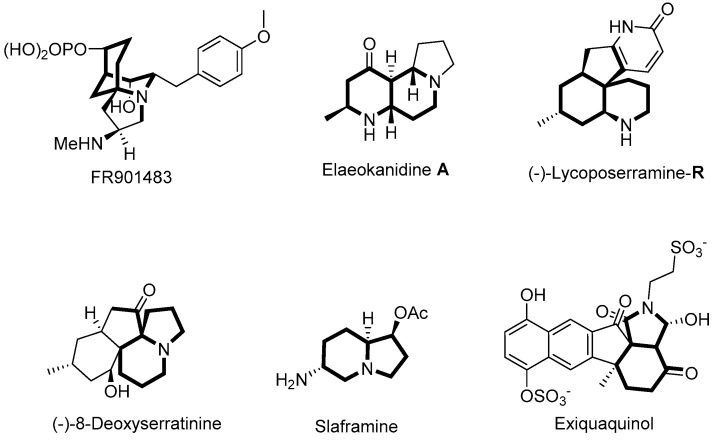
Biologically active natural products.

Natural products (NPs) have been a rich source of bioactive small molecules that fuel the drug discovery processes [1,2,3,4]. To a large extent, NPs owe this success, in addition to their enormous structural and chemical diversity, to their amazingly drug-like molecular properties [5,6,7]. A detailed analysis of new medicines approved by the FDA in the past three decades revealed that one third of small molecule medicines were based on either NPs or their derivatives [8]. In addition, the majority of the molecules that have entered into clinical trials, in particular the anticancer and antimicrobial agents, are based on NPs [9,10]. However, there are few significant challenges in engaging NPs in drug discovery and development process. While the extremely low abundance of NPs remains critical for their isolation, the highly complex structures of NPs pose a formidable challenge to their practical synthesis and further modifications to generate a decent sized compound collection for screening platforms [11,12,13]. To tackle these problems, structural simplification of NPs and generation of compound collections around the core-scaffolds of the NPs present a promising strategy to gain access to biologically relevant chemical space [14,15,16,17,18,19]. The Joint European Compound Library (JECL) is a key component of the European Lead Factory (ELF) [20,21] that holds drug-like and lead-like compounds for ELF’s screening facility [22,23,24]. In order to enrich this collection with small molecules rich in *sp*^3^ features, chiral centers as well as drug-like molecular properties, different academic groups and small and medium enterprises (SMEs) are collaborating in library synthesis programs [25,26,27,28,29,30,31,32,33,34]. With this perspective and along with our continuing interest in the synthesis of natural product inspired compound collections for biological studies [34,35,36,37,38,39,40,41,42], we report here our synthesis efforts to build a compound library that is represented by six highly *sp*^3^-rich, novel and distinct hetero- and carbocyclic scaffolds that are found as core-structures in biologically active NPs, as depicted in Figure 1.

## 2. Results and Discussion

**Figure 2:**
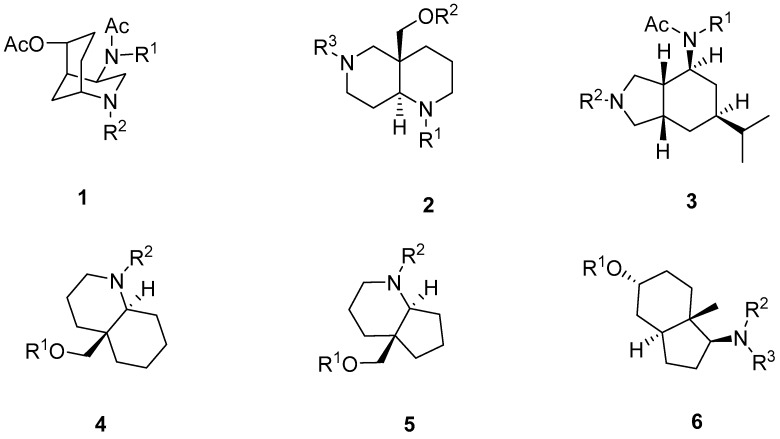
Natural product inspired bicyclic scaffolds.

Design and synthesis of novel and medicinally relevant chemical entities is one of the key objectives in drug discovery. In the ELF syntheses programs, we aim to generate a small molecule library around complex molecular frameworks with acceptable molecular properties and thereby facilitate the identification of lead candidates for further drug discovery campaigns. Although molecular complexity is a subjective term, we refer to a scaffold complex when it is rich in *sp*^3^ features, has a three-dimensionally complex framework supporting a number of chiral centers, and structural features that are reminiscent of NP structures [43,44,45]. With these desired structural features, six scaffolds were chosen as targets (**1**–**6**, Figure 2) for our synthetic planning. The scaffolds **1**–**6** represent the structural blueprints of biologically active NPs and we hope that a collection of small molecules based on these chemotypes could provide interesting hit and lead molecules in ELF campaigns.

### 2.1. Synthesis of Aza-Bicyclic Scaffolds

Scaffold **1** is a bridged bicyclic system with four chiral centers and three points of diversification and is based on an immunosuppressant natural product FR901483 [46].

**Scheme 1:**
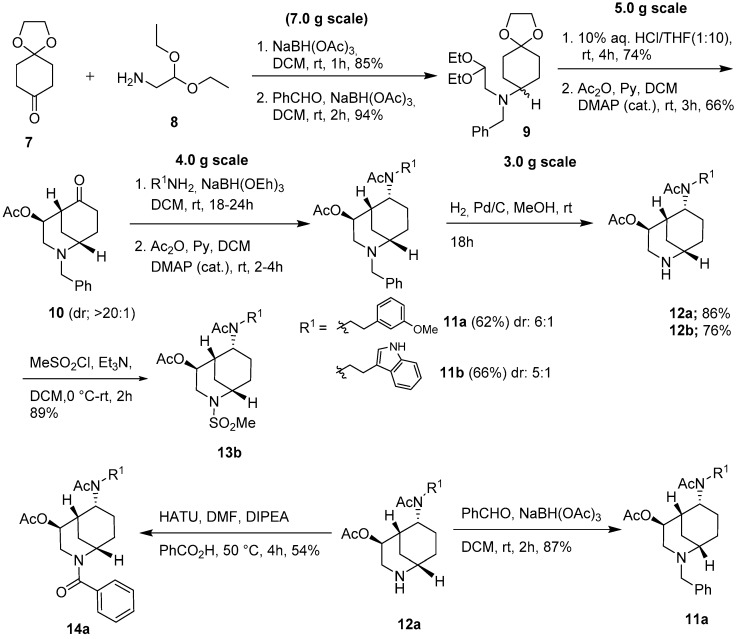
Synthesis optimization of an Immunosuppressant FR901483 based scaffold.

The scaffold synthesis began with mono-ketal **7** of cyclohexane 1,4-dione, which on sequential reductive amination reactions using amino-acetal **8**, and then with benzaldehyde using sodium triacetoxyborohydride, provided the corresponding amino-acetal **9** at an excellent yield (Scheme 1). The reactions were performed on a multigram scale to provide a sufficient amount of material for library synthesis. The key intramolecular aldol reaction between the ketone function and aldehyde (formed after acetal-deprotection) was performed with high diastereoselectivity (>20:1) in the presence of 10% aq. HCl [47]. Subsequent acylation of alcohol offered the ketone moiety in **10** as a handle for reductive amination reactions with a variety of aryl-ethyl amines—for instance, delivering secondary amines (**11a**–**b**) at a good yield and with very good diastereoselectivity. *N*-debenzylation of the major diastereomer under hydrogenolysis with 10% palladium on carbon gave the amines **12a** and **12b**, and the compounds **12a** and **12b** were successively converted into corresponding sulfonamide **13b** and amide **14a** (Scheme 1). As a representative case of another reductive amination with benzaldehyde, **11a** was also synthesized at a very good yield, thereby demonstrating the potential of the highly functionalized scaffold in compound collection synthesis. The relative stereochemistry of the major diastereomer was predicted based on an nOe experiment (see Supplementary Materials).

**Scheme 2:**
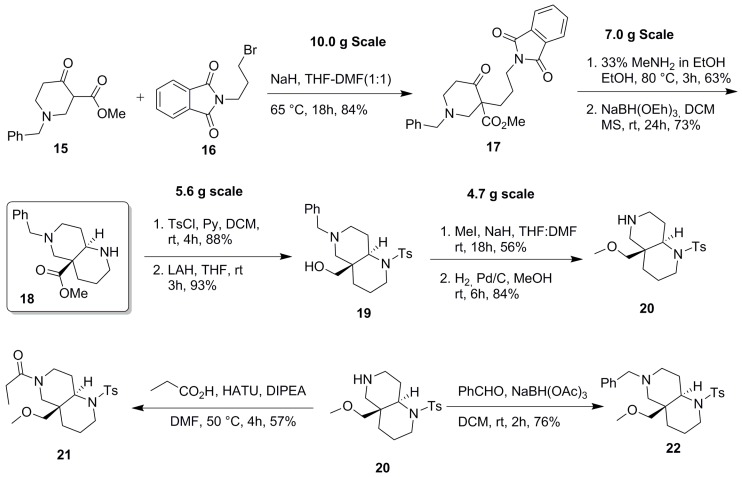
Stereoselective synthesis of 1,6-decahydronaphthyridine scaffold.

Scaffold **2** is based on natural product Elaeokanidine A [48] and is characterized by a 1,6-Naphthyridine framework with a quaternary carbon at the ring junction and two chiral centers. The scaffold holds three sites for diversification to make a compound collection. The synthesis in this case began with the inexpensive and commercial β-keto-ester **15** that was alkylated using 1-bromo-propylpthalimide **16** in the presence of sodium hydride at a very good yield (Scheme 2). The phthalimide group in **17** was removed under aminolysis conditions using 30% MeNH_2_ solution in ethanol followed by stereoselective reductive amination using a bulky hydride source i.e., sodium tri(2-ethylhexanoyloxy)borohydride (NaBH(OEh)_3_) [49] leading to the formation of bicyclic amine **18** at a good yield and with high stereoselectivity (dr: 10:1). The secondary amine in **18** was converted into sulfonamide at a very good yield using *p*-toluenesulfonylchloride with the presence of pyridine as a base. In addition, the ester group was reduced to alcohol (**19**). Alkylation of the later with methyl iodide in the presence of sodium hydride afforded the corresponding ether at a good yield (Scheme 2).

Finally, the *N*-benzyl group was removed under hydrogenolysis conditions with 10% palladium on carbon to give the corresponding free secondary amine **20**, which was successively converted into the corresponding amide **21** and *tert*-amine **22** using propionic acid and benzaldehyde, respectively (Scheme 2).

**Scheme 3:**
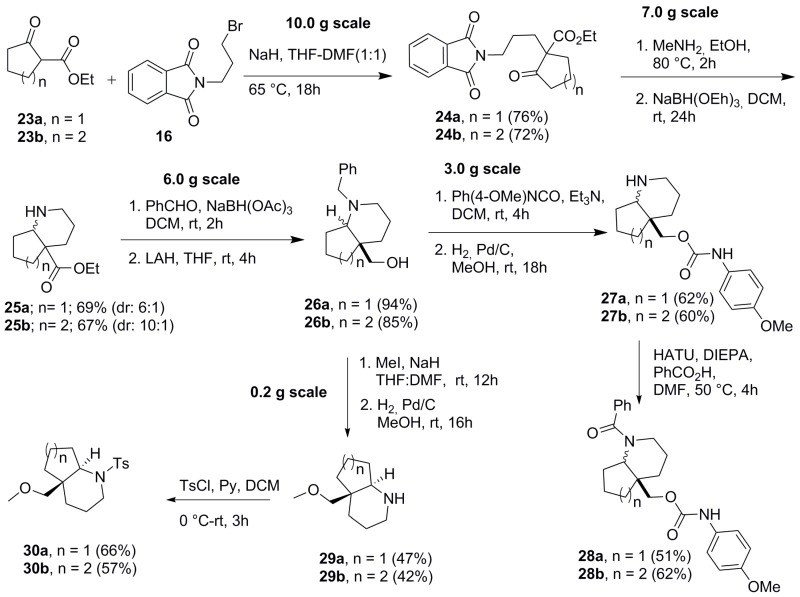
A lycoposerramine-R alkaloid inspired scaffold synthesis.

A similar strategy was used with other inexpensive and commercially available keto-esters **23** and leading to bicyclic scaffolds **25,** which is the core-structure of the lycoposerramine natural product (Scheme 3) [50,51,52,53,54]. With this synthesis design and following standard diversification reactions as depicted in Scheme 2, the final small molecules (**25**–**30**) were obtained with high stereoselectivity (**25a**:(6:1); **25b**:(10:1) Scheme 3). The diastereomers were purified by flash column chromatography and the major diastereomer was employed in the further reaction sequence (Scheme 3).

One of the important sources of three-dimensional complexity in small molecules is the commercial chiral pool. To exploit the potential of this source, we chose (*S*)-carvon (**31**) as 3D-rich starting material and subjected it to 1,3-dipolar cycloaddition reaction with an in situ generated azomethine ylide from commercially available *N*-(Methoxymethyl)-*N*-(trimethyl-silylmethyl)-benzylamine (NMNTB) hoping to achieve further molecular complexity by a [3 + 2]-cycloaddition reaction [55]. Unfortunately, all the reported reaction conditions for such a cycloaddition reaction failed to give the desired product, and, instead, decomposition of the ylide precursor was observed. We reasoned that the α-methyl group of the carvone **31** has a negative steric influence on the reactivity of the ylide. In addition, it reduces the electrophilicity of the α-β-unsaturated ketone and thereby further resists the cycloaddition reaction. Therefore, a *des*-methyl carvone **32** was synthesized according to the literature report with excellent enantioselectivity and employed in the desired cycloaddition reaction [56].

**Scheme 4:**
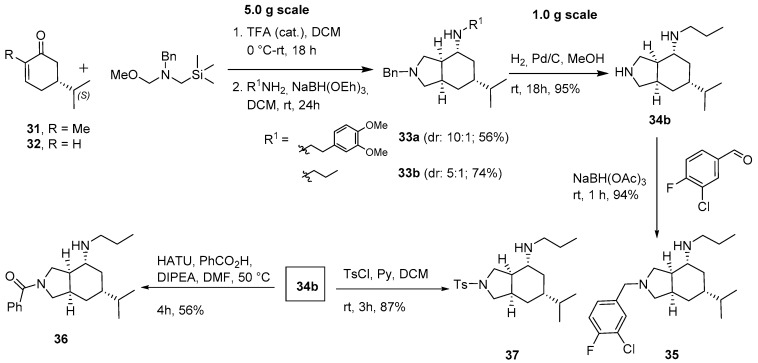
A (−)-*des*-methyl-Carvone derived compound collection.

The *des*-methyl carvone **32** smoothly underwent 1,3-dipolar cycloaddition with azomethine ylide generated from NMNTB in the presence of catalytic amount of trifluoroacetic acid (TFA) to give the corresponding cycloadduct **33** as a single diastereomer at a good yield (Scheme 4). Subsequently, the ketone moiety under the previously optimized stereoselective reductive amination conditions afforded the corresponding secondary amines **33a** and **33b**. Furthermore, debenzylation of the bicyclic compound **33a** under hydrogenolysis conditions afforded the diamine **34b** at a good yield. The diamine as such was converted to corresponding *tert*-amine (**35**), amide (**36**) and sulphonamide (**37**) at a good yield using the standard reaction conditions and thus demonstrating the amenability of the design to build a compound collection (Scheme 4).

### 2.2. Synthesis Optimization of a Carbo-Bicyclic Scaffold

**Scheme 5:**
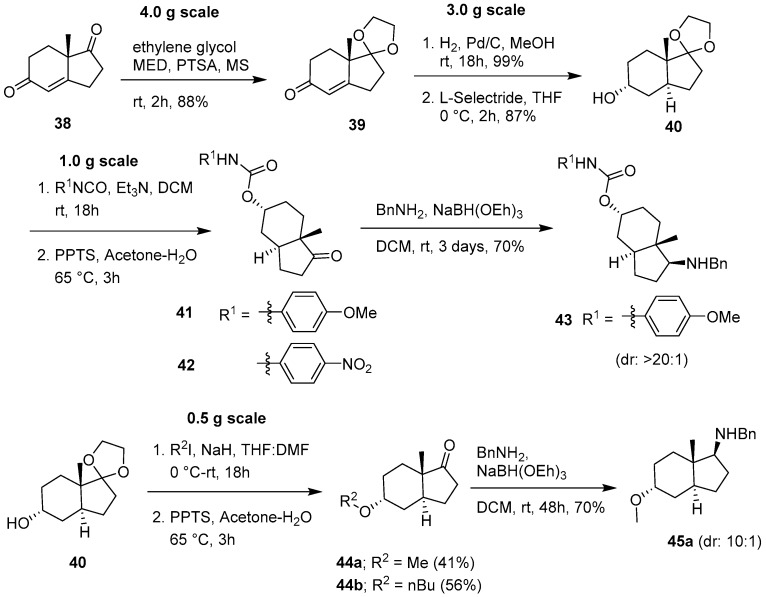
Synthesis of a carbocyclic scaffold for library synthesis using Hajas–Parrish Ketone.

In the majority of previous scaffolds validated towards compound collection synthesis, the piperidine ring was an integral part of the bicyclic framework. We intended to replace the aza-ring with a carbocycle in the next scaffold. In our synthesis design, we realized that commercially available chiral Hajos-Parrish ketone **38** was an excellent substrate to develop such a carbo-bicyclic scaffold. To this end, a chemoselective protection of diketone **38** was performed [56] yielding the corresponding mono-ketal **39** at an excellent yield (Scheme 5). A diastereoselective hydrogenation of the enone moiety using 10% palladium on carbon led to a saturated ketone at an excellent yield. The ketone was reduced to a secondary alcohol **40** using l-selectride in tetrahydrofuran (THF) at −78 °C with excellent stereoselectivity (dr: >20:1; please see supporting information). The secondary alcohol moiety was then utilized as the first diversity point by converting it to the corresponding carbamates (**41**–**42**) and ethers (**44**) using isocyanates and alkyl halides, respectively. Deprotection of the ketal to release the ketone function in **41**–**42** and **44** offered another site for diversification via stereoselective reductive amination with various aliphatic and aromatic primary amines to give the final compound (**43** and **45**) at a very good yield and selectivity (Scheme 5).

### 2.3. Representative Compound Collection Synthesis around Bicyclic Scaffolds

The academic and SME partners in ELF consortium work together on a library synthesis design and its execution. While the academic partner validates the synthesis of the scaffold that is decorated with functional groups or sites for modifications during library synthesis, the real compound collection is actually built up by the SME partner. Understandably, this requires a very sound collaboration between the two and a facile transfer of knowledge for executing the synthesis design to realize a compound library. To showcase this pragmatic and resourceful tactic [57], we exemplify the upscale and production of two compound collections based on bicyclic scaffolds **2** and **4**–**5** (Scheme 1).

**Scheme 6:**
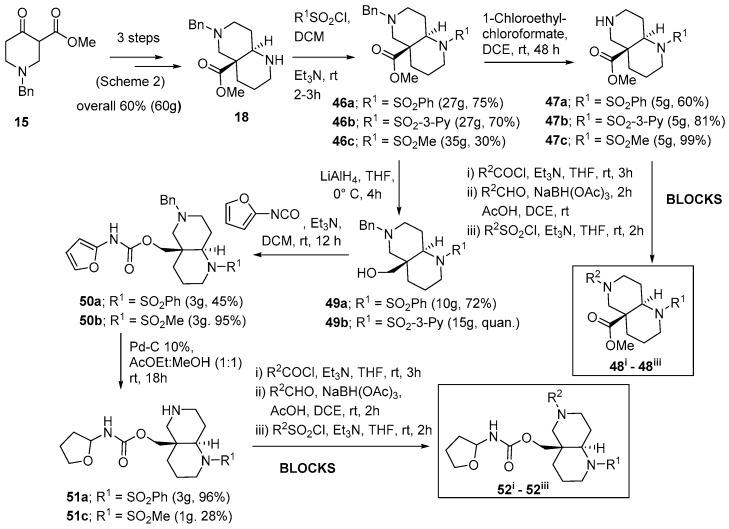
Production of a compound collection based on bicyclic scaffold **2**.

In the case of scaffold **2**, the first steps of the validation reaction sequence were optimized to obtain **18** with an overall yield of 60% after three steps from **15** in an 80 g scale operation. The critical point in this sequence was the purification of **18**, solved by the precipitation of the corresponding hydrochloride salt that was recovered by filtration with a 98% yield. Sulphonylation reactions performed using aryl and alkyl sulphonyl chlorides in more than 25 g scale provided clean products **46a**–**c** (Scheme 6). To exploit another diversity handle required the deprotection of the *N*-benzyl group on the fused piperidine. Interestingly, the palladium catalyzed standard debenzylation protocol did not work in the presence of ester moiety (vide infra). All three of the sulphonylated scaffolds **46a**–**c** failed to undergo hydrogenolysis to offer a secondary amine (Table 1). Pleasingly, reaction of these bicyclic molecules with 1-chloroethylchloroformate in dichloromethane (DCM) afforded the desired products with good yields (**47a–c**). At this stage, three different blocks for the parallel synthesis and employing a 24 position Mettler Toledo block equipped with 15 mL reaction tubes were used for three different reaction types with secondary amine **47** i.e., amide synthesis, reductive amination and the sulphonylation reactions to yield a compound collection **48^i^–48^iii^** (Scheme 6). Notably, in the production phase, exchanging the base pyridine with triethylamine facilitated the workup of the reaction.

The methyl ester on a quaternary carbon was not an easy handle for parallel reactions. Therefore, it was reduced to alcohol **49** using LiAlH_4_. Although *O*-alkylation was successfully performed, the next *N*-debenzylation again did not work effectively (data not shown), probably due to the formation of a quaternary salt so another approach was used by the formation of a carbamate **50** by the reaction of isocyanates with primary alcohol **49**. Removal of *N*-benzyl function under palladium catalyzed the hydrogenolysis condition (Table 1) in **50** again provided a site in **51** for parallel synthesis that was performed as described above using three different blocks to give a compound collection **52^i^**–**52^iii^** (Scheme 6). The choice of reagents employed was dictated by the desired drug-like molecular properties in the compound collection. After high performance liquid chromatography/mass spectrometry (HPLC/MS)-based analysis and purification, this improved synthetic protocol yielded a total of 354 compounds with an overall success rate of 80% with sufficient quantity (>5 µmol) and a median purity (LC-MS) of 99%.

**Scheme 7:**
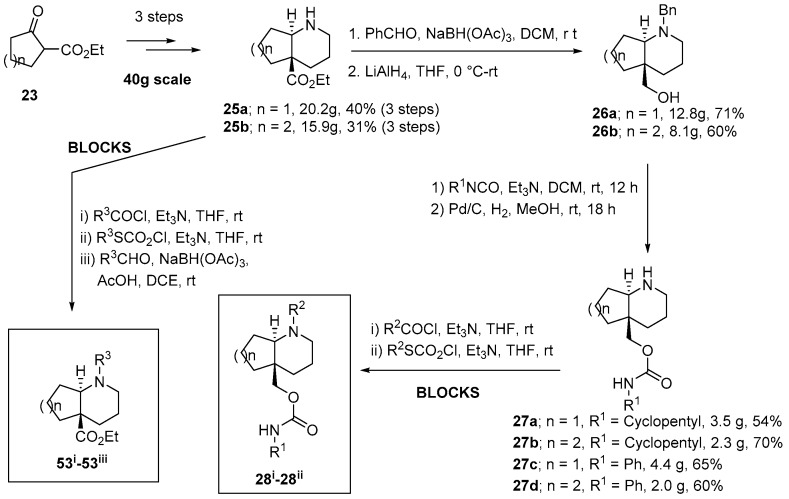
Production and diversification scaffolds **4**–**5**.

Encouraged by the successful production of a compound collection based on scaffold **2**, a similar strategy and optimization was followed for the production of a library based on scaffolds **4** and **5**. The desired intermediates **25a** and **25b** were obtained from commercially available ketoesters **23** on a 40 g scale. At this scale, prolonged reaction times were required for completion of the reaction, which afforded crude products of relatively lower purity (compared to the scaffold validation synthesis). However, the desired products **25a** and **25b** could still be obtained with very high purity by simple precipitation as hydrochloride salts, affording a global yield of 40% and 31%, respectively, after three steps. Furthermore, the stereoselectivity of the last step was completely reproducible at a large scale (Scheme 7). These intermediates were diversified in parallel fashion with three typical diversity reactions i.e., acylation, reductive amination and sulfonylation using three blocks for parallel synthesis and producing a collection **53^i^**–**53^iii^** (Scheme 7). Benzyl protection and ester reduction in **25** produced the corresponding alcohols **26a** and **26b** (up to 15 g scale) with similar yields as in the scaffold validation (Scheme 3). Furthermore, carbamate formation with cyclopentyl- and phenylisocyanate was performed to give intermediates carbamates, which, after debenzylation, yielded the four final intermediates **27a**–**d** in good yields on a 5 g scale. A compound collection using **27** was generated using combinatorial chemistry protocols for two diversity reactions (i.e., sulfonylation and acylation) yielding the final compounds **28^i^**–**28^ii^** of 35–75%. In this case, urea formation was avoided as final urea compounds were shown to be instable in the validation process. After HPLC/MS-based analysis and purification, a total of 333 compounds with an overall success rate of 82% with sufficient quantity (>5 µmol) and a median purity (LC-MS) of 99% were isolated.

**Figure 3:**
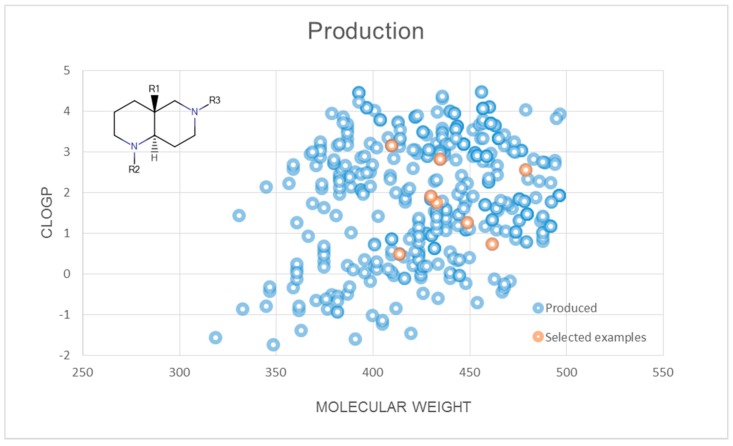
Production plot for scaffold **2**.

**Figure 4:**
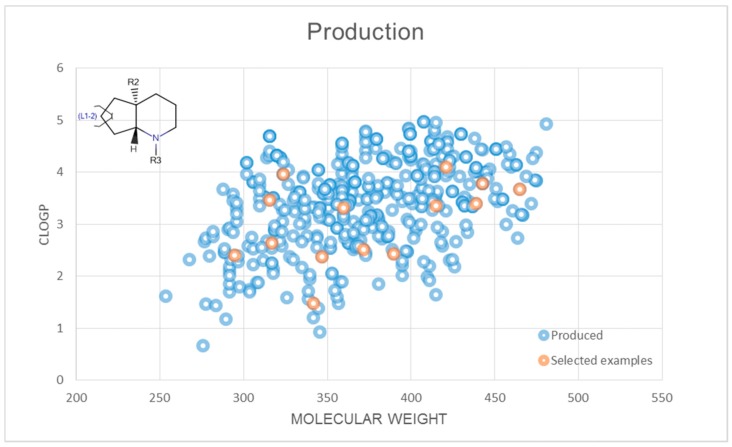
Production plot for scaffolds **5**–**6**.

Figure 3 and Figure 4 depict the physicochemical properties (Calculated LogP (clogP) and molecular weight (MW), JChem, ChemAxon 6.3.0) for the synthesized compounds in the two compound collections (blue circles). The selected compounds marked as orange circles are representative members of the complete compound collection synthesized (Scheme 6 and Scheme 7). As depicted in Figure 3 and Figure 4, all final compounds have “lead-like” properties (i.e., calculated LogP (CLogP) ≤ 5 and Molecular Weight (MW) ≤ 500) [29,58].

## 3. Conclusions

In conclusion, a synthesis planning targeting six *sp*^3^-rich scaffolds amenable to compound collection synthesis was designed and executed successfully. The three-dimensional core structures were inspired by natural products and were further modified using parallel synthesis platform and in-house collection of final diversification reagents. To ensure the structural-variability and -richness in the ensuing library members, at least three diversification points were explored with different vectors of diversity. All the compounds are novel and have drug-like molecular properties. Moreover, the synthetic routes were optimized to minimize the number of steps and establish a practical, robust and efficient synthetic process both in the scale up (5–10 g) synthesis of the intermediates and in the parallel synthesis at the final diversification point (5 μM). The purification of the final compounds by preparative HPLC-MS was also optimized allowing the successful isolation of compounds even in challenging cases like low yielding reactions or compounds devoid of UV absorbance with a median final purity of 99%. Finally, a well-established, integrated and efficient workflow was implemented. The collection is a part of JECL and is being evaluated to identify modulators of different therapeutically relevant protein targets in the screening portal of European Lead Factory [25,26,27,28,30,31,32,33]. 

## Figures and Tables

**Table 1:** Optimization of the *N*-debenzylation of **46** and **51**.

Entry	Product	R^1^	Reagent	Reaction Time (h)	Yield (%)
1	**47a**	SO_2_Ph	Pd/C 10% (0.3 Eqv.), H_2_ (1 atm)	96	0
2	**47a**	SO_2_Ph	1-Cholorethylchloroformate (2.5 Eqv.)	96	58
3	**47b**	SO_2_-3-Py	1-Cholorethylchloroformate (2.5 Eqv.)	48	81
4	**47c**	SO_2_Me	Pd/C 10% (0.3 Eqv.), H_2_ (1 atm)	48	0
5	**47c**	SO_2_Me	1-Cholorethylchloroformate (2.5 Eqv.)	48	99
6	**51a**	SO_2_-Ph	Pd/C 10% (0.3 Eqv.), H_2_ (1 atm)	48	96
7	**51c**	SO_2_Me	Pd/C 10% (0.3 Eqv.), H_2_ (1 atm)	96	28

## Supplementary Materials

Supplementary materials are available online.
