# Supplementary material for: Highly Stereoselective Synthesis of a Compound Collection Based on the Bicyclic Scaffolds of Natural Products
Source: Molecules. 2017 May 18;22(5):827. doi: 10.3390/molecules22050827 (PMC6153746; doi:10.3390/molecules22050827)
Supplement: Supplementary file 1 [file molecules-22-00827-s001.pdf]

Supporting Information

Highly Stereoselective Synthesis of a Compound Collection based on Bicyclic Scaffolds of Natural Products

Murali Annamalai[a], Stanimira Hristeva[b], Martyna Bielska[b], Raquel Ortega[b], and Kamal Kumar*[a]

a) Max-Planck-Institut für molekulare Physiologie, Otto-Hahn-Straße 11, 44227 Dortmund, Germany; b) Medicinal Chemistry, Taros Chemicals GmbH & Co. KG, Emil Figge-Str. 76a, 44227, Germany.

Email: Kamal.kumar@mpi-dortmund.mpg.de

Table of Contents for Chemical experiments *Pages*

1. *Materials and methods* …………………………………………………………….. ….*1*
2. *Experimental Procedures* ………………………………………………………………….*2*
3. *Experimental for the production phase of Elaeokanidine-A based scaffolds*.………….*13*
4. *Experimental for the production phase of 8-deoxy serratinine based scaffolds*……..35
5. *Copies of NMR Spectras*…………………………………………………………………….*63*

Materials and methods: Chemicals and solvents were obtained from commercial vendors and were used without further purification. All dry reactions were performed under argon atmosphere using commercial dry solvents. Column chromatography was performed on a silica column using 230−400 mesh silica gel. Thin-layer chromatography was performed on Macherey Nagel pre-coated TLC aluminum sheets with silica gel 60 UV254 (5-17 μm). Compound visualization was effected with UV lamp, Iodine, phosphomolybdic acid in ethanol and aq. KMnO4 solution. ^{1}H NMR spectra were recorded at rt on a Bruker Avance spectrometer operating at 400 MHz. Chemical shifts are given in ppm (δ) from tetramethylsilane as an internal standard or residual solvent peak. Significant ^{1}H NMR data are tabulated in the following order: multiplicity (s, singlet; d, doublet; t, triplet; q, quartet; m, multiplet; br, broad), coupling constant(s) in hertz, number of protons. Mass spectrometry was recorded using Agilent uHPLC (1290 Infinity) equipped with a Diode Array Detector and a Quadrupole MSD using mixture gradients of formic acid/water/acetonitrile as system solvent. High-resolution electrospray ionization mass spectra (ESI-FTMS) were recorded on a Thermo LTQ Orbitrap (high-resolution mass spectrometer from Thermo Electron) coupled to an "Accela" HPLC system supplied with a "Hypersil GOLD" column (Thermo Electron).

Experimental procedures:

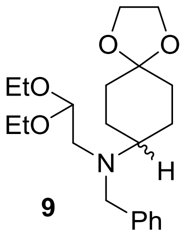

9

Chemical Formula: $C_{21}H_{33}NO_4$
Exact Mass: 363.2410
Molecular Weight: 363.4980

N-benzyl-N-(2,2-diethoxyethyl)-1,4-dioxaspiro[4.5]decan-8-amine (9): 2,2-diethoxyethan-1-amine **8** (3.63 g; 34.15 mmol) was dissolved in dry DCM (100 ml) and cooled to 0 °C, then 1,4-cyclohexanedione monoethylene acetal **7** (5.0 g; 31.05 mmol) was added dropwise. After 10 min $NaBH(OAc)_3$ (7.89 g; 37.26 mmol) was added in one portion and stirred for 2h at room temp. The reaction mixture was diluted with DCM and saturated aq. $NaHCO_3$ was added slowly, and then stirred for additional 15 min at rt. The layers were separated, the DCM layer was washed couple of times with saturated aq. $NaHCO_3$, water, brine solution and dried over anhydrous Na_2SO_4. After concentration the crude residue was filtered through silica column to afford pure compound **9a** 7.2 g (85% yield).

The amine compound **9a** (7.0 g; 25.60 mmol) was re-dissolved in DCM (105 mL), benzaldehyde (2.98 g; 28.16 mmol) was added dropwise at 0 °C, after 15 min $NaBH(OAc)_3$ (6.5 g; 30.72 mmol) was added in one portion. Then the reaction mixture was diluted with DCM, aq. $NaHCO_3$ solution was added allowed to stir for 10 min. The layers were separated, and washed couple of times with saturated aq. $NaHCO_3$ solution, then water, brine solution and dried over anhydrous Na_2SO_4. After evaporation the crude residue was filtered through silica column to give pure compound (**9**) 8.7 g (93% yield).; ^{1}H NMR (400 MHz, $cdcl_3$) δ 7.34 (d, J = 7.2 Hz, 2H), 7.29 – 7.24 (m, 2H), 7.19 (t, J = 7.2 Hz, 1H), 4.33 (t, J = 5.2 Hz, 1H), 3.89 (d, J = 1.1 Hz, 4H), 3.71 (s, 2H), 3.58 (dq, J = 9.1, 7.0 Hz, 2H), 3.47 – 3.38 (m, 2H), 2.68 – 2.57 (m, 3H), 1.77 (t, J = 9.9 Hz, 4H), 1.60 (ddd, J = 25.1, 12.6, 3.1 Hz, 2H), 1.52 – 1.43 (m, 2H), 1.13 (t, J = 7.1 Hz, 6H).; 13C NMR (101 MHz, $CDCll_3$) δ 141.5, 128.6, 128.2, 126.7, 108.8, 103.9, 64.5, 64.4, 62.5, 59.1, 55.8, 54.0, 34.4, 25.7, 15.6.; HPLC-MS (ESI) m/z calcd. for $C_{21}H_{34}NO_4$ $[M+H]^+$ = 364.24; Found: 364.22.

TLC stain: I_2, $KMnO_4$

R_f: 0.6 (20% EtOAc in Pet-ether)

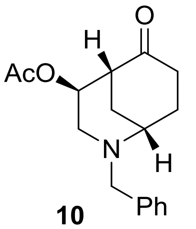

10

Chemical Formula: $C_{17}H_{21}NO_3$
Exact Mass: 287.15
Molecular Weight: 287.36

2-Benzyl-6-oxo-2-azabicyclo[3.3.1]nonan-4-yl acetate (10): The amino-acetal **9** (4.0 g; 11.0 mmol) was dissolved in a mixture of THF and 1.0 M aq. HCl (10:1; 55 mL), the resulting solution was stirred at rt. for 3 h. Then the reaction mixture was diluted with EtOAc, washed couple of times with saturated aq. $NaHCO_3$, water, and brine solution. After that dried over anhydrous Na_2SO_4, concentrated to give crude residue, which was carefully purified by column chromatography, using 5-10% of EtOAc in pet-ether as eluent to give the pure diastereomer 1.99g (74% yield).

TLC stain: UV, I_2, $KMnO_4$.

R_f: 0.2 (30% EtOAc in pet-ether).

The alcohol **10a** (1.0 g; 4.07 mmol) was re-dissolved in dry DCM (10 mL), and then Et_3N (0.495 g; 4.89 mmol) followed by Ac_2O (0.45 g; 4.48 mmol) and then catalytic amount of DMAP (0.05 g; 0.40 mmol) were added at 0 °C, the resulting solution was stirred 4 h. at rt. After the reaction completion, DCM was added washed couple of times with saturated aq. $NaHCO_3$ then water and brine solution. Then dried over anhydrous Na_2SO_4, concentrated and purified by column chromatography using 2-5% of EtOAc in pet-ether as gradient to give the pure acetate (**10)** 0.77 g (66% yield). ^{1}H NMR (400 MHz, CD_2Cl_2): δ 7.42 – 7.23 (m, 5H), 5.11 (dt, J = 10.7, 6.1 Hz, 1H), 3.84 (d, J = 13.4 Hz, 1H), 3.70 (d, J = 13.4 Hz, 1H), 3.02 (dd, J = 11.6, 6.4 Hz, 2H), 2.92 (d, J = 2.5 Hz, 1H), 2.68 – 2.53 (m, 2H), 2.48 – 2.39 (m, 2H), 2.21 – 2.14 (m, 1H), 2.02 (t, J = 3.3 Hz, 1H), 1.99 – 1.96 (m, 3H), 1.78 – 1.67 (m, 1H).; ^{13}C NMR (101 MHz, CD_2Cl_2): δ 209.9, 170.0, 137.0, 128.8, 128.5, 127.3, 69.6, 59.3, 50.8, 49.6, 47.8, 40.1, 31.7, 23.1, 20.9.; HPLC-MS (ESI) m/z calcd. for $C_{17}H_{22}NO_3$ $[M+H]^+$ = 288.15. Found: 288.11.

TLC stain: UV, I_2, $KMnO_4$

R_f: 0.6 (30% EtOAc in pet-ether)

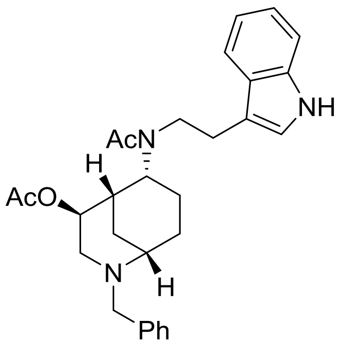

Chemical Formula: $C_{29}H_{35}N_3O_3$
Exact Mass: 473.27
Molecular Weight: 473.62

6-(N-(2-(1H-indol-3-yl)ethyl)acetamido)-2-benzyl-2-azabicyclo[3.3.1]nonan-4-yl acetate (11b): The bicyclic ketone **10** (0.5 g; 1.74 mmol) was added to the slurry of $NaBH(OEh)_3$ (1.21 g; 2.61 mmol) and MS (0.5 g) in dry DCM (5 mL) followed by tryptamine (0.31 g; 1.91 mmol) was added. Then it was allowed to stir for 24 h. at rt. After completion of reaction was confirmed by TLC, diluted with DCM and saturated aq. $NaHCO_3$ was added, stirred for addition 15 min at room temp. The DCM layer was washed with couple of times with saturated aq. $NaHCO_3$ then water and brine solution. After concentration the crude residue was filtered through silica column to get the mixture of diastereomers (>10:1) 0.53 g (71% yield).

TLC stain: I_2, $KMnO_4$; R_f: 0.4 (10% MeOH (Buffered with aq. NH_3) in DCM).

The amine (0.5 g; 1.15 mmol) in dry DCM (5 mL) was added Et_3N (0.14 g; 1.39 mmol), followed by Ac_2O (0.13 g; 1.27 mmol) dropwise at 0 °C and DMAP, and stirred for 4 h. at rt. After completion of reaction, the reaction mixture was diluted with DCM, washed with couple of times with saturated aq. $NaHCO_3$ then water and brine solution. After concentration the crude residue was purified by column chromatography using 2-5% of EtOAc in pet-ether as an eluent to give the pure acetate **(11b)** 0.44 g (81% yield).; ^{1}H NMR (500 MHz, $CDCl_3$) rotamers: δ 8.29 (s, 1H), 8.14 (s, 0.5H), 7.85 – 7.77 (m, 0. H), 7.59 (dd, *J* = 14.5, 6.2 Hz, 1H), 7.45 – 7.30 (m, 7H), 7.28 – 7.16 (m, 4H), 7.08 (dd, *J* = 8.9, 2.1 Hz, 1H), 5.28 (dd, *J* = 35.3, 28.2 Hz, 1H), 4.84 (s, 1H), 3.96 – 3.56 (m, 5H), 3.36 – 3.19 (m, 1H), 3.12 – 2.80 (m,6H), 2.75 – 2.58 (m, 2H), 2.54 – 2.32 (m, 4H), 2.23 – 2.18 (m, 5H), 2.09 – 2.04 (m, 3H), 1.95 – 1.87 (m, 6H).; HPLC-MS (ESI) m/z calcd. for $C_{29}H_{34}N_3O_3$ $[M+H]^+$ = 474.27; Found: 474.44.

TLC stain: UV, I_2, KMnO4

R_f: 0.5 (10% MeOH in DCM)

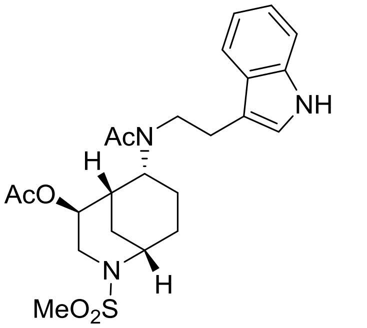

Chemical Formula: $C_{23}H_{31}N_3O_5S$
Exact Mass: 461.20
Molecular Weight: 461.58

6-(N-(2-(1H-indol-3-yl)ethyl)acetamido)-2-(methylsulfonyl)-2-azabicyclo[3.3.1]nonan-4-yl acetate (13b): The diacetate **11b** (0.3 g; 0.63 mmol) was dissolved in EtOAc and carefully 10% Pd/C was added under argon atmosphere. Then the reaction mixture was evacuated couple of times by applying vacuum and re-filling the hydrogen, stirred under H_2 atmosphere for overnight. After complete consumption SM, it was filtered through HPLC filter using syringe, washed couple of times with MeOH and concentrated to give pure product 210 mg (86% yield). TLC stain: UV, I_2, $KMnO_4$; R_f: 0.1 (10% MeOH (Buffered with aq. NH_3) in DCM).

Amine (0.05 g; 0.13 mmol) was dissolved in dry DCM was added Et_3N (0.02 g; 0.2 mmol), followed by MsCl (0.02 g; 0.012 mmol) in one portion at 0 °C, after 4h at room temp., it was then diluted with DCM, washed with couple of times with saturated aq. $NaHCO_3$ then water, brine solution and dried over Na_2SO_4. Then concentrated to give crude residue was purified by column chromatography using 2-5% of MeOH (Buffered with aq. NH_3) in DCM as an eluent to give the pure sulphonamide **(13)** 52 mg (86 % yield). ^{1}H NMR (500 MHz, $CDCl_3$) δ 8.29 (s, 1H), 7.47 (d, *J* = 7.9 Hz, 1H), 7.34 (d, *J* = 8.1 Hz, 1H), 7.18 – 7.14 (m, 1H), 7.11 – 7.06 (m, 1H), 6.97 (s, 1H), 5.08 – 4.97 (m, 1H), 4.79 – 4.69 (m, 1H), 3.99 – 3.91 (m, 1H), 3.85 – 3.77 (m, 1H), 3.64 – 3.55 (m, 4H), 3.44 – 3.32 (m, 1H), 3.00 – 2.79 (m, 4H), 2.60 (dd, *J* = 10.3, 6.5 Hz, 1H), 2.45 – 2.20 (m, 2H), 2.15 (d, *J* = 6.0 Hz, 3H), 2.06 – 1.96 (m, 2H), 1.92 – 1.71 (m, 5H).; ^{13}C NMR (126 MHz, $CDCl_3$) δ 171.2, 169.3, 136.3, 127.0, 122.4, 121.9, 119.8, 118.2, 112.2, 111.6, 70.7, 55.1, 52.6, 47.6, 45.8, 39.4, 33.3, 32.4, 31.9, 27.8, 25.4, 22.3, 21.1.; HPLC-MS (ESI) m/z calcd. for $C_{23}H_{32}O_5S$ $[M+H]^+$ = 462.20; Found: 462.16.

TLC stain: UV, I_2, $KMnO_4$

R_f: 0.1 (10% MeOH (Buffered with aq. NH_3) in DCM)

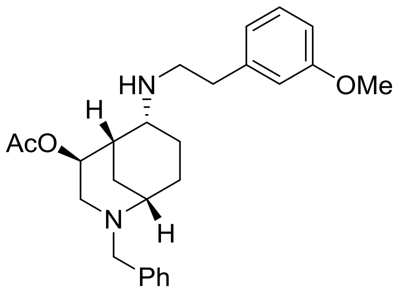

Chemical Formula: $C_{26}H_{34}N_2O_3$
Exact Mass: 422.26
Molecular Weight: 422.57

2-Benzyl-6-(N-(3-methoxyphenethyl)acetamido)-2-azabicyclo[3.3.1]nonan-4-yl acetate (11): The ketone (1.97 g; 6.85 mmol) in dry DCM (50 mL) was added 3-methoxyphenethylamine (1.14 g; 7.54 mmol) (dilution is very important to get diastereo selectivity!!!), MS followed by $NaBH(OEh)_3$ (5.96 g; 10.28 mmol) in one portion. Then it was allowed to stir for 24 h. at rt. After completion of reaction, diluted with DCM and saturated aq. $NaHCO_3$ was added, stirred for additional 15 min at rt. The DCM layer was washed couple of times with saturated aq. $NaHCO_3$ then water and brine solution. After concentration the crude residue was filtered through silica column to get the mixture of diastereomers (>10:1) 1.9 g (66% yield). ^{1}H NMR (400 MHz, $cdcl_3$) δ 7.33 – 7.17 (m, 6H), 6.82 – 6.72 (m, 3H), 5.35 – 5.28 (m, 1H), 3.79 – 3.73 (m, 4H), 3.63 (d, J = 13.4 Hz, 1H), 3.00 – 2.88 (m, 4H), 2.85 – 2.72 (m, 4H), 2.50 (dd, J = 15.3, 11.4 Hz, 2H), 2.33 – 2.25 (m, 1H), 2.18 – 2.09 (m, 1H), 1.95 – 1.87 (m, 4H), 1.67 – 1.56 (m, 2H), 1.40 (tdd, J = 14.0, 7.0, 3.7 Hz, 1H).; ^{13}C NMR (101 MHz, $CDCl_3$) δ 170.2, 159.9, 141.9, 139.2, 129.7, 128.7, 128.5, 127.2, 121.2, 114.8, 111.6, 73.9, 60.7, 59.4, 55.4, 53.1, 49.8, 48.7, 37.2, 34.4, 33.4, 31.2, 25.1, 21.4.; HPLC-MS (ESI) m/z calcd. for $C_{26}H_{35}N_2O_3$ $[M+H]^+$ = 423.26. Found: 423.38.
TLC stain: I_2, $KMnO_4$
R_f: 0.5 (10% MeOH (Buffered with aq. NH_3) in DCM)

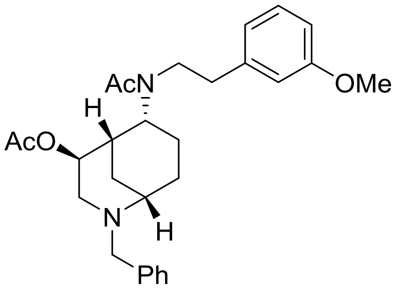

Chemical Formula: $C_{28}H_{36}N_2O_4$
Exact Mass: 464.27
Molecular Weight: 464.61

2-Benzyl-6-(N-(3-methoxyphenethyl)acetamido)-2-azabicyclo[3.3.1]nonan-4-yl acetate (11a): The amine (1.8 g; 4.26 mmol) in dry DCM (23 mL) was added Et_3N (0.52 g; 5.11 mmol), followed by Ac_2O (0.48 g; 4.69 mmol) dropwise at 0 °C and DMAP (0.05 g; 0.426 mmol), and stirred for 4 h. at rt. After completion of reaction, it was diluted with DCM, washed couple of times with saturated aq. $NaHCO_3$ then water and brine solution. After concentration the crude residue was purified by column chromatography using 2-5% of EtOAc:pet-ether as an eluent to give the pure acetate 1.56 g (79% yield).; HPLC-MS (ESI) m/z calcd. for $C_{28}H_{36}N_2O_4$ $[M]^+$ = 464.27; Found: 464.37.

TLC stain: UV, I_2, $KMnO_4$

R_f: 0.7 (10% MeOH in DCM)

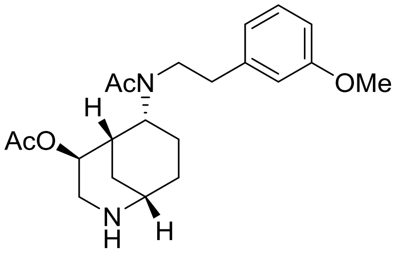

Chemical Formula: $C_{21}H_{30}N_2O_4$
Exact Mass: 374.22
Molecular Weight: 374.48

6-(N-(3-methoxyphenethyl)acetamido)-2-azabicyclo[3.3.1]nonan-4-yl acetate (12a): The diacetate **11a** (1.6 g; 3.79 mmol) was dissolved in EtOAc and carefully 10% Pd/C was added under argon atmosphere. Then the reaction mixture was evacuated couple of times by applying vacuum and re-filling the hydrogen, stirred under H_2 atmosphere for overnight. After complete consumption SM, it was filtered through filter using syringe, washed couple of times with MeOH and concentrated to give pure product **12** 0.95 g (67% yield). Rotamers:

^{1}H NMR (500 MHz, DMSO) δ 7.30 – 7.15 (m, 1H), 6.90 – 6.69 (m, 3H), 5.02 – 4.83 (m, 1H), 4.68 – 4.54 (m, 0.6 H), 4.18 (d, *J* = 11.5 Hz, 0.4H), 3.94 – 3.69 (m, 3H), 3.64 – 3.43 (m, 1H), 3.25 – 3.06 (m, 6H), 2.95 – 2.69 (m, 4H), 2.56 – 2.33 (m, 5H), 2.08 – 1.66 (m, 11H).; ^{13}C NMR (126 MHz, DMSO) δ 170.1, 169.7, 160.0, 159.9, 142.1, 140.9, 130.0, 129.9, 121.3, 121.2, 115.0, 114.7, 112.2, 111.9, 73.6, 73.4, 59.2, 55.8, 55.5, 55.3, 47.8, 46.2, 45.8, 44.6, 44.3, 37.9, 36.7, 35.9, 35.4, 35.3, 33.8, 32.4, 27.3, 26.5, 22.6, 22.1, 21.3.; HPLC-MS (ESI) m/z calcd. for $C_{21}H_{31}N_2O_4$ $[M+H]^+$ = 375.22. Found: 375.27.

TLC stain: UV, I_2, $KMnO_4$

R_f: 0.1 (10% MeOH (Buffered with aq. NH_3) in DCM)

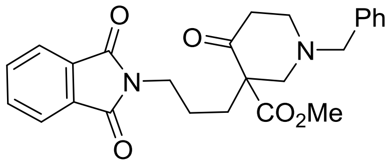

Chemical Formula: $C_{25}H_{26}N_2O_5$
Exact Mass: 434.1842
Molecular Weight: 434.4920

Ethyl 1-benzyl-3-(3-(1,3-dioxoisoindolin-2-yl)propyl)-4-oxopiperidine-3-carboxylate (17): NaH (60% in mineral oil) (3.0 g; 75.2 mmol) was suspended in a mixture of dry THF-DMF (1:1; 200 mL), Keto-ester **15** (10.0 g; 34.2 mmol) dissolved in dry THF was added dropwise at 0 °C, the resulting solution allowed to stir for 10 min to complete deprotonation. Then bromopropyl-pthalimide **16** (11.2 g; 41.02 mmol) in dry THF was added slowly at 0 °C. Then the reaction mixture was fitted with a refluxing condenser and heated at 65 °C for overnight. The reaction completion was monitored by TLC and cooled to room temp and then the pH of the reaction mixture was adjusted to neutral with 1.0M aq. HCl. The layers were separated and aq. layer was extracted with EtOAc, combined organic layer was washed with water, brine solution dried over anhydrous Na_2SO_4. After concentration the crude residue was then purified by column chromatography using 10-15% EtOAc in pet-ether as a gradient to give pure pthalimide derivative **(17)** 9.5 g (64% yield). ^{1}H NMR (400 MHz, $CDCl_3$) δ 7.84 – 7.79 (m, 2H), 7.72 – 7.67 (m, 2H), 7.33 – 7.22 (m, 5H), 3.71 (s, 3H), 3.65 (q, *J* = 6.8 Hz, 2H), 3.55 (d, *J* = 7.8 Hz, 2H), 3.33 (d, *J* = 11.1 Hz, 1H), 2.91 (d, *J* = 49.9 Hz, 2H), 2.37 (d, *J* = 14.5 Hz, 2H), 2.21 (d, *J* = 11.2 Hz, 1H), 1.89 – 1.65 (m, 2H), 1.49 (dtd, *J* = 17.4, 13.0, 5.1

Hz, 2H).; ^{13}C NMR (101 MHz, cdcl$_3$) δ 206.0, 168.5, 134.1, 132.3, 129.0, 128.5, 127.6, 123.4, 61.9, 61.1, 60.6, 53.8, 52.5, 40.6, 38.2, 29.4, 24.1, 21.3, 14.4.; HPLC-MS (ESI) m/z calcd. for $C_{25}H_{27}N_2O_5$ $[M+H]^+$ = 435.18. Found: 435.24.
TLC stain: UV, I_2, $KMnO_4$
R_f: 0.3 (30% EtOAc in pet-ether)

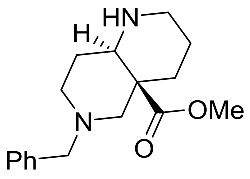

Chemical Formula: $C_{17}H_{24}N_2O_2$
Exact Mass: 288.18
Molecular Weight: 288.39

Methyl 6-benzyloctahydro-1,6-naphthyridine-4a(2H)-carboxylate (18): Pthalimide **(17)** (7.0 g; 15.3 mmol) was dissolved in analytical grade ethanol, was added methyl amine (33% in ethanol) (4.32 g; 45.9 mmol) dropwise at room temp. The reaction mixture was fitted with a refluxing condenser and refluxed at 80 °C for 3h. The reaction completion was monitored by TLC and cooled to room temp (the by-product di-amide precipitated out). The solid di-amide separated by filtration and filtrate was concentrated and filtered through silica column to give pure product 2.99 g (63% yield). TLC stain: I_2, $KMnO_4$; R_f: 0.6 (10% MeOH (Buffered with aq. NH_3) in DCM. The amino-ketone (1.0 g; 3.28 mmol) was dissolved in dry DCM (dilution is very important to get diastereo selectivity!!!) was added equivalent amount of oven dried powdered MS followed by $NaBH(OEh)_3$ (2.29 g; 4.93 mmol) in one portion, then it was allowed to stir for 18 h. at rt. After completion of SM was confirmed by TLC, diluted with DCM and saturated aq. NaHCO3 was added, stirred for addition 15 min at room temp. The DCM layer was washed with couple of times with saturated aq. NaHCO3 then water and brine solution. After concentration the crude residue was filtered through silica column to get the mixture of diastereomers **(18)** (>10:1) 0.691 g (73% yield).; ^{1}H NMR (400 MHz, CD_2Cl_2) δ 7.23 – 7.12 (m, 5H), 3.56 (s, 3H), 3.45 (d, J = 13.4 Hz, 1H), 3.21 (d, J = 13.4 Hz, 1H), 3.05 – 2.94 (m, 2H), 2.84 (ddt, J = 11.0, 4.4, 2.2 Hz, 1H), 2.58 (ddd, J = 12.6, 6.3, 3.6 Hz, 1H), 2.30 (dd, J = 12.1, 4.3 Hz, 1H), 2.09 (ddd, J = 18.5, 9.9, 4.4 Hz, 1H), 2.02 – 1.88 (m, 1H), 1.77 (dd, J = 7.5, 2.9 Hz, 1H), 1.67 – 1.56 (m, 2H), 1.45 – 1.38 (m, 1H), 1.31 – 1.20 (m, 2H).; ^{13}C NMR (101 MHz, CD_2Cl_2) δ 175.2, 139.0, 128.9, 128.1, 127.1, 62.5, 61.9,

61.8, 54.6, 51.4, 47.5, 46.5, 32.5, 29.2, 23.6.; HPLC-MS (ESI) m/z calcd. for $C_{17}H_{25}N_2O_2$ $[M+H]^+$ = 289.18. Found: 289.24.

TLC stain: I_2, $KMnO_4$

R_f: 0.4 (10% MeOH (Buffered with aq. NH_3) in DCM)

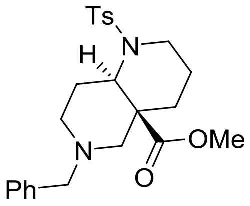

Chemical Formula: $C_{24}H_{30}N_2O_4S$
Exact Mass: 442.19
Molecular Weight: 442.57

Methyl 6-benzyl-1-tosyloctahydro-1,6-naphthyridine-4a(2H)-carboxylate (18a): Amine **18** (0.5 g; 1.73 mmol) was dissolved in dry DCM was added Et_3N (0.21 g; 2.08 mmol), followed by TsCl (0.36 g; 1.90 mmol) in one portion at 0 °C, after 4 h. at rt., it was then diluted with DCM, washed with couple of times with saturated aq. $NaHCO_3$ then water, brine solution and dried over Na_2SO_4. Then concentrated to give crude residue was purified by column chromatography using 2-5% of MeOH (Buffered with aq. NH_3) in DCM as an eluent to give the pure sulphonamide **(18a)** 674 mg (88 % yield).; ^1H NMR (400 MHz, CD_2Cl_2) δ 7.59 – 7.55 (m, 1H), 7.24 (d, *J* = 8.0 Hz, 1H), 7.19 (ddd, *J* = 7.4, 4.4, 1.5 Hz, 1H), 7.16 – 7.09 (m, 2H), 4.06 – 3.98 (m, 1H), 3.58 (s, 2H), 3.43 – 3.36 (m, 1H), 3.14 (d, *J* = 13.4 Hz, 1H), 2.94 (dd, *J* = 11.4, 2.4 Hz, 1H), 2.82 (ddt, *J* = 11.2, 4.8, 2.5 Hz, 1H), 2.59 (ddd, *J* = 25.3, 12.4, 4.7 Hz, 1H), 2.50 – 2.37 (m, 1H), 2.34 (s, 2H), 2.02 (ddd, *J* = 13.1, 7.0, 2.8 Hz, 1H), 1.87 (ddd, *J* = 15.3, 11.7, 5.6 Hz, 1H), 1.62 – 1.39 (m, 2H), 1.03 (td, *J* = 13.3, 4.5 Hz, 1H). ^{13}C NMR (101 MHz, CD_2Cl_2) δ 172.68, 143.38, 138.62, 137.43, 129.72, 128.84, 128.20, 127.40, 127.16, 65.04, 62.39, 62.16, 54.25, 51.38, 50.17, 49.05, 33.62, 28.31, 23.20, 21.41.; HPLC-MS (ESI) m/z calcd. for $C_{24}H_{31}N_2O_4S$ $[M+H]^+$ = 443.19; Found: 443.35.

TLC stain: I_2, $KMnO_4$

R_f: 0.4 (10% MeOH (Buffered with aq. NH_3) in DCM)

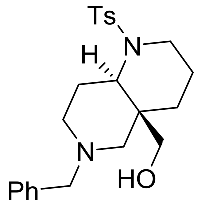

Chemical Formula: $C_{23}H_{30}N_2O_3S$
Exact Mass: 414.20
Molecular Weight: 414.56

6-benzyl-1-tosyloctahydro-1,6-naphthyridin-4a(2H)-yl)methanol (19): To a suspension of LAH (0.043 g; 1.13 mmol) in dry THF was added amino-ester (0.5 g; 1.13 mmol) dissolved in dry THF at 0 °C. Then the reaction mixture was stirred for overnight at rt, after completion of reaction monitored by TLC, the excess LAH was quenched with wet Na_2SO_4!!! (Few drops of water were added to Na_2SO_4 to make paste, which used for quenching). The greyish white solid was filtered washed several times with EtOAc and the filtrate was concentrated to give pure product **(19)** 0.44g, (93% yield).; ^{1}H NMR (400 MHz, CD_2Cl_2) δ 7.55 – 7.50 (m, 2H), 7.26 – 7.14 (m, 7H), 4.20 (dd, *J* = 10.9, 1.5 Hz, 1H), 4.14 – 4.06 (m, 1H), 3.45 (d, *J* = 10.9 Hz, 1H), 3.37 – 3.25 (m, 2H), 2.90 – 2.76 (m, 2H), 2.56 – 2.37 (m, 3H), 2.33 (s, 3H), 1.88 – 1.76 (m, 2H), 1.70 – 1.64 (m, 2H), 1.50 – 1.35 (m, 2H), 0.85 (td, *J* = 13.3, 4.4 Hz, 1H).; ^{13}C NMR (101 MHz, CD_2Cl_2) δ 143.4, 137.7, 137.7, 129.9, 129.1, 128.6, 127.5, 127.1, 66.4, 65.4, 62.8, 62.6, 53.5, 51.2, 38.5, 34.1, 27.5, 21.9, 21.4.; HPLC-MS (ESI) m/z calcd. for $C_{23}H_{31}N_2O_3S$ $[M+H]^+$ = 415.20. Found: 415.41.
TLC stain: UV, I_2, $KMnO_4$
R_f: 0.3 (10% MeOH (Buffered with aq. NH_3) in DCM)

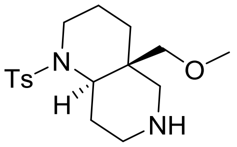

Chemical Formula: $C_{17}H_{26}N_2O_3S$
Exact Mass: 338.17
Molecular Weight: 338.47

4a-(Methoxymethyl)-1-tosyldecahydro-1,6-naphthyridine (20): The ether **19a** (2.4 g; 5.60 mmol) was dissolved in EtOAc:MeOH (1:1) and carefully 10% Pd/C was added under argon atmosphere. Then the reaction mixture was evacuated couple of times by applying vacuum and re-filling the hydrogen, stirred under H_2 atmosphere for overnight. After complete

consumption SM, it was filtered through HPLC filter using syringe, washed couple of times with MeOH and concentrated to give pure product **(20)** 1.6 g (84% yield).; ^{1}H NMR (400 MHz, cdcl$_3$) δ 7.60 (dd, *J* = 8.5, 1.9 Hz, 1H), 7.31 – 7.27 (m, 1H), 4.31 – 4.23 (m, 1H), 3.92 (d, *J* = 9.6 Hz, 1H), 3.59 (d, *J* = 9.6 Hz, 1H), 3.37 (d, *J* = 3.8 Hz, 1H), 3.21 – 3.06 (m, 2H), 2.75 – 2.62 (m, 1H), 2.54 (td, *J* = 12.8, 2.8 Hz, 1H), 2.41 (s, 2H), 2.28 – 2.15 (m, 1H), 1.94 – 1.76 (m, 2H), 1.62 – 1.55 (m, 1H), 0.93 – 0.80 (m, 1H).; ^{13}C NMR (101 MHz, cdcl$_3$) δ 143.3, 137.9, 129.9, 126.9, 70.4, 66.1, 59.7, 53.4, 51.4, 46.3, 38.7, 32.2, 26.8, 22.0, 21.7.; HPLC-MS (ESI) m/z calcd. for $C_{17}H_{27}N_2O_3S$ $[M+H]^+$ = 339.17. Found: 339.32.

TLC stain: UV, I_2, $KMnO_4$

R*f*: 0.2 (10% MeOH (Buffered with aq. NH_3) in DCM)

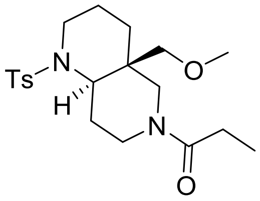

Chemical Formula: $C_{20}H_{30}N_2O_4S$
Exact Mass: 394.19
Molecular Weight: 394.53

1-(-4a-(methoxymethyl)-1-tosyloctahydro-1,6-naphthyridin-6(2H)-yl)propan-1-one (21): Amine **20** (0.1 g; 0.29 mmol) dissolved in dry DCM was added Et_3N (0.04 g; 0.35 mmol), followed by propionyl chloride (0.03 g; 0.33 mmol) dropwise at 0 °C, and stirred for 4 h. at rt. After completion of reaction, the reaction mixture was diluted with DCM, washed couple of times with saturated aq. $NaHCO_3$ then water and brine solution. After concentration the crude residue was purified by column chromatography using 2-5% of MeOH (Buffered with aq. NH_3) in DCM as an eluent to give the pure amide **(21)** 76 mg (57% yield).; ^{1}H NMR (500 MHz, $CDCl_3$) δ 7.54 (d, *J* = 8.1 Hz, 3H), 7.24 (d, *J* = 8.0 Hz, 3H), 4.67 (d, *J* = 13.5 Hz, 1H), 4.23 (t, *J* = 13.5 Hz, 1H), 3.79 (dd, *J* = 13.5, 1.8 Hz, 1H), 3.57 (t, *J* = 8.9 Hz, 1H), 3.25 (d, *J* = 7.0 Hz, 4H), 2.69 – 2.58 (m, 3H), 2.39 – 2.33 (m, 5H), 2.31 – 2.21 (m, 3H), 2.00 – 1.77 (m, 7H), 1.58 (d, *J* = 14.1 Hz, 1H), 1.39 – 1.30 (m, 1H), 1.07 – 1.00 (m, 3H), 0.81 (ddd, *J* = 16.1, 11.2, 5.1 Hz, 2H).; ^{13}C NMR (126 MHz, $CDCl_3$) δ 172.9, 143.4, 137.5, 129.9, 126.8, 67.4, 65.7, 59.1, 51.7, 51.0, 41.3, 39.7, 30.2, 29.7, 25.7, 25.6, 21.6, 21.5, 9.4.; HPLC-MS (ESI) m/z calcd. for $C_{20}H_{31}N_2O_4S$ $[M+H]^+$ = 395.19. Found: 395.30.

TLC stain: UV, I_2, KMnO4

R$_f$: 0.7(10% MeOH (Buffered with aq. NH_3) in DCM

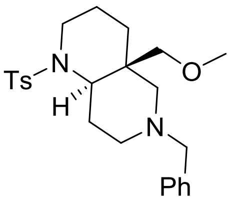

Chemical Formula: $C_{24}H_{32}N_2O_3S$
Exact Mass: 428.21
Molecular Weight: 428.59

6-Benzyl-4a-(methoxymethyl)-1-tosyldecahydro-1,6-naphthyridine (22): To a solution of amine **20** (45 mg; 0.13 mmol) in dry DCM (3 mL) was added benzaldehyde (16 µL; 0.16 mmol) dropwise at 0 °C. After 10 min $NaBH(OAc)_3$ (34 mg; 0.16 mmol) was added in one portion and stirred for 2 h. at rt. The reaction mixture was diluted with DCM and saturated aq. $NaHCO_3$ was added slowly, and then stirred for additional 15 min at rt. The layers were separated, the DCM layer was washed couple of times with saturated aq. $NaHCO_3$, water, brine solution and dried over anhydrous Na_2SO_4. After concentration the crude residue was filtered through silica column to afford pure compound (**22)** 43 mg (76% yield).; ^{1}H NMR (400 MHz, $CDCl_3$) δ 7.59 (d, *J* = 8.3 Hz, 1H), 7.33 – 7.18 (m, 4H), 4.28 – 4.21 (m, 1H), 4.00 (d, *J* = 8.8 Hz, 1H), 3.69 (d, *J* = 8.8 Hz, 1H), 3.45 – 3.29 (m, 3H), 2.90 (t, *J* = 13.2 Hz, 1H), 2.62 (td, *J* = 12.5, 2.7 Hz, 1H), 2.49 (d, *J* = 9.1 Hz, 1H), 2.41 (s, 2H), 2.18 (dd, *J* = 29.5, 14.7 Hz, 1H), 1.98 – 1.78 (m, 2H), 1.59 – 1.52 (m, 1H), 1.45 – 1.37 (m, 1H), 0.96 – 0.72 (m, 1H).; ^{13}C NMR (101 MHz, $CDCl_3$) δ 143.18, 137.66, 129.88, 128.81, 128.29, 127.07, 69.73, 66.28, 62.41, 60.71, 59.52, 53.67, 51.48, 39.67, 31.24, 27.11, 21.86, 21.69.; HPLC-MS (ESI) m/z calcd. for $C_{24}H_{33}N_2O_3S$ $[M+H]^+$ = 429.21; Found: 429.46.
TLC stain: UV, I_2, $KMnO_4$
R$_f$: 0.6 (30% EtOAc in Pet-Ether)

Experimental for the production phase of Elaeokanidine-A based scaffolds:

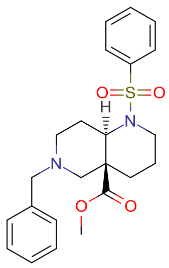

Methyl 1-(benzenesulfonyl)-6-benzyl-decahydro-1,6-naphthyridine-4a-carbo-xylate (46a): To a solution of the amine **18** (27.0 g, 94 mmol) in dry DCM (270 mL) at 0 °C was added triethylamine (28.4 g, 282 mmol) and benzenesulfonyl chloride (18.4 g, 103 mmol) and the reaction mixture was allowed to warm up to room temperature. After the complete consumption of the starting material monitored by TLC, the reaction mixture was washed with saturated aq. $NaHCO_3$ and saturated aq. NH_4Cl. After concentration, the crude residue was purified by column chromatography using 1-3% of MeOH, affording the sulfonamide 29.9 g (75% yield).; ^1H NMR (300 MHz, $CDCl_3$): δ 7.85 (d, J = 6.6 Hz, 2H), 7.57 – 7.48 (m, 3H), 7.30 – 7.25 (m, 3H), 7.21 – 7.19 (m, 2H), 4.21 (d, J = 11.9 Hz, 1H), 3.72 (s, 3H), 3.70 - 3.69 (m, 1H), 3.48 (d, J = 13.4 Hz, 1H), 3.28 (d, J = 13.4 Hz, 1H), 3.10 (dd, J = 2.2, 11.3 Hz, 1H), 2.90 (d, J = 15.2 Hz, 1H), 2.72 – 2.62 (m, 2H), 2.13 – 2.10 (m, 1H), 2.06 – 2.02 (m, 1H), 2.01 – 1.93 (m, 1H), 1.72 (d, J = 11.4 Hz, 1H), 1.66 – 1.58 (m, 1H), 1.16 (dt, J = 4.9, 12.9 Hz, 1H), 0.91 – 0.88 (m, 1H).; UHPLC-MS (ES+APCI) m/z calcd. for $C_{23}H_{29}N_2O_4S$ $[M]^+$ = 429.54. Found: 428.90.

TLC stain: UV, I_2, $KMnO_4$

R_f: 0.4 (Cyclohexane/EtOAc 7:1)

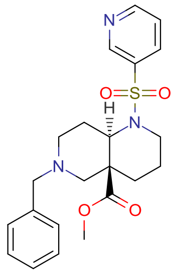

Methyl 6-benzyl-1-(pyridine-3-sulfonyl)-decahydro-1,6-naphthyridine-4a-carboxylate (46b): To a solution of the amine **18** (27.0 g, 94 mmol) in dry DCM (270 mL) at 0 °C was added triethylamine (28.4 g, 282 mmol) and pyridine-3-sulfonyl chloride (18.3 g, 103 mmol) and the reaction mixture was allowed to warm up to room temperature. After the complete consumption of the starting material monitored by TLC, the reaction mixture was washed with saturated aq. $NaHCO_3$ and saturated aq. NH_4Cl. After concentration, the crude residue was purified by column chromatography using 10-15% ethyl acetate in cyclohexane, affording the sulfonamide 28.3 g (70% yield).; ^1H NMR (300 MHz, $CDCl_3$): δ 8.97 (d, J = 1.9 Hz, 1H), 8.70 (d, J = 4.9 Hz, 1H), 8.07 (dd, J = 1.9, 8.0 Hz, 1H), 7.38 (dd, J = 4.9, 8.0 Hz, 1H), 7.20 – 7.08 (m, 5H), 4.13 (d, J = 12.0 Hz, 1H), 3.59 (s, 3H), 3.39 (d, J = 13.4 Hz, 1H),

3.17 (d, J = 13.4 Hz, 1H), 3.01 (dd, J = 1.8, 11.4 Hz, 1H), 2.81 (dd, J = 1.7, 9.6 Hz, 1H), 2.68 – 2.60 (m, 2H), 2.55 (dq, J = 4.5, 12.3 Hz, 1H), 2.04 – 1.86 (m, 3H), 1.65 – 1.42 (m, 3H), 1.10 (dt, J = 4.2, 13.3 Hz, 1H).; UHPLC-MS (ES+APCI) m/z calcd. for $C_{22}H_{28}N_3O_4S$ $[M]^+$ = 430.53. Found: 429.95.

TLC stain: UV, I_2, $KMnO_4$

R_f: 0.5 (Cyclohexane/EtOAc 5:1)

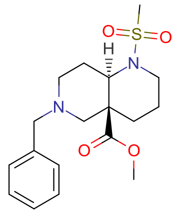

Methyl 6-benzyl-1-methanesulfonyl-decahydro-1,6-naphthyridine-4a-carbo-xylate (46c): To a solution of the amine **18** (35.0 g, 121 mmol) in dry DCM (350 mL) at 0 °C was added triethylamine (14.8 g, 146 mmol) and methanesulfonyl chloride (15.3 g, 134 mmol) and the reaction mixture was allowed to warm up to room temperature. After the complete consumption of the starting material monitored by TLC, the reaction mixture was washed with saturated aq. $NaHCO_3$ and saturated aq. NH_4Cl. After concentration, the crude residue was purified by column chromatography using 1-3% of MeOH, affording the sulfonamide 12.7 g (29 % yield).; ^1H NMR (300 MHz, $CDCl_3$): δ 7.33 – 7.21 (m, 5H), 4.13 (d, J = 12.4 Hz, 1H), 3.68 (s, 3H), 3.55 (d, J = 13.4 Hz, 1H), 3.29 (d, J = 13.4 Hz, 1H), 3.12 (dd, J = 2.3, 11.4 Hz, 1H), 3.04 (s, 3H), 3.02 – 3.01 (m, 1H), 2.85 – 2.81 (m, 2H), 2.70 (dt, J = 2.6, 12.3 Hz, 1H), 2.49 -2.40 (m, 1H), 2.16 – 2.01 (m, 2H), 1.78 (d, J = 11.4 Hz, 1H), 1.66 – 1.59 (m, 1H), 1.53 – 1.40 (m, 1H), 1.21 (dt, J = 4.0, 13.4 Hz, 1H).; UHPLC-MS (ES+APCI) m/z calcd. for $C_{18}H_{27}N_2O_4S$ $[M]^+$ = 367.48. Found: 366.95.

TLC stain: I2, $KMnO_4$

R_f: 0.5 (2% MeOH (Buffered with aq. NH_3) in DCM)

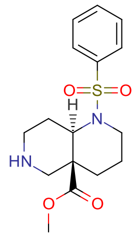

Methyl 1-(benzenesulfonyl)-decahydro-1,6-naphthyridine-4a-carboxylate (47a): To a solution of the sulfonamide **46a** (5.0 g, 12 mmol) in dry 1,2-dichloroethane (120 mL) was added 1-chloroethyl chloroformate (4.2 g, 30 mmol) and the reaction mixture was refluxed for 15 hours. After complete consumption of the starting material, to the reaction mixture was added methanol and it was refluxed for 3 more hours. After concentration, the crude residue was purified by column chromatography using 5-10% methanol in DCM as eluent to give the pure 2.3 g (58% yield).; ^{1}H NMR (300 MHz, MeOD-d_4): δ 7.82 – 7.53 (m, 2H), 7.64 – 7.53 (m, 3H), 4.25 – 4.02 (m, 1H), 3.83 (s, 3H), 3.54 – 3.43 (m, 2H), 2.97 – 2.75 (m, 4H), 2.23 – 2.11 (m, 2H), 1.69 – 1.49 (m, 2H), 1.27 – 1.24 (m, 1H).; UHPLC-MS (ES+APCI) m/z calcd. for $C_{16}H_{22}N_2O_4S$ $[M]^+$ = 339.42. Found: 338.9.

TLC stain: UV, I_2, $KMnO_4$

R_f: 0.3 (10% MeOH (Buffered with aq. NH_3) in DCM)

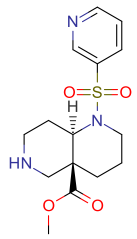

Methyl 1-(pyridine-3-sulfonyl)-decahydro-1,6-naphthyridine-4a-carboxylate (47b): To a solution of the sulfonamide **46b** (5.0g, 12 mmol) in dry 1,2-dichloroethane (120 mL) was added 1-chloroethyl chloroformate (4.2 g, 30 mmol) and the reaction mixture was refluxed for 15 hours. After complete consumption of the starting material, to the reaction mixture was added methanol and it was refluxed for 3 more hours. After concentration, the crude residue was purified by column chromatography using 5-10% methanol in DCM as eluent to give the pure 3.2 g (81% yield).; ^{1}H NMR (300 MHz, MeOD-d_4): δ 8.86 (s, 1H), 8.69 – 8.71 (m, 1H), 8.12 (d, J = 7.6 Hz, 1H), 7.56 (t, J = 7.6 Hz, 1H), 4.09 (d, J = 14.8 Hz, 1H), 3.74 (s, 3H), 3.46 (t, J = 11.7Hz, 2H), 3.36 (d, J = 12.7Hz, 2H), 3.00 – 2.86 (m, 3H), 2.63 (q, J = 11.9 Hz, 1H), 2.02 (d, J = 12.9 Hz, 3H), 1.62 (d, J = 14.4 Hz, 1H), 1.41 (dt, J = 4.2, 14.4 Hz, 1H).; UHPLC-MS (ES+APCI) m/z calcd. for $C_{15}H_{22}N_3O_4S$ $[M]^+$ = 340.41. Found: 339.95.

TLC stain: UV, I_2, $KMnO_4$

R_f: 0.3 (15% MeOH (Buffered with aq. NH_3) in DCM)

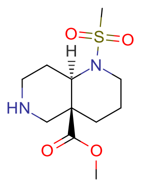

Methyl (4aS,8aR)-1-methanesulfonyl-decahydro-1,6-naphthyridine-4a-carboxylate (47c): To a solution of the sulfonamide **46c** (4.0 g, 11 mmol) in dry methanol (110 mL) was added palladium on charcoal (10 wt%) and the reaction mixture was stirred under hydrogen atmosphere overnight. After complete consumption of the starting material monitored by TLC, the reaction mixture was filtered through a pad of celite. Concentration of the filtrate afforded the pure product 3.0 g (99% yield).; ^{1}H NMR (300 MHz, MeOD-d_4): δ 3.93 (d, J = 12.2 Hz, 1H), 3.71 (s, 3H), 3.21 – 3.06 (m, 3H), 2.86 (s, 3H), 2.81 (dd, J = 3.4, 12.0 Hz, 1H), 7.22 (dd, J = 3.0, 17.5 Hz, 1H), 2.65 – 2.60 (m, 1H), 2.55 (dd, J = 3.8, 11.9 Hz, 1H), 2.46 (d, J = 13.3 Hz, 1H), 2.03 (d, J = 13.3 Hz, 2H), 1.77 – 1.61 (m, 2H), 1.27 (dt, J = 4.6, 13.3 Hz, 1H).; UHPLC-MS (ES+APCI) m/z calcd. for $C_{11}H_{21}N_2O_4S$ $[M]^+$ = 277.35. Found: 277.10.

TLC stain: UV, I_2, $KMnO_4$

R_f: 0.3 (15% MeOH (Buffered with aq. NH_3) in DCM)

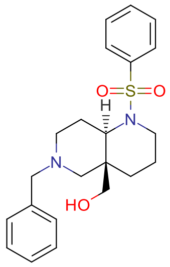

1-(Benzenesulfonyl)-6-benzyl-decahydro-1,6-naphthyridin-4a-yl]methanol (49a): To a suspension of lithium aluminium hydride (1.7 g, 45 mmol) in dry THF (225 mL) at 0 °C was added dropwise a solution of the ester **46a** (19.2 g, 45 mmol) in dry THF (225 mL) and then the reaction mixture was left at room temperature. After complete consumption of the starting material monitored by TLC, the reaction mixture was cooled down to 0 °C and was quenched with *i*-propanol and water. The reaction mixture was then dried with excess amount of $MgSO_4$ and concentrated in vacuo affording the product 13.0 g (72% yield).; ^{1}H NMR (300 MHz, $CDCl_3$): δ 7.68 (d, J = 7.5 Hz, 2H), 7.48 – 7.40 (m, 3H), 7.21 – 7.15 (m, 5H), 4.27 (d, J = 10.9 Hz, 1H), 4.19 (d, J = 11.7 Hz, 1H), 3.52 (d, J = 10.9 Hz, 1H), 3.33 (d, J = 5.6 Hz, 2H), 2.86 (d, J = 10.6 Hz. 2H), 2.61 – 2.48 (m, 2H), 1.91 – 1.81 (m, 2H), 1.72 (d, J = 11.8 Hz, 2H),

1.49 – 1.41 (m, 2H), 1.20 (d, J = 8.1 Hz, 1H), 0.92 – 0.74 (m, 2H).; UHPLC-MS (ES+APCI) m/z calcd. for $C_{22}H_{29}N_2O_3S$ $[M]^+$ = 401.53. Found: 401.00.

TLC stain: UV, I_2, $KMnO_4$

R_f: 0.3 (10% MeOH (Buffered with aq. NH_3) in DCM)

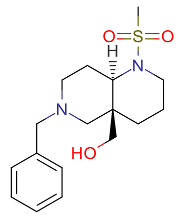

6-Benzyl-1-methanesulfonyl-decahydro-1,6-naphthyridin-4a-yl]methanol (49c): To a suspension of lithium aluminium hydride (0.8 g, 20 mmol) in dry THF (100 mL) at 0 °C was added dropwise a solution of the ester **46c** (7.2 g, 20 mmol) in dry THF (100 mL) and then the reaction mixture was left at room temperature. After complete consumption of the starting material monitored by TLC, the reaction mixture was cooled down to 0 °C and was quenched with *i*-propanol and water. . The reaction mixture was then dried with excess amount of $MgSO_4$ and concentrated in vacuo affording the product 6.7 g (quant.).; ^{1}H NMR (300 MHz, $CDCl_3$): δ 7.30 – 7.28 (m, 5H), 4.32 (d, J = 11.0 Hz, 1H), 4.10 – 3.98 (m, 2H), 3.58 (d, J = 10.7 Hz, 1H), 3.46 (s, 3H), 3.06 – 2.95 (m, 2H), 2.85 – 2.76 (s, 3H; m, 1H), 2.64 (t, J = 12.3 Hz, 2H), 2.05 – 2.01 (m, 2H), 1.89 (d, J = 11.6 Hz, 1H), 1.63 – 1.50 (m, 2H), 1.02 (dt, J = 3.3, 13.3 Hz, 1H).; UHPLC-MS (ES+APCI) m/z calcd. for $C_{17}H_{27}N_2O_3S$ $[M]^+$ = 339.47. Found: 339.05.

TLC stain: UV, I_2, $KMnO_4$

R_f: 0.4 (5% MeOH (Buffered with aq. NH_3) in DCM)

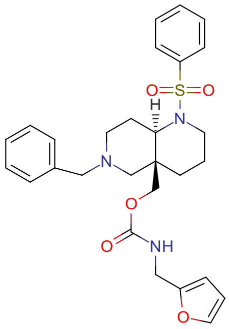

[(4aS,8aR)-1-(benzenesulfonyl)-6-benzyl-decahydro-1,6-naphthyridin-4a-yl]methyl N-[(furan-2-yl)methyl]carbamate (50a): To a solution of the alcohol **49a** (6.6 g, 16 mmol)

in dry DCM (100 mL) was added triethylamine (2.5 g, 24 mmol) and furfuryl isocyanate (2.4 g, 19 mmol) and the reaction mixture was stirred at room temperature for 15 hours. After the complete consumption of the starting material monitored by TLC, the reaction mixture was washed with saturated aq. $NaHCO_3$ and brine. After concentration, the crude residue was purified by column chromatography using 15-20% of EtOAc in cyclohexane as an eluent to give the carbamate 3.7 g (43% yield).; ^{1}H NMR (300 MHz, $CDCl_3$): δ 7.68 (d, J = 7.2 Hz, 2H), 7.49 – 7.41 (m, 3H), 7.19 – 7.09 (m, 6H), 6.22 (m, 2H), 4.64 – 4.38 (m, 2H), 4.40 (d, J = 10.6 Hz, 1H), 4.29 – 4.28 (m, 1H), 4.18 (d, 13.1 Hz, 1H), 3.43 (d, J = 13.2 Hz, 1H), 3.17 (d, J = 13.2 Hz, 1H), 2.87 (d, J = 8.8 Hz, 1H), 2.64 – 2.50 (m, 3H), 2.10 (dq, J = 2.0, 13.1 Hz, 1H), 1.93 – 1.81 (m, 3H), 1.60 – 1.48 (m, 3H), 1.19 (t, J = 7.2 Hz, 1H), 0.78 (dt, J = 3.5, 13.2 Hz, 1H).; UHPLC-MS (ES+APCI) m/z calcd. for $C_{28}H_{34}N_3O_5S$ $[M]^+$ = 524.64. Found: 523.85.
TLC stain: UV, I_2, $KMnO_4$
R_f: 0.4 (Cyclohexane/EtOAc 2:1)

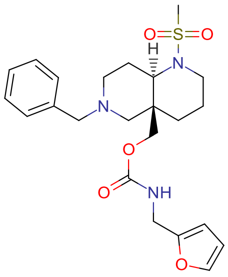

[(4aS,8aR)-6-benzyl-1-methanesulfonyl-hexahydro-2H-1,6-naphthyridin-4a-yl]methyl N-(furan-2-ylmethyl)carbamate (50c): To a solution of the alcohol **49c** (2.8 g, 8 mmol) in dry DCM (50 mL) was added triethylamine (1.2 g, 12 mmol) and furfuryl isocyanate (1.2 g, 10 mmol) and the reaction mixture was stirred at room temperature for 15 hours. After the complete consumption of the starting material monitored by TLC, the reaction mixture was washed with saturated aq. $NaHCO_3$ and brine. After concentration, the crude residue was purified by column chromatography using 2% MeOH in DCM as an eluent to give the carbamate 3.6 g (94% yield).; ^{1}H NMR (300 MHz, $CDCl_3$): δ 7.35 – 7.16 (m, 6H), 6.31 – 6.22 (m, 2H), 4.74 – 4.71 (m, 1H), 4.43 – 4.37 (m, 1H), 4.33 (d, J = 5.2 Hz, 2H), 4.04 (d, J = 11.9 Hz, 1H), 3.56 (d, J = 13.3 Hz, 1H), 3.28 (d, J = 12.9 Hz, 1H), 3.05 – 3.02 (m, 1H), 2.77 (s, 3H), 2.75 – 2.71 (m, 1H), 2.60 (dt, J = 2.8, 12.5 Hz, 1H), 2.28 (dq, J = 3.9, 12.5 Hz, 1H), 2.10 – 1.86 (m, 3H), 1.67 (d, J = 12.9 Hz, 1H), 1.56 – 1.43 (m, 2H), 1.12 (t, J = 7.1 Hz, 1H),

0.94 – 0.83 (m, 1H).; UHPLC-MS (ES+APCI) m/z calcd. for $C_{23}H_{32}N_3O_5S$ $[M]^+$ = 462.57. Found: 461.90.

TLC stain: UV, I_2, $KMnO_4$

R_f: 0.4 (2% MeOH (Buffered with aq. NH_3) in DCM)

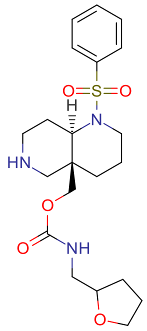

1-(Benzenesulfonyl)-decahydro-1,6-naphthyridin-4a-yl]methyl N-[(oxolan-2-yl)methyl]carbamate (51a): To a solution of the carbamate **50a** (3.7 g, 7 mmol) in dry methanol (70 mL) was added palladium on charcoal (10 wt%) and the reaction mixture was stirred under hydrogen atmosphere overnight. After complete consumption of the starting material monitored by TLC, the reaction mixture was filtered through a pad of celite. Concentration of the filtrate afforded the pure product 3.0 g (96% yield).; ^1H NMR (300 MHz, $CDCl_3$): δ 7.71 (d, J = 7.4 Hz, 2H), 7.55 – 7.47 (m, 3H), 4.76 (t, J = 10.5 Hz, 1H), 4.27 (d, J = 11.4 Hz, 2H), 3.9 (bs, 1H), 3.82 (dd, J = 7.2, 13.9 Hz, 1H), 3.72 (dd, J = 7.2, 13.9 Hz, 1H), 3.44 – 3.88 (m, 2H), 3.18 – 3.13 (m, 2H), 2.87 – 2.66 (m, 5H), 2.53 (t, J = 12.7 Hz, 1H), 2.16 (d, J = 13.5 Hz, 1H), 2.11 – 1.80 (m, 6H), 1.73 (d, J = 13.1 Hz, 1H), 1.64 – 1.50 (m, 2H), 0.92 (t, J = 10.6 Hz, 1H).; UHPLC-MS (ES+APCI) m/z calcd. for $C_{21}H_{32}N_3O_5S$ $[M]^+$ = 438.55. Found: 437.90.

TLC stain: UV, I_2, $KMnO_4$

R_f: 0.3 (10% MeOH (Buffered with aq. NH_3) in DCM)

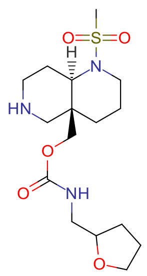

1-Methanesulfonyl-octahydro-1,6-naphthyridin-4a-yl]methyl N-(oxolan-2-yl-methyl)carbamate (51c): To a solution of the carbamate **50c** (4.4 g, 9 mmol) in dry methanol (90 mL) was added palladium on charcoal (10 wt%) and the reaction mixture was stirred under hydrogen atmosphere overnight. After complete consumption of the starting material monitored by TLC, the reaction mixture was filtered through a pad of celite. After concentration, the crude residue was purified by column chromatography using 10% MeOH in DCM as an eluent to give the pure product 1.0 g (28% yield).; ^{1}H NMR (300 MHz, MeOD-d_4): δ 4.43 (g, J = 10.0 Hz, 2H), 3.99 – 3.90 (m, 2H), 3.87 – 3.79 (m, 1H), 3.71 (q, J = 7.1 Hz, 1H), 3.51 – 3.45 (m, 2H), 3.41 – 3.28 (m, 2H), 3.30 – 3.28 (m, 1H), 3.21 – 3.09 (m, 3H), 2.91 (s, 3H), 2.86 – 2.81 (m, 2H), 2.41 – 2.23 (m, 2H), 1.99 – 1.79 (m, 5H), 1.68 – 1.54 (m, 2H), 1.29 – 1.27 (m, 1H); UHPLC-MS (ES+APCI) m/z calcd. for $C_{16}H_{30}N_3O_5S$ $[M]^+$ = 376.48. Found: 376.00.
TLC stain: UV, I_2, $KMnO_4$
R_f: 0.4 (10% MeOH (Buffered with aq. NH_3) in DCM)

General procedures for final diversification steps:

General procedure for the amide coupling: Reactions were performed in parallel in 15 ml reaction tubes in a 24 position Mettler-Toledo Miniblock® equipped with a heat transfer block and inert gas manifold. Each reaction tube was loaded with TEA (5.0 eq) and a previously prepared solution of 30 mg of the corresponding intermediate in 1mL of dry THF. Then the corresponding acyl chlorides (1.5 eq) were added. The reactions were stirred at room temperature overnight. The mixtures were evaporated until dryness. The crudes were rediluted in 1.0 mL of ACN, filtered and purified with preparative HPLC.

General procedure for reductive amination: Reactions were performed in parallel in 15 ml reaction tubes in a 24 position Mettler-Toledo Miniblock® equipped with a heat transfer block and inert gas manifold. Each reaction tube was loaded with the corresponding aldehyde

(2.0 eq), $NaBH(OAc)_3$ (2.5 eq.), acetic acid (2.0 eq) and 1.0 mL of 1,2-dichloroethene (DCE). The reaction mixture was stirred for 30 min, then a previously prepared solution of 30 mg of the corresponding intermediate in 1.0 mL DCE was added. The reactions were stirred at room temperature overnight. The mixtures were diluted with dichloromethane (2.0 mL) and washed with water (1.0 mL). The organic layers were evaporated until dryness. The crudes were rediluted in 1.0 mL of acetonitrile, filtered and purified with preparative HPLC.

General procedure for sulfonylation: Reactions were performed in parallel in 15 ml reaction tubes in a 24 position Mettler-Toledo Miniblock® equipped with a heat transfer block and inert gas manifold. Each reaction tube was loaded with TEA (5.0 eq) and a previously prepared solution of 30 mg of the corresponding intermediate in 1mL of dry THF. Then the corresponding sulfonyl chlorides (1.5 eq) were added. The reactions were stirred at room temperature overnight. The mixtures were evaporated until dryness. The crudes were rediluted in 1.0 mL of ACN, filtered and purified with preparative HPLC.

General procedure for urea formation: Reactions were performed in parallel in 15 ml reaction tubes in a 24 position Mettler-Toledo Miniblock® equipped with a heat transfer block and inert gas manifold. Each reaction tube was loaded with TEA (5.0 eq) and a previously prepared solution of 30 mg of the corresponding intermediate in 1mL of DCM. Then the corresponding isocyanates (1.5 eq) were added. The reactions were stirred at room temperature overnight. The mixtures were evaporated until dryness. The crudes were rediluted in 1.0 mL of ACN, filtered and purified with preparative HPLC

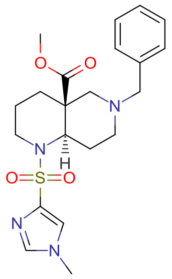

Methyl 6-benzyl-1-[(1-methyl-1H-imidazol-4-yl)sulfonyl]-decahydro-1,6-naphthyridine-4a-carboxylate (46d): 10.2 mg (23% yield).; ^{1}H NMR (300 MHz, $CDCl_3$): δ 7.45 (d, J = 1.4 Hz, 1H), 7.39 (d, J = 1.4 Hz, 1H), 7.29 – 7.26 (m, 5H), 4.14 (dt, J = 3.0, 11.8 Hz, 1H), 3.74 (s, 3H), 3.73 (s, 3H), 3.65 (d, J = 13.4 Hz, 1H), 3.42 (d, J = 13.4 Hz, 1H), 3.14

(dd, J = 2.1, 11.6 Hz, 1H), 3.07 (d, J = 3.6 Hz, 1H), 2.88 (dt, J = 4.3, 12.6 Hz, 1H), 2.78 – 2.67 (m, 2H), 2.37 (dd, J = 3.1, 12.8 Hz, 1H), 2.16 (t, J = 9.9 Hz, 1H), 2.02 (d, J = 12.8 Hz, 1H), 1.92 (d, J = 11.4 Hz, 1H), 1.67 – 1.59 (m, 2H), 1.21 – 1.11 (m, 1H).; UHPLC-MS (ES+APCI) m/z calcd. for $C_{21}H_{29}N_4O_4S$ [M]+ = 433.54. Found: 433.20.

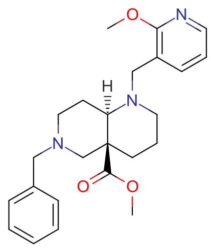

Methyl 6-benzyl-1-[(2-methoxypyridin-3-yl)methyl]-decahydro-1,6-naphthyridine-4a-carboxylate (46e): 11.5 mg (27% yield).; ^{1}H NMR (300 MHz, $CDCl_3$): δ 8.14 (dd, J = 1.8, 5.0 Hz, 1H), 8.01 (dd, J = 1.7, 7.3 Hz, 1H), 7.31 – 7.20 (m, 4H), 6.96 (dd, J = 5.1, 7.3 Hz, 2H), 4.33 (d, J = 14.4 Hz, 1H), 4.12 (d, J = 14.4 Hz, 1H), 4.04 (s, 3H), 3.72 (s, 3H), 3.55 (d, J = 13.3 Hz, 1H), 3.32 (d, J = 13.3 Hz, 1H), 3.27 – 3.25 (m, 1H), 3.14 (dd, J = 2.2, 11.5 Hz, 1H), 3.08 (d, J = 11.5 Hz, 1H), 2.99 (dd, J = 1.6, 10.1 Hz, 2H), 2.30 – 1.45 (m, 8H).; UHPLC-MS (ES+APCI) m/z calcd. for $C_{24}H_{32}N_3O_3$ $[M]^+$ = 410.52. Found: 410.40. (94.2%)

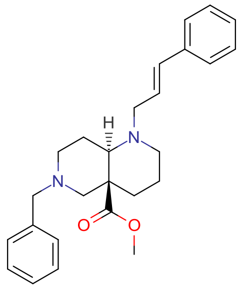

Methyl 6-benzyl-1-[(2E)-3-phenylprop-2-en-1-yl]-decahydro-1,6-naphthyridine-4a-carboxylate (46f): 14.3 mg (34% yield).; ^{1}H NMR (300 MHz, $CDCl_3$): δ 8.48 (s, 1H), 7.42 – 7.28 (m, 7H), 7.22 – 7.20 (m, 2H), 6.71 (d, J = 15.8 Hz, 1H), 6.25 (dt, J = 7.3, 15.8 Hz, 1H), 3.90 (d, J = 7.2 Hz, 2H), 3.74 (s, 3H), 3.51 (d, J = 16.3 Hz, 2H), 3.32 (d, J = 13.3 Hz, 1H), 3.15 (dd, J = 1.9, 11.3 Hz, 1H), 3.07 (d, J = 9.4 Hz, 1H), 2.77 – 2.48 (m, 3H), 2.17 (dt, J = 3.1, 11.3 Hz, 1H), 2.06 – 1.97 (m, 2H), 1.80 (d, J = 11.3 Hz, 1H), 1.71 – 1.69 (m, 2H), 1.30 – 1.20 (m, 1H).; UHPLC-MS (ES+APCI) m/z calcd. for $C_{26}H_{33}N_2O_2$ $[M]^+$ = 405.54. Found: 405.20.

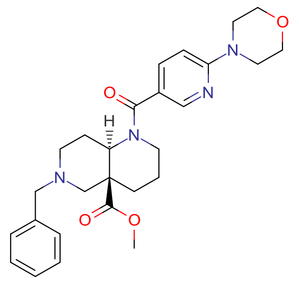

Methyl-6-benzyl-1-[6-(morpholin-4-yl)pyridine-3-carbonyl]-decahydro-1,6-naphthyridine-4a-carboxylate (46g): 15.2 mg (31% yield).; ^{1}H NMR (300 MHz, $CDCl_3$): δ 8.22 (d, J = 1.9 Hz, 1H), 8.11 (s, 1H), 7.60 (dd, J = 2.4, 6.4 Hz, 1H), 7.25 – 7.20 (m, 4H), 6.54 (d, J = 8.8 Hz, 1H), 3.92 (dt, J = 3.3, 7.1 Hz, 1H), 3.74 (dd, J = 1.3, 5.1 Hz, 4H), 3.66 (s, 3H), 3.74 (dd, J = 1.3, 5.1 Hz, 4H), 3.42 (s, 3H), 3.19 (dt, J = 3.3, 12.0 Hz, 2H), 3.05 – 2.89 (m, 2H), 2.69 (dq, J = 4.3, 13.4 Hz, 1H), 2.37 (dd, J = 2.4, 11.7 Hz, 1H), 2.23 (dt, J = 2.4, 11.7 Hz, 1H), 1.95 (d, J = 4.2 Hz, 1H), 1.62 – 1.42 (m, 2H), 1.33 (dt, J = 5.0, 12.7 Hz, 1H).; UHPLC-MS (ES+APCI) m/z calcd. for $C_{27}H_{35}N_4O_4$ [M]+ = 479.58. Found: 479.20.

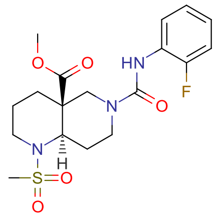

Methyl 6-[(2-fluorophenyl)carbamoyl]-1-methanesulfonyl-decahydro-1,6-naphthyridine-4a-carboxylate (52a^i): 26.2 mg (58% yield).; ^{1}H NMR (300 MHz, $CDCl_3$): δ 7.95 (dt, J = 1.8, 8.7 Hz, 1H), 7.10 – 7.02 (m, 2H), 6.98 – 6.91 (m, 1H), 4.38 – 4.30 (m, 2H), 4.16 (dt, J = 2.4, 13.5 Hz, 1H), 3.77 (s, 3H), 3.04 (dd, J = 4.7, 11.5 Hz, 1H), 2.97 (s, 3H), 2.91 – 2.71 (m, 3H), 2.60 – 2.42 (m, 2H), 2.17 (d, J = 14.2 Hz, 1H), 2.00 (s, 2H), 1.74 – 1.69 (m, 1H), 1.39 – 1.28 (m, 1H).; UHPLC-MS (ES+APCI) m/z calcd. for $C_{18}H_{25}FN_3O_5S$ [M]+ = 414.46. Found: 414.20.

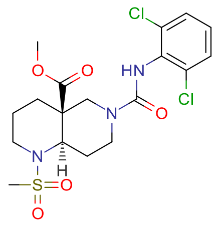

Methyl-6-[(2,6-dichlorophenyl)carbamoyl]-1-methanesulfonyl-decahydro-1,6-naphthyridine-4a-carboxylate (52b[i]): 18.9 mg (37% yield).; ^{1}H NMR (300 MHz, $CDCl_3$): δ 7.33 (d, J = 8.0 Hz, 2H), 7.08 (dd, J = 7.7, 8.5 Hz, 1H), 6.69 (bs, 1H), 4.48 (dd, J = 2.6, 13.9 Hz, 1H), 4.39 – 4.33 (m, 1H), 4.17 (dt, J = 2.7, 6.5 Hz, 1H), 3.80 (s, 3H), 3.06 (dd, J = 4.0, 12.1 Hz, 1H), 2.97 (s, 3H), 2.88 (dd, J = 3.1, 13.5 Hz, 1H), 2.88 – 2.73 (m, 2H), 2.57 (dq, J = 4.5, 12.6 Hz, 1H), 2.46 – 2.39 (m, 1H), 2.15 (d, J = 13.5 Hz, 1H), 1.74 – 1.68 (m, 2H), 1.39 – 1.29 (m, 1H).; UHPLC-MS (ES+APCI) m/z calcd. for $C_{18}H_{24}Cl_2N_3O_5S$ [M]+ = 465.36. Found: 465.20.

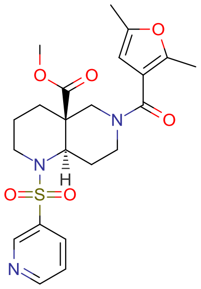

Methyl-6-(2,5-dimethylfuran-3-carbonyl)-1-(pyridine-3-sulfonyl)-decahydro-1,6-naphthyridine-4a-carboxylate (52c[i]): 15.4 mg (38% yield).; ^{1}H NMR (300 MHz, $CDCl_3$): δ 9.02 (s, 1H), 8.80 (d, J = 3.9 Hz, 1H), 8.14 (d, J = 10.0 Hz, 1H), 7.50 (dd, J = 4.9, 8.0 Hz, 1H), 5.85 (s, 1H), 4.23 (d, J = 12.3 Hz, 2H), 3.65 (s, 3H), 3.03 – 2.97 (m, 1H), 2.86 – 2.77 (m, 3H), 2.60 – 2.42 (m, 1H), 2.34 – 2.29 (m, 2H), 2.26 (s, 3H), 2.22 (s, 3H), 2.09 – 2.06 (m, 1H), 1.78 – 1.72 (m, 2H), 1.26 – 1.16 (m, 1H).; UHPLC-MS (ES+APCI) m/z calcd. for $C_{22}H_{28}N_3O_6S$ [M]+ = 462.53. Found: 462.20.

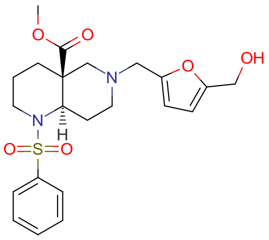

Methyl 1-(benzenesulfonyl)-6-{[5-(hydroxymethyl)furan-2-yl]methyl}-decahydro-1,6-naphthyridine-4a-carboxylate (52d[ii]): 13.8 mg (35% yield).; ^{1}H NMR (300

MHz, $CDCl_3$): δ 7.83 – 7.79 (m, 2H), 7.56 – 7.40 (m, 3H), 6.20 (d, J = 3.1 Hz, 1H), 6.11 (d, J = 3.1 Hz, 1H), 4.54 (d, J = 2.3 Hz, 2H), 4.19 (d, J = 14.7 Hz, 1H), 3.74 (s, 3H), 3.62 (d, J = 14.5 Hz, 1H), 3.43 (d, J = 14.5 Hz, 1H), 3.18 – 3.14 (m, 3H), 2.98 (d, J = 11.0 Hz, 1H), 2.70 – 2.61 (m, 3H), 2.17 – 2.12 (m, 1H), 2.09 – 2.03 (m, 1H), 1.86 (d, J = 11.6 Hz, 1H), 1.69 – 1.59 (m, 2H), 1.23 – 1.12 (m, 1H).; UHPLC-MS (ES+APCI) m/z calcd. for $C_{22}H_{29}N_2O_6S$ [M]+ = 449.53; Found: 449.20.

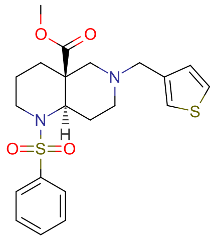

Methyl 1-(benzenesulfonyl)-6-[(thiophen-3-yl)methyl]-decahydro-1,6-naphthyridine-4a-carboxylate (52e[ii]): 12.6 mg (33% yield).; ^{1}H NMR (300 MHz, $CDCl_3$): δ 7.82 (dd, J = 1.3, 7.9 Hz, 2H), 7.58 – 7.47 (m, 3H), 7.24 (d, J = 3.0 Hz, 1H), 7.08 (d, J = 2.0 Hz, 1H), 6.95 (dd, J = 1.3, 4.9 Hz, 1H), 4.19 (dt, J = 2.3, 11.7 Hz, 1H), 3.73 (s, 3H), 3.60 (d, J = 13.6 Hz, 1H), 3.42 (d, J = 13.6 Hz, 1H), 3.17 (dd, J = 2.3, 11.5 Hz, 1H), 3.00 (d, J = 11.5 Hz, 1H), 2.70 – 2.62 (m, 3H), 2.16 – 2.03 (m, 3H), 1.83 (d, J = 11.7 Hz, 1H), 1.68 – 1.61 (m, 2H), 1.22 – 1.11 (m, 1H).; UHPLC-MS (ES+APCI) m/z calcd. for $C_{21}H_{27}N_2O_4S_2$ [M]+ = 435.57. Found: 435.20.

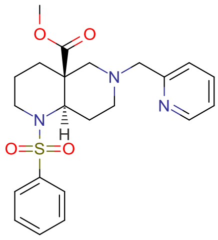

Methyl 1-(benzenesulfonyl)-6-[(pyridin-2-yl)methyl]-decahydro-1,6-naphthyridine-4a-carboxylate (52f[ii]): 14.4 mg (38% yield).; ^{1}H NMR (300 MHz, $CDCl_3$): δ 8.50 (d, J = 6.5 Hz, 1H), 7.82 – 7.79 (m, 2H), 7.69 (dt, J = 1.7, 7.7 Hz, 1H), 7.56 – 7.47 (m, 3H), 7.38 (d, J = 7.6 Hz, 1H), 7.20 (dd, J = 5.1, 7.4 Hz, 1H), 4.21 (d, J = 11.8 Hz, 1H), 3.74 (s, 3H),

3.69 (d, J = 14.6 Hz, 1H), 3.54 (d, J = 14.6 Hz, 1H), 3.10 (d, J = 11.5 Hz, 1H), 2.94 (d, J = 13.3 Hz, 1H), 2.78 – 2.62 (m, 3H), 2.21 – 1.97 (m, 4H), 1.68 – 1.62 (m, 2H), 1.25 – 1.14 (m, 1H).; UHPLC-MS (ES+APCI) m/z calcd. for $C_{22}H_{28}N_3O_4S$ [M]+ = 430.53. Found: 430.20.

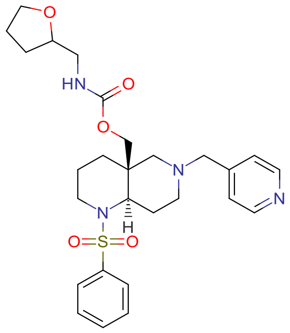

1-(Benzenesulfonyl)-6-[(pyridin-4-yl)methyl]-decahydro-1,6-naphthyridin-4a-yl]methyl N-[(oxolan-2-yl)methyl]carbamate (52g[ii]): 10.8 mg (30% yield).; ^{1}H NMR (300 MHz, $CDCl_3$): δ 8.52 (t, J = 5.3 Hz, 2H), 7.76 – 7.73 (m, 2H), 7.57 – 7.49 (m, 3H), 7.38 – 7.34 (m, 2H), 4,90 (bs, 1H), 4.81 (d, J = 10.5 Hz, 1H), 4.38 (d, J = 10.5 Hz, 1H), 4.26 (d, J = 13.3 Hz, 1H), 4.01 – 3.94 (m, 1H), 3.88 – 3.71 (m, 3H), 3.56 (d, J = 14.8 Hz, 1H), 3.33 (dd, J = 5.5, 14.8 Hz, 1H), 3.13 – 2.99 (m, 1H), 2.94 – 2.91 (m, 1H), 2.67 – 2.60 (m, 3H), 2.24 – 2.15 (m, 1H), 2.04 – 1.85 (m, 6H), 1.71 (d, J = 12.7 Hz, 1H), 1.60 – 1.47 (m, 3H), 0.86 (t, J = 11.1 Hz, 1H).; UHPLC-MS (ES+APCI) m/z calcd. for $C_{27}H_{37}N_4O_5S$ [M]+ = 529.66. Found: 529.20.

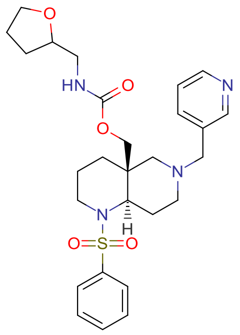

1-(Benzenesulfonyl)-6-[(pyridin-3-yl)methyl]-decahydro-1,6-naphthyridin-4a-yl]methyl N-[(oxolan-2-yl)methyl]carbamate (52h[ii]): 18.9 mg (37% yield).; ^{1}H NMR (300 MHz, $CDCl_3$): δ 8.50 – 8.46 (m, 2H), 7.85 (d, J = 7.2 Hz, 1H), 7.76 – 7.73 (m, 2H), 7.57 – 7.48 (m, 3H), 7.37 – 7.30 (m, 1H), 5.02 (bs, 1H), 4.70 (d, J = 10.9 Hz, 1H), 4.41 (d, J = 10.9 Hz, 1H), 4.26 (d, J = 11.2 Hz, 1H), 3.98 – 3.93 (m, 1H), 3.86 – 3.81 (m, 1H), 3.78 – 3.71 (m, 1H), 3.58 (d, J = 13.0 Hz, 1H), 3.46 – 3.33 (m, 2H), 3.16 – 2.93 (m, 2H), 2.65 (t, J = 11.7 Hz, 3H), 2.25

– 2.15 (m, 1H), 2.00 – 1.87 (m, 6H), 1.70 – 1.50 (m, 4H), 0.86 (t, J = 11.1 Hz, 1H).; UHPLC-MS (ES+APCI) m/z calcd. for $C_{27}H_{37}N_4O_5S$ [M]+ = 529.66. Found: 529.20.

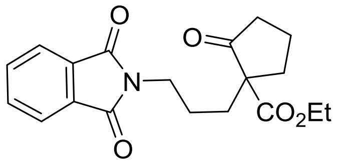

Chemical Formula: $C_{19}H_{21}NO_5$
Exact Mass: 343.14
Molecular Weight: 343.38

Ethyl 1-(3-(1,3-dioxoisoindolin-2-yl)propyl)-2-oxocyclopentane-1-carboxylate (24a): NaH (60% in mineral oil) (2.92 g; 72.1 mmol) was suspended in a mixture of dry THF-DMF (1:1; 200 mL), Keto-ester **23a** (10.0 g; 60.83 mmol) dissolved in dry THF was added dropwise and 0 °C, the resulting solution allowed to stir for 10 min to complete deprotonation. Then bromopropyl-pthalimide **16** (18.3 g; 66.91 mmol) in dry THF was added slowly at 0 °C. Then the reaction mixture was fitted with a refluxing condenser and heated at 65 °C for overnight. The reaction completion was monitored by TLC and cooled to rt. and then the pH of the reaction mixture was adjusted to neutral with 1.0 M aq. HCl. The layers were separated and aq. layer was extracted with EtOAc, combined organic layer was washed with water, brine solution dried over anhydrous Na_2SO_4. After concentration the crude residue was then purified by column chromatography using 10-15% EtOAc in pet-ether as a gradient to give pure pthalimide derivative **(24a)** 16.7g (80% yield).; ^{1}H NMR (400 MHz, $CDCl_3$) δ 7.70 – 7.64 (m, 1H), 7.61 – 7.56 (m, 1H), 4.05 – 3.92 (m, 1H), 3.52 (q, *J* = 7.0 Hz, 1H), 2.41 – 2.32 (m, 1H), 2.31 – 2.19 (m, 1H), 2.10 (dt, *J* = 12.8, 8.3 Hz, 1H), 1.92 – 1.70 (m, 2H), 1.69 – 1.58 (m, 1H), 1.55 – 1.42 (m, 1H), 1.12 – 1.05 (m, 1H).: ^{13}C NMR (101 MHz, $cdcl_3$) δ 214.4, 170.8, 168.3, 134.1, 132.2, 123.3, 61.5, 60.1, 38.0, 37.9, 33.1, 30.9, 27.0, 24.2, 19.7, 14.2.; HPLC-MS (ESI) m/z calcd. for $C_{19}H_{21}NO_5$ $[M+H]^+$ = 344.14. Found: 344.08.
TLC stain: UV, KMnO4
R_f: 0.5 (30% EtOAc in pet-ether)

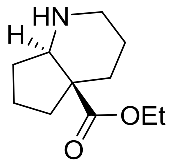

Chemical Formula: $C_{11}H_{19}NO_2$
Exact Mass: 197.14
Molecular Weight: 197.28

Ethyl octahydro-4aH-cyclopenta[b]pyridine-4a-carboxylate (25a): Pthalimide **24a** (16.0 g; 44.27 mmol) was dissolved in analytical grade ethanol, was added methyl amine (33% in ethanol) (12.5 g; 132.80 mmol) dropwise at rt. The reaction mixture was fitted with a refluxing condenser and refluxed at 80 °C for 3h. The reaction completion was monitored by TLC and cooled to rt. (the by-product di-amide precipitated out). The solid di-amide separated by filtration and filtrate was concentrated and filtered through silica column to give pure product 6.56 g (69% yield).

TLC stain: I_2, $KMnO_4$;

Rf: 0.6 (10% MeOH (Buffered with aq. NH_3) in DCM.

The amino-ketone (5.0 g; 23.44 mmol) was dissolved in dry DCM (230 mL; dilution is very important to get diastereo selectivity!!!) was added equivalent amount of flame dried powdered MS (5.0 g) followed by $NaBH(OEh)_3$ (16.33 g; 35.17 mmol) in one portion, then it was allowed to stir for 24 h. at rt. After completion of SM was confirmed by TLC, diluted with DCM and saturated aq. $NaHCO_3$ was added, stirred for addition 15 min at rt. The DCM layer was washed couple of times with saturated aq. $NaHCO_3$ then water and brine solution. After concentration the crude residue was filtered through silica column to get the mixture of diastereomers **(25a)** (6:1) 3.3 g (69% yield).; ^{1}H NMR (400 MHz, CD_3OD) δ 4.22 – 4.12 (m, 1H), 3.16 – 3.06 (m, 1H), 2.66 (td, *J* = 13.2, 3.5 Hz, 1H), 2.52 (dt, *J* = 19.9, 9.9 Hz, 1H), 2.38 – 2.28 (m, 1H), 2.11 (ddd, *J* = 12.7, 8.3, 1.4 Hz, 1H), 1.92 – 1.81 (m, 1H), 1.76 – 1.63 (m, 1H), 1.56 (dddd, *J* = 14.5, 12.9, 9.7, 6.1 Hz, 1H), 1.40 (dddd, *J* = 21.7, 17.3, 8.8, 4.0 Hz, 1H), 1.29 – 1.21 (m, 2H).; ^{13}C NMR (101 MHz, CD_3OD) δ 175.8, 65.6, 60.3, 50.7, 46.4, 34.2, 34.0, 26.4, 23.1, 18.6, 13.4.; HPLC-MS (ESI) m/z calcd. for $C_{11}H_{20}NO_5$ $[M+H]^+$ = 198.14; Found: 198.09.

TLC stain: I_2, $KMnO_4$

R_f: 0.3 (10% MeOH (Buffered with aq. NH_3) in DCM)

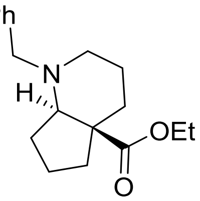

Chemical Formula: $C_{18}H_{25}NO_2$
Exact Mass: 287.19
Molecular Weight: 287.40

Ethyl 1-benzyloctahydro-4aH-cyclopenta[b]pyridine-4a-carboxylate (26): Mixture of diastereomers **25a** (3.0 g; 14.44 mmol) were dissolved in dry DCM, was added benzaldehyde (1.84 g; 17.33 mmol) followed by $NaBH(OAc)_3$ (3.67 g; 17.33 mmol) stirred for 1h. at rt. The reaction mixture was diluted with DCM washed with saturated aq. $NaHCO_3$ and stirred for 10 min. The DCM layer washed couple of times with saturated aq. $NaHCO_3$ then water and brine solution. After concentration the crude residue was carefully purified by column chromatography using 2-10% of EtOAc in pet-ether as an eluent to give the pure diastereomer 3.7g (89% yield).; ^{1}H NMR (400 MHz, CD_3OD) δ 7.24 (ddd, *J* = 27.3, 17.7, 7.1 Hz, 1H), 4.21 – 4.08 (m, 1H), 3.91 (d, *J* = 13.3 Hz, 1H), 3.09 (d, *J* = 13.3 Hz, 1H), 2.83 – 2.68 (m, 1H), 2.39 (d, *J* = 12.6 Hz, 1H), 2.23 – 2.09 (m, 1H), 1.95 – 1.84 (m, 1H), 1.83 – 1.58 (m, 1H), 1.50 (ddd, *J* = 15.4, 9.4, 6.5 Hz, 1H), 1.27 (t, *J* = 7.1 Hz, 1H), 1.09 (td, *J* = 12.9, 4.3 Hz, 1H).; ^{13}C NMR (101 MHz, CD_3OD) δ 175.6, 139.4, 128.9, 127.9, 126.6, 73.7, 60.2, 59.8, 54.6, 53.5, 48.5, 48.3, 48.1, 47.8, 47.6, 47.4, 47.2, 35.3, 34.5, 27.3, 23.7, 19.8, 13.5.; HPLC-MS (ESI) m/z calcd. for $C_{18}H_{25}NO_2$ $[M+H]^+$ = 288.19. Found: 288.30.
TLC stain: UV, I_2, $KMnO_4$
R_f: 0.6 (5% MeOH (Buffered with aq. NH_3) in DCM)

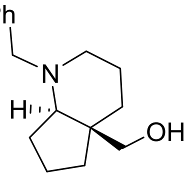

Chemical Formula: $C_{16}H_{23}NO$
Exact Mass: 245.18
Molecular Weight: 245.37

1-Benzyloctahydro-4aH-cyclopenta[b]pyridin-4a-yl)methanol (26a): To a suspension of LAH (3.1 g; 10.79 mmol) in dry THF (55 mL) was added amino-ester **26** (0.45 g; 11.86 mmol) dissolved in dry THF at 0 °C. Then the reaction mixture was stirred for overnight at

rt., after completion of reaction, excess LAH was quenched with moistured Na_2SO_4. The greyish white solid was filtered washed several times with EtOAc and the filtrated was concentrated to give pure product 2.48g, (94% yield).; HPLC-MS (ESI) m/z calcd. for $C_{16}H_{23}NO$ $[M+H]^+$ = 246.08. Found: 246.22.

TLC stain: UV, I_2, $KMnO_4$

R_f: 0.3 (10% MeOH (Buffered with aq. NH_3) in DCM)

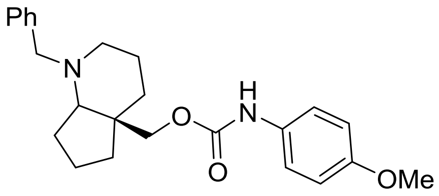

Chemical Formula: $C_{24}H_{30}N_2O_3$
Exact Mass: 394.23
Molecular Weight: 394.52

1-Benzyloctahydro-4aH-cyclopenta[b]pyridin-4a-yl)methyl (4-methoxyphenyl)carbamate (27): The pure alcohol **26a** (1.46 g; 5.65 mmol) was dissolved in dry DCM (28 mL), was added Et_3N (0.858 g; 8.48 mmol) followed by 4-methoxy phenyl isocyanate (0.946 g; 6.22 mmol) and stirred for 4 h. at rt. The reaction mixture was diluted with DCM, washed couple of times with saturated aq. $NaHCO_3$ then water and brine solution. After concentration the crude residue was purified by column chromatography using 2-10% of EtOAc in pet-ether as a gradient to give the pure carbamate 1.38g (62% yield).; ^{1}H NMR (400 MHz, CD_3OD) δ 7.38 – 7.15 (m, 1H), 6.85 – 6.79 (m, 1H), 4.59 (dd, *J* = 11.1, 1.8 Hz, 1H), 4.02 (d, *J* = 11.2 Hz, 1H), 3.83 (d, *J* = 13.2 Hz, 1H), 2.93 (d, *J* = 13.2 Hz, 1H), 2.80 (d, *J* = 8.1 Hz, 1H), 2.11 (d, *J* = 12.4 Hz, 1H), 2.02 (dd, *J* = 12.3, 7.6 Hz, 1H), 1.97 – 1.86 (m, 1H), 1.86 – 1.63 (m, 1H), 1.54 – 1.35 (m, 1H), 1.17 (tt, *J* = 10.4, 8.0 Hz, 1H), 1.05 – 0.94 (m, 1H).; ^{13}C NMR (101 MHz, CD_3OD) δ 155.9, 139.6, 128.7, 127.9, 126.6, 120.6, 113.9, 72.9, 63.7, 60.4, 54.7, 54.6, 45.0, 32.9, 32.2, 26.8, 21.8, 18.3.; HPLC-MS (ESI) m/z calcd. for $C_{24}H_{31}N_2O_3$ $[M+H]^+$ = 395.23; Found: 395.33.

TLC stain: UV, I_2, $KMnO_4$

R_f: 0.5 (10% MeOH (Buffered with aq. NH_3) in DCM)

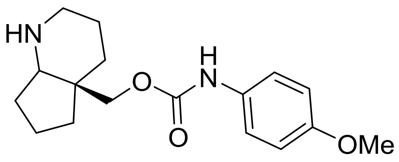

Chemical Formula: $C_{17}H_{24}N_2O_3$
Exact Mass: 304.18
Molecular Weight: 304.39

Octahydro-4aH-cyclopenta[b]pyridin-4a-yl)methyl(4-methoxyphenyl)carbamate (27a): The carbamate **27** (0.196 g; 0.472 mmol) was dissolved in MeOH (10 mL) and carefully 10% Pd/C was added under argon atmosphere. Then the reaction mixture was evacuated couple of times by applying vacuum and re-filling the hydrogen, stirred under H2 atmosphere for overnight. After complete consumption SM, it was filtered through HPLC filter using syringe, washed couple of times with MeOH and concentrated to give pure product **27a** 123 mg (86% yield).; HPLC-MS (ESI) m/z calcd. for $C_{17}H_{24}N_2O_3$ $[M+H]^+$ = 305.18. Found: 305.22.

TLC stain: UV, I_2, $KMnO_4$

R_f: 0.2 (10% MeOH (Buffered with aq. NH_3) in DCM)

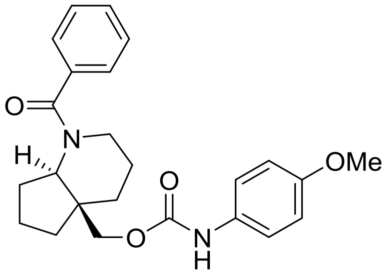

Chemical Formula: $C_{24}H_{28}N_2O_4$
Exact Mass: 408.2049
Molecular Weight: 408.4980

1-Benzoyloctahydro-4aH-cyclopenta[b]pyridin-4a-yl)methyl 2-(4-methoxyphenyl)acetate (28a): The benzoic acid (0.05 g; 0.37 mmol) dissolved in dry DMF (5 mL), added DIPEA (0.08 g; 0.63 mmol), followed by HATU (0.136 g; 0.35 mmol) and amine **27a** (0.01 g; 0.32 mmol) at rt, and the resultant solution was stirred at 50 °C for overnight. The reaction mixture was cooled to rt, water was added and extracted with EtOAc, washed with couple of times with water followed by brine solution. After concentration the crude residue was purified by column chromatography using 2-5% of MeOH (Buffered with aq. NH_3) in DCM as an eluent to give the pure amide 65mg (50% yield).; ^1H NMR (400

MHz, CD_3OD) δ 7.33 – 7.14 (m, 1H), 3.86 – 3.76 (m, 1H), 3.36 – 3.31 (m, 1H), 3.15 (dd, J = 11.6, 8.2 Hz, 1H), 2.94 (dd, J = 13.2, 4.3 Hz, 1H), 2.84 – 2.75 (m, 1H), 2.09 (dt, J = 8.5, 5.0 Hz, 1H), 1.99 – 1.82 (m, 1H), 1.78 – 1.59 (m, 1H), 1.49 – 1.33 (m, 1H), 1.07 (dtd, J = 17.7, 10.6, 5.2 Hz, 1H), 0.96 – 0.82 (m, 1H).; ^{13}C NMR (101 MHz, CD_2Cl_2) δ 172.5, 138.3, 130.1, 129.7, 128.6, 128.5, 127.4, 114.3, 67.3, 63.8, 55.6, 50.7, 45.4, 33.2, 31.8, 26.9, 22.0, 19.2.; HPLC-MS (ESI) m/z calcd. for $C_{24}H_{29}N_2O_4$ $[M+H]^+$ = 409.20; Found: 409.12.

TLC stain: UV, I_2, $KMnO_4$

R$_f$: 0.6 (10% MeOH (Buffered with aq. NH_3) in DCM

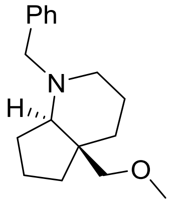

Chemical Formula: $C_{17}H_{25}NO$
Exact Mass: 259.19
Molecular Weight: 259.39

1-Benzyl-4a-(methoxymethyl)octahydro-1H-cyclopenta[b]pyridine (29): NaH (0.05 g; 1.27 mmol) was suspended in a mixture of dry THF-DMF (1:1), pure alcohol **26a** (0.26 g; 1.06 mmol) in dry THF (7 mL) was added dropwise and 0 °C, the resulting solution allowed to stir for 10 min to complete deprotonation. Then iodomethane (0.22 g; 1.56 mmol) in dry THF was added slowly at 0 °C. The reaction mixture was stirred for overnight at room temperature. The reaction completion was monitored by TLC, upon completion; pH of the reaction mixture was adjusted to neutral with 1.0 M aq. HCl. Extracted the reaction mixture with EtOAc and the combined organic layer were washed with water, brine solution dried over anhydrous Na_2SO_4, concentrated to give the cure product. It was then purified by column chromatography using 10-15% EtOAc in pet-ether to give pure ether **(29)** 200 mg 73% yield).; ^{1}H NMR (400 MHz, CD_3OD) δ 7.37 – 7.09 (m, 30H), 3.89 – 3.77 (m, 11H), 3.34 (s, 16H), 3.15 (dd, J = 11.6, 8.2 Hz, 6H), 2.94 (dd, J = 13.2, 4.3 Hz, 6H), 2.85 – 2.76 (m, 6H), 2.09 (dt, J = 8.5, 5.0 Hz, 6H), 2.00 – 1.84 (m, 18H), 1.80 – 1.59 (m, 26H), 1.51 – 1.34 (m, 17H), 1.16 – 1.02 (m, 8H), 0.98 – 0.84 (m, 12H).; ^{13}C NMR (101 MHz, CD_3OD) δ 139.6, 128.8, 127.9, 126.6, 73.0, 71.7, 60.5, 58.6, 54.7, 45.4, 33.0, 32.3, 26.8, 21.9, 18.5.; HPLC-MS (ESI) m/z calcd. for $C_{17}H_{26}NO$ $[M+H]^+$ = 260.19; Found: 260.31.

TLC stain: UV, I_2, $KMnO_4$

R_f: 0.6 (5% MeOH (Buffered with aq. NH_3) in DCM)

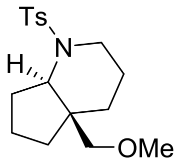

Chemical Formula: $C_{17}H_{25}NO_3S$
Exact Mass: 323.1555
Molecular Weight: 323.4510

4a-(Methoxymethyl)octahydro-1H-cyclopenta[b]pyridine (30a): The ether **29a** (0.186 g; 0.72 mmol) was dissolved in MeOH (8 mL) and carefully 10% Pd/C was added under argon atmosphere. Then the reaction mixture was evacuated couple of times by applying vacuum and re-filling the hydrogen, stirred under H_2 atmosphere for overnight. After complete consumption SM, it was filtered through HPLC filter using syringe, washed couple of times with MeOH and concentrated to give pure product 118 mg (97% yield). Amine (0.03 g; 0.17 mmol) was dissolved in dry DCM (2 mL) was added Et_3N (29 µL; 0.21 mmol), followed by TsCl (0.036 g; 0.19 mmol) in one portion at 0 °C, after 4 h. at rt., it was then diluted with DCM, washed with couple of times with saturated aq. $NaHCO_3$ then water, brine solution and dried over Na_2SO_4. Then concentrated to give crude residue was purified by column chromatography using 2-5% of MeOH (Buffered with aq. NH_3) in DCM as an eluent to give the pure sulphonamide **30a** 38 mg (66 % yield).; HPLC-MS (ESI) m/z calcd. for $C_{17}H_{26}NO_3S$ $[M+H]^+$ = 324.15; Found: 324.22.

TLC stain: UV, I_2, $KMnO_4$

R_f: 0.4 (10% MeOH (Buffered with aq. NH_3) in DCM)

<u>Experimental for the production phase of 8-deoxy serratinine based scaffolds:</u>

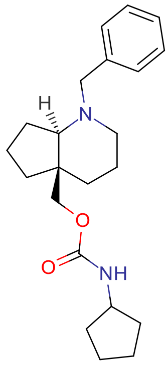

1-Benzyl-octahydro-1H-cyclopenta[b]pyridin-4a-yl]methyl N-cyclopentyl-carbamate (27a^{i}): To a solution of the alcohol **26a** (6.0 g, 25 mmol) in dry DCM (250 mL) was added triethylamine (3.7 g, 29 mmol) and cyclopentylisocyanate (3.3 g, 29 mmol) and the reaction mixture was stirred at room temperature for 15 hours. After the complete consumption of the starting material monitored by TLC, the reaction mixture was washed with saturated aq. $NaHCO_3$ and brine. After concentration, the crude residue was purified by column chromatography using 10-15% of EtOAc in cyclohexane as an eluent to give the carbamate 5.5 g (63% yield).; ^{1}H NMR (300 MHz, $CDCl_3$): δ 7.25 – 7.09 (m, 5H), 5.00 – 4.98 (m, 1H), 4.63 – 4.61 (m, 1H), 4.43 (d, J = 10.9 Hz, 1H), 3.89 (d, J = 10.0 Hz, 1H), 3.76 (d, J = 13.3 Hz, 1H), 2.83 (d, J = 13.3 Hz, 1H), 2.74 (d, J = 7.3 Hz, 1H), 1.96 – 1.75 (m, 7H), 1.63 – 1.42 (m, 9H), 1.12 – 1.02 (m, 1H), 0.92 – 0.70 (m, 2H).; UHPLC-MS (ES+APCI) m/z calcd. for $C_{22}H_{33}N_2O_2$ $[M]^+$ = 357.51. Found: 357.05.

TLC stain: I_2, $KMnO_4$

R_f: 0.5 (Cyclohexane/EtOAc 5:1)

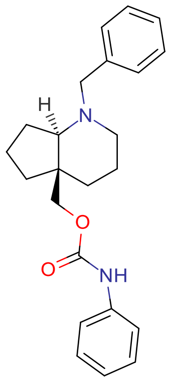

1-Benzyl-octahydro-1H-cyclopenta[b]pyridin-4a-yl]methyl N-phenylcar-bamate (27c^{i}): To a solution of the alcohol **26b** (6.0 g, 25 mmol) in dry DCM (250 mL) was added triethylamine (5.1 g, 37 mmol) and phenylisocyanate (3.2 g, 29 mmol) and the reaction mixture was stirred at room temperature for 15 hours. After the complete consumption of the starting material monitored by TLC, the reaction mixture was washed with saturated aq. $NaHCO_3$ and brine. After concentration, the crude residue was purified by column chromatography using 10-15% of EtOAc in cyclohexane as an eluent to give the carbamate 6.0 g (67% yield).; ^{1}H NMR (300 MHz, $CDCl_3$): δ 7.35 (d, J = 7.9 Hz, 2H), 7.34 – 7.16 (m, 7H), 7.02 (t, J = 7.3 Hz, 1H), 4.60 (d, J = 11.2 Hz, 1H), 3.82 (d, J = 13.3 Hz, 1H), 2.90 (d, J = 13.3 Hz, 1H), 2.80 (d, J = 7.0 Hz, 1H), 2.01 (dd, J = 7.6, 12.1 Hz, 2H), 1.92 – 1.80 (m, 2H),

1.74 – 1.65 (m, 2H), 1.47 (dd, J = 4.8, 11.7 Hz, 1H), 1.42 – 1.36 (m, 1H), 1.23 – 1.21 (m, 1H), 1.17 – 1.07 (m, 1H), 0.97 (dt, J = 4.4, 12.8 Hz,1H), 0.86 – 0.78 (m, 1H).; UHPLC-MS (ES+APCI) m/z calcd. for $C_{23}H_{29}N_2O_2$ $[M]^+$ = 365.48. Found: 365.00.

TLC stain: I_2, $KMnO_4$

R_f: 0.4 (Cyclohexane/EtOAc 5:1)

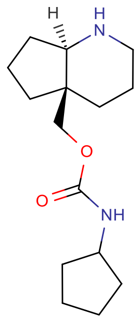

Octahydro-1H-cyclopenta[b]pyridin-4a-yl]methyl N-cyclopentylcarbamate (27a): To a solution of the carbamate (**27a^i^**) (5.5 g, 15 mmol) in dry methanol (150 mL) was added palladium on charcoal (10 wt%) and the reaction mixture was stirred under hydrogen atmosphere overnight. After complete consumption of the starting material monitored by TLC, the reaction mixture was filtered through a pad of celite. Concentration of the filtrate afforded the pure product 3.5 g (85% yield).; ^{1}H NMR (300 MHz, $CDCl_3$): δ 4.28 (d, J = 10.5 Hz, 1H), 4.15– 3.93 (m, 1H), 3.89 (d, J = 11.0 Hz, 1H), 3.16 (dd, J = 4.5, 12.1 Hz, 1H), 2.66 (dt, J = 2.6, 12.5 Hz, 1H), 2.49 (dd, J = 7.4, 12.3 Hz, 1H), 2.07 (d, J = 12.5 Hz, 1H), 2.02 – 1.90 (m, 2H), 1.84 – 1.54 (m, 10H), 1.45 – 1. 32 (m, 3 H), 1.31 – 1.23 (m, 1H), 1.08 (dt, J = 4.2, 13.1 Hz, 2H), 0.90 (t, J = 7.1 Hz, 1H).; UHPLC-MS (ES+APCI) m/z calcd. for $C_{15}H_{27}N_2O_2$ $[M]^+$ = 267.34. Found: 267.05.

TLC stain: UV, I_2, KMnO4

R_f: 0.4 (15% MeOH (Buffered with aq. NH_3) in DCM)

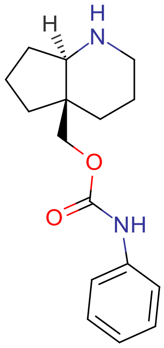

Octahydro-1H-cyclopenta[b]pyridin-4a-yl]methyl N-phenylcarbamate (27c): To a solution of the carbamate (**27c**[i]) (6.0 g, 16 mmol) in dry methanol (160 mL) was added palladium on charcoal (10 wt%) and the reaction mixture was stirred under hydrogen atmosphere overnight. After complete consumption of the starting material monitored by TLC, the reaction mixture was filtered through a pad of celite. Concentration of the filtrate afforded the pure product 4.4 g (97% yield).; ^{1}H NMR (300 MHz, $CDCl_3$): δ 7.45 (d, J = 8.1 Hz, 2H), 7.26 (t, J = 7.5 Hz, 2H), 7.02 (d, J = 7.4 Hz, 1H), 4.33 (d, J = 11.8 Hz, 1H), 4.06 (d, J = 11.7 Hz, 1H), 3.66 (dd, J = 4.1, 12.9 Hz, 1H), 2.97 – 2.81 (m, 2H), 2.21 – 2.13 (m, 3H), 1.86 – 1.71 (m, 4H), 1.70 – 1.57 (m, 1H), 1.49 (dt, J = 3.4, 14.8 Hz, 1H), 1.29 – 1.18 (m, 1H).; UHPLC-MS (ES+APCI) m/z calcd. for $C_{16}H_{23}N_2O_2$ $[M]^+$ = 275.36. Found: 275.00.
TLC stain: UV, I_2, $KMnO_4$
R_f: 0.3 (10% MeOH (Buffered with aq. NH_3) in DCM)

Experimental for the representative examples of final diversification:

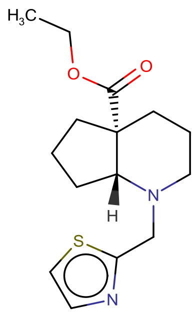

Ethyl 1-[(1,3-thiazol-2-yl)methyl]-octahydro-1H-cyclopenta[b]pyridine-4a-carboxylate (53a[iii]**)** 17.5 mg (39% yield).; 1H NMR (300 MHz, $CDCl_3$): δ 7.67 (d, J = 3.3Hz, 1H), 7.24 (d, J = 3.3Hz, 1H), 4.21 (m, 1H), 4.12 (d, 1H, J = 15.5Hz), 3.74 (d, 1H, J = 15.3Hz), 2.45 (m, 1H), 2.97 (dd, J = 3.6Hz, J = 10.6Hz, 1H), 2.19 (s, 1H), 1.81 (m, 1H), 1.50 (s, 1H), 1.29 (s, 1H), 1.08 (dt, 1H, J=4.3Hz, J = 12.9Hz).; UHPLC-MS (ES+APCI) m/z calcd. for $C_{15}H_{22}N_2O_2S$ $[M]^+$ = 294.14. Found: 295.20.

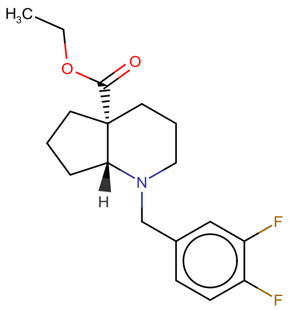

Ethyl 1-[(3,4-difluorophenyl)methyl]-octahydro-1H-cyclopenta[b]pyridine-4a-carboxylate (53b[iii]) 10.3 mg (21% yield).; ^{1}H NMR (300 MHz, $CDCl_3$): δ 7.24-7.18 (m, 1H), 7.09-6.99 (dd, J = 6.9Hz, J=16.6Hz, 2H), 4.18 (c, J = 1.0Hz, J = 7.1Hz, 2H,), 3.85 (d, J = 13.9Hz, 1H), 3.06 (d, J = 13.8Hz, 1H), 2.77 (dd, J = 3.0Hz, J=12.1Hz, 1H), 2.44 (d, J = 12.6Hz, 1H,), 2.18-2.07 (m, 2H), 1.98-1.87 (m, 1H), 1.85-1.61 (m, 4H), 1.57-1.43 (m, 3H), 1.29 (t, J = 7.1Hz, 3H), 1.07 (dt, J = 4.3Hz, J = 12.7Hz, 1H).; UHPLC-MS (ES+APCI) m/z calcd. for $C_{18}H_{23}F_2NO_2$ $[M]^+$ = 323.17. Found:324.2.

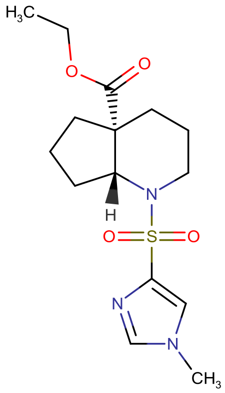

Ethyl 1-[(1-methyl-1H-imidazol-4-yl)sulfonyl]-octahydro-1H-cyclopenta[b]pyridine-4a-carboxylate (53c[iii]) 24.4 mg (47% yield).; ^{1}H NMR (300 MHz, $CDCl_3$): δ 7.45 (dd, J = 1.3, 14.3 Hz, 2H), 4.17 (q, J = 7.1 Hz, 2H), 4.00 – 3.96 (m, 1H), 3.75 (s, 3H), 2.76 (ddd, J = 7.3, 11.9, 23.7 Hz, 1H), 2.62 (dt, J = 3.5, 12.4 Hz, 1H), 2.46 (td, J = 6.1, 12.3 Hz, 2H), 2.22 – 2.11 (m, 1H), 2.00 (ddd, J = 2.5, 9.1, 13.0 Hz, 1H), 1.82 – 1.61 (m, 4H), 1.39 – 1.33 (m, 1H), 1.28 (t, J = 7.1 Hz, 3H), 1.07 (dt, J = 4.3, 13.1 Hz, 1H).; UHPLC-MS (ES+APCI) m/z calcd. for $C_{15}H_{24}N_3O_4S$ $[M]^+$ = 342.43. Found: 342.20.

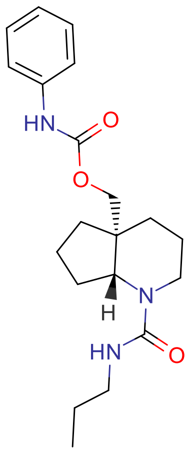

1-(Propylcarbamoyl)-hexahydro-2H-cyclopenta[b]pyridin-4a-yl]methyl N-phenylcarbamate (28a^{i}) 18 mg (46% yield).; ^{1}H NMR (300 MHz, $CDCl_3$): δ 7.34 – 7.32 (m, 2H), 7.24 (t, J = 7.5 Hz, 2H), 6.99 (t, J = 8,.5 Hz, 1H), 6.84 (bs, 1H), 4.56 (t, J = 4.8 Hz, 1H), 4.23 (d, J = 12.4 Hz, 1H), 3.96 – 3.86 (m, 2H), 3.42 (s, 2H), 3.07 (q, J = 7.1 Hz, 2H), 2.75 (dd, J = 7.1, 12.7 Hz, 1H), 2.64 (dt, J = 3.4, 12.7 Hz, 1H), 2.35 – 2.25 (m, 1H), 2.09 – 1.94 (m, 2H), 1.80 – 1.71 (m, 2H), 1.50 – 1.36 (m, 3H), 1.09 (dt, J = 4.5, 13.6 Hz, 2H), 0.84 (t, J = 7.1 Hz, 3H).; UHPLC-MS (ES+APCI) m/z calcd. for $C_{20}H_{30}N_3O_3$ $[M]^+$ = 360.46. Found:360.20.

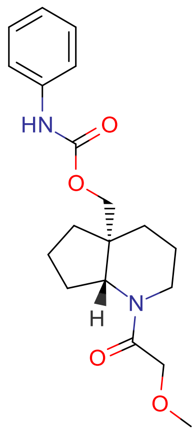

1-(2-Methoxyacetyl)-hexahydro-2H-cyclopenta[b]pyridin-4a-yl]methyl N-phenylcarbamate (28b^{i}) 15.5 mg (41% yield).; ^{1}H NMR (300 MHz, $CDCl_3$): δ 7.38 – 7.2 (m, 4H), 7.06 (t, J = 7.3 Hz, 1H), 6.74 (bs, 1H), 4.25 (dd, J = 1.6, 11.3 Hz, 1H), 4.11 – 3.97 (m, 4H), 3.43 (s, 3H), 2.91 – 2.77 (m, 2H), 2.69 – 2.55 (m, 1H), 2.42 – 2.32 (m, 1H), 2.17 (d, J = 13.3 Hz, 1H), 1.85 – 1.77 (m, 3H), 1.55 – 1.53 (m, 2H), 1.23 – 1.10 (m, 2H).; UHPLC-MS (ES+APCI) m/z calcd. for $C_{19}H_{27}N_2O_4$ $[M]^+$ = 347.42. Found: 347.20.

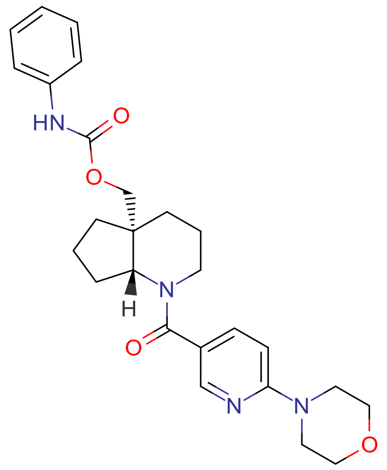

1-[6-(Morpholin-4-yl)pyridine-3-carbonyl]-hexahydro-2H-cyclopenta[b]pyridin-4a-yl]methyl N-phenylcarbamate (28c[i]) 18.9 mg (37% yield).; ^{1}H NMR (300 MHz, $CDCl_3$): δ 8.35 (d, J = 2.1 Hz, 1H), 7.66 (dd, J = 2.4, 8.8 Hz, 1H), 7.41 – 7.39 (m, 2H), 7.31 (t, J = 6.1 Hz, 2H), 7.07 (t, J = 6.1 Hz, 1H), 6.81 (bs, 1H), 6.59 (d, J = 8.8 Hz, 1H), 4.40 (dm, J = 11.3 Hz, 1H), 4.07 (d, J = 11.3 Hz, 2H), 3.81 (dd, J = 1.1, 4.6 Hz, 4H), 2.57 (dd, J = 1.1, 4.6 Hz, 4H), 3.06 (dd, J = 5.4, 7.3 Hz, 1H), 2.88 (dt, J = 3.2, 13.3 Hz, 1H), 2.47 – 2.37 (m, 1H), 2.23 – 2.08 (m, 2H), 1.94 – 1.66 (m, 4H), 1.54 (d, J = 13.3 Hz, 1H), 1.30 – 1.13 (m, 2H).; UHPLC-MS (ES+APCI) m/z calcd. for $C_{26}H_{33}N_4O_4$ $[M]^+$ = 465.56. Found: 465.20.

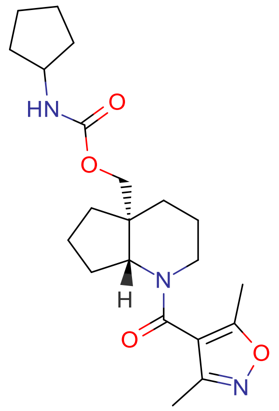

1-(3,5-Dimethyl-1,2-oxazole-4-carbonyl)-hexahydro-2H-cyclopenta[b]pyridin-4a-yl]methyl N-cyclopentylcarbamate (28d[i]) 19.6 mg (45% yield).; ^{1}H NMR (300 MHz, $CDCl_3$): δ 4.63 (bs, 1H), 4.27 (d, J = 11.0 Hz, 1H), 3.98 – 3.83 (m, 3H), 2.98 (t, J = 10.7 Hz, 1H), 2.87 (dt, J = 3.4, 13.3 Hz, 1H), 2.44 (s, 3H), 2.38 – 2.33 (m, 2H), 2.30 (s, 3H), 2.14 (d, J = 12.7 Hz, 1H), 1.98 – 1.76 (m, 5H), 1.71 – 1.53 (m, 6H), 1.42 – 1.35 (m, 2H), 1.19 – 1.12 (m, 2H).; UHPLC-MS (ES+APCI) m/z calcd. for $C_{21}H_{32}N_3O_4$ $[M]^+$ = 390.49. Found: 390.20.

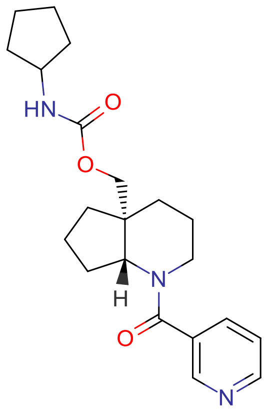

1-(Pyridine-3-carbonyl)-hexahydro-2H-cyclopenta[b]pyridin-4a-yl]methyl N-cyclopentylcarbamate (28e[i]) 21.2 mg (51% yield).; ^{1}H NMR (300 MHz, $CDCl_3$): δ 8.67 – 8.62 (m, 2H), 7.76 (dt, J = 1.9, 7.8 Hz, 1H), 7.33 (ddd, J = 0.6, 4.9, 7.8 Hz, 1H), 4.71 (bs, 1H), 4.25 (d, J = 10.7 Hz, 1H), 3.98 (t, 11.7 Hz, 3H), 3.47 (s, 2H), 3.03 (dd, J = 7.4, 12.8 Hz, 1H), 2.87 (dt, J = 3.2, 13.3 Hz, 1H), 2.20 – 2.15 (m, 2H), 1.98 – 1.81 (m, 4H), 1.71 – 1.53 (m, 6H), 1.46 – 1.35 (m, 2H), 1.23 – 1.12 (m, 2H).; UHPLC-MS (ES+APCI) m/z calcd. for $C_{21}H_{30}N_3O_3$ $[M]^+$ = 372.47. Found: 372.20.

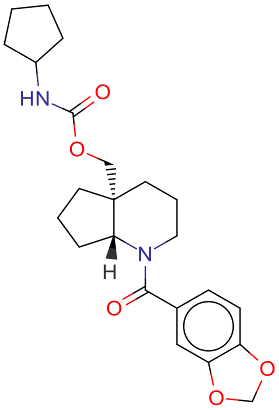

1-(2H-1,3-Benzodioxole-5-carbonyl)-hexahydro-2H-cyclopenta[b]pyridin-4a-yl]methyl N-cyclopentylcarbamate (28f[i]) 20.8 mg (45% yield).; ^{1}H NMR (300 MHz, $CDCl_3$): δ 6.99 (dd, J = 1.6, 7.9 Hz, 1H), 6.94 (d, J = 1.6 Hz, 1H), 6.79 (d, J = 7.9 Hz, 1H), 5.98 (s, 2H), 4.67 (bs, 1H), 4.29 (d, J = 11.0 Hz, 1H), 4.06 – 3.92 (m, 3H), 2.96 (dd, J = 7.4, 12.8 Hz, 1H), 2.79 (dt, J = 3.2, 13.2 Hz, 1H), 2.39 – 2.35 (m, 1H), 2.18 – 2.13 (m, 2H), 2.00 – 1.81 (m, 5H), 1.69 – 1.58 (m, 5H), 1.51 – 1.37 (m, 3H), 1.20 – 1.11 (m, 2H).; UHPLC-MS (ES+APCI) m/z calcd. for $C_{23}H_{31}N_2O_5$ $[M]^+$ = 415.50. Found: 415.20.

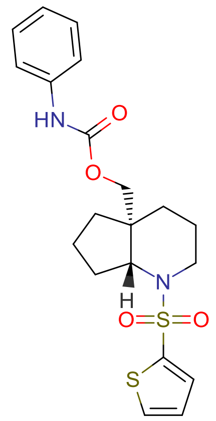

1-(Thiophene-2-sulfonyl)-hexahydro-2H-cyclopenta[b]pyridin-4a-yl]methyl N-phenylcarbamate (28g[ii]) 21.4 mg (47% yield).; ^{1}H NMR (300 MHz, $CDCl_3$): δ 7.63 (dd, J = 1.2, 5.0 Hz, 1H), 7.54 (dd, J = 1.3, 3.7 Hz, 1H), 7.40 – 7.37 (m, 2H), 7.31 (t, J = 7.4 Hz, 2H), 7.15 (dd, J = 3.8, 5.0 Hz, 1H), 7.06 (t, J = 7.3 Hz, 1H), 6.62 (bs, 1H), 4.46 (dd, J = 1.8, 11.4 Hz, 1H), 4.11 (d, J = 11.4 Hz, 1H), 4.0 (dd, J = 3.7, 11.1 Hz, 1H), 2.3 (dt, J = 3.2, 12.6 Hz, 1H), 2.22 – 2.09 (m, 4H), 2.04 – 1.83 (m, 3H), 1.69 – 1.63 (m, 2H), 1.10 – 0.91 (m, 2H).; UHPLC-MS (ES+APCI) m/z calcd. for $C_{20}H_{25}N_2O_4S_2$ $[M]^+$ = 421.55. Found: 421.20.

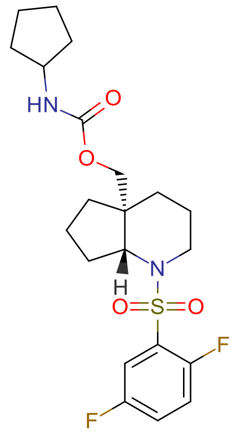

1-(2,5-difluorobenzenesulfonyl)-hexahydro-2H-cyclopenta[b]pyridin-4a-yl]methyl N-cyclopentylcarbamate (28h[ii]) 19.8 mg (40% yield).; ^{1}H NMR (300 MHz, $CDCl_3$): δ 7.59 – 7.54 (m, 1H), 7.24 – 7.15 (m, 2H), 4.57 (bs, 1H), 4.19 (d, J = 11.2 Hz, 1H), 4.12 (dd, J = 3.7, 12.2 Hz, 1H), 3.95 – 3.87 (m, 2H), 2.65 (tt, J = 3.0, 12.8 Hz, 1H), 2.55 (t, J = 9.9 Hz, 1H), 2.12 – 1.76 (m, 8H), 1.68 – 1.56 (m, 6H), 1.43 – 1.37 (m, 2H), 1.04 – 0.96 (m, 2H).; UHPLC-MS (ES+APCI) m/z calcd. for $C_{21}H_{29}F_2N_2O_4S$ $[M]^+$ = 443.52. Found: 443.20.

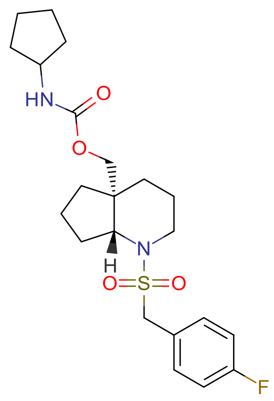

1-[(4-Fluorophenyl)methanesulfonyl]-hexahydro-2H-cyclopenta[b]pyridin-4a-yl]methyl N-cyclopentylcarbamate (28i[ii]) 15.3 mg (31% yield).; ^{1}H NMR (300 MHz, $CDCl_3$): δ 7.30 (dd, J = 5.3, 8.7 Hz, 2H), 7.00 (t, J = 8.7 Hz, 2H), 4.50 (bs, 1H), 4.13 (d, J = 10.9 Hz, 1H), 4.07 (s, 2H), 3.88 – 3.77 (m, 3H), 2.56 (dd, J = 7.3, 12.4 Hz, 1H), 2.42 (dt, J = 3.2, 12.4 Hz, 1H), 2.05 – 2.00 (m, 2H), 1.89 – 1.71 (m, 6H), 1.60 – 1.45 (m, 6H), 1.37 – 1.25 (m, 2H), 1.01 – 0.87 (m, 2H).; UHPLC-MS (ES+APCI) m/z calcd. for $C_{22}H_{32}FN_2O_4S$ $[M]^+$ = 439.56. Found: 439.20.

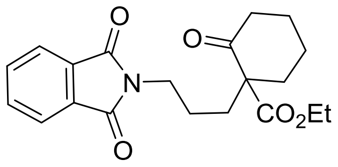

Chemical Formula: $C_{20}H_{23}NO_5$
Exact Mass: 357.16
Molecular Weight: 357.41

Ethyl 1-(3-(1,3-dioxoisoindolin-2-yl)propyl)-2-oxocyclohexane-1-carboxylate (24b): NaH (60% in mineral oil) (2.76 g; 69.09 mmol) was suspended in a mixture of dry THF-DMF (1:1; 200 mL), Keto-ester **23b** (10.0 g; 57.56 mmol) dissolved in dry THF was added dropwise and 0 °C, the resulting solution allowed to stir for 10 min to complete deprotonation. Then bromopropyl-pthalimide **16** (17.3 g; 63.33 mmol) in dry THF was added slowly at 0 °C. Then the reaction mixture was fitted with a refluxing condenser and heated at 65 °C for overnight. The reaction completion was monitored by TLC and cooled to room temp and then the pH of the reaction mixture was adjusted to neutral with 1.0M aq. HCl. The layers were separated and aq. layer was extracted with EtOAc, combined organic layer was washed with water, brine solution dried over anhydrous Na_2SO_4. After concentration the crude residue was then purified by column chromatography using 10-15% EtOAc in pet-ether

as a gradient to give pure pthalimide derivative **(24b)** 14.8g 72% yield).; ^{1}H NMR (400 MHz, $CDCl_3$) δ 7.76 – 7.71 (m, 2H), 7.64 (ddd, *J* = 8.5, 4.6, 2.8 Hz, 2H), 4.15 – 4.07 (m, 2H), 3.58 (dd, *J* = 12.7, 5.5 Hz, 2H), 2.44 – 2.27 (m, 3H), 1.95 – 1.77 (m, 2H), 1.69 – 1.46 (m, 6H), 1.41 – 1.30 (m, 2H), 1.17 (dt, *J* = 11.1, 4.1 Hz, 3H).; ^{13}C NMR (101 MHz, $CDCl_3$) δ 207.6, 171.9, 168.4, 134.0, 132.3, 123.3, 61.5, 60.6, 41.2, 38.3, 36.3, 31.9, 27.7, 27.1, 23.8, 22.7, 14.3.; HPLC-MS (ESI) m/z calcd. for $C_{20}H_{24}NO_5$ $[M+H]^+$ = 358.16. Found: 358.10.
TLC stain: UV, KMnO4
R_f: 0.5 (30% EtOAc in pet-ether)

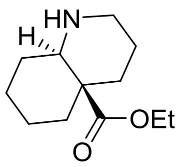

Chemical Formula: $C_{12}H_{21}NO_2$
Exact Mass: 211.16
Molecular Weight: 211.31

Ethyl octahydroquinoline-4a(2H)-carboxylate (25b): Pthalimide **24b** (14.8 g; 41.41 mmol) was dissolved in analytical grade ethanol, was added methyl amine (33% in ethanol) (11.7 g; 124.23 mmol) dropwise at rt. The reaction mixture was fitted with a refluxing condenser and refluxed at 85 °C for 3h. The reaction completion was monitored by TLC and cooled to rt. (the by-product di-amide precipitated out). The solid di-amide separated by filtration and filtrate was concentrated and filtered through silica column to give pure product 6.49g (69% yield); TLC stain: I_2, $KMnO_4$; R_f: 0.6 (10% MeOH (Buffered with aq. NH_3) in DCM.
The amino-ketone (11.7 g; 124.23 mmol) was dissolved in dry DCM (dilution is very important to get diastereo selectivity!!!) was added equivalent amount of oven dried powdered MS followed by $NaBH(OEh)_3$ (11.7 g; 124.23 mmol) in one portion, then allowed to stir for 18 h. at rt. After completion of SM was confirmed by TLC, diluted with DCM and saturated aq. $NaHCO_3$ was added, stirred for additional 15 min at rt. The DCM layer was washed with couple of times with saturated aq. $NaHCO_3$ then water and brine solution. After concentration the crude residue was filtered through silica column to get the mixture of pure diastereomers **25b** (10:1) 3.91 g, 67% yield.; ^{1}H NMR (400 MHz, CD_3OD) δ 4.20 (q, *J* = 7.1 Hz, 2H), 3.08 (ddd, *J* = 11.8, 3.8, 1.8 Hz, 1H), 2.70 – 2.61 (m, 1H), 2.38 (dd, *J* = 11.6, 4.3 Hz, 1H), 2.13 – 2.08 (m, 1H), 2.04 – 1.98 (m, 1H), 1.81 – 1.63 (m, 3H), 1.61 – 1.51 (m, 2H), 1.47 – 1.36 (m, 3H), 1.27 (t, *J* = 7.1 Hz, 3H), 1.18 – 1.10 (m, 2H).; ^{13}C NMR (101 MHz,

CD_3OD) δ 175.5, 62.7, 60.2, 47.5, 46.2, 36.4, 36.1, 29.0, 25.7, 23.7, 23.2, 13.4.; HPLC-MS (ESI) m/z calcd. for $C_{12}H_{22}NO_2$ $[M+H]^+$ = 212.16. Found: 212.17.

TLC stain: I_2, $KMnO_4$

R_f: 0.2 (10% MeOH (Buffered with aq. NH_3) in DCM)

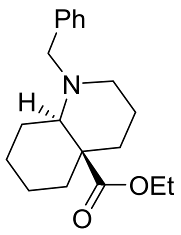

Chemical Formula: $C_{19}H_{27}NO_2$
Exact Mass: 301.20
Molecular Weight: 301.43

Ethyl 1-benzyloctahydroquinoline-4a(2H)-carboxylate (26'): The mixture of diastereomers **25b** (39.9 g; 18.46 mmol) were dissolved in dry DCM, was added benzaldehyde (2.2 g; 20.30 mmol) followed by $NaBH(OAc)_3$ (4.8 g; 22.15 mmol) stirred for 30 min at rt. The reaction mixture was diluted with DCM (184 mL) followed by saturated aq. $NaHCO_3$ and stirred for 10 min. The DCM layer was washed with couple of times with saturated aq. $NaHCO_3$ then water and brine solution. After concentration the crude residue was carefully purified by column chromatography using 2-10% of EtOAc in pet-ether as an eluent to give the pure diastereomer (**26'**)4.72g (85% yield).; ^1H NMR (600 MHz, $CDCl_3$) ^1H NMR (600 MHz, $CDCl_3$) δ 7.37 (d, *J* = 7.6 Hz, 2H), 7.31 (t, *J* = 7.4 Hz, 2H), 7.23 (t, *J* = 7.2 Hz, 1H), 4.30 – 4.19 (m, 2H), 4.09 (d, *J* = 13.9 Hz, 1H), 3.23 (d, *J* = 13.9 Hz, 1H), 2.85 (d, *J* = 10.8 Hz, 1H), 2.23 (d, *J* = 12.8 Hz, 1H), 2.18 (dt, *J* = 23.4, 8.4 Hz, 2H), 2.05 (t, *J* = 11.7 Hz, 1H), 1.97 – 1.83 (m, 3H), 1.60 (qt, *J* = 13.3, 3.8 Hz, 1H), 1.51 (d, *J* = 5.4 Hz, 1H), 1.44 (d, *J* = 13.4 Hz, 1H), 1.37 – 1.29 (m, 5H), 1.29 – 1.21 (m, 1H), 1.14 (td, *J* = 13.3, 3.8 Hz, 1H).; ^{13}C NMR (151 MHz, $CDCl_3$) δ 175.0, 141.1, 128.5, 128.0, 126.3, 69.4, 59.6, 56.6, 54.7, 48.6, 37.9, 37.4, 26.2, 25.9, 23.6, 22.2, 14.4.; HPLC-MS (ESI) m/z calcd. for $C_{19}H_{28}NO_2$ $[M+H]^+$ = 302.20; Found: 302.34.

TLC stain: UV, I_2, KMnO4

R_f: 0.6 (5% MeOH (Buffered with aq. NH_3) in DCM)

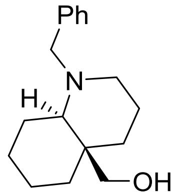

Chemical Formula: $C_{17}H_{25}NO$
Exact Mass: 259.19
Molecular Weight: 259.39

1-Benzyloctahydroquinolin-4a(2H)-yl)methanol (26b): To a suspension of LAH (0.35 g; 9.12 mmol) in dry THF (40 mL) was added amino-ester **26'**(2.5 g; 8.29 mmol) dissolve in dry THF at 0 °C. Then the reaction mixture was stirred for overnight, after completion of reaction monitored by TLC, the excess LAH was quenched with wet Na_2SO_4!!! (Few drops of water were added to Na_2SO_4 to make paste). The greyish white solid was filtered washed several times with EtOAc and the filtrated was concentrated to give pure product **(26b)** 2.02g, (94% yield).; ^{1}H NMR (400 MHz, CD_3OD) δ 7.42 – 7.08 (m, 5H), 4.26 (d, *J* = 11.1 Hz, 1H), 4.06 (t, *J* = 8.6 Hz, 1H), 3.63 (d, *J* = 11.2 Hz, 1H), 2.91 (t, *J* = 11.2 Hz, 1H), 2.87 – 2.80 (m, 1H), 2.23 – 2.13 (m, 1H), 2.05 – 1.84 (m, 4H), 1.76 (dd, *J* = 14.3, 3.0 Hz, 1H), 1.57 – 1.49 (m, 2H), 1.48 – 1.32 (m, 4H), 1.08 (td, *J* = 13.2, 4.7 Hz, 1H), 1.02 – 0.91 (m, 1H).; ^{13}C NMR (101 MHz, CD_3OD) δ 139.8, 128.5, 128.2, 126.7, 70.9, 63.8, 56.9, 54.5, 37.9, 37.6, 35.1, 25.8, 24.9, 22.4, 20.8.; HPLC-MS (ESI) m/z calcd. for $C_{17}H_{26}NO$ $[M+H]^+$ = 260.19. Found: 260.32.

TLC stain: UV, I_2, $KMnO_4$

R_f: 0.3 (10% MeOH (Buffered with aq. NH_3) in DCM)

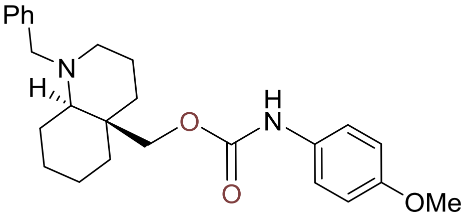

Chemical Formula: $C_{25}H_{32}N_2O_3$
Exact Mass: 408.24
Molecular Weight: 408.54

1-Benzyloctahydroquinolin-4a(2H)-yl)methyl (4-methoxyphenyl)carbamate (27'): The pure alcohol (1.7 g; 6.55 mmol) was dissolved in dry DCM (33 mL), was added Et_3N (0.99 g; 9.83 mmol) followed by 4-methoxy phenyl isocyanate (1.1 g; 7.21 mmol) and stirred for 4 h.

at rt. The reaction mixture was diluted with DCM, washed couple of times with saturated aq. $NaHCO_3$ then water and brine solution. After concentration the crude residue was purified by column chromatography using 2-10% of EtOAc in pet-ether as an eluent to give the pure carbamate **27**' 1.61 g 62% yield).; ^{1}H NMR (400 MHz, CD_3OD) δ 7.36 – 7.13 (m, 7H), 7.36 – 7.13 (m, 7H), 6.88 – 6.78 (m, 2H), 6.85 – 6.80 (m, 2H), 4.76 (d, *J* = 11.3 Hz, 1H), 4.48 (d, *J* = 11.3 Hz, 1H), 3.95 (d, *J* = 13.5 Hz, 1H), 2.94 (d, *J* = 13.5 Hz, 1H), 2.87 – 2.74 (m, 1H), 2.06 (dd, *J* = 12.0, 3.8 Hz, 1H), 1.97 – 1.77 (m, 6H), 1.65 – 1.48 (m, 1H), 1.48 – 1.31 (m, 3H), 1.05 – 0.86 (m, 2H). ^{13}C NMR (101 MHz, CD_3OD) δ 156.0, 155.8, 140.8, 132.1, 128.4, 128.0, 126.4, 120.6, 113.9, 70.2, 57.3, 55.1, 54.7, 38.1, 34.7, 34.4, 25.8, 25.3, 21.7, 20.6.; HPLC-MS (ESI) m/z calcd. for $C_{25}H_{33}N_2O_3$ $[M+H]^+$ = 409.24. Found: 409.42.

TLC stain: UV, I_2, $KMnO_4$

R_f: 0.5 (10% MeOH (Buffered with aq. NH_3) in DCM)

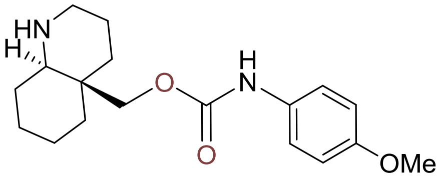

Chemical Formula: $C_{18}H_{26}N_2O_3$
Exact Mass: 318.19
Molecular Weight: 318.42

Octahydroquinolin-4a(2H)-yl)methyl (4-methoxyphenyl)carbamate (**27b**) : The carbamate **27'** (1.5 g; 3.49 mmol) was dissolved in MeOH (65 mL) and carefully 10% Pd/C was added under argon atmosphere. Then the reaction mixture was evacuated couple of times by applying vacuum and re-filling the hydrogen, stirred under H_2 atmosphere for overnight. After complete consumption SM, it was filtered through HPLC filter using syringe, washed couple of times with MeOH and concentrated to give pure product 130 mg (86% yield).; ^{1}H NMR (400 MHz, CD_3OD) δ 7.29 (t, *J* = 17.1 Hz, 2H), 6.91 – 6.79 (m, 2H), 4.55 – 4.40 (m, 2H), 3.74 (s, 3H), 3.04 (dd, *J* = 12.1, 4.4 Hz, 1H), 2.79 – 2.56 (m, 1H), 2.41 (dt, *J* = 13.7, 6.7 Hz, 1H), 1.83 (ddd, *J* = 24.5, 23.8, 13.9 Hz, 4H), 1.60 (dd, *J* = 14.9, 11.2 Hz, 1H), 1.51 – 1.33 (m, 5H), 1.11 – 0.85 (m, 2H).; ^{13}C NMR (101 MHz, CD_3OD) δ 156.0, 155.5, 132.1, 120.6, 113.9, 64.0, 61.4, 54.7, 37.1, 34.2, 34.2, 27.8, 25.6, 22.0, 20.9.; HPLC-MS (ESI) m/z calcd. for $C_{18}H_{27}N_2O_3$ $[M+H]^+$ = 319.19. Found: 319.30.

TLC stain: UV, I_2, $KMnO_4$

R_f: 0.2 (10% MeOH (Buffered with aq. NH_3) in DCM)

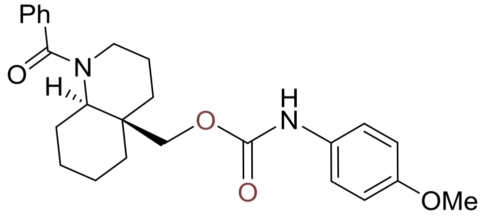

Chemical Formula: $C_{25}H_{30}N_2O_4$
Exact Mass: 422.22
Molecular Weight: 422.53

1-Benzoyloctahydroquinolin-4a(2H)-yl)methyl (4-methoxyphenyl)carbamate (28b): To amine **27b** (0.1 g; 0.3 mmol) dissolved in dry DCM (3 mL) was added Et_3N (0.05 g; 0.45 mmol), followed by BzCl (0.05 g; 0.33 mmol) dropwise at 0 °C, and stirred for 4 h. at rt. After completion of reaction, it was diluted with DCM, washed with couple of times with saturated aq. $NaHCO_3$ then water and brine solution. After concentration the crude residue was purified by column chromatography using 2-5% of MeOH (Buffered with aq. NH_3) in DCM as an eluent to give the pure sulphonamide **(28b)** 78 mg 62% yield).; ^{1}H NMR (400 MHz, DMSO) δ 9.35 (s, 1H), 7.38 (dddd, J = 6.8, 5.7, 4.4, 2.1 Hz, 7H), 6.97 – 6.74 (m, 2H), 4.51 (d, J = 11.8 Hz, 1H), 4.33 (d, J = 11.7 Hz, 1H), 3.69 (s, 3H), 3.58 (d, J = 12.5 Hz, 1H), 3.20 (dd, J = 12.6, 3.5 Hz, 1H), 3.06 (dd, J = 13.2, 10.7 Hz, 1H), 2.82 – 2.65 (m, 1H), 1.93 – 1.70 (m, 4H), 1.65 – 1.39 (m, 3H), 1.34 (d, J = 12.5 Hz, 1H), 1.29 – 1.07 (m, 2H), 1.07 – 0.90 (m, 1H).; ^{13}C NMR (101 MHz, DMSO) δ 171.6, 155.4, 154.7, 138.8, 133.0, 130.0, 129.1, 127.31, 120.4, 114.6, 67.2, 61.1, 55.9, 52.5, 35.1, 34.9, 27.2, 27.1, 23.7, 21.5.; HPLC-MS (ESI) m/z calcd. for $C_{25}H_{31}N_2O_4$ $[M+H]^+$ = 423.22. Found: 423.10.

TLC stain: UV, I_2, $KMnO_4$

R_f: 0.3 (10% MeOH (Buffered with aq. NH_3) in DCM

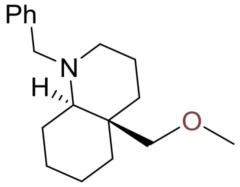

Chemical Formula: $C_{18}H_{27}NO$
Exact Mass: 273.21
Molecular Weight: 273.42

1-Benzyl-4a-(methoxymethyl)decahydroquinoline (29'): NaH (0.04 g; 0.88 mmol) was suspended in a mixture of dry THF-DMF (6 mL; 1:1), pure alcohol **(26b)** (0.2 g; 0.73 mmol) in dry THF (2 mL) was added dropwise and 0 °C, the resulting solution allowed to stir for 10 min to complete deprotonation. Then iodomethane (0.11 g; 0.81 mmol) was added slowly at 0 °C. The reaction mixture was stirred for overnight at rt. The reaction upon completion; pH of the reaction mixture was adjusted to neutral with 1.0M aq. HCl. extracted the reaction mixture with EtOAc, and the combined organic layer was washed with water, brine solution dried over anhydrous Na_2SO_4, concentrated to give the pure product. It was then purified by column chromatography using 10-15% EtOAc in pet-ether to give pure ether **(29')** 96 mg 46% yield).; ^{1}H NMR (600 MHz, $CDCl_3$) δ 7.33 (d, *J* = 4.1 Hz, 3H), 7.27 – 7.17 (m, 1H), 3.98 (t, *J* = 10.1 Hz, 2H), 3.63 (d, *J* = 9.6 Hz, 1H), 3.42 (s, 3H), 3.01 (d, *J* = 13.0 Hz, 1H), 2.85 (t, *J* = 26.2 Hz, 1H), 2.10 – 1.73 (m, 7H), 1.60 – 1.16 (m, 5H), 0.98 – 0.83 (m, 2H).; ^{13}C NMR (151 MHz, $CDCl_3$) δ 141.2, 128.4, 128.1, 126.4, 70.5, 70.1, 59.6, 57.5, 55.5, 38.5, 34.8, 34.7, 26.0, 25.3, 22.0, 20.8.; HPLC-MS (ESI) m/z calcd. for $C_{18}H_{28}NO$ $[M+H]^+$ = 274.21; Found: 274.29.

TLC stain: UV, I_2, $KMnO_4$

R_f: 0.6 (5% MeOH (Buffered with aq. NH_3) in DCM)

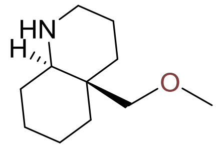

Chemical Formula: $C_{11}H_{21}NO$
Exact Mass: 183.16
Molecular Weight: 183.30

4a-(Methoxymethyl)decahydroquinoline (29b): The ether **29'** (0.1 g; mmol) was dissolved in MeOH (3 mL) and carefully 10% Pd/C was added under argon atmosphere. Then the reaction mixture was evacuated couple of times by applying vacuum and re-filling the hydrogen, stirred under H_2 atmosphere for overnight. After complete consumption SM, it was filtered through HPLC filter using syringe, washed couple of times with MeOH and concentrated to give pure product **(29b)** 56 mg (crude).; HPLC-MS (ESI) m/z calcd. for $C_{11}H_{21}NO$ $[M+H]^+$ = 184.16. Found: 184.15.

TLC stain: UV, I_2, $KMnO_4$

R_f: 0.2 (10% MeOH (Buffered with aq. NH_3) in DCM)

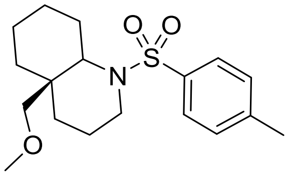

Chemical Formula: $C_{18}H_{27}NO_3S$
Exact Mass: 337.17
Molecular Weight: 337.48

4a-(Methoxymethyl)-1-tosyldecahydroquinoline (30b): Amine **29b** (0.05 g; 0.25 mmol) was dissolved in dry DCM (2 mL) was added Et_3N (0.03 g; 0.27 mmol), followed by TsCl (0.05 g; 0.27 mmol) in one portion at 0 °C, after 4 h. at rt., it was diluted with DCM, washed with couple of times with saturated aq. $NaHCO_3$ then water, brine solution and dried over Na_2SO_4. Then concentrated to give crude residue was purified by column chromatography using 2-5% of MeOH (Buffered with aq. NH_3) in DCM as an eluent to give the pure sulphonamide **(30b)** 47 mg (57% yield).; ^{1}H NMR (500 MHz, $CDCl_3$) δ 7.53 (d, J = 8.3 Hz, 2H), 7.26 – 7.15 (m, 2H), 4.27 – 4.17 (m, 1H), 3.63 (d, J = 9.8 Hz, 1H), 3.59 – 3.49 (m, 1H), 3.29 (d, J = 5.7 Hz, 2H), 2.69 – 2.51 (m, 1H), 2.34 (s, 2H), 1.97 – 1.67 (m, 3H), 1.52 – 1.44 (m, 1H), 1.35 (ddd, J = 24.3, 12.5, 7.9 Hz, 1H), 1.10 – 0.95 (m, 1H), 0.78 (dtd, J = 36.0, 13.3, 4.5 Hz, 1H).; ^{13}C NMR (126 MHz, $CDCl_3$) δ 142.7, 138.6, 129.7, 126.5, 68.9, 67.9, 59.6, 51.7, 38.8, 34.9, 34.6, 26.3, 26.0, 22.4, 21.5, 20.6.; HPLC-MS (ESI) m/z calcd. for $C_{18}H_{28}NO_3S$ $[M+H]^+$ = 338.17. Found: 338.19.
R_f = 0.4 (DCM/MeOH (9:1; MeOH : aq. NH_3) 9:1.

Experimentals for the production phase: Intermediates prepared in large scale

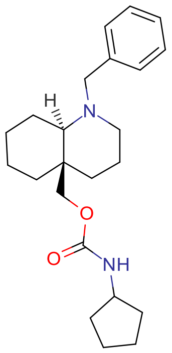

1-Benzyl-decahydroquinolin-4a-yl]methyl N-cyclopentylcarbamate (27b^i): To a solution of the alcohol **26b** (3.0 g, 12 mmol) in dry DCM (120 mL) was added triethylamine (1.8 g, 17 mmol) and cyclopentylisocyanate (1.5 g, 14 mmol) and the reaction mixture was stirred at

room temperature for 15 hours. After the complete consumption of the starting material monitored by TLC, the reaction mixture was washed with saturated aq. $NaHCO_3$ and brine. After concentration, the crude residue was purified by column chromatography using 15-20% of EtOAc in cyclohexane as an eluent to give the carbamate 3.2 g (75% yield).; ^{1}H NMR (300 MHz, $CDCl_3$): δ 7.30 – 7.10 (m, 5H), 4.68 (d, J = 7.2 Hz, 1H), 4.63 (d, J = 11.3 Hz, 1H), 3.92 (d, J = 13.4 Hz, 1H), 2.87 (d, J = 13.4, 1H), 2.79 (d, J = 9.7 Hz, 1H), 2.02 – 1.69 (m, 9H), 1.64 – 1.48 (m, 6H), 1.40 – 1.16 (m, 7H), 0.95 – 0.78 (m, 2H).; UHPLC-MS (ES+APCI) m/z calcd. for $C_{23}H_{35}N_2O_2$ $[M]^+$ = 371.53. Found: 371.15.

TLC stain: I_2, $KMnO_4$

R_f: 0.5 (Cyclohexane/EtOAc 3:1)

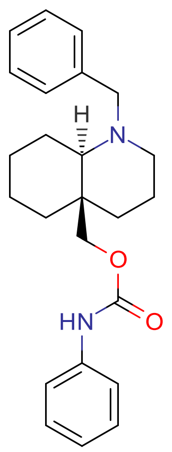

1-Benzyl-decahydroquinolin-4a-yl]methyl N-phenylcarbamate (27d^i): To a solution of the alcohol **26b** (3.0 g, 12 mmol) in dry DCM (120 mL) was added triethylamine (1.8 g, 17 mmol) and phemylisocyanate (1.6 g, 14 mmol) and the reaction mixture was stirred at room temperature for 15 hours. After the complete consumption of the starting material monitored by TLC, the reaction mixture was washed with saturated aq. $NaHCO_3$ and brine. After concentration, the crude residue was purified by column chromatography using 15-20% of EtOAc in cyclohexane as an eluent to give the carbamate 2.7 g (62% yield).; ^{1}H NMR (300 MHz, $CDCl_3$): δ 7.37 – 7.33 (m, 3H), 7.29 – 7.28 (m, 1H), 7.25 – 7.21 (m, 4H), 7.19 – 7.15 (m, 1H), 8.49 (t, J = 7.0 Hz, 1H), 4.77 (d, J = 11.3 Hz, 1H), 4.52 (d, J = 11.3 Hz, 1H), 3.95 (d, J = 13.6 Hz, 1H), 2.90 (d, J =13.6 Hz, 1H), 2.81 (d, J = 10.7 Hz, 1H), 2.04 (dd, J = 3.8, 11.8 Hz, 1H), 1.90 – 1.67 (m, 6H), 1.54 – 1.18 (m, 6H), 1.00 – 0.87 (m, 2H).; UHPLC-MS (ES+APCI) m/z calcd. for $C_{24}H_{31}N_2O_2$ $[M]^+$ = 379.51. Found: 378.95.

TLC stain: I_2, $KMnO_4$

R_f: 0.4 (Cyclohexane/EtOAc; 9:1)

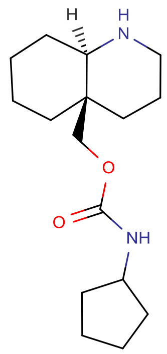

Decahydroquinolin-4a-yl]methyl N-cyclopentylcarbamate (27b): To a solution of the carbamate (**27b^{i})** (3.2 g, 9 mmol) in dry methanol (90 mL) was added palladium on charcoal (10 wt%) and the reaction mixture was stirred under hydrogen atmosphere overnight. After complete consumption of the starting material monitored by TLC, the reaction mixture was filtered through a pad of celite. Concentration of the filtrate afforded the pure product 2.3 g (93% yield).; ^{1}H NMR (300 MHz, $CDCl_3$): δ 4.40 (q, J = 11.0 Hz, 2H), 3.95 – 3.78 (m, 1H), 3.42 (s, 2H), 3.11 (d, J = 11.2 Hz, 1H), 2.69 (dt, J = 3.4, 12.2 Hz, 1H), 2.41 (dd, J = 4.6, 10.4 Hz, 1H), 2.28 – 2.18 (m, 2H), 1.93 – 1.85 (m, 2H), 1.79 – 1.72 (m, 2H), 1.64 – 1.51 (m, 5H), 1.46 – 1.32 (m, 6H), 1.02 – 0.82 (m, 2H).; UHPLC-MS (ES+APCI) m/z calcd. for $C_{16}H_{29}N_2O_2$ $[M]^+$ = 281.40. Found: 281.10.

TLC stain: UV, I_2, $KMnO_4$

R_f: 0.5 (15% MeOH (Buffered with aq. NH_3) in DCM)

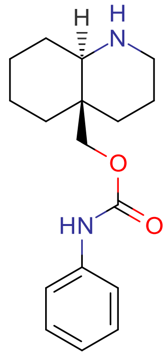

Decahydroquinolin-4a-yl]methyl N-phenylcarbamate (27d): To a solution of the carbamate (**27d^{i})** (2.7 g, 7 mmol) in dry methanol (70 mL) was added palladium on charcoal (10 wt%) and the reaction mixture was stirred under hydrogen atmosphere overnight. After

complete consumption of the starting material monitored by TLC, the reaction mixture was filtered through a pad of celite. Concentration of the filtrate afforded the pure product 2.0 g (96% yield).; ^1H NMR (300 MHz, $CDCl_3$): δ 7.55 (d, J = 7.5 Hz, 2H), 7.17 (t, J = 7.5 Hz, 2H), 6.95 (t, J = 7.5 Hz, 1H), 4.49 (d, J = 11.9 Hz, 1H), 4.26 (d, J = 11.9 Hz, 1H), 3.75 (d, J = 11.7 Hz, 1H), 3.40 (s, 1H), 3.02 – 2.87 (m, 2H), 2.24 – 2.15 (m, 2H), 1.87 – 1.67 (m, 4H), 1.51 – 1.10 (m, 6H).; UHPLC-MS (ES+APCI) m/z calcd. for $C_{17}H_{25}N_2O_2$ $[M]^+$ = 289.38. Found: 289.10.

TLC stain: I_2, $KMnO_4$

R_f: 0.5 (15% MeOH (Buffered with aq. NH_3) in DCM)

Experimentals for the final diversification:

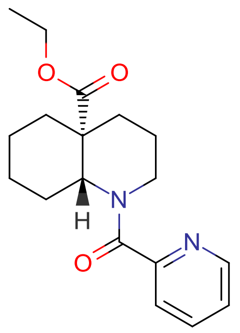

Ethyl 1-(pyridine-2-carbonyl)-octahydroquinoline-4a-carboxylate (53d^i) 16.8 mg (34% yield).; ^1H NMR (300 MHz, $CDCl_3$): δ 8.51 (ddd, J = 0.9, 1.6, 4.9 Hz, 1H), 7.74 (dt, J = 1.7, 7.7 Hz, 1H), 7.56 (d, J = 7.8 Hz, 1H), 7.25 (ddd, J = 1.2, 4.9, 7.6 Hz, 1H), 4.21 (q, J = 7.1 Hz, 2H), 3.96 (d, J = 14.2 Hz, 1H), 3.21 (dd, J = 3.8, 12.4 Hz, 1H), 2.96 (ddd, J = 3.0, 12.1, 13.5 Hz, 1H), 2.42 – 2.33 (m, 1H), 2.22 – 2.12 (m, 3H), 1.81 – 1.68 (m, 2H), 1.52 – 1.41 (m, 3H), 1.34 – 1.32 (m, 1H), 1.28 (t, J = 7.1 Hz, 3H), 1.22 – 1.13 (m, 2H).; UHPLC-MS (ES+APCI) m/z calcd. for $C_{18}H_{25}N_2O_3$ $[M]^+$ = 317.39. Found: 317.20.

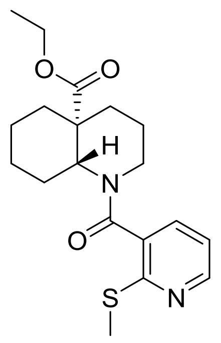

Ethyl -1-[2-(methylsulfanyl)pyridine-3-carbonyl]-octahydroquinoline-4a-carboxylate (53e[i]) 25.5 mg (45% yield).; ^{1}H NMR (300 MHz, $CDCl_3$): δ 8.41 (dd, J = 1.8, 4.9 Hz, 1H), 7.54 – 7.52 (m, 1H), 7.01 (dd, J = 4.9, 7.5 Hz, 1H), 4.23 (q, J = 6.9 Hz, 2H), 3.76 – 3.74 (m, 1H), 3.21 (dd, J = 3.5, 12.3 Hz, 1H), 2.96 (dt, J = 3.1, 11.4 Hz, 1H), 2.55 (s, 3H), 2.31 – 2.14 (m, 3H), 1.85 – 1.47 (m, 5H), 1.36 – 1.20 (m, 7H).; UHPLC-MS (ES+APCI) m/z calcd. for $C_{19}H_{27}N_2O_3S$ $[M]^+$ = 363.49. Found: 363.20.

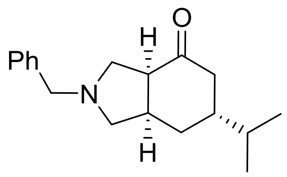

Chemical Formula: $C_{18}H_{25}NO$
Exact Mass: 271.19
Molecular Weight: 271.40

2-Benzyl-6-isopropyloctahydro-4H-isoindol-4-one (32a): *N*-(Methoxymethyl)-*N*-(trimethylsilylmethyl)benzylamine (10.9 g; 43.41 mmol) added to *des*-methyl carvone **32** (5.0 g; 36.17 mmol) in DCM (180 mL) followed by TFA (0.83 g; 0.54 mmol) at 0 °C. After 2 h $NaHCO_3$ was added stirred for 15 min. The layers were separated and aq. layer was extracted twice with DCM then washed with water, brine and dried over Na_2SO_4. After evaporation, the crude residue was purified by silica column using 2% MeOH (9 : 1; MeOH : aq. NH_3) in DCM.; ^{1}H NMR (400 MHz, CD_3OD) δ 7.27 – 7.18 (m, 5H), 3.59 – 3.51 (m, 2H), 3.01 – 2.93 (m, 1H), 2.83 – 2.67 (m, 4H), 2.37 – 2.30 (m, 2H), 2.23 (t, *J* = 9.0 Hz, 1H), 2.13 – 2.05 (m, 1H), 1.72 – 1.61 (m, 3H), 1.55 – 1.46 (m, 1H), 0.86 (dd, *J* = 6.8, 3.1 Hz, 6H).; ^{13}C NMR (101 MHz, CD_3OD) δ 170.0, 138.4, 128.9, 128.1, 128.1, 127.1, 120.4, 60.3, 58.0, 58.0, 54.6, 54.5, 44.1, 43.9, 39.2, 39.8, 37.9, 37.9, 32.1, 28.8, 18.7, 18.5.; HR-MS (ESI) m/z calcd. for $C_{18}H_{26}ON$ $[M+H]^+$ = 272.2014; Found: 272.2008.

R_f = 0.4 (DCM/MeOH (9:1; MeOH : aq. NH_3) 9:1.

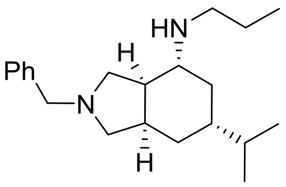

Chemical Formula: $C_{21}H_{34}N_2$
Exact Mass: 314.27
Molecular Weight: 314.52

2-Benzyl-6-isopropyl-N-propyloctahydro-1H-isoindol-4-amine (33b): To flam dried MS (1.0 g), added ketone **32a** ((1.0 g; 3.68 mmol)) in 1,2-DCM (72 mL) followed by *n*-propyl amine (0.24 g; 4.05 mmol) stirred for 45 min. Then $(NaBH(OEh)_3$ (4.4 g; 7.37 mmol) was added and stirred for 18 h. Then saturated aq. $NaHCO_3$ solution (~30 mL) was added and stirred for 15 min. The DCM layer was collected and the aqueous layer was extracted twice with DCM, washed with water, brine dried over Na_2SO_4. The solvent evaporated to afford crude product, which was then purified by silica column using 10-15% MeOH/DCM (9 : 1; MeOH : aq. NH_3).; ^{1}H NMR (400 MHz, CD_3OD) δ 7.42 – 7.17 (m, 5H), 3.75 – 3.63 (m, 2H), 3.03 – 2.87 (m, 2H), 2.78 – 2.69 (m, 1H), 2.56 (ddd, *J* = 14.6, 11.8, 7.1 Hz, 4H), 2.41 (d, *J* = 7.1 Hz, 1H), 2.32 (dd, *J* = 9.7, 5.1 Hz, 1H), 1.74 – 1.29 (m, 8H), 0.90 (td, *J* = 7.1, 3.9 Hz, 9H).; ^{13}C NMR (101 MHz, CD_3OD) δ 138.4, 129.2, 128.1, 127.1, 60.8, 59.3, 52.4, 51.9, 49.0, 39.6, 37.4, 34.6, 29.8, 28.2, 28.1, 22.4, 19.7, 19.3, 10.9.; HR-MS (ESI) m/z calcd. for $C_{21}H_{35}N_2$ $[M+H]^+$ = 315.2799. Found: 315.2794.

R_f = 0.2 (DCM/MeOH (8.5 : 1.5; MeOH : aq. NH_3) 9:1.

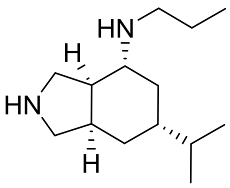

Chemical Formula: $C_{14}H_{28}N_2$
Exact Mass: 224.23
Molecular Weight: 224.39

6-Isopropyl-N-propyloctahydro-1H-isoindol-4-amine (34b): To the amine **xx** (0.83 g; 2.64 mmol) in MeOH (26 mL), was added $Pd(OH)_2$ (10 wt.%) under argon atmosphere. Then the flask was filled with H_2 using balloon and stirred for 18 h. at rt. The reaction mixture was filtered and concentrated to give pure product was taken for next step without further purification.; ^{1}H NMR (400 MHz, CD_3OD) δ 3.08 – 2.83 (m, 4H), 2.67 (d, *J* = 10.8 Hz, 1H),

2.57 (dd, J = 9.2, 6.0 Hz, 2H), 2.52 – 2.42 (m, 1H), 2.24 (td, J = 11.6, 5.8 Hz, 1H), 1.91 – 1.69 (m, 2H), 1.63 (d, J = 13.9 Hz, 1H), 1.58 – 1.46 (m, 2H), 1.44 – 1.25 (m, 3H), 1.01 – 0.86 (m, 9H).; ^{13}C NMR (101 MHz, CD_3OD) δ 52.0, 51.2, 49.0, 43.0, 42.2, 40.4, 35.1, 28.4, 26.8, 22.6, 20.5, 20.8, 11.0.; HR-MS (ESI) m/z calcd. for $C_{14}H_{29}N_2$ $[M+H]^+$ = 225.2332. Found: 225.2325.

R_f = 0.2 (DCM : MeOH (8.5 : 1.5; MeOH : aq. NH_3) 9:1.

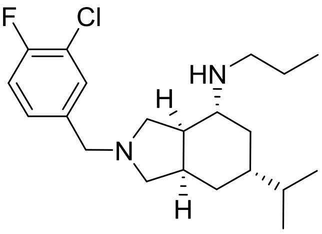

Chemical Formula: $C_{21}H_{32}ClFN_2$
Exact Mass: 366.22
Molecular Weight: 366.95

2-(3-Chloro-4-fluorobenzyl)-6-isopropyl-N-propyloctahydro-1H-isoindol-4-amine (35): To amine **34b** (56 mg; 0.25 mmol) in DCM (5 mL) added aldehyde (42 mg g; 0.26 mmol) BzOH (152 mg; 1.24 mmol), $NaBH(OAc)_3$ (64 mg; 0.29 mmol) and stirred for 1 h. Saturated $NaHCO_3$ (~3 mL) was added for 15 min. Layers were separated and the aqueous layer was extracted twice with DCM. The combined DCM extracts were washed with water, brine, dried over anhydrous Na_2SO_4, concentrated to afford pure product **35**. ^{1}H NMR (400 MHz, CD_3OD) δ 7.49 – 7.00 (m, 3H), 3.69 (d, J = 9.0 Hz, 2H), 2.95 (d, J = 6.8 Hz, 2H), 2.78 – 2.48 (m, 5H), 2.45 – 2.23 (m, 2H), 1.84 – 1.29 (m, 8H), 0.93 (ddd, J = 17.9, 9.2, 3.7 Hz, 9H). ^{13}C NMR (101 MHz, CD_3OD) δ 159.3, 156.8, 140.8, 130.3, 125.5, 118.9, 116.9, 114.6, 112.5, 59.6, 52.3, 48.9, 39.7, 37.7, 34.8, 29.5, 28.3, 22.3, 19.9, 19.4, 10.9.; HR-MS (ESI) m/z calcd. for $C_{21}H_{33}N_2ClF$ $[M+H]^+$ = 367.2319; Found: 367.2310.

R_f = 0.4 (DCM/MeOH (9:1; MeOH:aq. NH_3) 9:1.

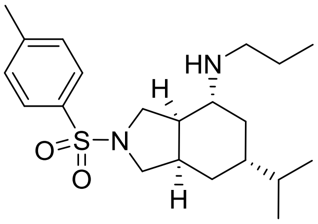

Chemical Formula: $C_{21}H_{34}N_2O_2S$
Exact Mass: 378.23
Molecular Weight: 378.58

6-Isopropyl-N-propyl-2-tosyloctahydro-1H-isoindol-4-amine (37): To the compound **34b** (50 mg; 0.22 mmol) in DCM (5 mL), added DIPEA (43 mg; 0.33 mmol) followed by TsCl (44 mg; 0.23 mmol) at rt. After 4 h., saturated $NaHCO_3$ was added followed by usual workup to give pure product **(37)** 84 mg (78% yield).; ^1H NMR (400 MHz, CD_3OD) δ 7.81 – 7.70 (m, 2H), 7.41 (dd, *J* = 9.7, 9.1 Hz, 2H), 3.37 – 3.23 (m, 3H), 3.13 – 3.01 (m, 2H), 2.65 – 2.55 (m, 1H), 2.44 (s, 3H), 2.24 (dt, *J* = 17.8, 5.4 Hz, 1H), 1.85 (d, *J* = 13.2 Hz, 1H), 1.68 – 1.47 (m, 5H), 1.35 – 1.19 (m, 3H), 0.96 – 0.83 (m, 11H).; ^{13}C NMR (101 MHz, CD_3OD) δ 144.0, 134.140, 129.7, 127.4, 54.3, 51.3, 45.4, 40.4, 34.2, 31.1, 29.3, 28.1, 27.2, 26.0, 24.8, 22.4, 21.8, 20.4, 20.2, 13.1, 10.8.; HR-MS (ESI) m/z calcd. for $C_{21}H_{35}\ O_2N_2S$ $[M+H]^+$ = 379.2422; Found: 379.2413.

R_f = 0.4 (DCM : MeOH (8.5:1.5; MeOH : aq. NH_3) 9:1.

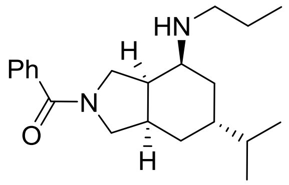

Chemical Formula: $C_{21}H_{32}N_2O$
Exact Mass: 328.25
Molecular Weight: 328.50

6-Isopropyl-4-(propylamino)octahydro-2H-isoindol-2-yl)(phenyl)methanone (36): To benzoic acid (34 mg; 0.27 mmol) in DMF (3 mL), was added DIEPA (48 mg; 0.37 mmol), HATU (107 mg; 0.27 mmol) followed by diamine **34b** (56 mg; 0.25 mmol), the resultant mixture was heated at 50 °C for 5h. Water was added to the cooled reaction mixture and extracted several times with EtOAc. The EtOAc layer was washed with water, brine and dried over anhydrous Na_2SO_4. Concentrated to give crude product, which was then purified by silica column using 10-15% MeOH:DCM (9:1; MeOH : aq. NH_3).; ^1H NMR (400 MHz, CD_3OD) δ 7.57 – 7.43 (m, 3H), 7.16 (tt, *J* = 20.1, 7.3 Hz,1H), 3.76 – 3.58 (m, 2H), 3.55 – 3.34 (m, 2H), 3.17 (d, *J* = 10.8 Hz, 1H), 3.00 – 2.92 (m, 1H), 2.85 – 2.70 (m, 1H), 2.85 – 2.70 (m, 1H), 2.58 – 2.34 (m, 1H), 2.05 (dd, *J* = 22.2, 13.3 Hz,1H), 1.91 – 1.40 (m, 6H), 1.38 – 1.19 (m, 1H), 1.08 – 0.86 (m, 10H); HR-MS (ESI) m/z calcd. for $C_{21}H_{33}ON_2$ $[M+H]^+$ = 329.2596. Found: 329.2587.

R_f = 0.3 (DCM : MeOH (8.5 : 1.5; MeOH : aq. NH_3) 9:1.

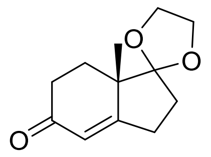

Chemical Formula: $C_{12}H_{16}O_3$
Exact Mass: 208.11
Molecular Weight: 208.26

(*S*)-7a-methyl-2,3,7,7a-tetrahydrospiro[indene-1,2'-[1,3]dioxolan]-5(6H)-one (39): To the diketone **38** (4.183g, 22.92 mmol) were added 4Å MS, dry MED (45mL), and anhydrous ethylene glycol (45 mL). To this two phases mixture was added PTSA.H_2O (4.0 g, 20.63 mmol) at one portion. The mixture was stirred at rt. for 2 h. The reaction was monitored by GC-MS. Then, the reaction mixture was poured into the aq. solution of $NaHCO_3$ and the aqua layer was extracted couple of times with EtOAc. The combined organic layers were washed with brine, dried over $MgSO_4$, concentrated under reduced pressure and the crude product was purified through flash chromatography pet-ether: acetone (10:1) to afford colorless oil **39** in 88% yield.; ^{1}H NMR (400 MHz, $CDCl_3$) δ 5.78 (s, 1H), 3.98 – 3.87 (m,4H), 2.67 (ddt, *J* = 19.4, 11.5, 2.4 Hz, 1H), 2.58 – 2.51 (m, 1H), 2.51 – 2.46 (m, 1H), 2.40 (dd, *J* = 5.3, 2.1 Hz, 1H), 2.35 (dd, *J* = 5.3, 2.0 Hz, 1H), 2.32 – 2.23 (m, 1H), 2.16 (ddd, *J* = 13.6, 11.1, 9.0 Hz, 1H), 1.90 (ddd, *J* = 13.6, 9.4, 2.7 Hz, 1H), 1.60 (ddd, *J* = 12.7, 5.3, 2.1 Hz, 1H), 1.25 (s, 3H).; GC-MS (ESI) m/z calcd. for $C_{12}H_{16}O_3$ $[M]^+$ = 208.11; Found: 208.10.
R_f = 0.35 in pet-ether: acetone (6:1).

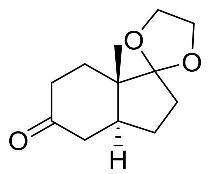

Chemical Formula: $C_{12}H_{18}O_3$
Exact Mass: 210.13
Molecular Weight: 210.27

7a-Methylhexahydrospiro[indene-1,2'-[1,3]dioxolan]-5(4H)-one (39a): The flask was charged with Pd/C (10% Pd/C, 425 mg) under argon. The solution of unsaturated ketone **39** (3.078g, 14.78 mmol) in MeOH (160 mL) was added to the black suspension under argon. The mixture was filled with H_2 and stirred for 2 hours. The reaction was monitored by TLC and GC-MS. The catalyst was filtered through celite, the solvent was evaporated in vacuo to afford the desired product **(39a)** 3.066g (99% yield).; ^{1}H NMR (400 MHz, $CDCl_3$) δ 3.91 – 3.82 (m, 4H), 3.10 (t, *J* = 7.1 Hz, 1H), 2.46 – 2.15 (m, 5H), 1.95 – 1.75 (m, 4H), 1.67 – 1.60

(m, 1H), 1.37 – 1.24 (m, 1H), 1.10 (d, J = 0.5 Hz, 3H).; GC-MS (ESI) m/z calcd. for $C_{12}H_{18}O_3$ $[M]^+$ = 210.13. Found: 210.10.

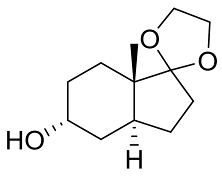

Chemical Formula: $C_{12}H_{20}O_3$
Exact Mass: 212.14
Molecular Weight: 212.29

7a-Methyloctahydrospiro[indene-1,2'-[1,3]dioxolan]-5-ol: The solution of L-Selectride (1M in THF, 18 mL, 18 mmol) was cooled to 0 °C. The solution of ketone **39a** (3.0 g, 14.33 mmol) in anhydrous THF (60 mL) was added at 0 °C, stirred for 1 hour. The reaction process was monitored by TLC and GC-MS. The reaction was quenched with water, and 20% KOH solution (10 mL), 30% H_2O_2 solution (10 mL) were added (exothermic) at rt. The mixture was stirred at rt. for additional 1 h and extracted with diethyl ether couple of tims, the combined organic layers were washed with brine and dried over $MgSO_4$, concentrated in vacuo. The product was purified through column chromatography using pet-ether-EtOAc (2:1) as a gradient to afford colorless oil **(40)** 2.652g (87% yield).; ^{1}H NMR (400 MHz, $CDCl_3$) δ 3.89 – 3.68 (m, 5H), 1.92 – 1.48 (m, 10H), 1.20 – 1.10 (m, 1H), 0.87 (s, 3H).; GC-MS (ESI) m/z calcd. for $C_{12}H_{20}O_3$ $[M]^+$ = 212.14. Found: 212.12.
R_f = 0.34 in pet-ether: EtOAc (1.5:1)

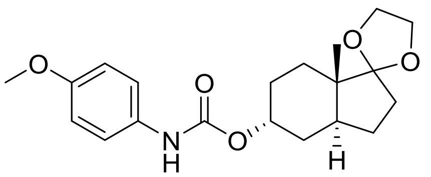

Chemical Formula: $C_{20}H_{27}NO_5$
Exact Mass: 361.19
Molecular Weight: 361.44

7a-Methyloctahydrospiro[indene-1,2'-[1,3]dioxolan]-5-yl (4-methoxyphenyl)carbamate (40a): To a solution of alcohol **40** (1.0 g, 4.71 mmol) in anhydrous THF (30 mL) were added *p*-methoxyphenyl isocyanate (1.065g ,7.07 mmol) and Et_3N (1.43 g, 14.13 mmol). The mixture was stirred at rt. for overnight and the reaction was monitored by TLC and LC-MS. The solvent was removed in vacuo and crude product was dissolved in DCM for flash

chromatography. The product was purified through flash chromatography (pet-ether: EtOAc = 5:1) to afford light brown oil **(40a)** 1.2g (70% yield).; ^{1}H NMR (400 MHz, $CDCl_3$) δ 7.26 (s, 2H), 6.88 – 6.79 (m,2H), 6.44 (s, 1H), 3.95 – 3.85 (m, 4H), 3.77 (s, 3H), 1.90 – 1.71 (m, 10H), 1.33 – 1.18 (m, 2H), 0.95 (s, 3H).; HPLC-MS (ESI) m/z calcd. for $C_{20}H_{28}NO_5$ $[M+H]^+$ = 362.19; Found: 362.21.

R_f = 0.42 in pet-ether: EtOAc (3:1)

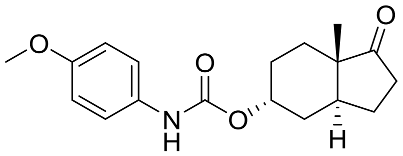

Chemical Formula: $C_{18}H_{23}NO_4$
Exact Mass: 317.1627
Molecular Weight: 317.3850

7a-Methyl-1-oxooctahydro-1H-inden-5-yl (4-methoxyphenyl)carbamate (41): The Ketal **40a** (1.2 g, 3.38 mmol) was dissolved in acetone-water (15:1, 32 mL), and then PPTS (283 mg, 1.13 mmol) was added. The resulting mixture was refluxed for 3 h. and the reaction was monitored by TLC and LC-MS. Upon completion the solvent acetone was removed in vacuo and the residue was dissolved in EtOAc, washed with aq. $NaHCO_3$ solution, brine, dried over $MgSO_4$, concentrated to give brown foamy solid. The crude product was purified by column chromatography using pet-ether: EtOAc (5:1) to afford the yellowish foamy solid **(41)** 945 mg (90% yield).; HPLC-MS (ESI) m/z calcd. for $C_{18}H_{24}NO_5$ $[M+H]^+$ = 318.16. Found: 318.11.

R_f = 0.41 in pet-ether: EtOAc (3:1).

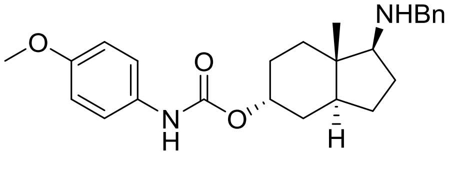

Chemical Formula: $C_{25}H_{32}N_2O_3$
Exact Mass: 408.2413
Molecular Weight: 408.5420

1-(Benzylamino)-7a-methyloctahydro-1H-inden-5-yl (4-methoxyphenyl)carbamate (43): To $NaBH(OEh)_3$ (0.424 g, 0.91 mmol) were added the solution of ketone **41** (145 mg, 0.46 mmol) in dry DCM followed by benzyl amine (73 mg, 0.68 mmol) and molecular sieves (50 mg). The mixture was stirred at rt. for 3 days. The reaction was monitored by TLC and LC-

MS. Then aq. $NaHCO_3$ solution was added and stirred for 15 min. washed the DCM layer couple of times with aq. $NaHCO_3$, brine dried over $MgSO_4$ concentrated to afford crude product, which was then purified by column chromatography using DCM: MeOH: Et_3N 100: 2: 0.5 as a gradient to give 131 mg of colorless oil **43** (70% yield).; ^{1}H NMR (400 MHz, $CDCl_3$) δ 7.44 – 7.25 (m, 7H), 6.88 – 6.78 (m, 2H), 6.61 (s, 1H), 5.02 (s, 1H), 3.77 (d, *J* = 12.9 Hz, 3H), 2.71 (s, 1H), 2.23 (d, *J* = 5.3 Hz, 1H), 1.99 – 1.40 (m, 9H), 1.24 (dd, *J* = 27.1, 15.1 Hz, 3H), 1.04 (s, 3H), 0.90 (ddd, *J* = 12.5, 7.2, 5.8 Hz, 2H).; HPLC-MS (ESI) m/z calcd. for $C_{25}H_{33}N_2O_3$ $[M+H]^+$ = 409.24. Found: 409.22.

R_f = 0.26 in DCM : MeOH (10:1)

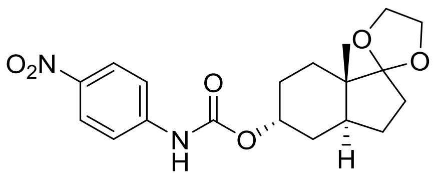

Chemical Formula: $C_{19}H_{24}N_2O_6$
Exact Mass: 376.1634
Molecular Weight: 376.4090

7a-Methyloctahydrospiro[indene-1,2'-[1,3]dioxolan]-5-yl (4-nitrophenyl)carbamate (40a): To the solution of alcohol **40** (536 mg, 2.52 mmol) in anhydrous THF (16 mL) were added *p*-nitrophenyl isocyanate (0.640 g , 3.78 mmol) and Et_3N (383 mg, 3.78 mmol). The mixture was stirred at rt. overnight. The reaction was monitored by TLC and LC-MS. The solvent was removed under vacuo and the crude product was purified through flash chromatography using pet-ether: acetone (6:1) as a gradient to afford 775 mg as brown oil **40a** (82% yield).; ^{1}H NMR (400 MHz, $CDCl_3$) δ 8.16 – 8.09 (m, 2H), 7.49 (d, *J* = 9.2 Hz, 2H), 6.88 (s, 1H), 4.92 – 4.75 (m, 1H), 3.93 – 3.74 (m, 4H), 1.93 – 1.65 (m, 9H), 1.56 (dd, *J* = 9.3, 4.6 Hz, 1H), 1.24 (dd, *J* = 9.4, 7.4 Hz, 1H), 0.91 (d, *J* = 6.2 Hz, 3H).; HPLC-MS (ESI) m/z calcd. for $C_{19}H_{25}N_2O_6$ $[M+H]^+$ = 377.16; Found: 377.08.

R_f = 0.36 pet-ether: acetone (5:1)

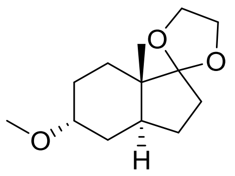

Chemical Formula: $C_{13}H_{22}O_3$
Exact Mass: 226.1569
Molecular Weight: 226.3160

5-Methoxy-7a-methyloctahydrospiro[indene-1,2'-[1,3]dioxolane] (44a): NaH (60% in oil, 29 mg, 0.73 mmol) was suspended in anhydrous THF (3 mL) under argon. The solution of alcohol **40** (104 mg, 0.5 mmol) in anhydrous THF (2 mL) was added to the suspension at 0 °C. The mixture was heated to 40 °C and stirred for 0.5 hour. After cooling to 0 °C, iodomethane was added to the mixture and stirred at rt. for overnight. The reaction was monitored by TLC and GC-MS. The reaction was quenched with water and $Na_2S_2O_3$ solution, the aqueous layer was extracted couple of times with EtOAc and the combined layers were washed with brine, dried over $MgSO_4$, concentrated in vacuo. The product was purified through flash chromatography using pet-ether: EtOAc (20:1) to give the colorless liquid **(44a)** 46 mg (40% yield).; ^{1}H NMR (400 MHz, $CDCl_3$) δ 3.91 – 3.85 (m, 4H), 3.31 – 3.26 (m, 4H), 1.83 – 1.54 (m, 10H), 1.24 – 1.11 (m, 1H), 0.93 (s, 3H).; HPLC-MS (ESI) m/z calcd. for $C_{13}H_{22}O_3$ $[M]^+$ = 226.15. Found: 226.21.

R_f = 0.49 in pet-ether: EtOAc (8:1)

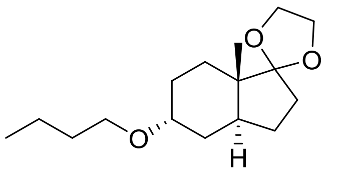

Chemical Formula: $C_{16}H_{28}O_3$
Exact Mass: 268.2038
Molecular Weight: 268.3970

5-Butoxy-7a-methyloctahydrospiro[indene-1,2'-[1,3]dioxolane] (44b): NaH (60% in oil, 166 mg, 4.14 mmol) was suspended in anhydrous THF (2 mL) under argon. The solution of alcohol **40** (587 mg, 2.76 mmol) in anhydrous THF (2 mL) was added to the suspension at 0 °C. The mixture was heated to 40 °C and stirred for 0.5 hour. After cooling to rt., 1-iodobutane was added to the reaction mixture and stirred at rt. for 18 h. The reaction was monitored by TLC and GC-MS. The reaction was quenched with water and $Na_2S_2O_3$ solution, the aqueous layer was extracted couple of times with EtOAc and the combined layers were washed with brine, dried over $MgSO_4$, concentrated in vacuo. The product was purified through flash chromatography using pet-ether: acetone (100:2) to give the colorless liquid **(44b)** 387 mg (52% yield).; GC-MS (ESI) m/z calcd. for $C_{16}H_{28}O_3$ $[M]^+$ = 268.20; Found: 268.15.

R_f = 0.52 in pet-ether: acetone (20:1)

Copy of ^{1}H and ^{13}C NMR spectra's:

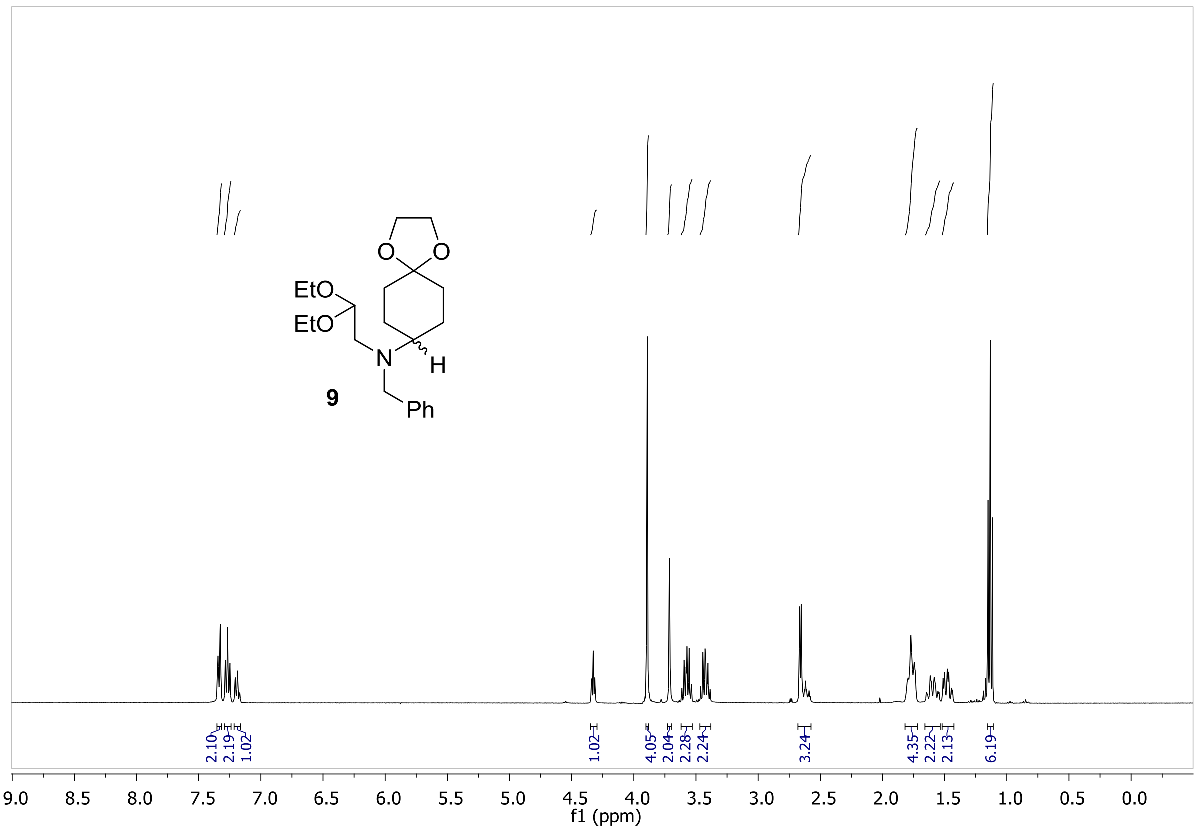

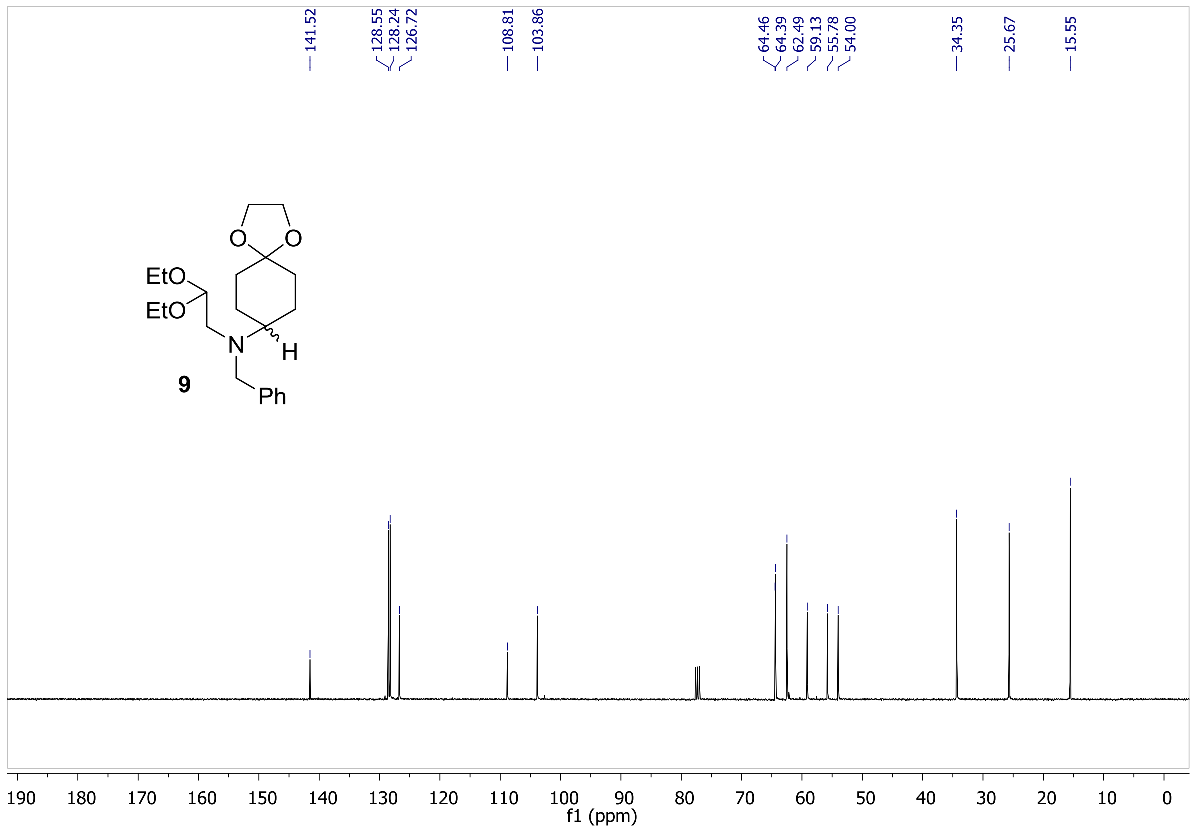

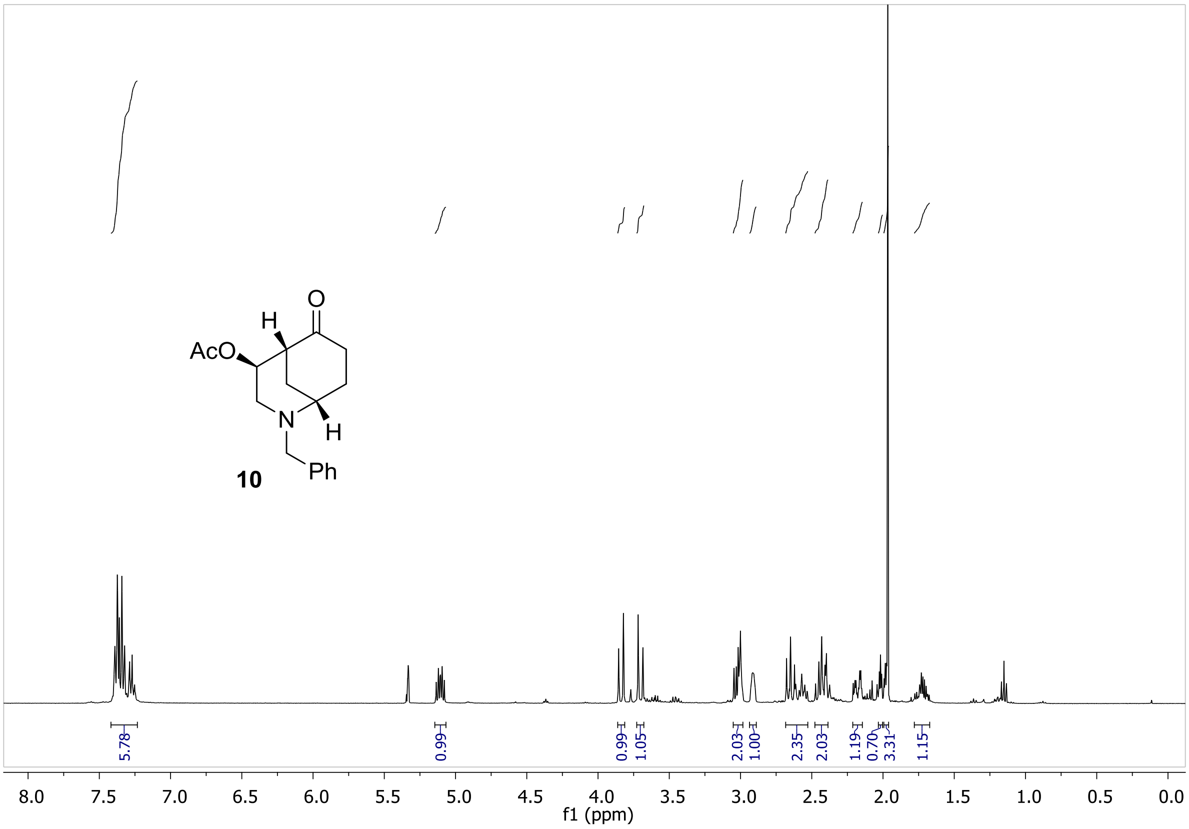
AcO
H
O
N
H
Ph
10
5.78
0.99
0.99
1.05
2.03
1.00
2.35
2.03
1.19
0.70
3.31
1.15
8.0 7.5 7.0 6.5 6.0 5.5 5.0 4.5 4.0 3.5 3.0 2.5 2.0 1.5 1.0 0.5 0.0
f1 (ppm)

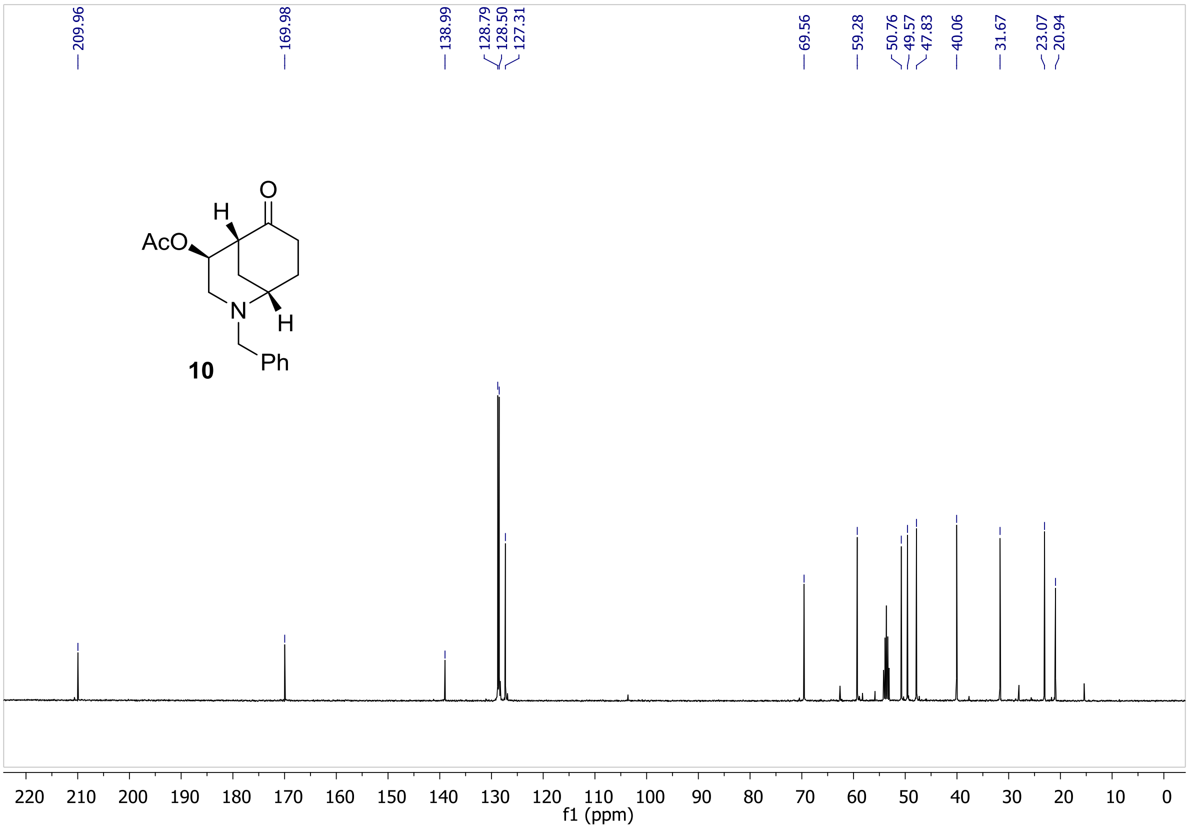
209.96
169.98
138.99
128.79
128.50
127.31
69.56
59.28
50.76
49.57
47.83
40.06
31.67
23.07
20.94
AcO
H
O
N
H
Ph
10
220 210 200 190 180 170 160 150 140 130 120 110 100 90 80 70 60 50 40 30 20 10 0
f1 (ppm)

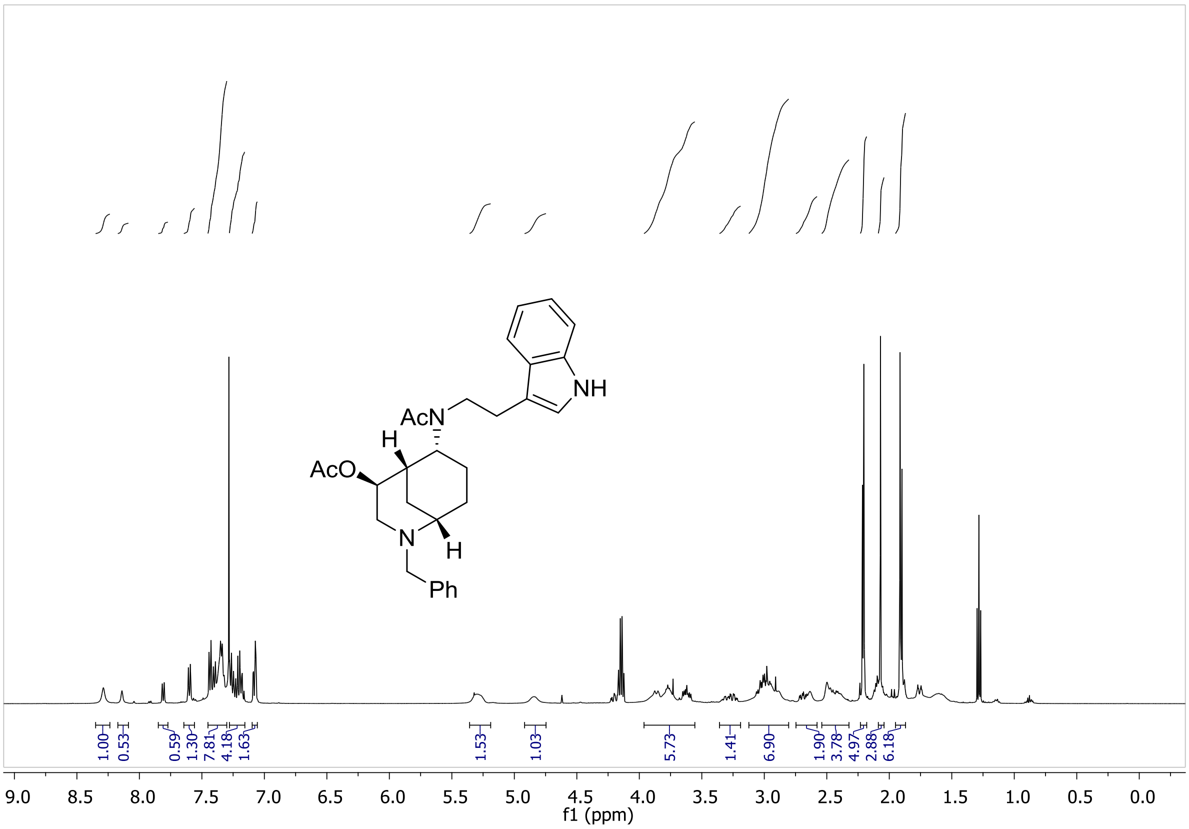
AcN
NH
H
AcO
N
H
Ph
1.00
0.53
0.59
1.30
7.81
4.18
1.63
1.53
1.03
5.73
1.41
6.90
1.90
3.78
4.97
2.88
6.18
9.0 8.5 8.0 7.5 7.0 6.5 6.0 5.5 5.0 4.5 4.0 3.5 3.0 2.5 2.0 1.5 1.0 0.5 0.0
f1 (ppm)

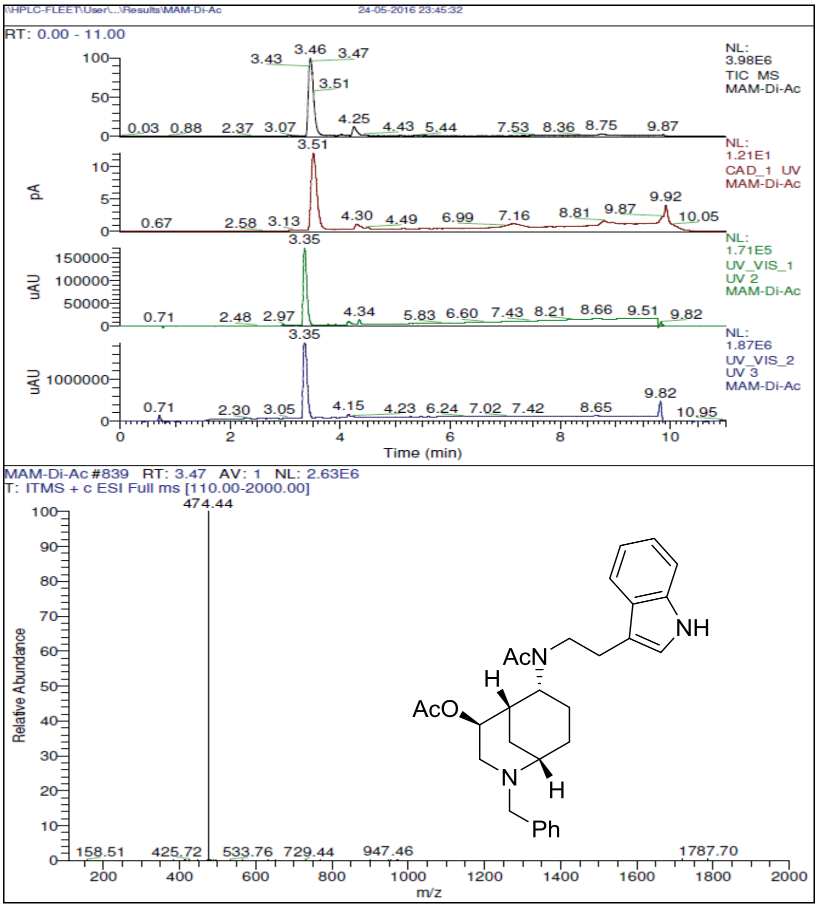
\HPLC-FLEET\User\...\Results\MAM-Di-Ac
24-05-2016 23:45:32
RT: 0.00 - 11.00
NL: 3.98E6 TIC MS MAM-Di-Ac
NL: 1.21E1 CAD_1 UV MAM-Di-Ac
NL: 1.71E5 UV_VIS_1 UV 2 MAM-Di-Ac
NL: 1.87E6 UV_VIS_2 UV 3 MAM-Di-Ac
pA
uAU
Time (min)
MAM-Di-Ac #839 RT: 3.47 AV: 1 NL: 2.63E6
T: ITMS + c ESI Full ms [110.00-2000.00]
474.44
158.51
425.72
533.76
729.44
947.46
1787.70
Relative Abundance
m/z
AcN
NH
H
AcO
N
H
Ph

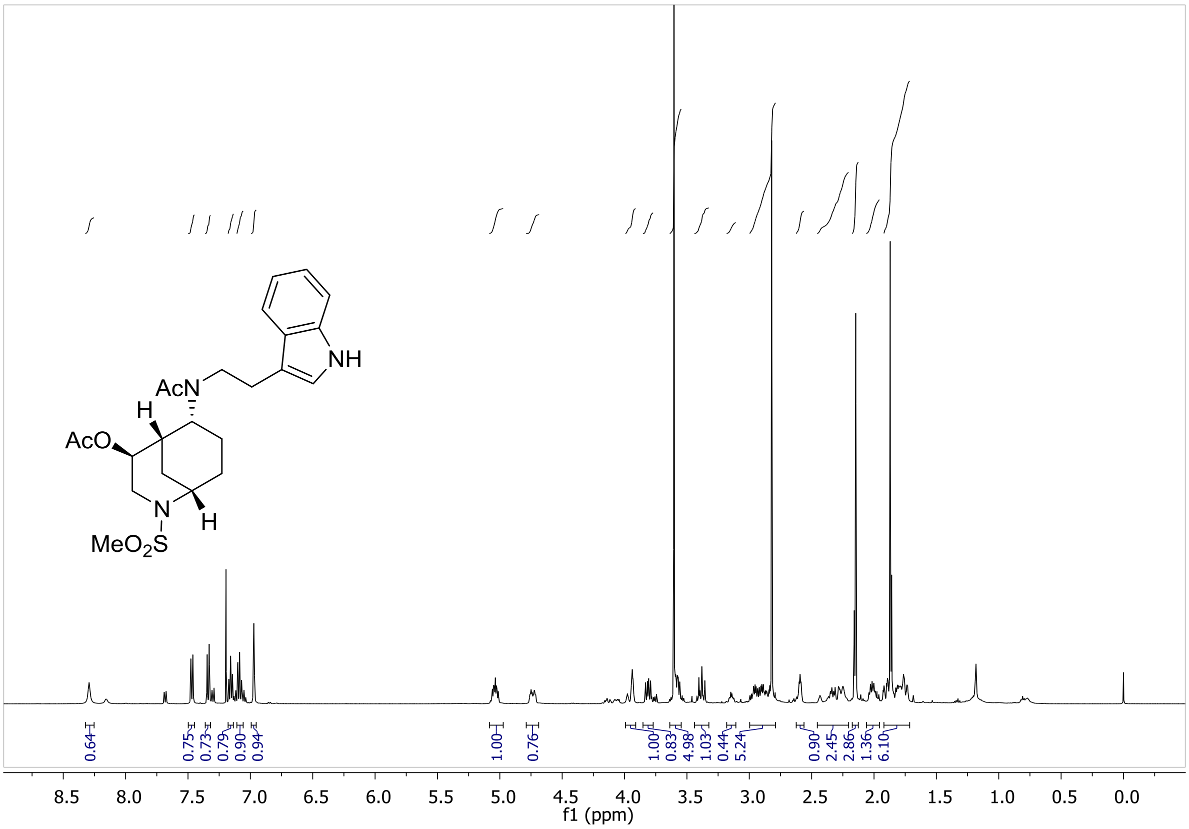
AcN
NH
H
AcO
N
H
MeO2S
0.64
0.75
0.73
0.79
0.90
0.94
1.00
0.76
1.00
0.83
4.98
1.03
0.44
5.24
0.90
2.45
2.86
1.36
6.10
8.5
8.0
7.5
7.0
6.5
6.0
5.5
5.0
4.5
4.0
3.5
3.0
2.5
2.0
1.5
1.0
0.5
0.0
f1 (ppm)

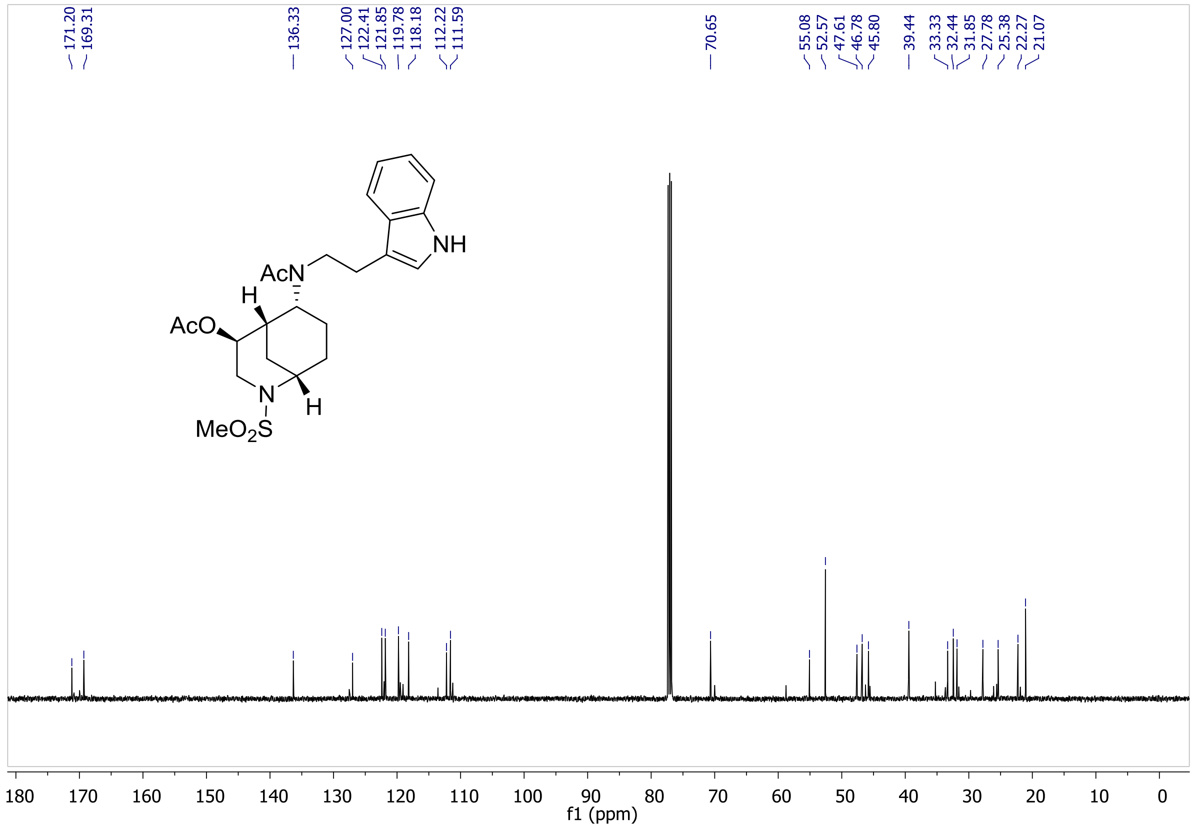
171.20
169.31
136.33
127.00
122.41
121.85
119.78
118.18
112.22
111.59
70.65
55.08
52.57
47.61
46.78
45.80
39.44
33.33
32.44
31.85
27.78
25.38
22.27
21.07
AcN
NH
H
AcO
N
H
MeO2S
180
170
160
150
140
130
120
110
100
90
80
70
60
50
40
30
20
10
0
f1 (ppm)

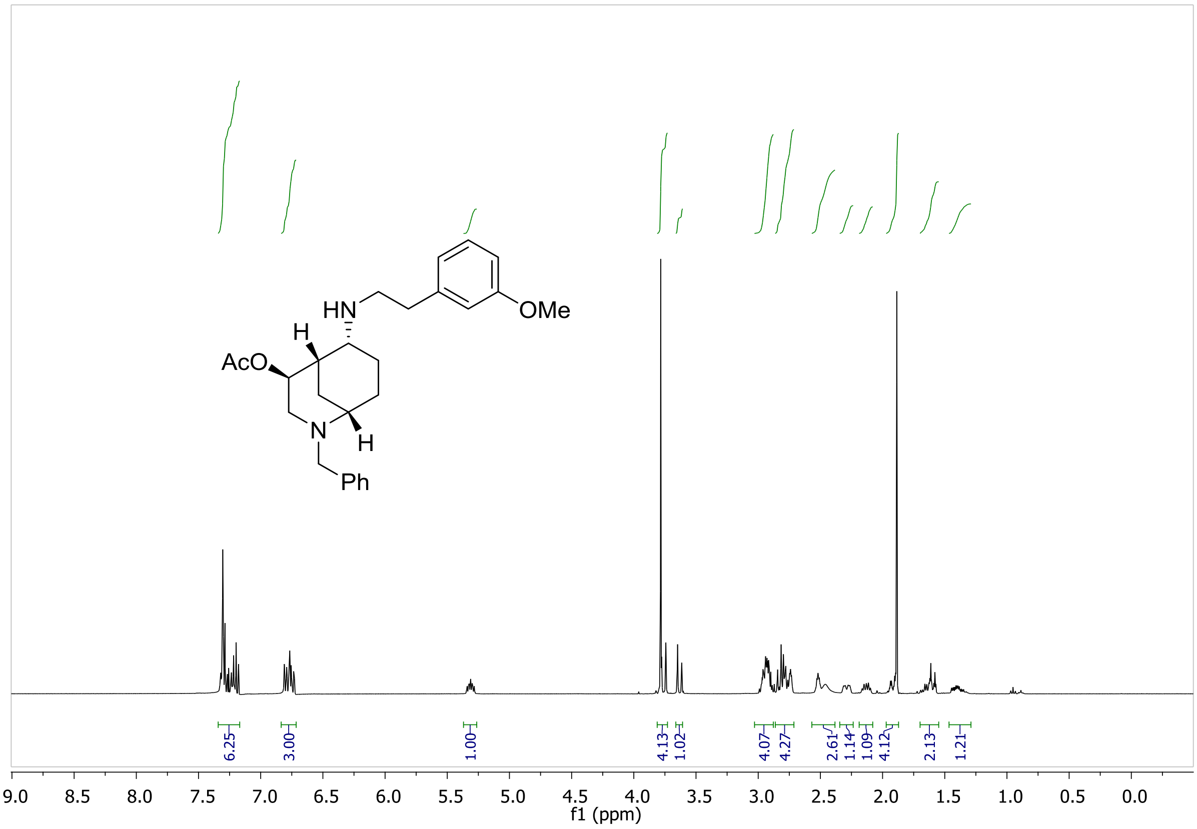
HN
OMe
AcO
H
N
H
Ph
6.25
3.00
1.00
4.13
1.02
4.07
4.27
2.61
1.14
1.09
4.12
2.13
1.21
9.0
8.5
8.0
7.5
7.0
6.5
6.0
5.5
5.0
4.5
4.0
3.5
3.0
2.5
2.0
1.5
1.0
0.5
0.0
f1 (ppm)

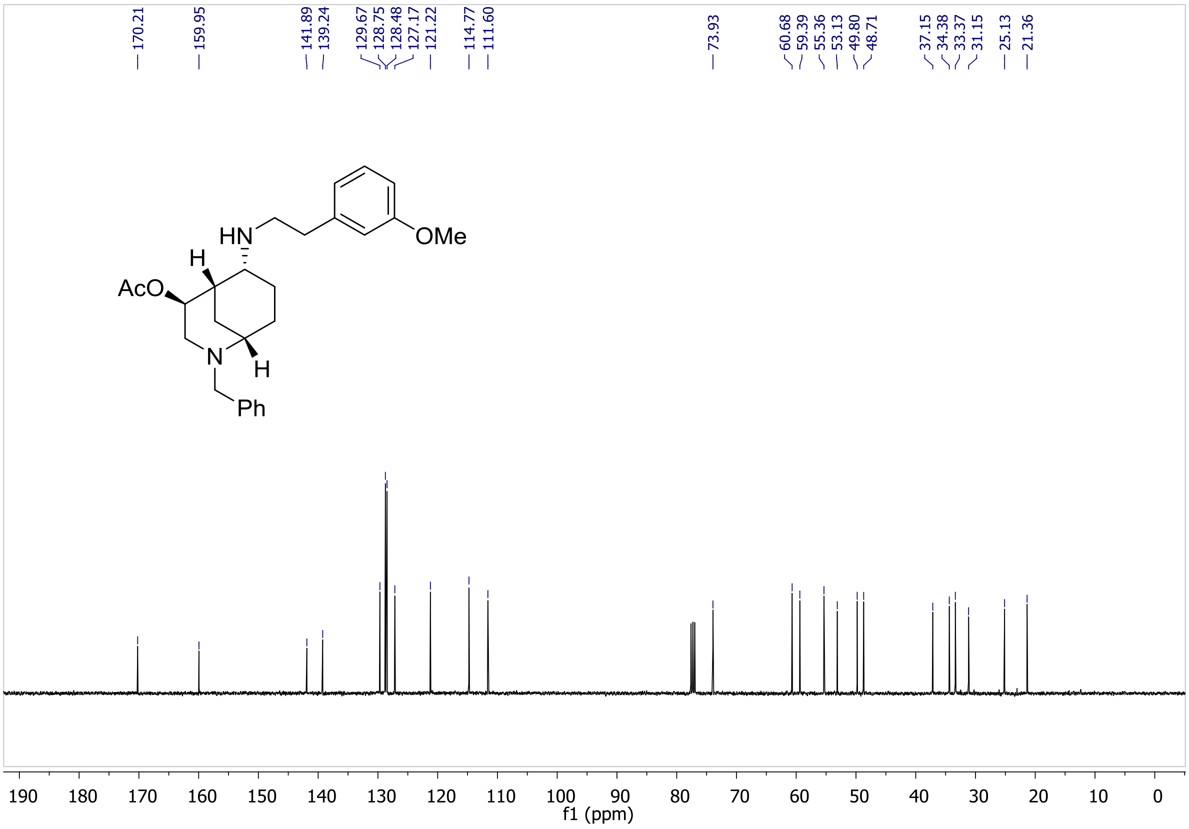
170.21
159.95
141.89
139.24
129.67
128.75
128.48
127.17
121.22
114.77
111.60
73.93
60.68
59.39
55.36
53.13
49.80
48.71
37.15
34.38
33.37
31.15
25.13
21.36
HN
OMe
AcO
H
N
H
Ph
190
180
170
160
150
140
130
120
110
100
90
80
70
60
50
40
30
20
10
0
f1 (ppm)

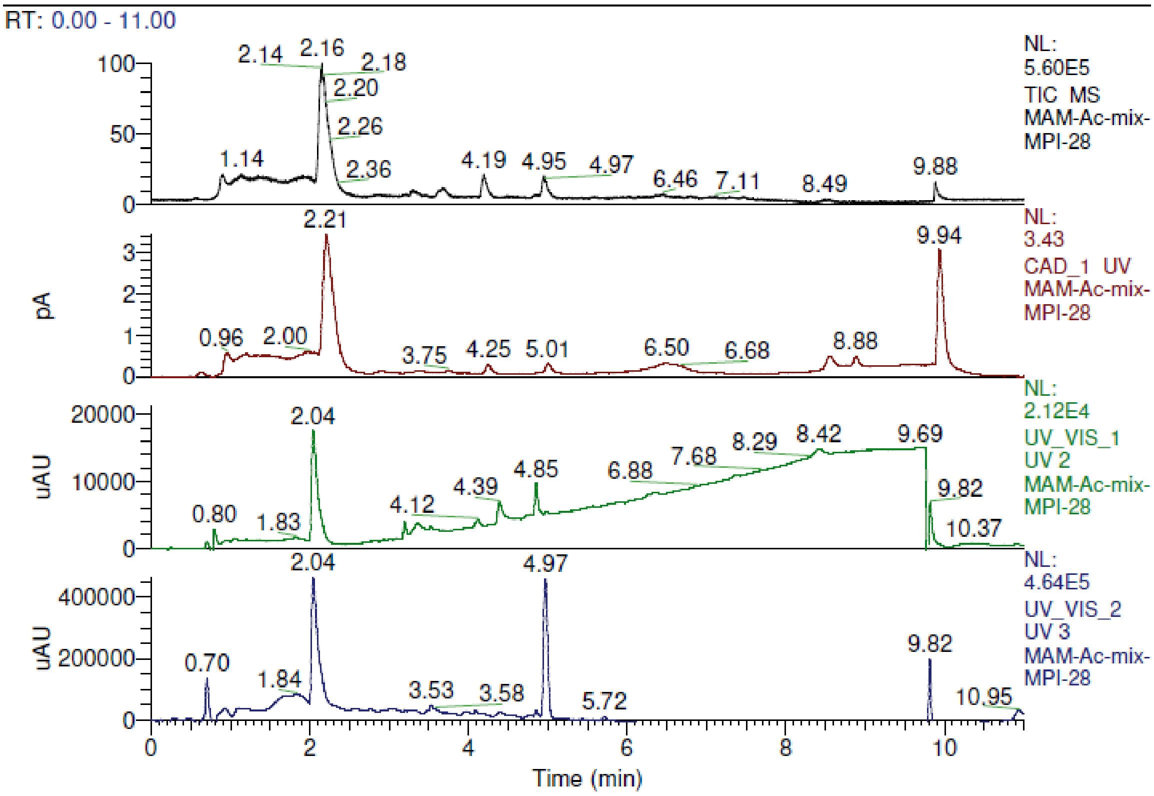

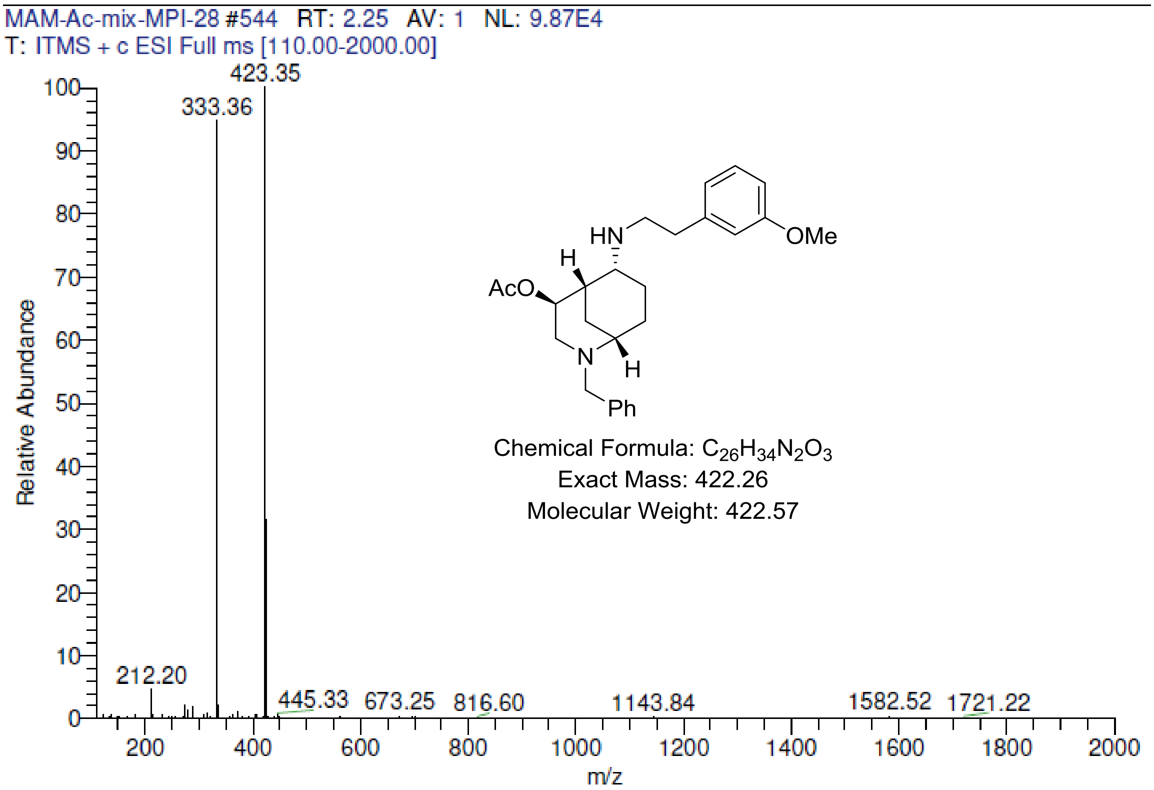

Chemical Formula: $C_{26}H_{34}N_2O_3$
Exact Mass: 422.26
Molecular Weight: 422.57

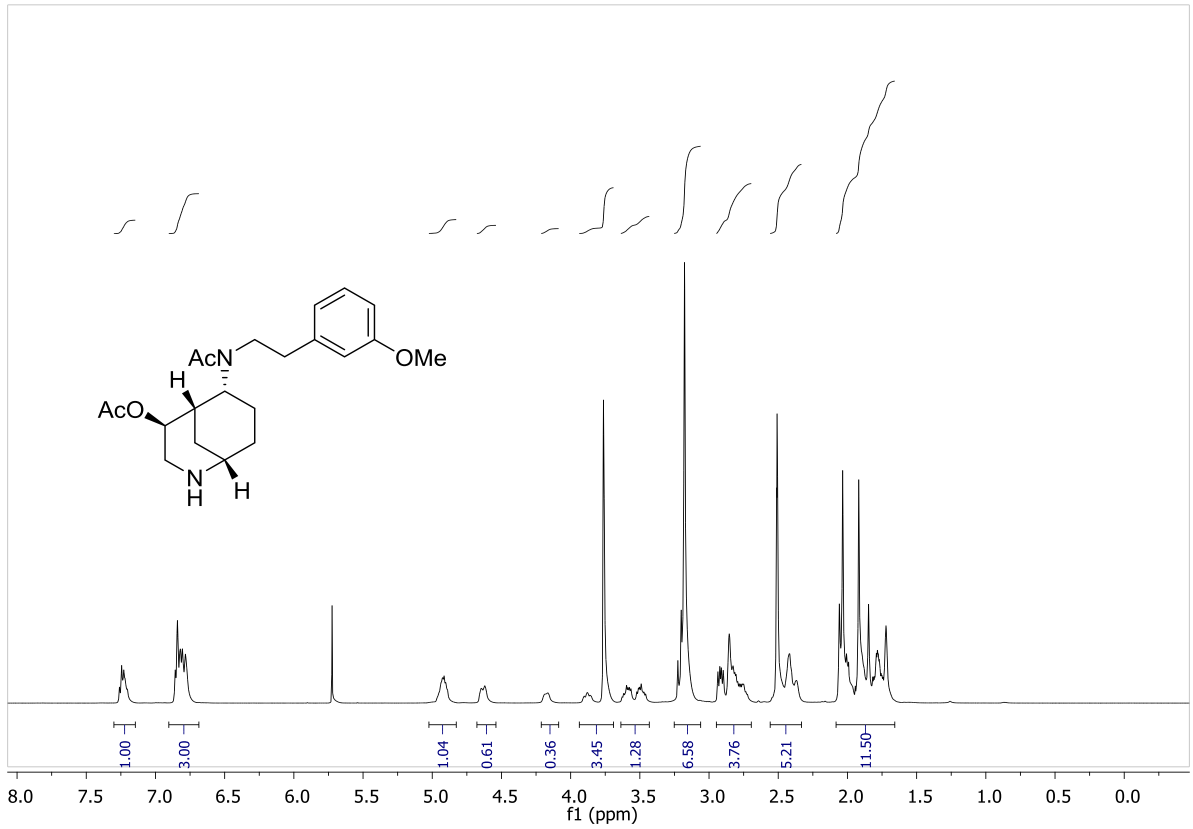
AcN
OMe
H
AcO
N
H
H
1.00
3.00
1.04
0.61
0.36
3.45
1.28
6.58
3.76
5.21
11.50
8.0 7.5 7.0 6.5 6.0 5.5 5.0 4.5 4.0 3.5 3.0 2.5 2.0 1.5 1.0 0.5 0.0
f1 (ppm)

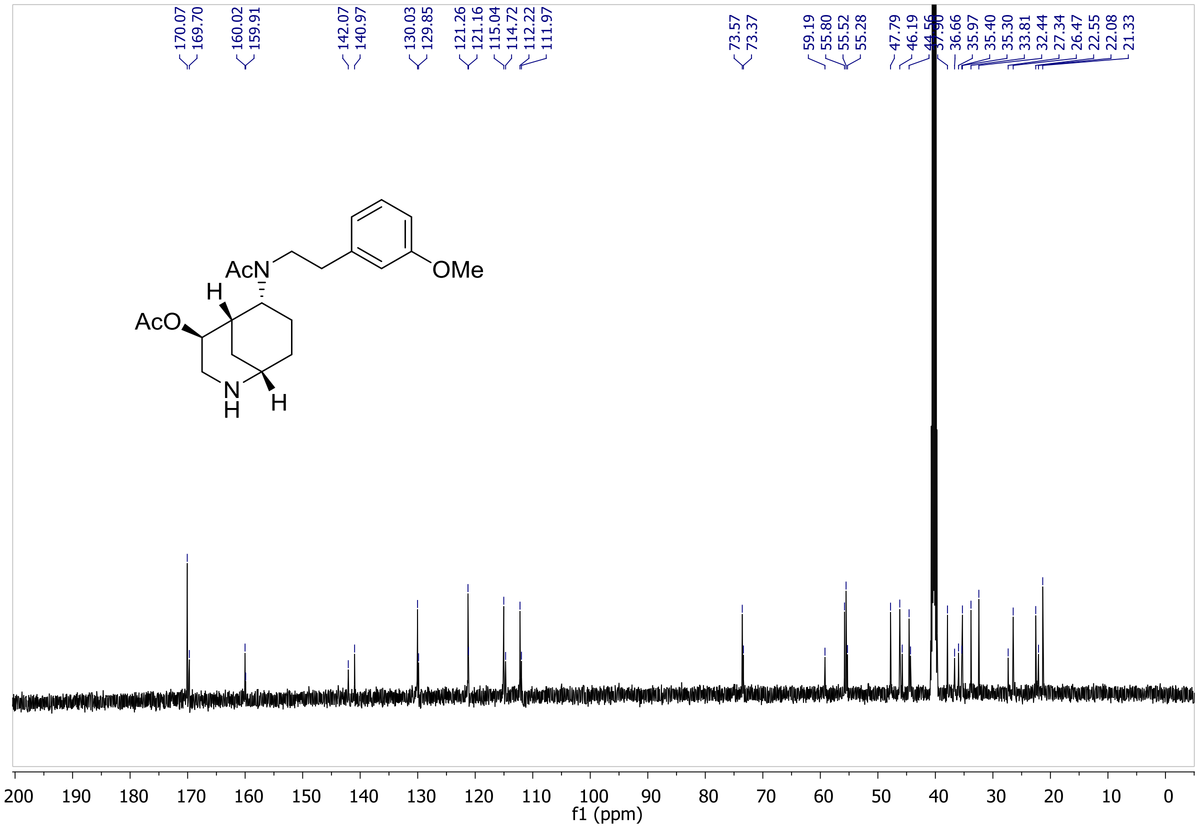
170.07
169.70
160.02
159.91
142.07
140.97
130.03
129.85
121.26
121.16
115.04
114.72
112.22
111.97
73.57
73.37
59.19
55.80
55.52
55.28
47.79
46.19
44.56
37.90
36.66
35.97
35.40
35.30
33.81
32.44
27.34
26.47
22.55
22.08
21.33
AcN
OMe
H
AcO
N
H
H
200 190 180 170 160 150 140 130 120 110 100 90 80 70 60 50 40 30 20 10 0
f1 (ppm)

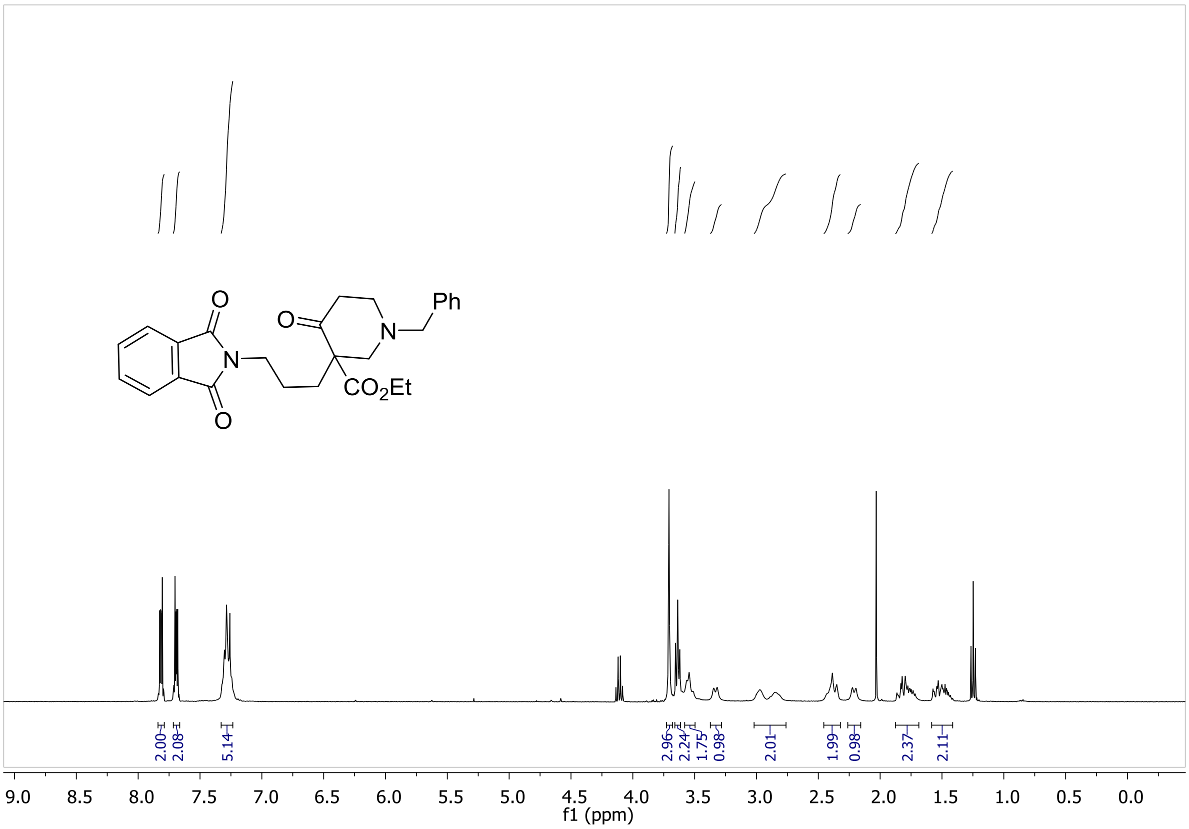

CO2Et
Ph
2.00
2.08
5.14
2.96
2.24
1.75
0.98
2.01
1.99
0.98
2.37
2.11
f1 (ppm)

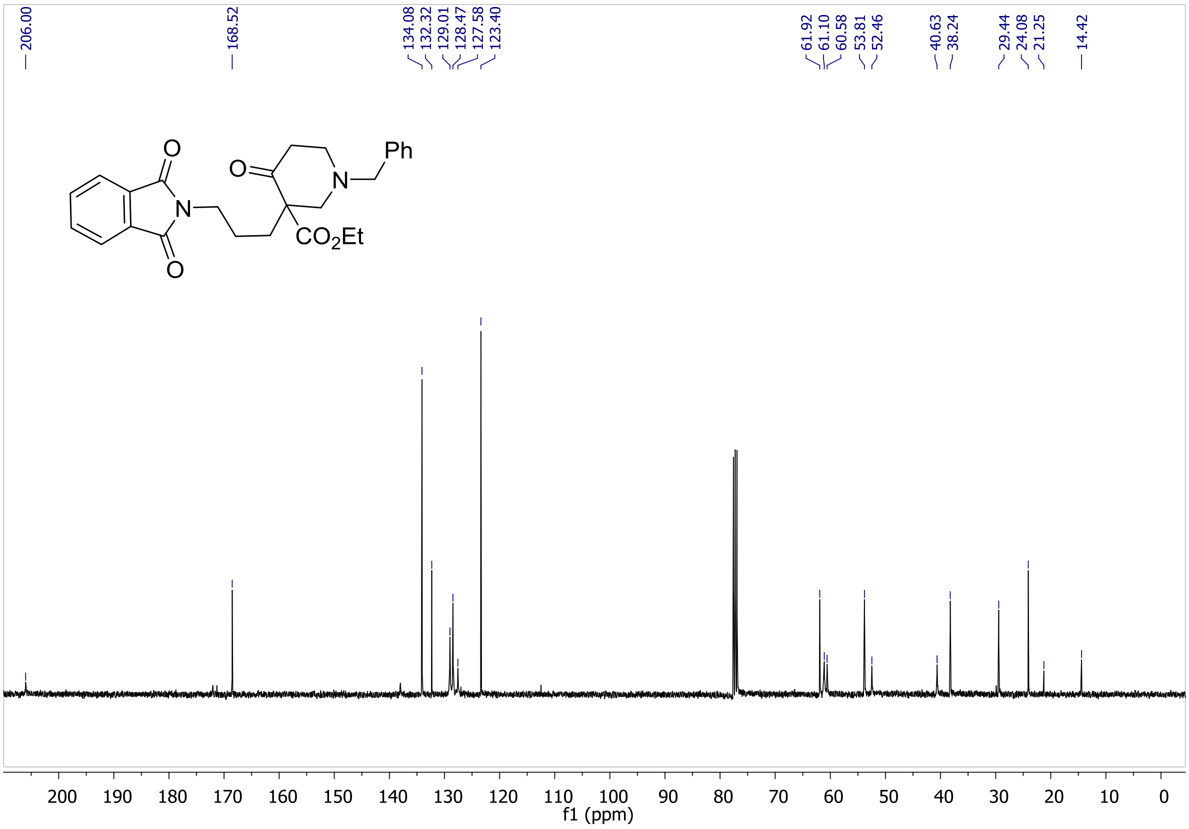

206.00
168.52
134.08
132.32
129.01
128.47
127.58
123.40
61.92
61.10
60.58
53.81
52.46
40.63
38.24
29.44
24.08
21.25
14.42
CO2Et
Ph
f1 (ppm)

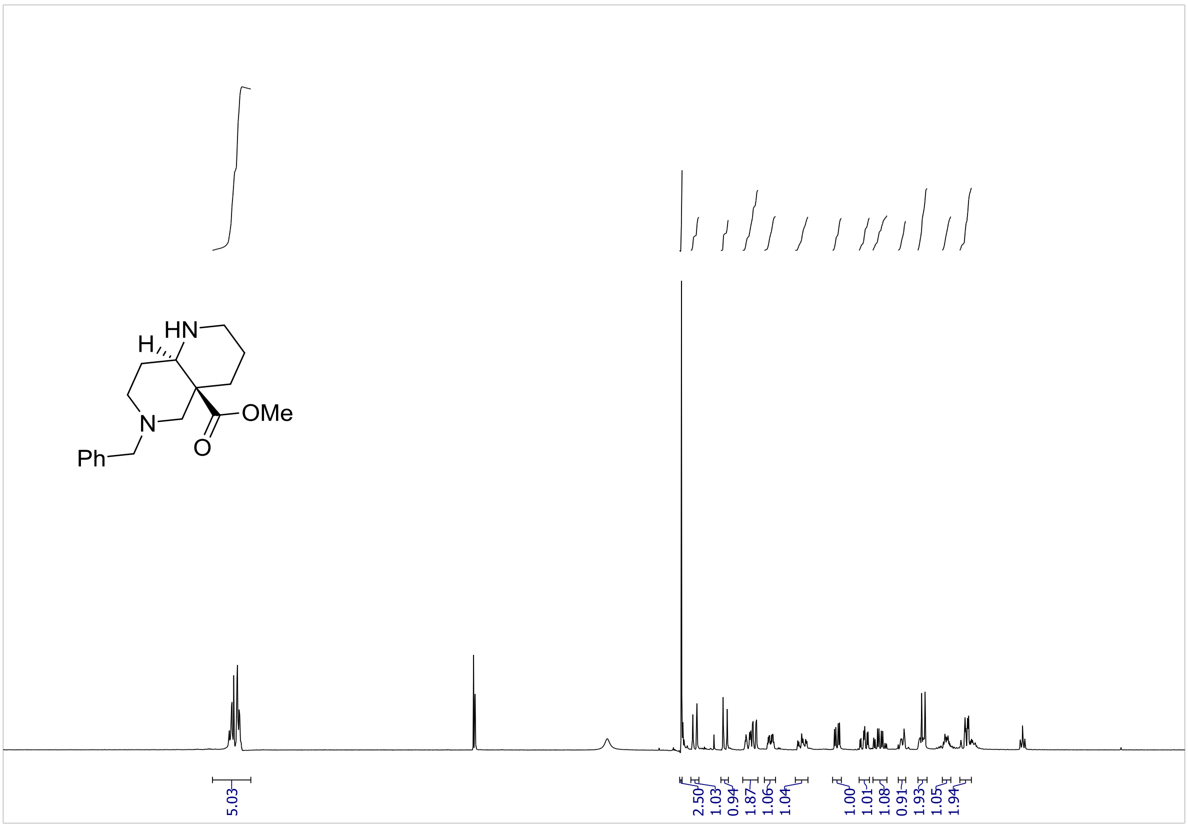
HN
H
N
OMe
O
Ph
5.03
2.50
1.03
0.94
1.87
1.06
1.04
1.00
1.01
1.08
0.91
1.93
1.05
1.94

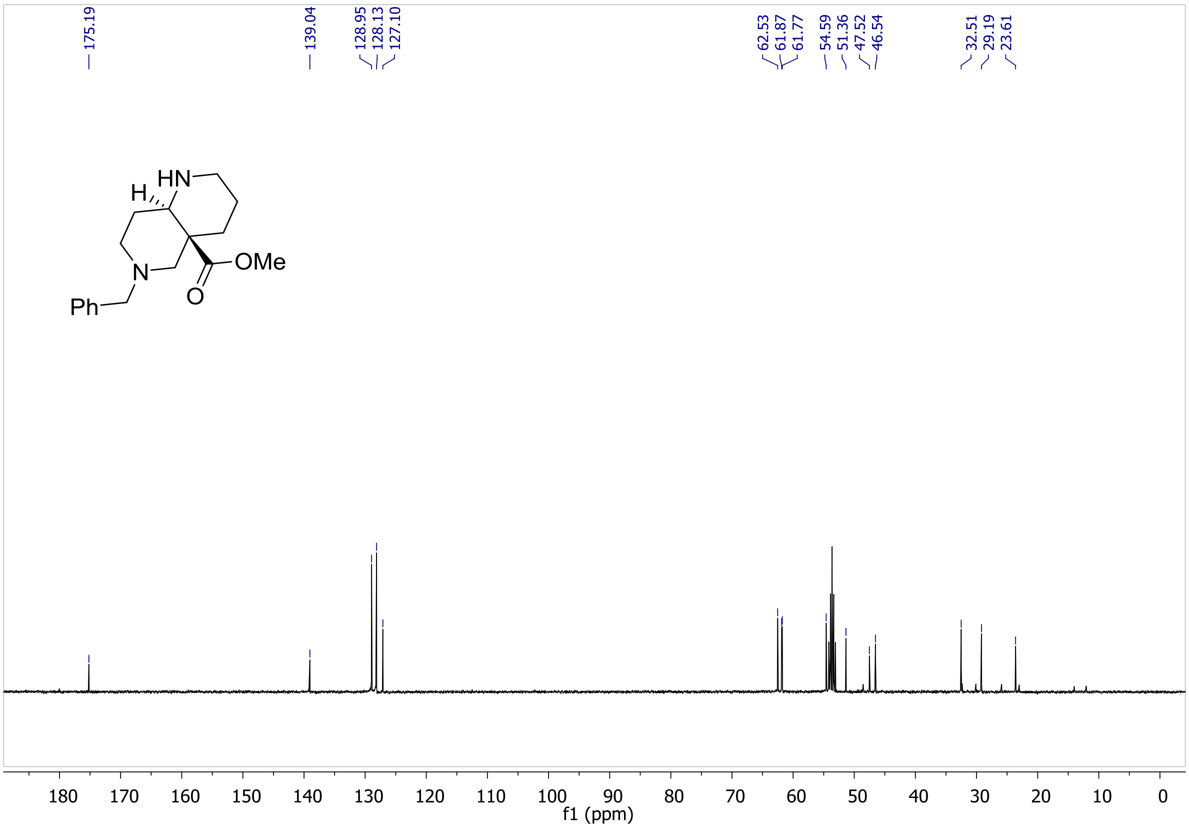
175.19
139.04
128.95
128.13
127.10
62.53
61.87
61.77
54.59
51.36
47.52
46.54
32.51
29.19
23.61
HN
H
N
OMe
O
Ph
180 170 160 150 140 130 120 110 100 90 80 70 60 50 40 30 20 10 0
f1 (ppm)

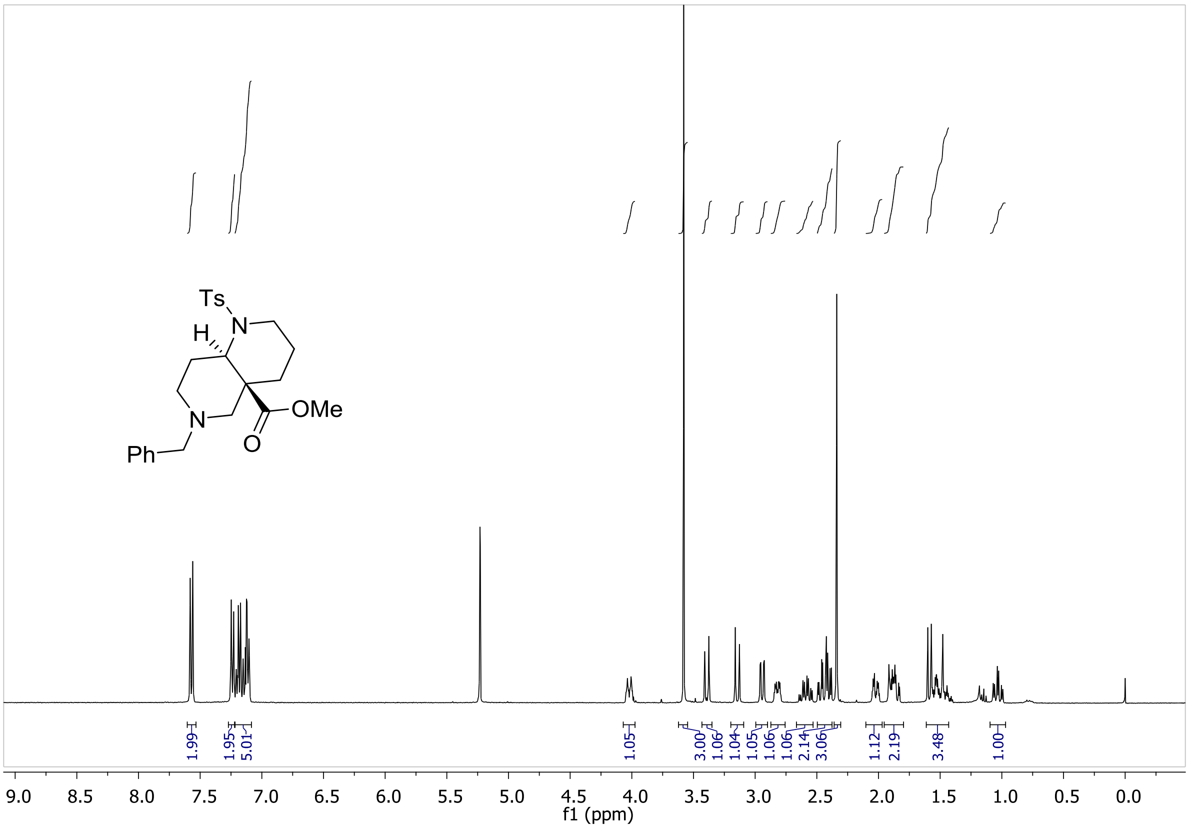
Ts
H
N
N
OMe
O
Ph
1.99
1.95
5.01
1.05
3.00
1.06
1.04
1.05
1.06
1.06
2.14
3.06
1.12
2.19
3.48
1.00
9.0 8.5 8.0 7.5 7.0 6.5 6.0 5.5 5.0 4.5 4.0 3.5 3.0 2.5 2.0 1.5 1.0 0.5 0.0
f1 (ppm)

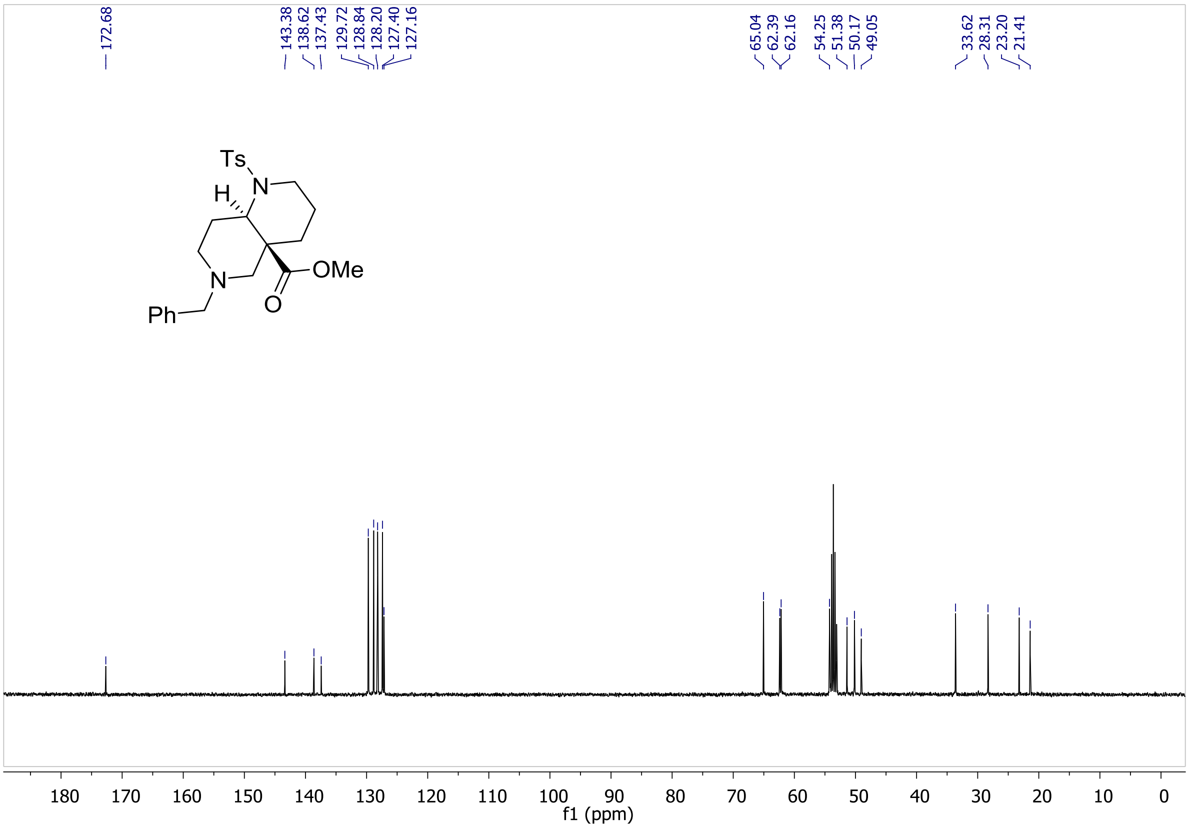
172.68
143.38
138.62
137.43
129.72
128.84
128.20
127.40
127.16
65.04
62.39
62.16
54.25
51.38
50.17
49.05
33.62
28.31
23.20
21.41
Ts
H
N
N
OMe
O
Ph
180 170 160 150 140 130 120 110 100 90 80 70 60 50 40 30 20 10 0
f1 (ppm)

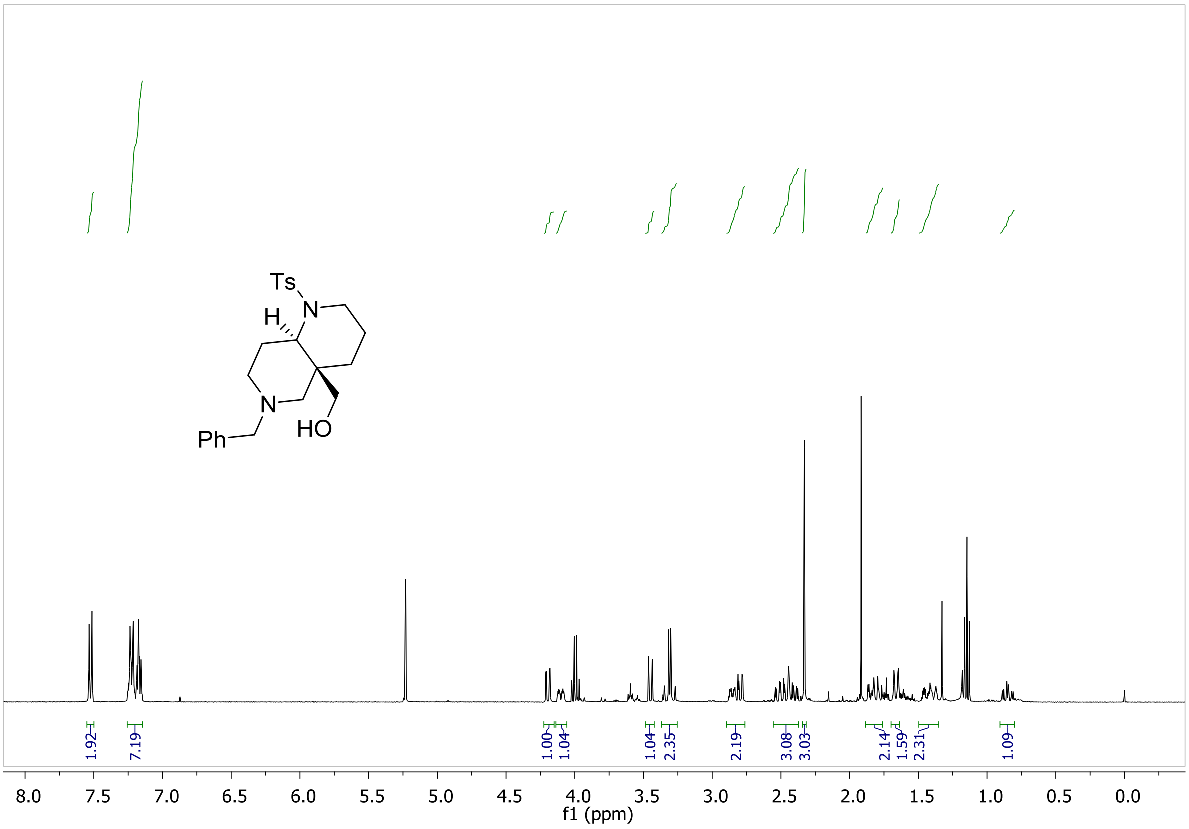
Ts
N
H
N
Ph
HO
1.92
7.19
1.00
1.04
1.04
2.35
2.19
3.08
3.03
2.14
1.59
2.31
1.09
8.0 7.5 7.0 6.5 6.0 5.5 5.0 4.5 4.0 3.5 3.0 2.5 2.0 1.5 1.0 0.5 0.0
f1 (ppm)

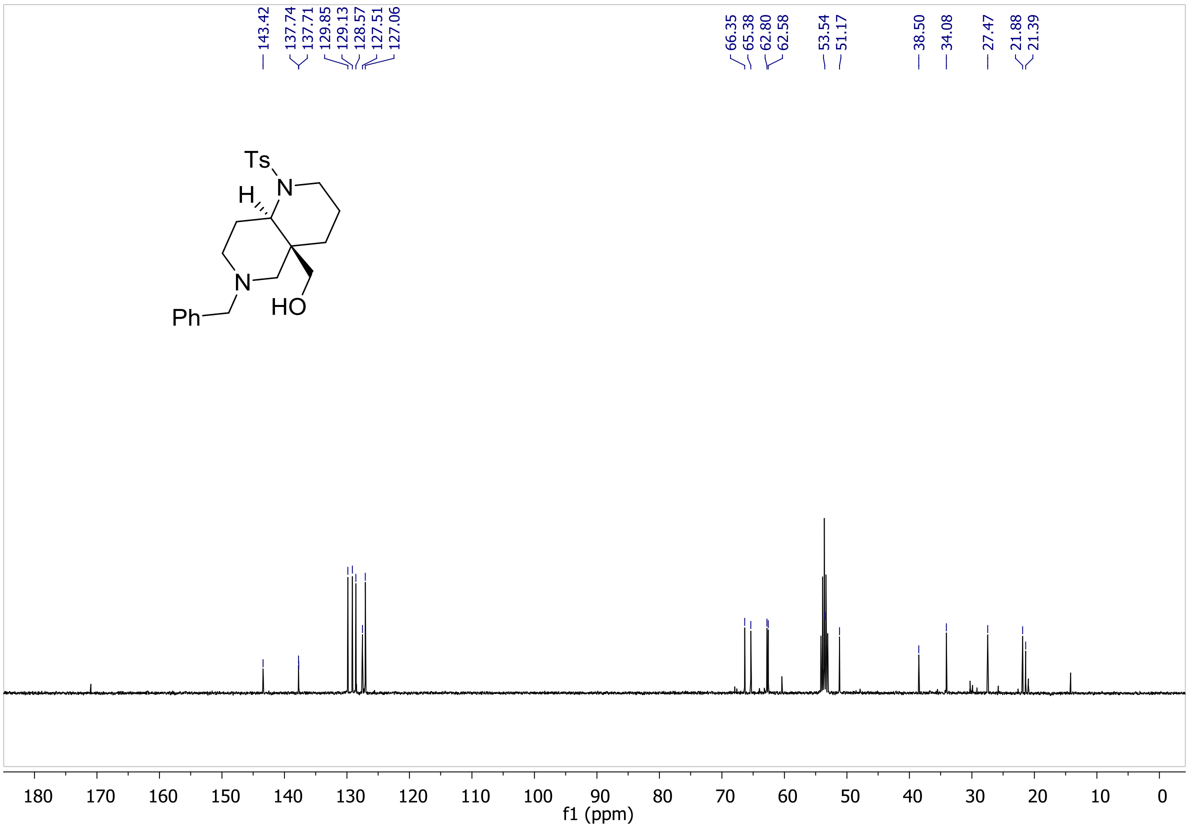
143.42
137.74
137.71
129.85
129.13
128.57
127.51
127.06
66.35
65.38
62.80
62.58
53.54
51.17
38.50
34.08
27.47
21.88
21.39
Ts
N
H
N
Ph
HO
180 170 160 150 140 130 120 110 100 90 80 70 60 50 40 30 20 10 0
f1 (ppm)

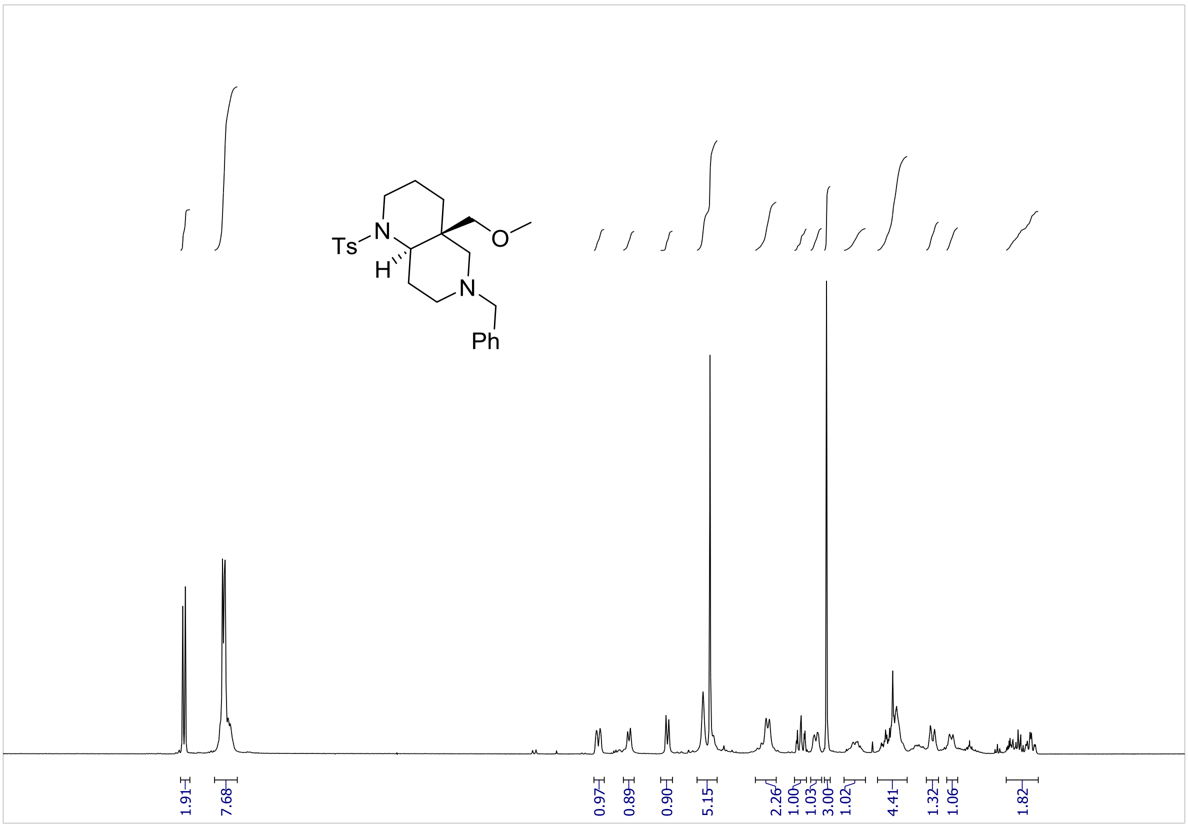
Ts
N
H
O
N
Ph
1.91
7.68
0.97
0.89
0.90
5.15
2.26
1.00
1.03
3.00
1.02
4.41
1.32
1.06
1.82

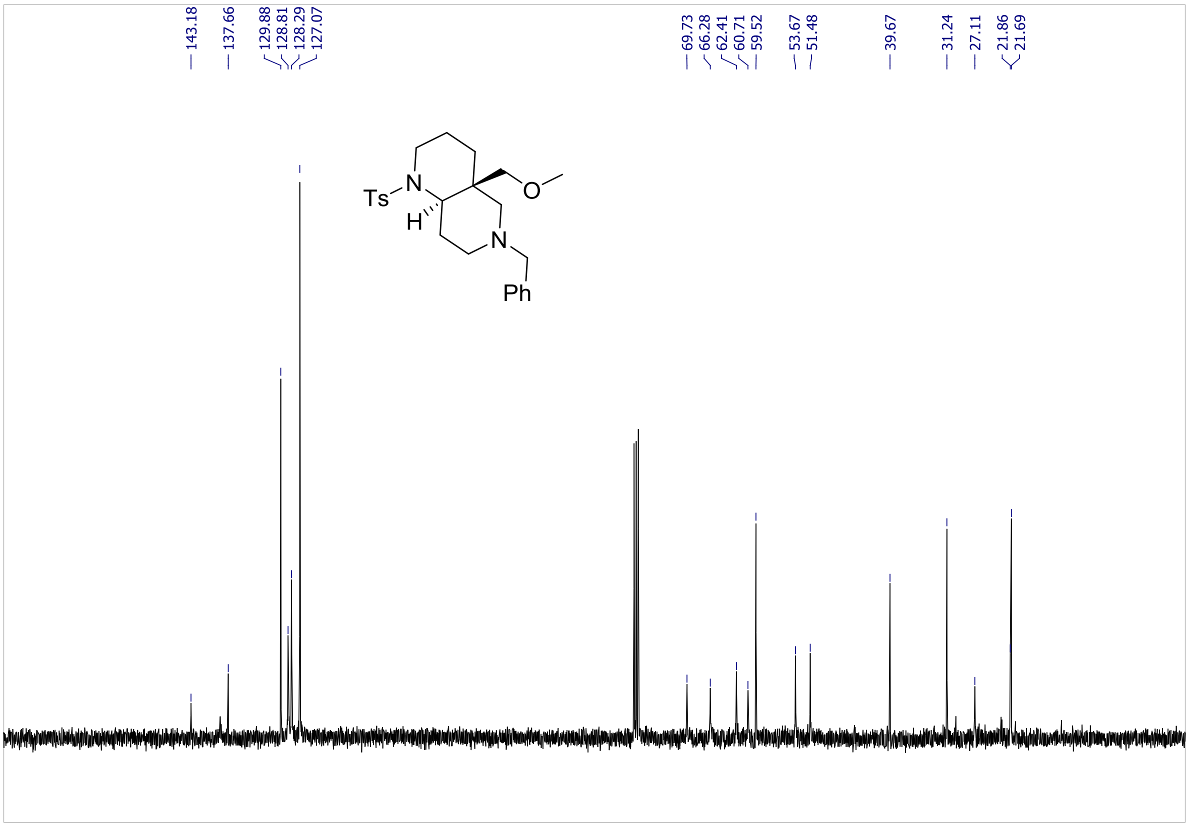
143.18
137.66
129.88
128.81
128.29
127.07
69.73
66.28
62.41
60.71
59.52
53.67
51.48
39.67
31.24
27.11
21.86
21.69
Ts
N
H
O
N
Ph

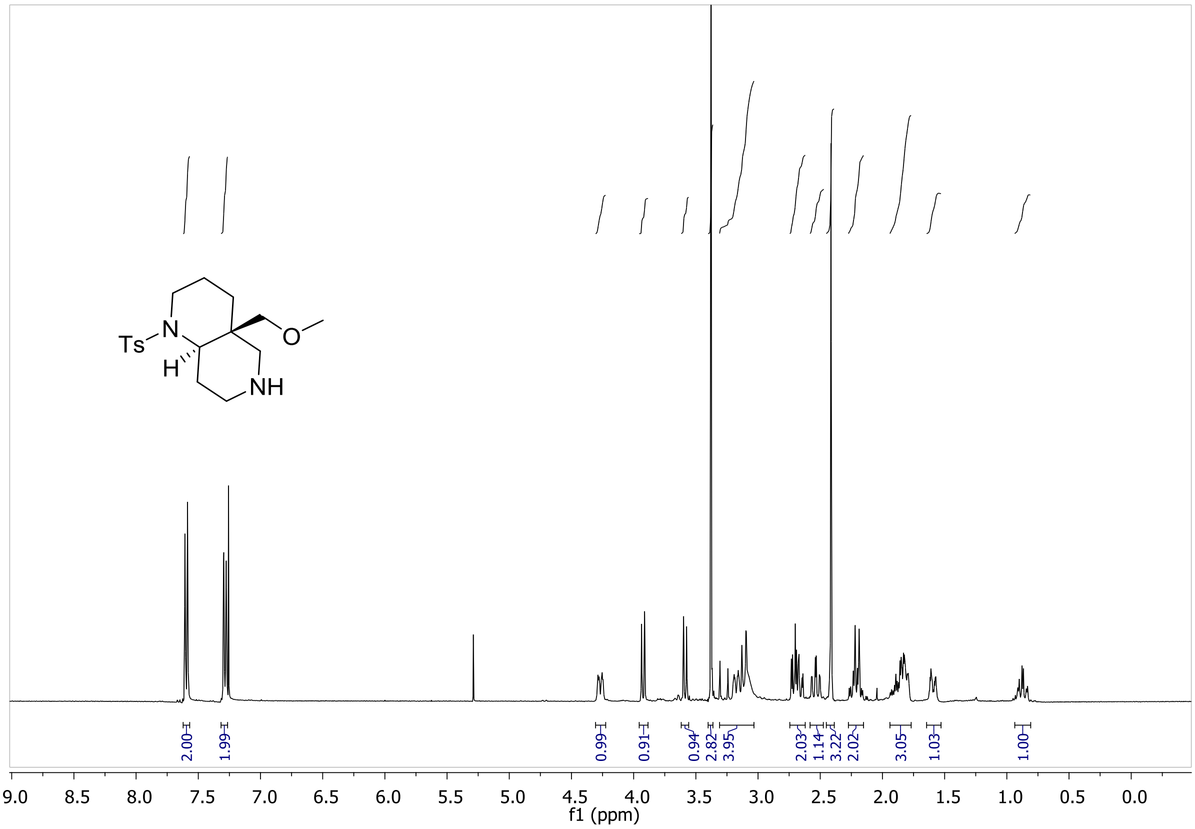
Ts
N
H
O
NH
2.00
1.99
0.99
0.91
0.94
2.82
3.95
2.03
1.14
3.22
2.02
3.05
1.03
1.00
9.0 8.5 8.0 7.5 7.0 6.5 6.0 5.5 5.0 4.5 4.0 3.5 3.0 2.5 2.0 1.5 1.0 0.5 0.0
f1 (ppm)

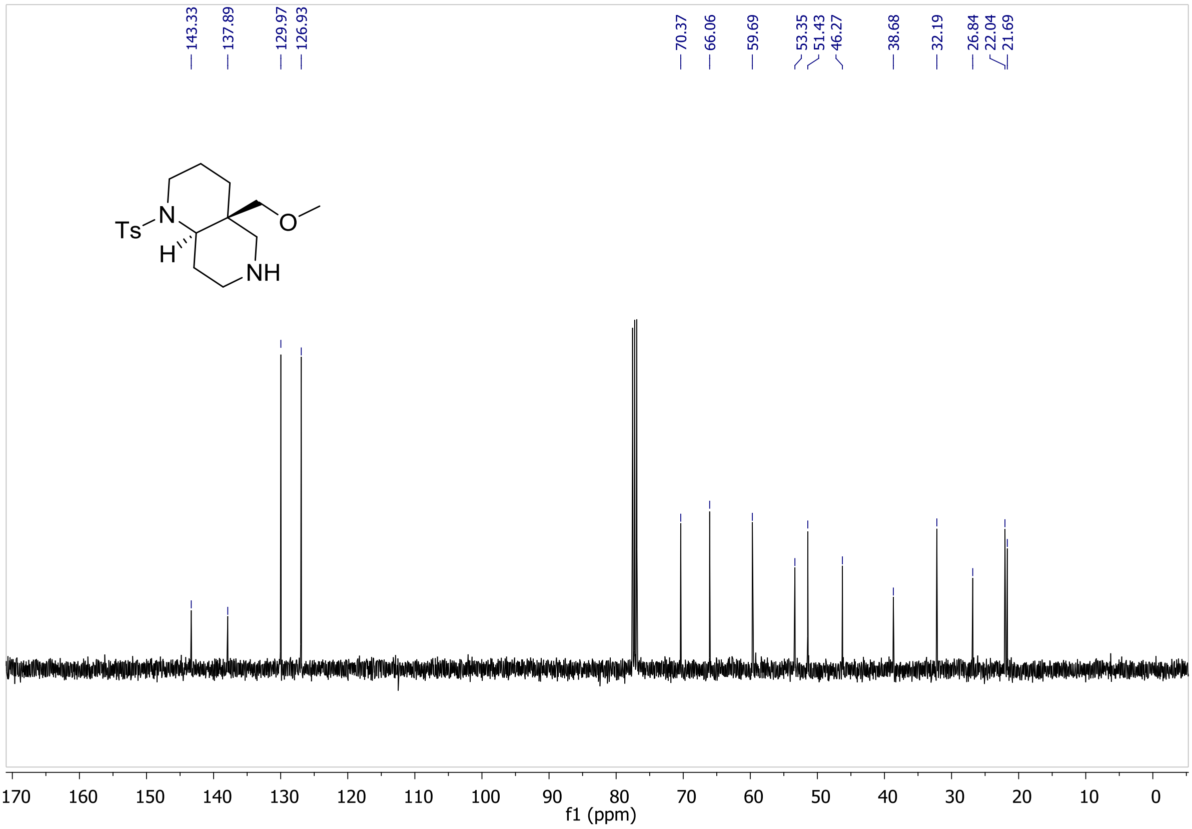
143.33
137.89
129.97
126.93
70.37
66.06
59.69
53.35
51.43
46.27
38.68
32.19
26.84
22.04
21.69
Ts
N
H
O
NH
170 160 150 140 130 120 110 100 90 80 70 60 50 40 30 20 10 0
f1 (ppm)

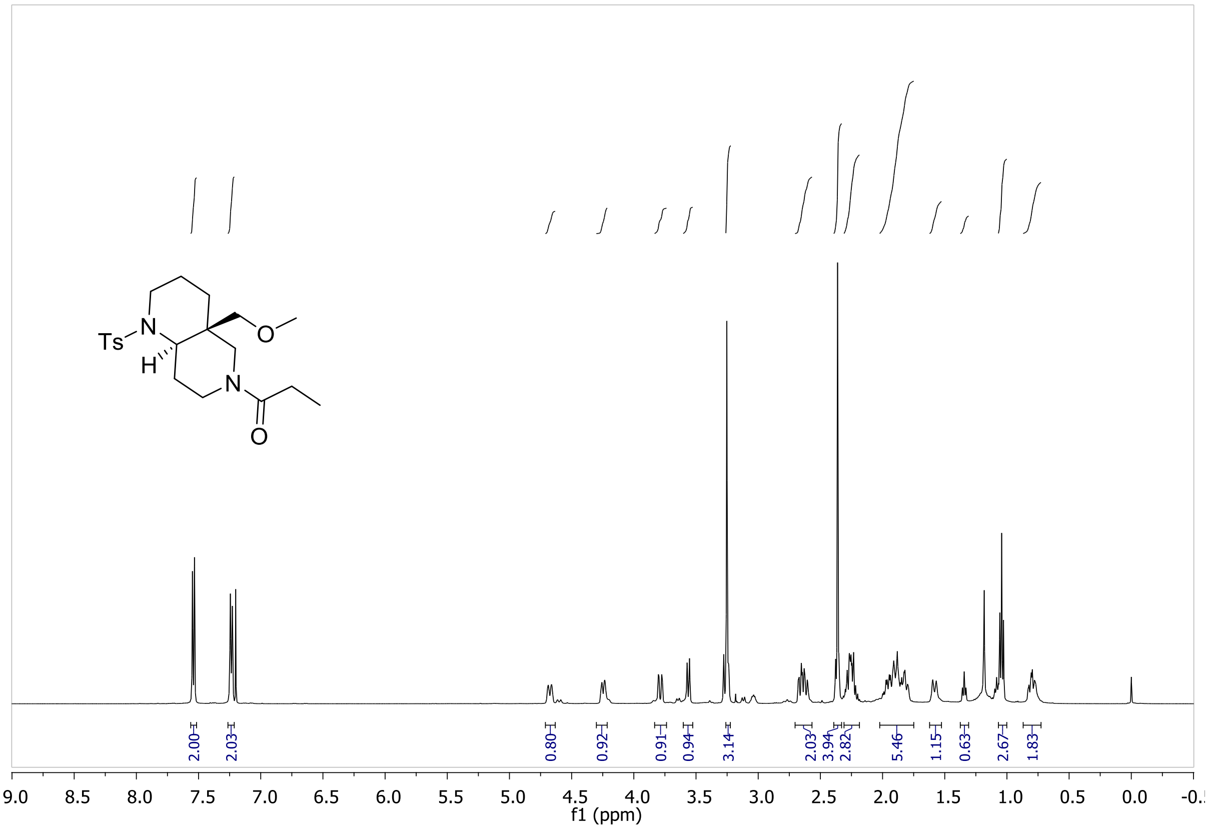
Ts
N
H
O
N
O
2.00
2.03
0.80
0.92
0.91
0.94
3.14
2.03
3.94
2.82
5.46
1.15
0.63
2.67
1.83
9.0 8.5 8.0 7.5 7.0 6.5 6.0 5.5 5.0 4.5 4.0 3.5 3.0 2.5 2.0 1.5 1.0 0.5 0.0
f1 (ppm)

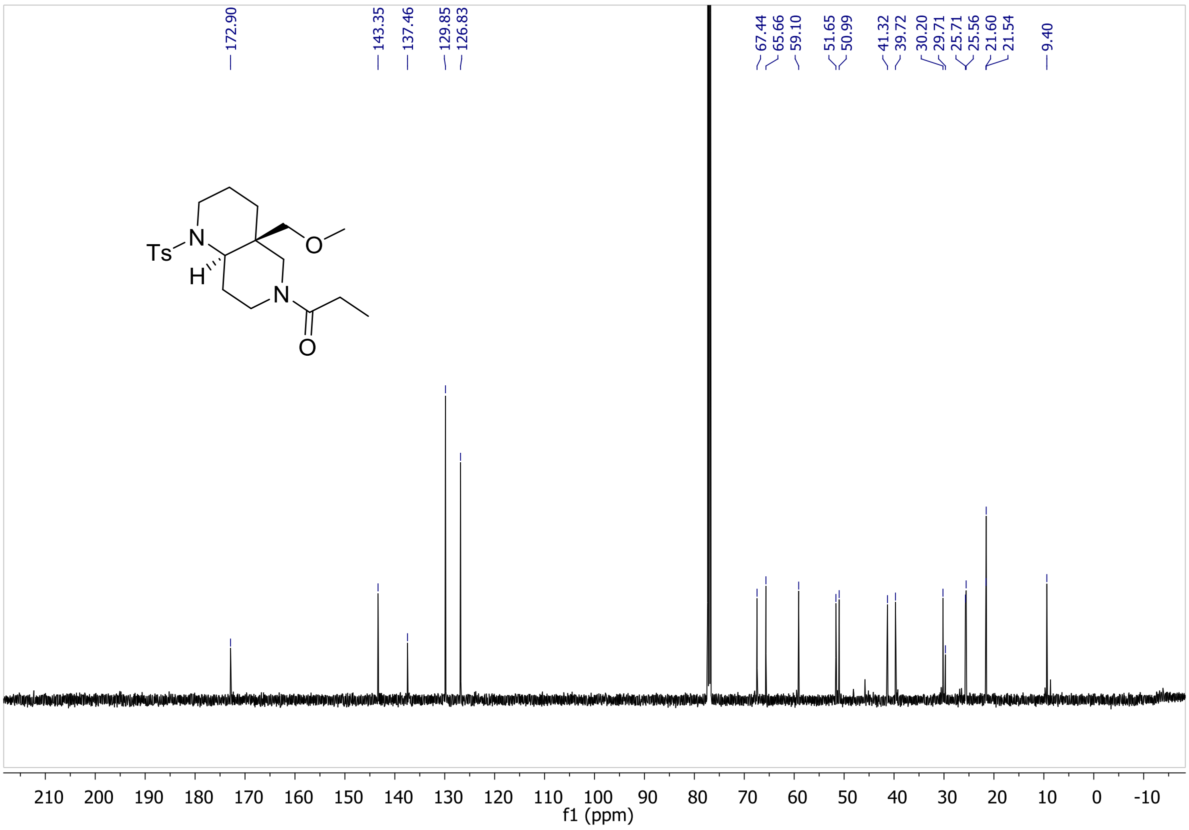
172.90
143.35
137.46
129.85
126.83
67.44
65.66
59.10
51.65
50.99
41.32
39.72
30.20
29.71
25.71
25.56
21.60
21.54
9.40
Ts
N
H
O
N
O
210 200 190 180 170 160 150 140 130 120 110 100 90 80 70 60 50 40 30 20 10 0 -10
f1 (ppm)

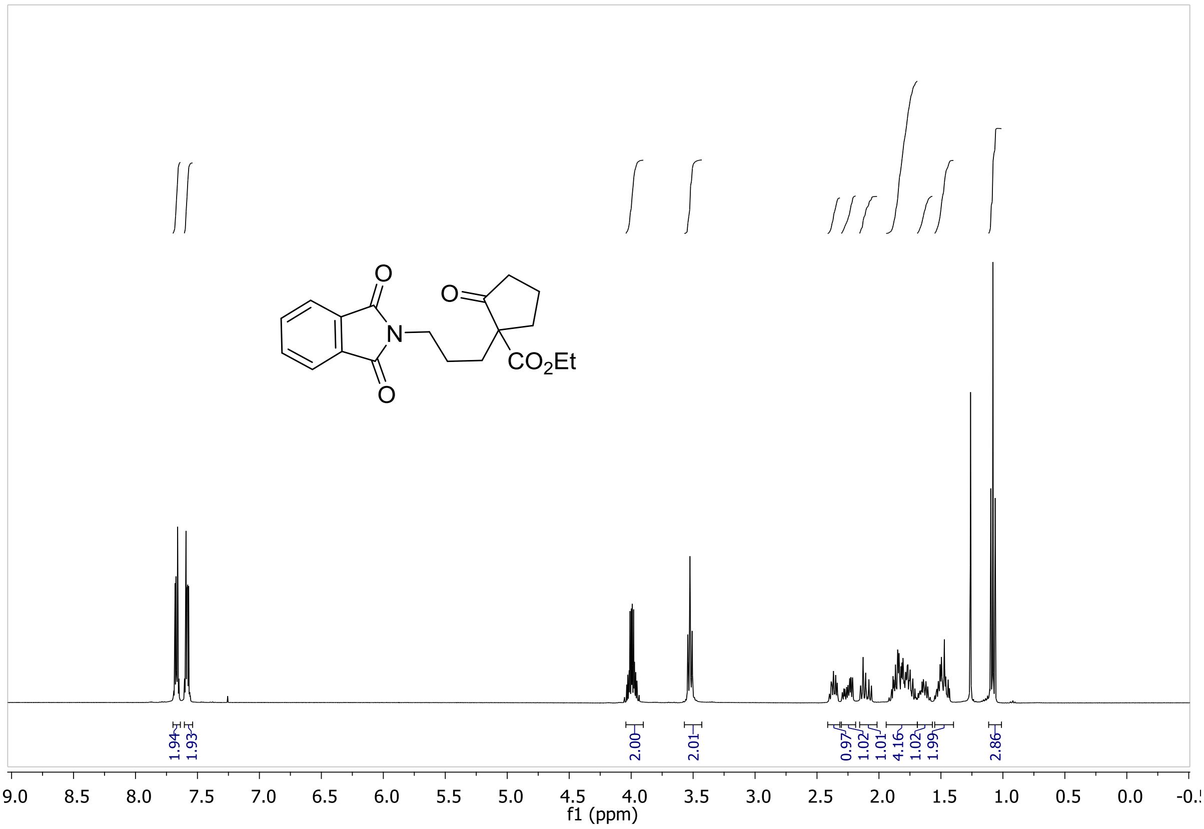
O
O
N
CO2Et
O
1.94
1.93
2.00
2.01
0.97
1.02
1.01
4.16
1.02
1.99
2.86
9.0 8.5 8.0 7.5 7.0 6.5 6.0 5.5 5.0 4.5 4.0 3.5 3.0 2.5 2.0 1.5 1.0 0.5 0.0
f1 (ppm)

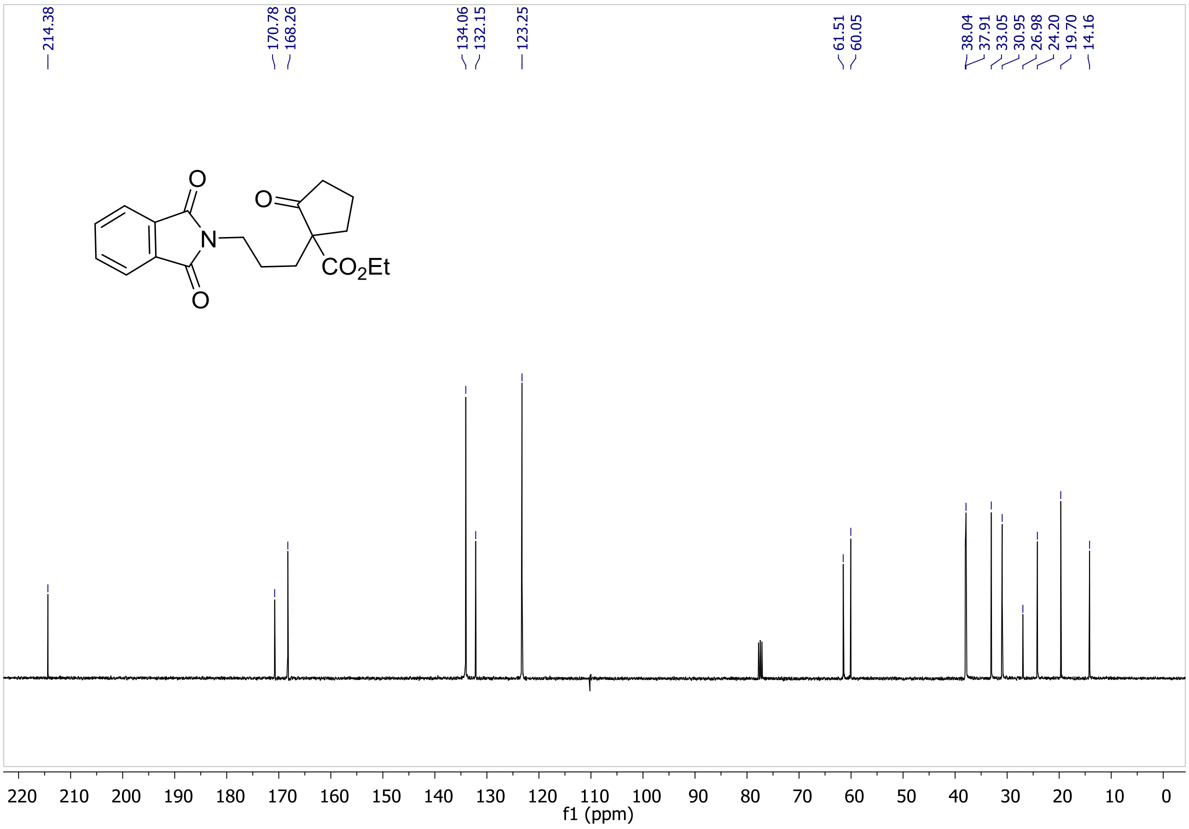
214.38
170.78
168.26
134.06
132.15
123.25
61.51
60.05
38.04
37.91
33.05
30.95
26.98
24.20
19.70
14.16
O
O
N
CO2Et
O
220 210 200 190 180 170 160 150 140 130 120 110 100 90 80 70 60 50 40 30 20 10 0
f1 (ppm)

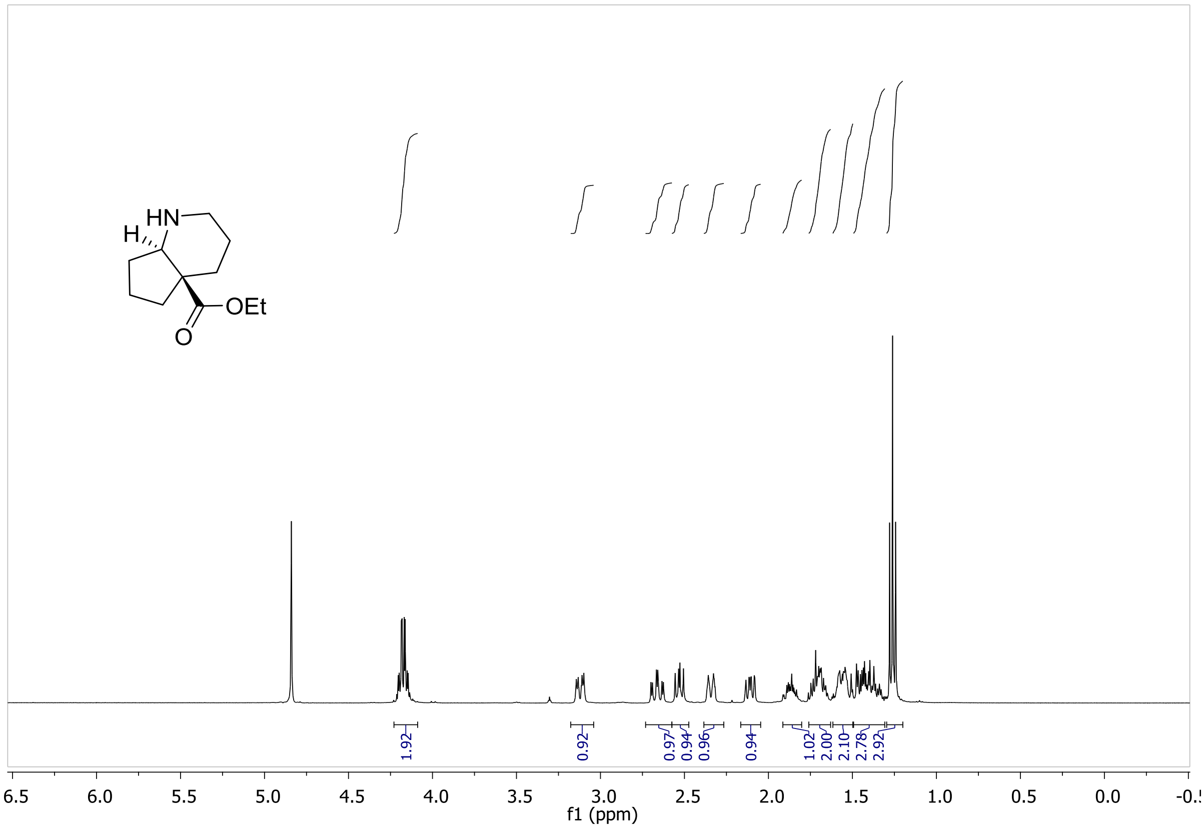
HN
H
OEt
O
1.92
0.92
0.97
0.94
0.96
0.94
1.02
2.00
2.10
2.78
2.92
6.5
6.0
5.5
5.0
4.5
4.0
3.5
3.0
2.5
2.0
1.5
1.0
0.5
0.0
f1 (ppm)

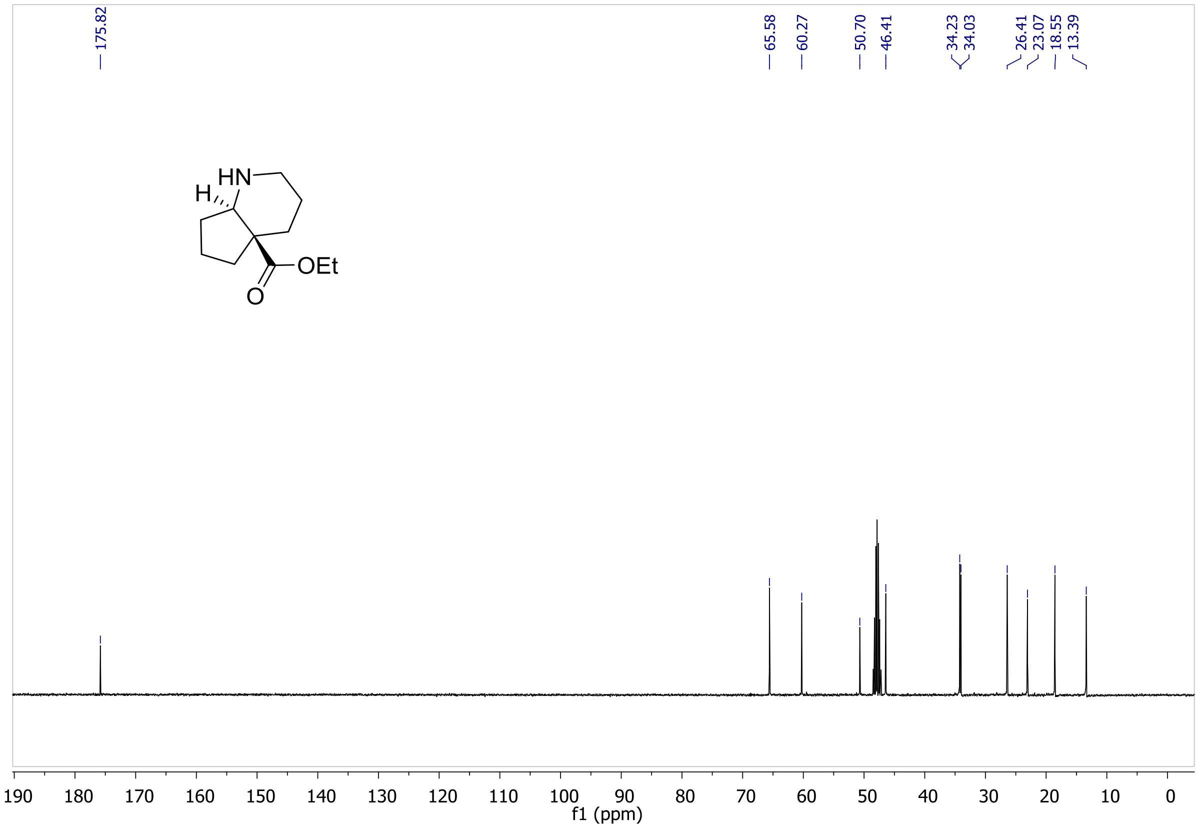
175.82
65.58
60.27
50.70
46.41
34.23
34.03
26.41
23.07
18.55
13.39
HN
H
OEt
O
190
180
170
160
150
140
130
120
110
100
90
80
70
60
50
40
30
20
10
0
f1 (ppm)

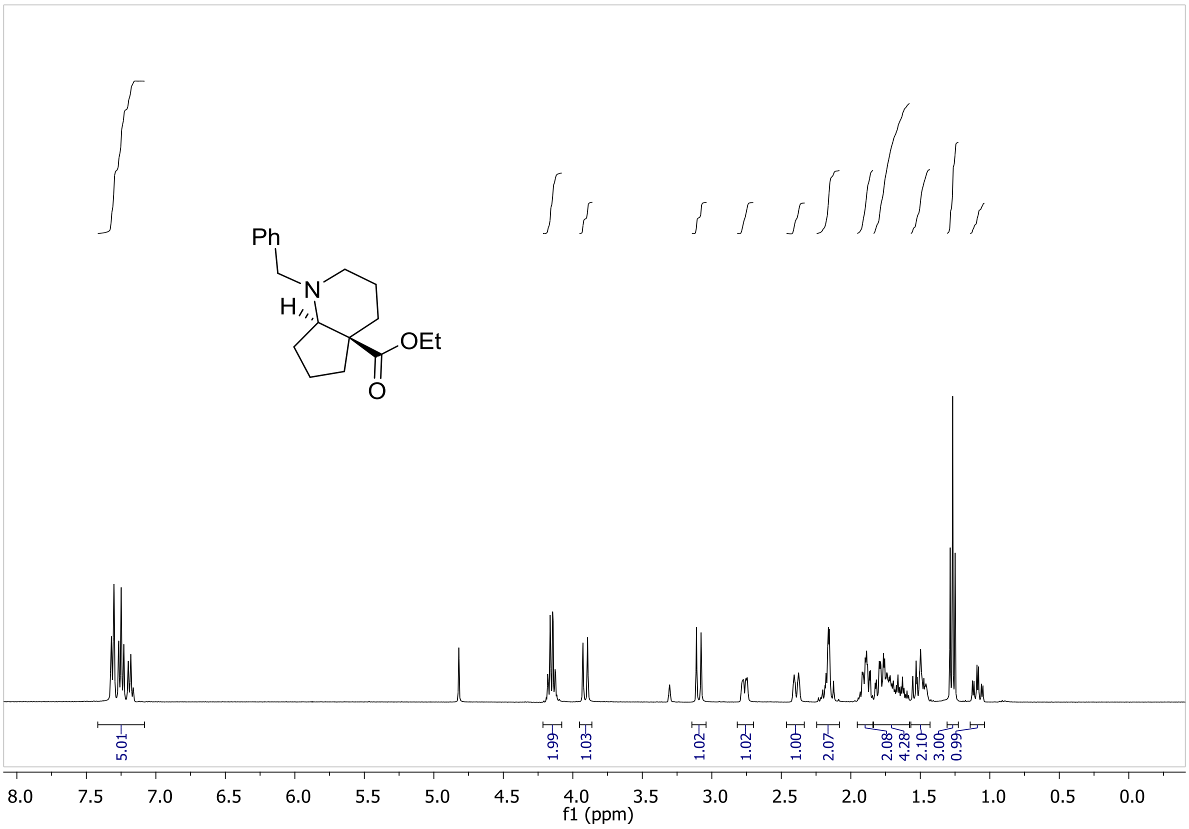
Ph
N
H
OEt
O
5.01
1.99
1.03
1.02
1.02
1.00
2.07
2.08
4.28
2.10
3.00
0.99
8.0 7.5 7.0 6.5 6.0 5.5 5.0 4.5 4.0 3.5 3.0 2.5 2.0 1.5 1.0 0.5 0.0
f1 (ppm)

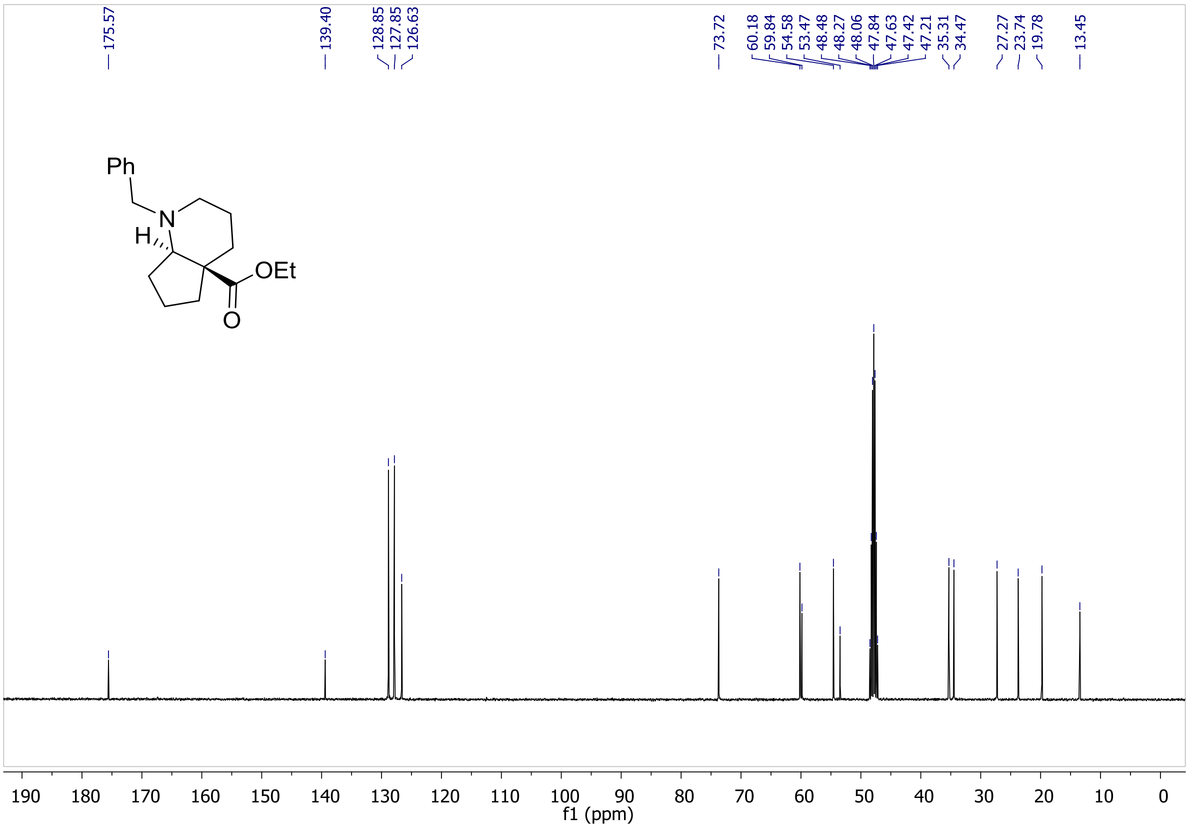
175.57
139.40
128.85
127.85
126.63
73.72
60.18
59.84
54.58
53.47
48.48
48.27
48.06
47.84
47.63
47.42
47.21
35.31
34.47
27.27
23.74
19.78
13.45
Ph
N
H
OEt
O
190 180 170 160 150 140 130 120 110 100 90 80 70 60 50 40 30 20 10 0
f1 (ppm)

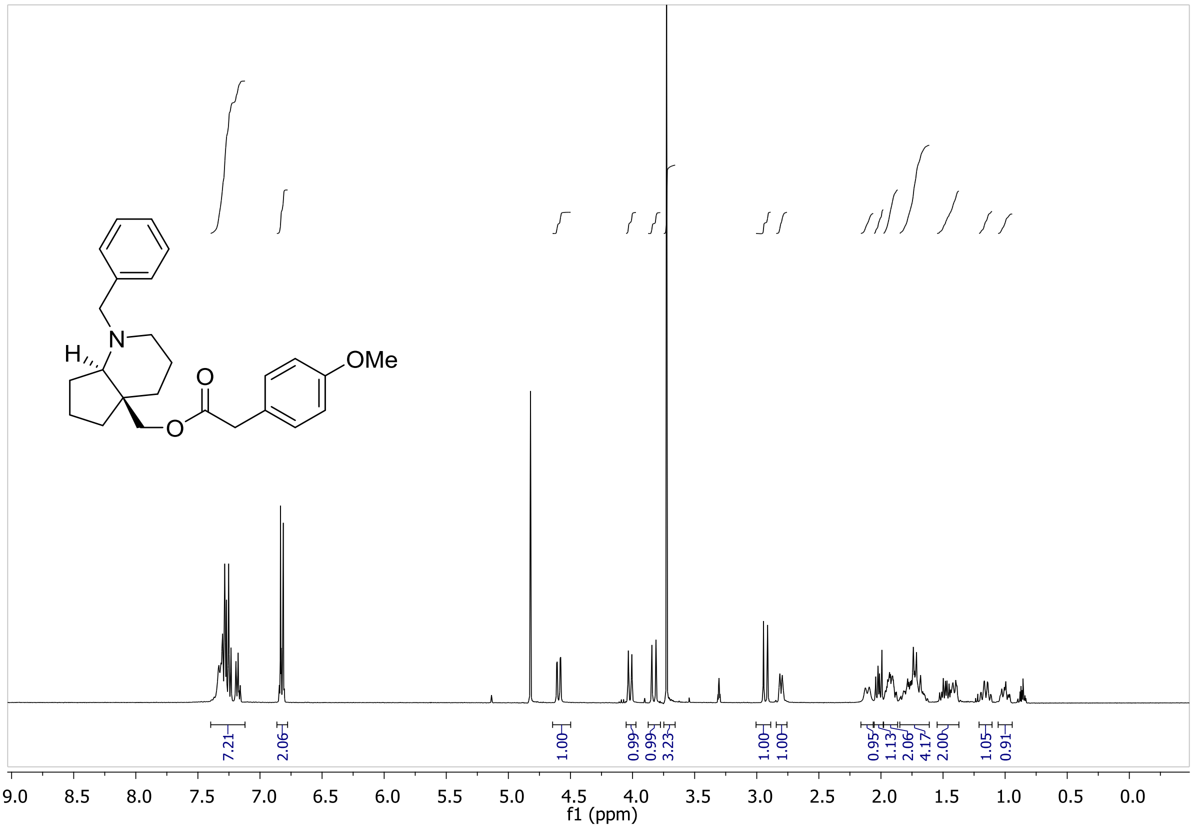

OMe
7.21
2.06
1.00
0.99
0.99
3.23
1.00
1.00
0.95
1.13
2.06
4.17
2.00
1.05
0.91
f1 (ppm)

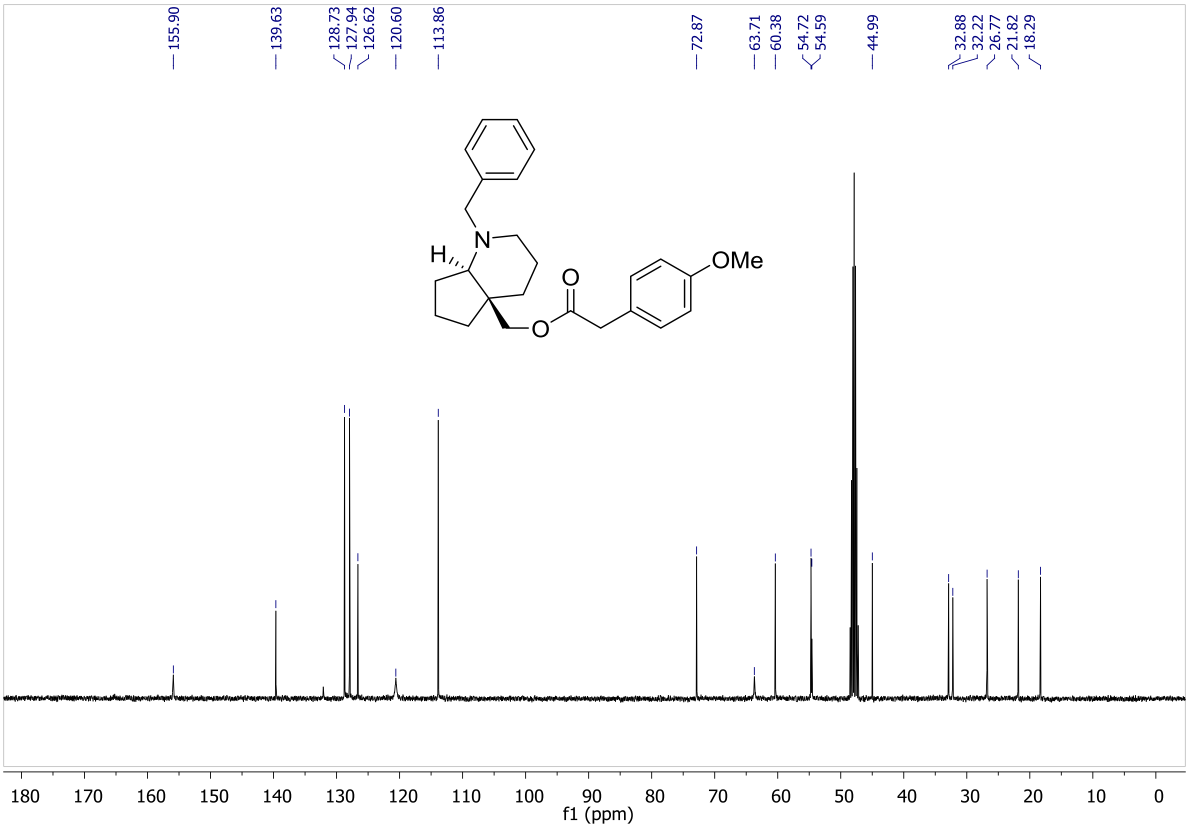

155.90
139.63
128.73
127.94
126.62
120.60
113.86
72.87
63.71
60.38
54.72
54.59
44.99
32.88
32.22
26.77
21.82
18.29
OMe
f1 (ppm)

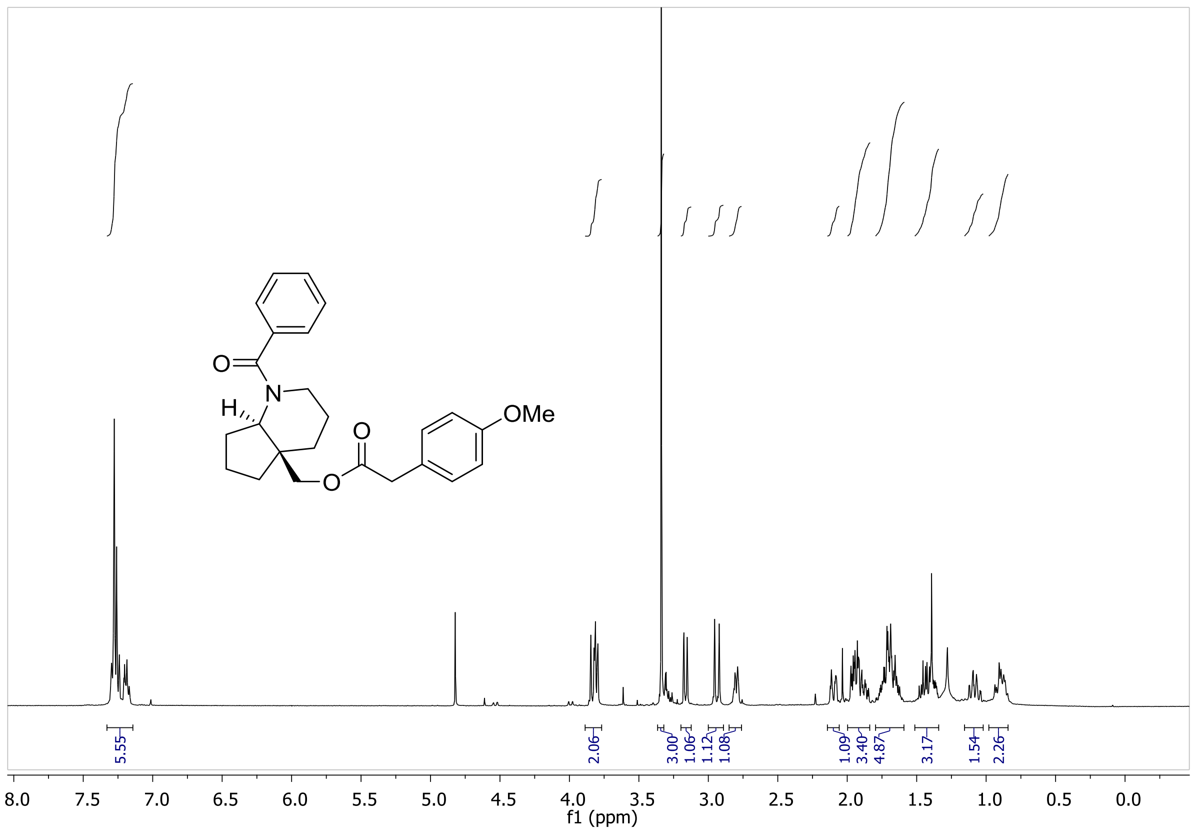
O
N
H
O
OMe
O
5.55
2.06
3.00
1.06
1.12
1.08
1.09
3.40
4.87
3.17
1.54
2.26
8.0 7.5 7.0 6.5 6.0 5.5 5.0 4.5 4.0 3.5 3.0 2.5 2.0 1.5 1.0 0.5 0.0
f1 (ppm)

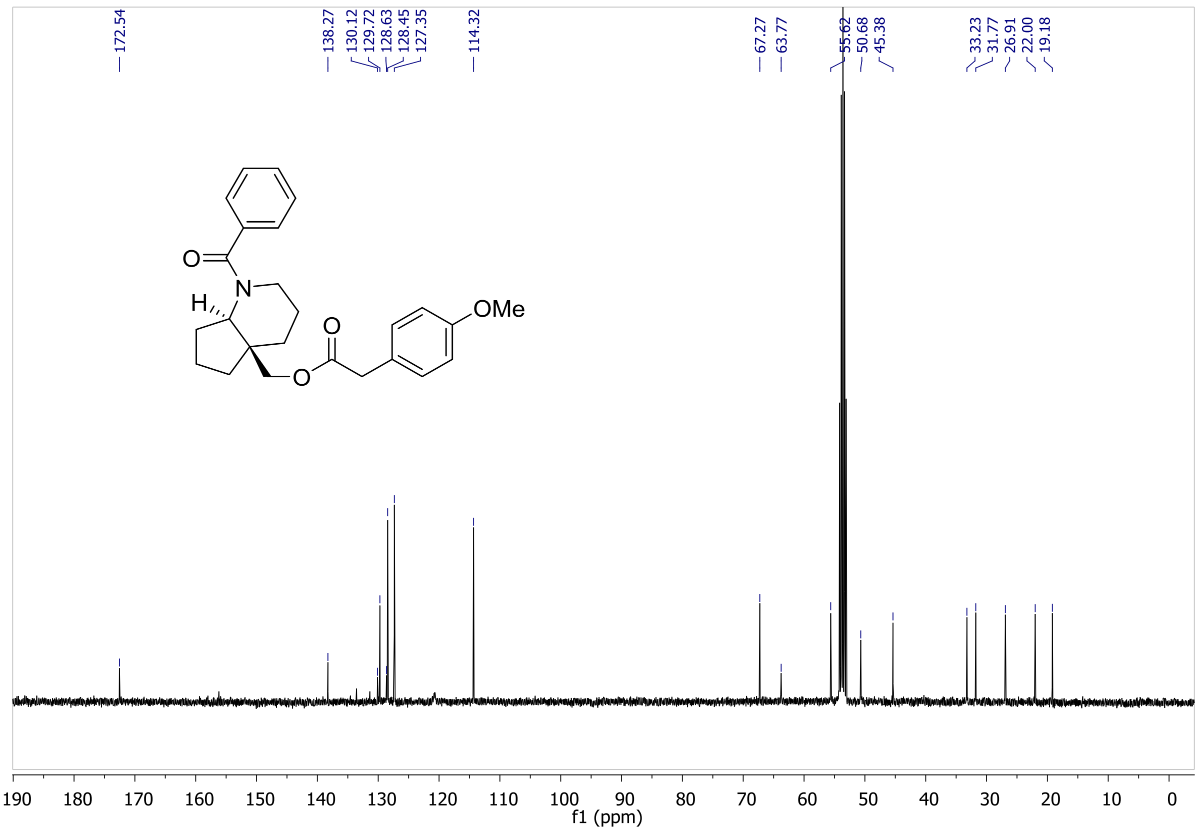
172.54
138.27
130.12
129.72
128.63
128.45
127.35
114.32
67.27
63.77
55.62
50.68
45.38
33.23
31.77
26.91
22.00
19.18
O
N
H
O
OMe
O
190 180 170 160 150 140 130 120 110 100 90 80 70 60 50 40 30 20 10 0
f1 (ppm)

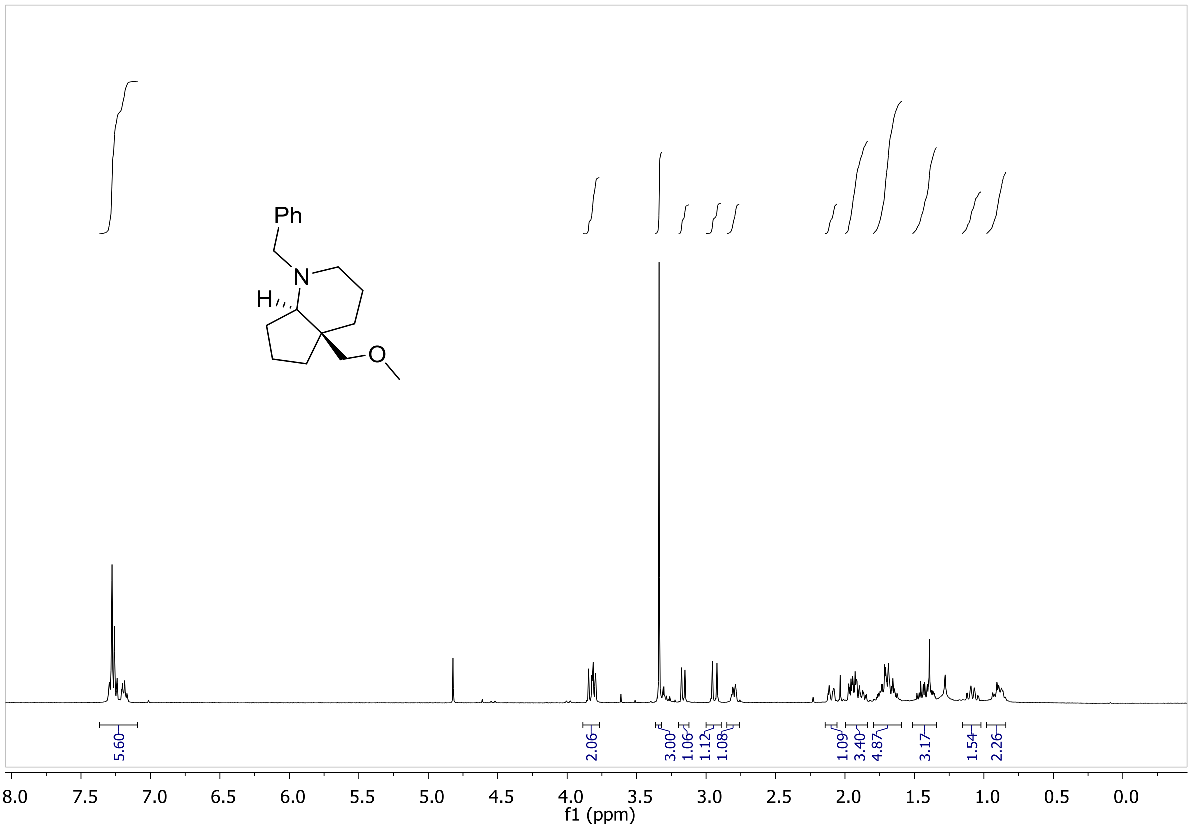
Ph
N
H
O
5.60
2.06
3.00
1.06
1.12
1.08
1.09
3.40
4.87
3.17
1.54
2.26
8.0 7.5 7.0 6.5 6.0 5.5 5.0 4.5 4.0 3.5 3.0 2.5 2.0 1.5 1.0 0.5 0.0
f1 (ppm)

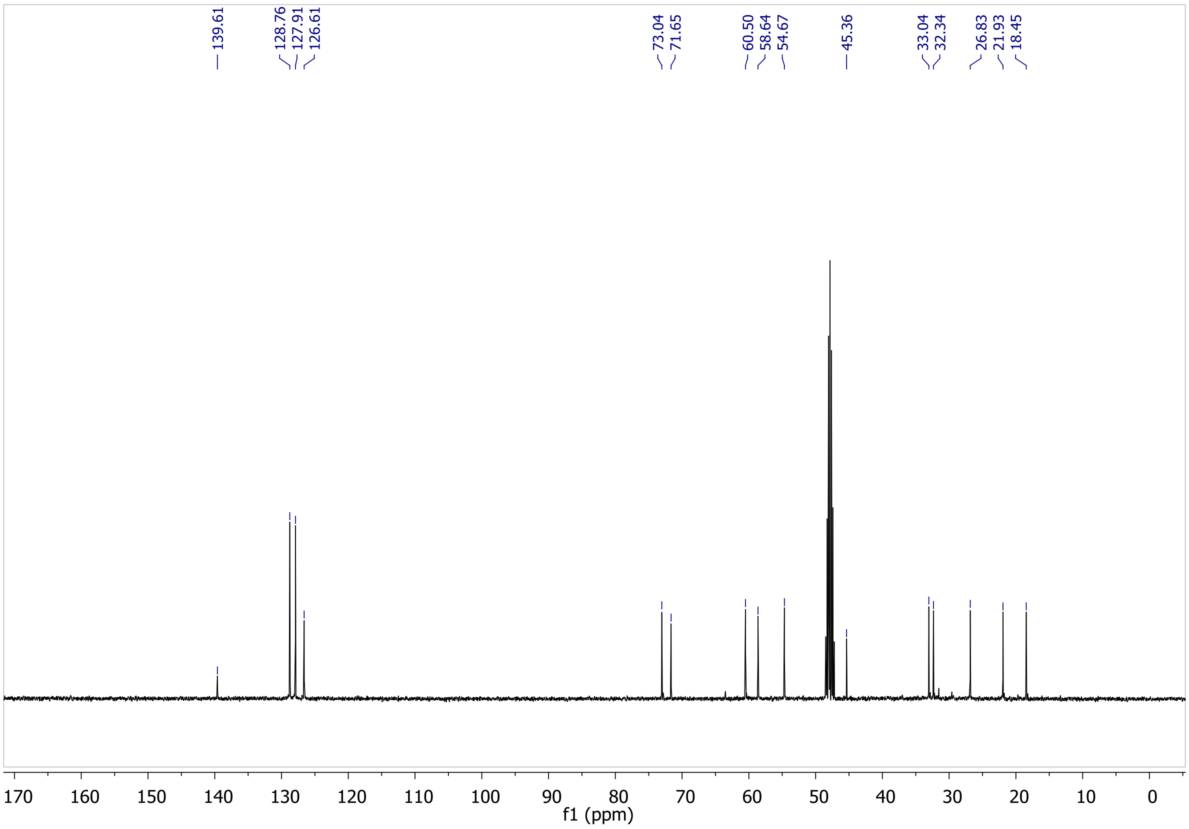
139.61
128.76
127.91
126.61
73.04
71.65
60.50
58.64
54.67
45.36
33.04
32.34
26.83
21.93
18.45
170 160 150 140 130 120 110 100 90 80 70 60 50 40 30 20 10 0
f1 (ppm)

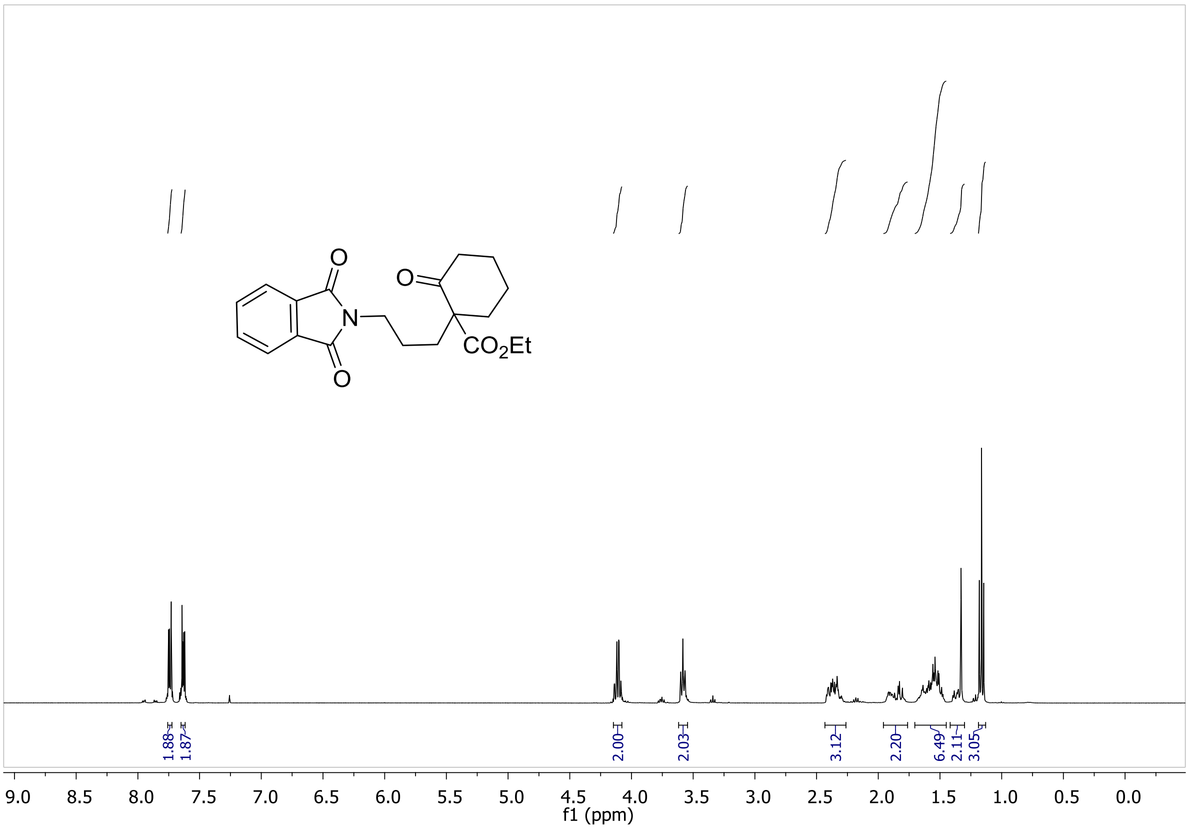

O
N
O
O
CO2Et
1.88
1.87
2.00
2.03
3.12
2.20
6.49
2.11
3.05
9.0 8.5 8.0 7.5 7.0 6.5 6.0 5.5 5.0 4.5 4.0 3.5 3.0 2.5 2.0 1.5 1.0 0.5 0.0
f1 (ppm)

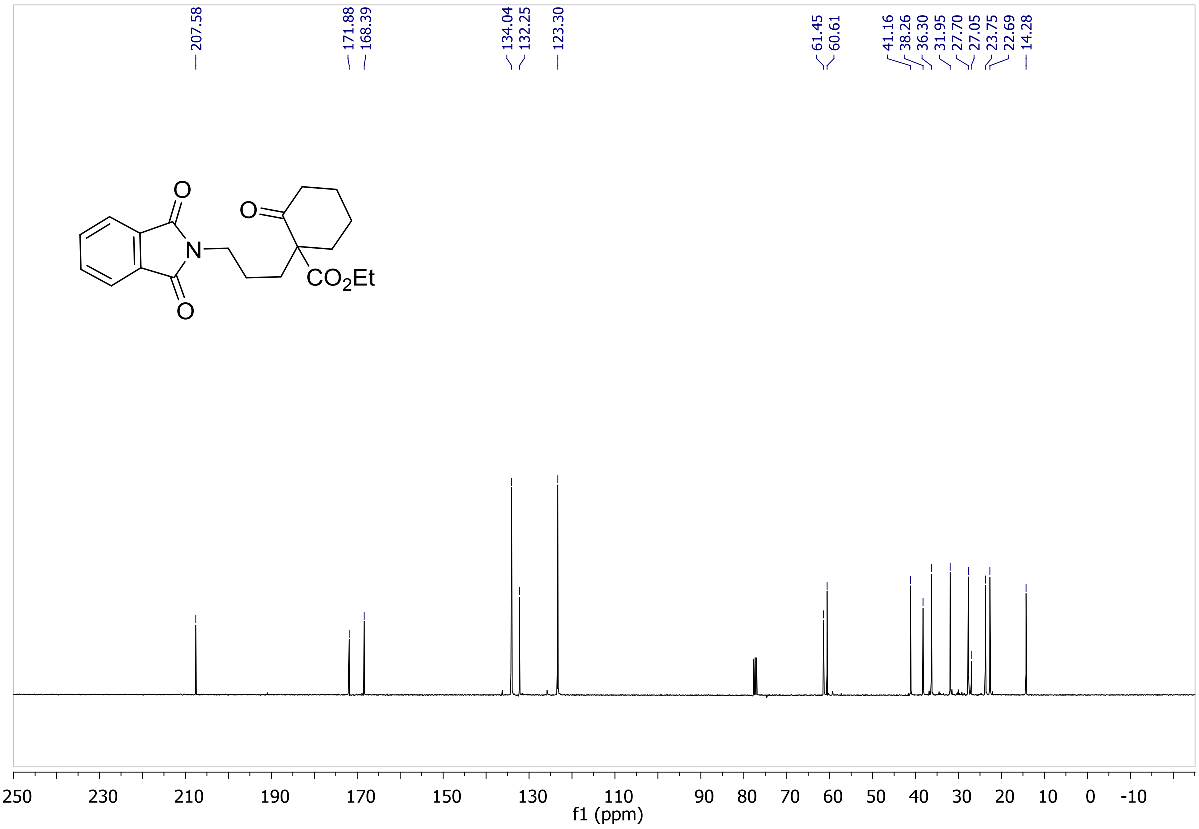

207.58
171.88
168.39
134.04
132.25
123.30
61.45
60.61
41.16
38.26
36.30
31.95
27.70
27.05
23.75
22.69
14.28
O
N
O
O
CO2Et
250 230 210 190 170 150 130 110 90 80 70 60 50 40 30 20 10 0 -10
f1 (ppm)

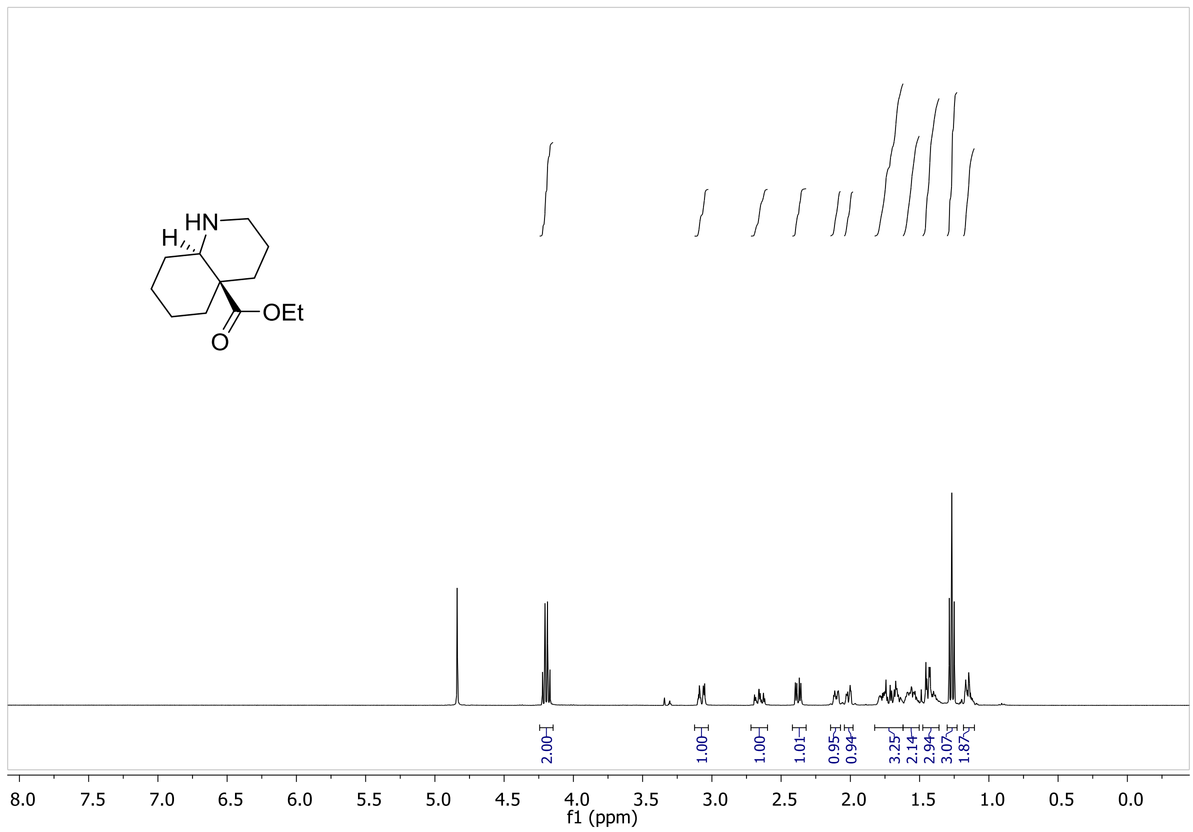
HN
H
OEt
O
2.00
1.00
1.00
1.01
0.95
0.94
3.25
2.14
2.94
3.07
1.87
8.0 7.5 7.0 6.5 6.0 5.5 5.0 4.5 4.0 3.5 3.0 2.5 2.0 1.5 1.0 0.5 0.0
f1 (ppm)

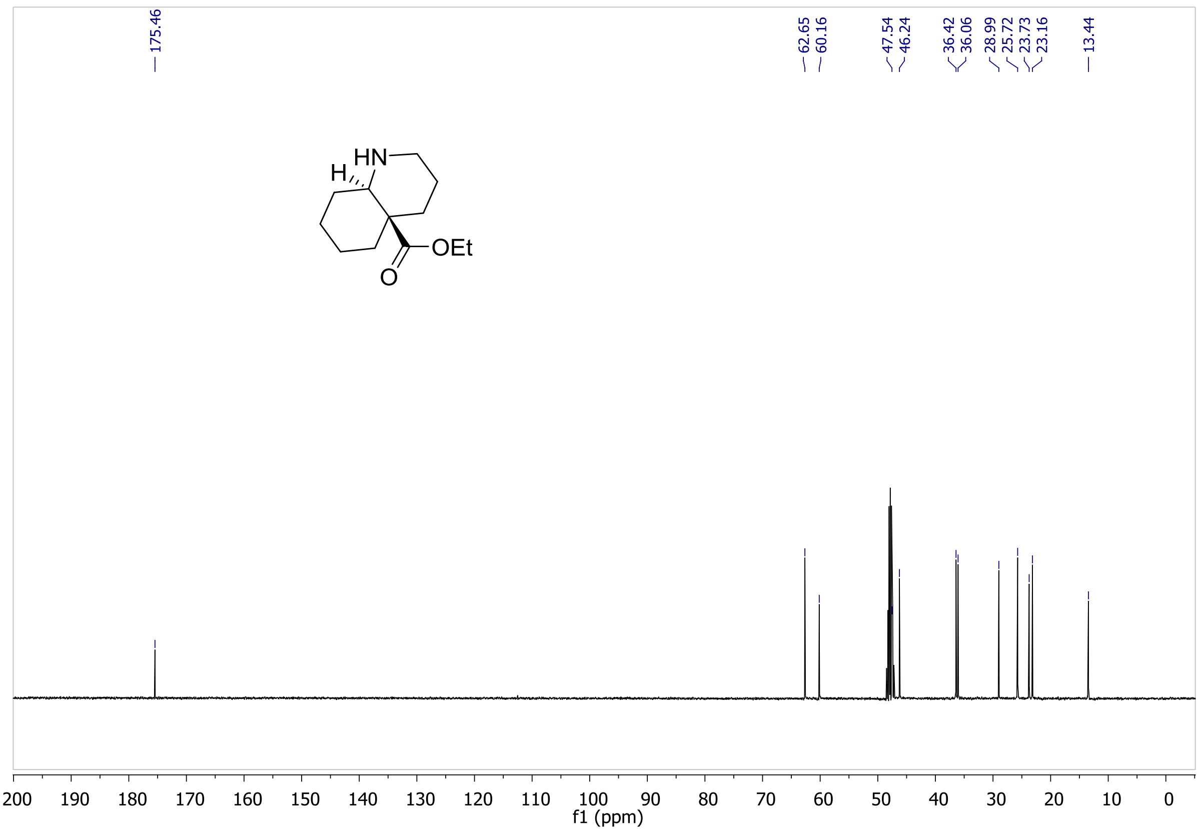
175.46
62.65
60.16
47.54
46.24
36.42
36.06
28.99
25.72
23.73
23.16
13.44
HN
H
OEt
O
200 190 180 170 160 150 140 130 120 110 100 90 80 70 60 50 40 30 20 10 0
f1 (ppm)

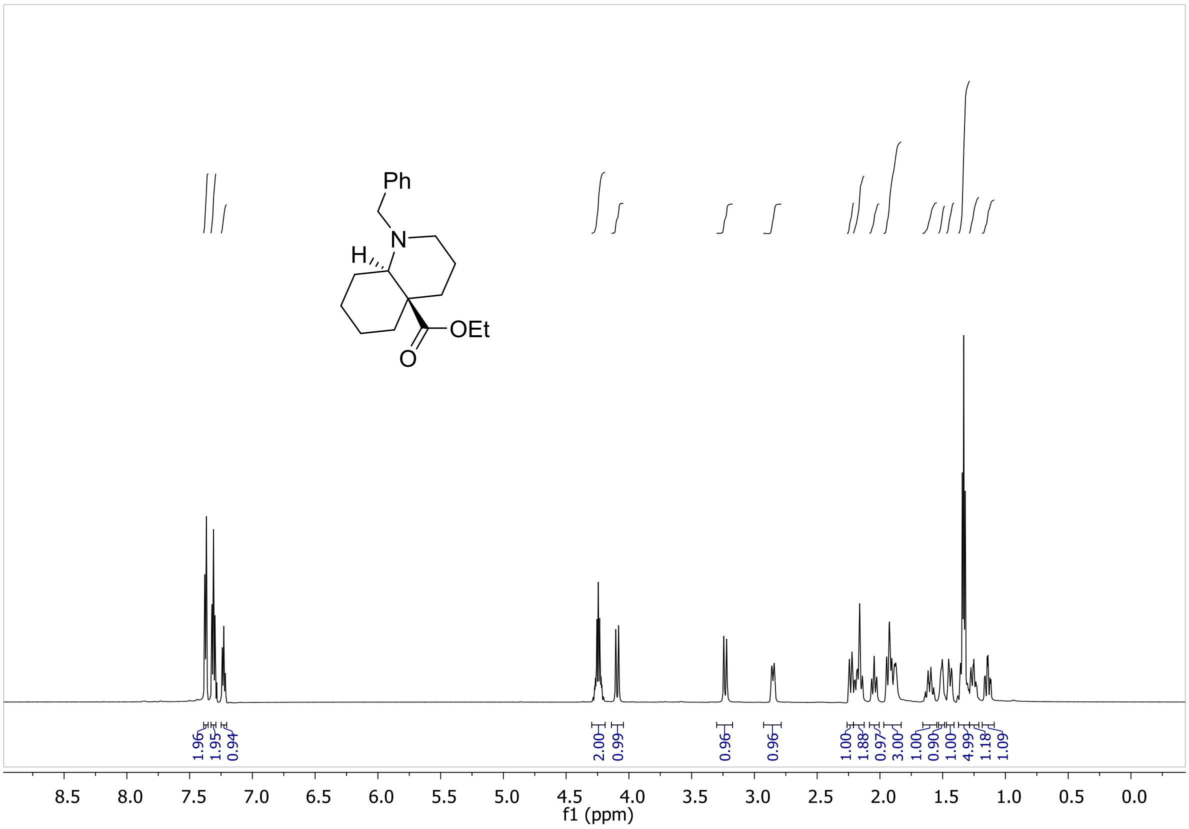
Ph
N
H
OEt
O
1.96
1.95
0.94
2.00
0.99
0.96
0.96
1.00
1.88
0.97
3.00
1.00
0.90
1.00
4.99
1.18
1.09
8.5
8.0
7.5
7.0
6.5
6.0
5.5
5.0
4.5
4.0
3.5
3.0
2.5
2.0
1.5
1.0
0.5
0.0
f1 (ppm)

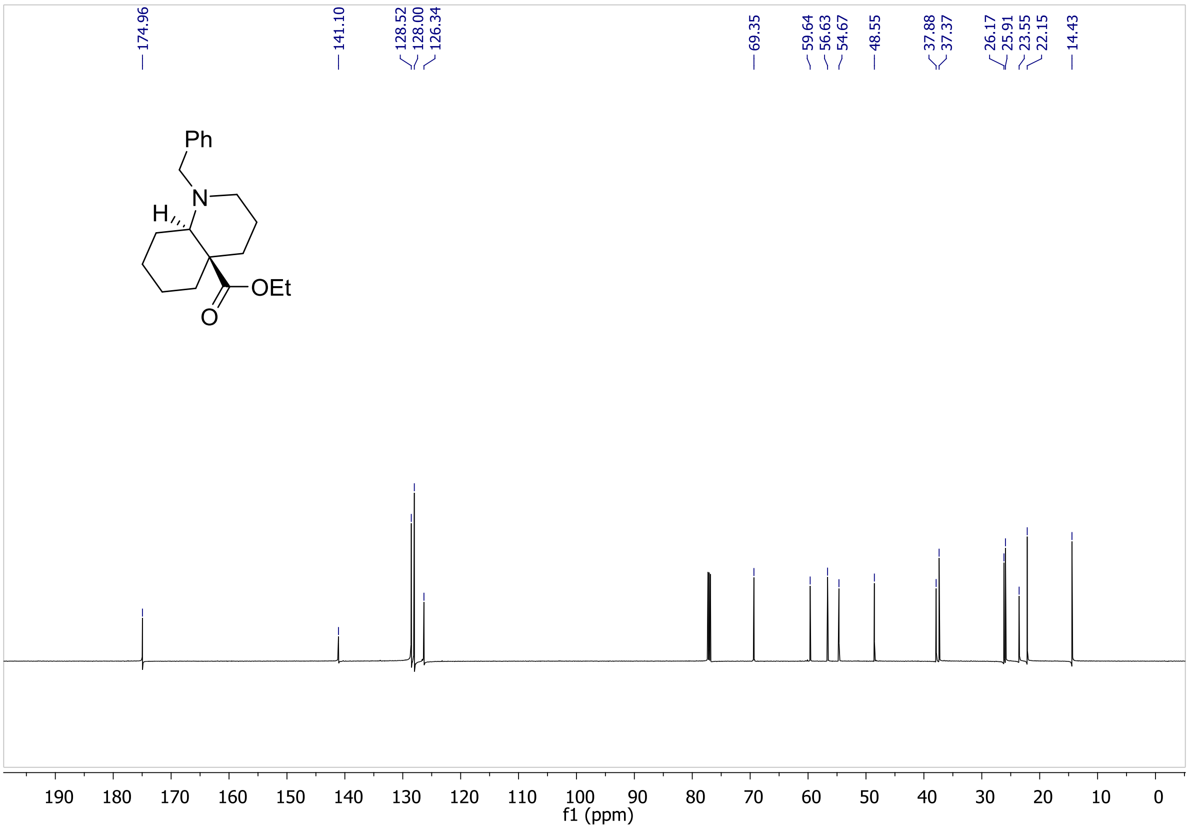
174.96
141.10
128.52
128.00
126.34
69.35
59.64
56.63
54.67
48.55
37.88
37.37
26.17
25.91
23.55
22.15
14.43
Ph
N
H
OEt
O
190
180
170
160
150
140
130
120
110
100
90
80
70
60
50
40
30
20
10
0
f1 (ppm)

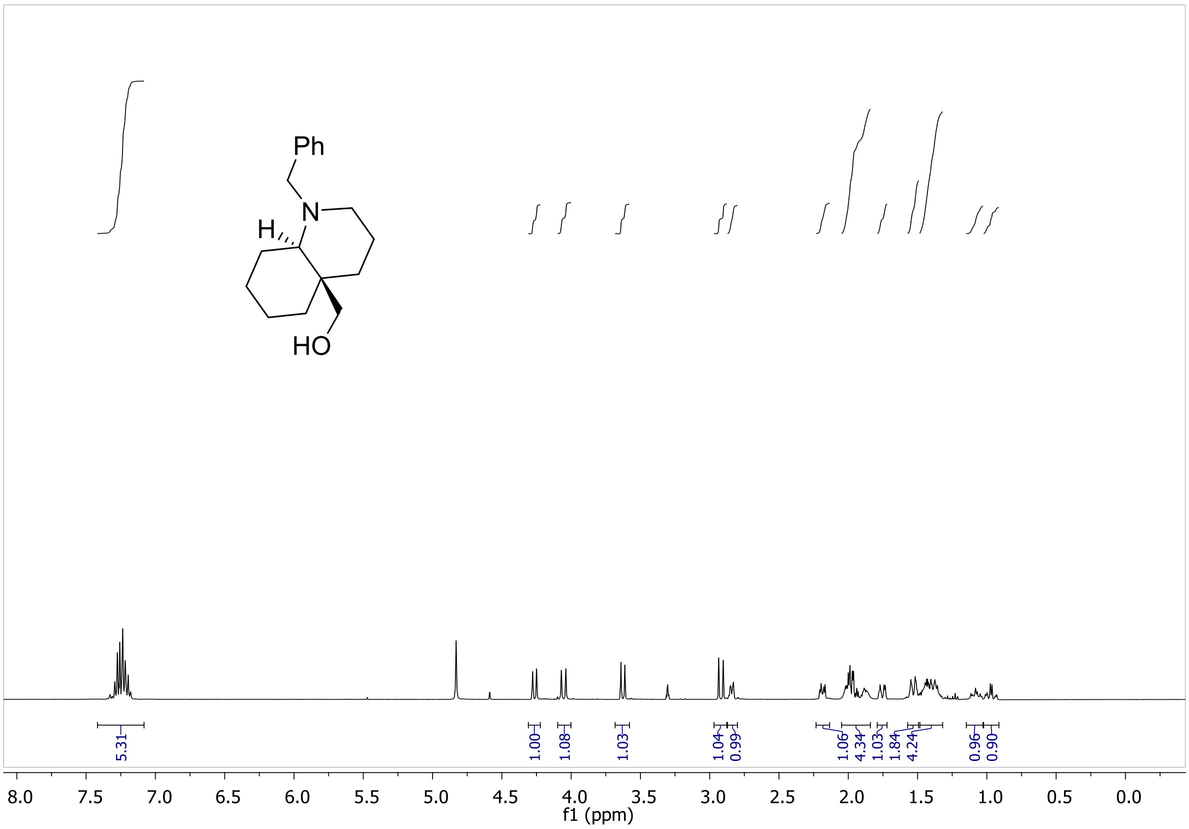
Ph
N
H
HO
5.31
1.00
1.08
1.03
1.04
0.99
1.06
4.34
1.03
1.84
4.24
0.96
0.90
8.0 7.5 7.0 6.5 6.0 5.5 5.0 4.5 4.0 3.5 3.0 2.5 2.0 1.5 1.0 0.5 0.0
f1 (ppm)

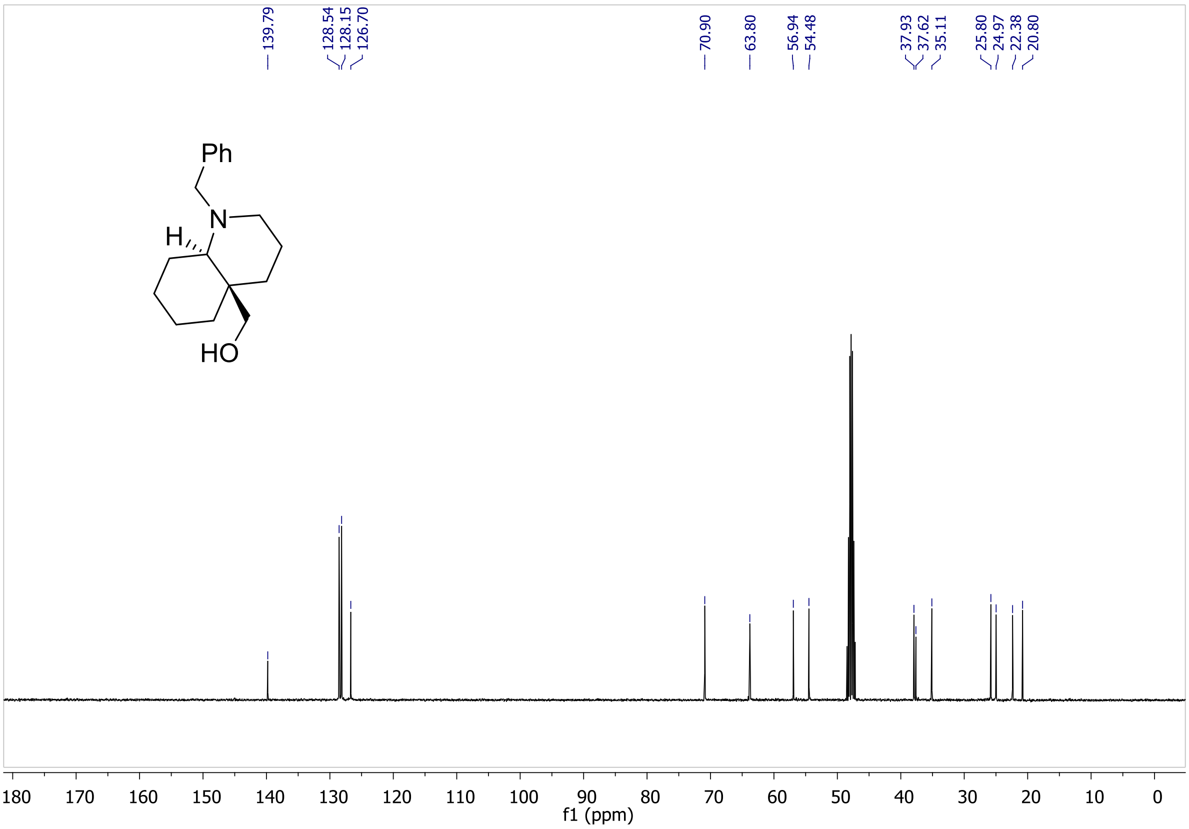
139.79
128.54
128.15
126.70
70.90
63.80
56.94
54.48
37.93
37.62
35.11
25.80
24.97
22.38
20.80
Ph
N
H
HO
180 170 160 150 140 130 120 110 100 90 80 70 60 50 40 30 20 10 0
f1 (ppm)

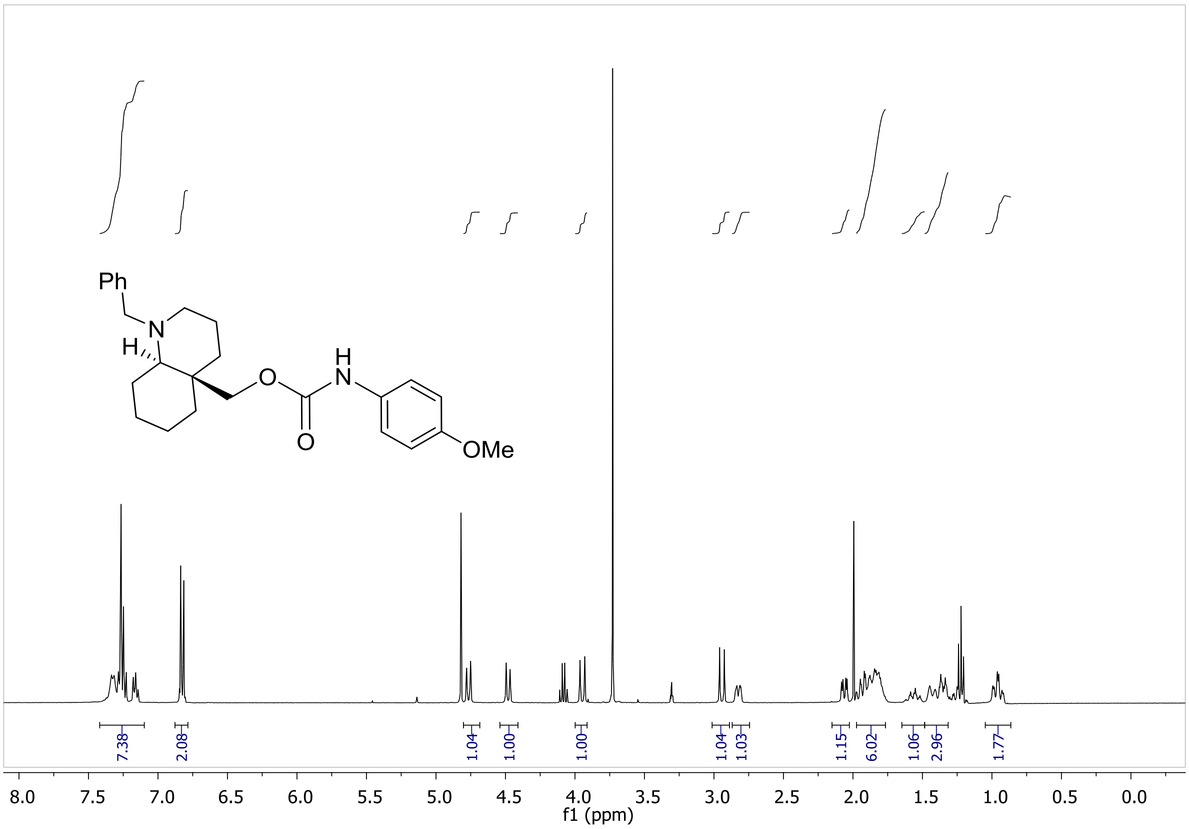
Ph
N
H
O
H
N
O
OMe
7.38
2.08
1.04
1.00
1.00
1.04
1.03
1.15
6.02
1.06
2.96
1.77
8.0
7.5
7.0
6.5
6.0
5.5
5.0
4.5
4.0
3.5
3.0
2.5
2.0
1.5
1.0
0.5
0.0
f1 (ppm)

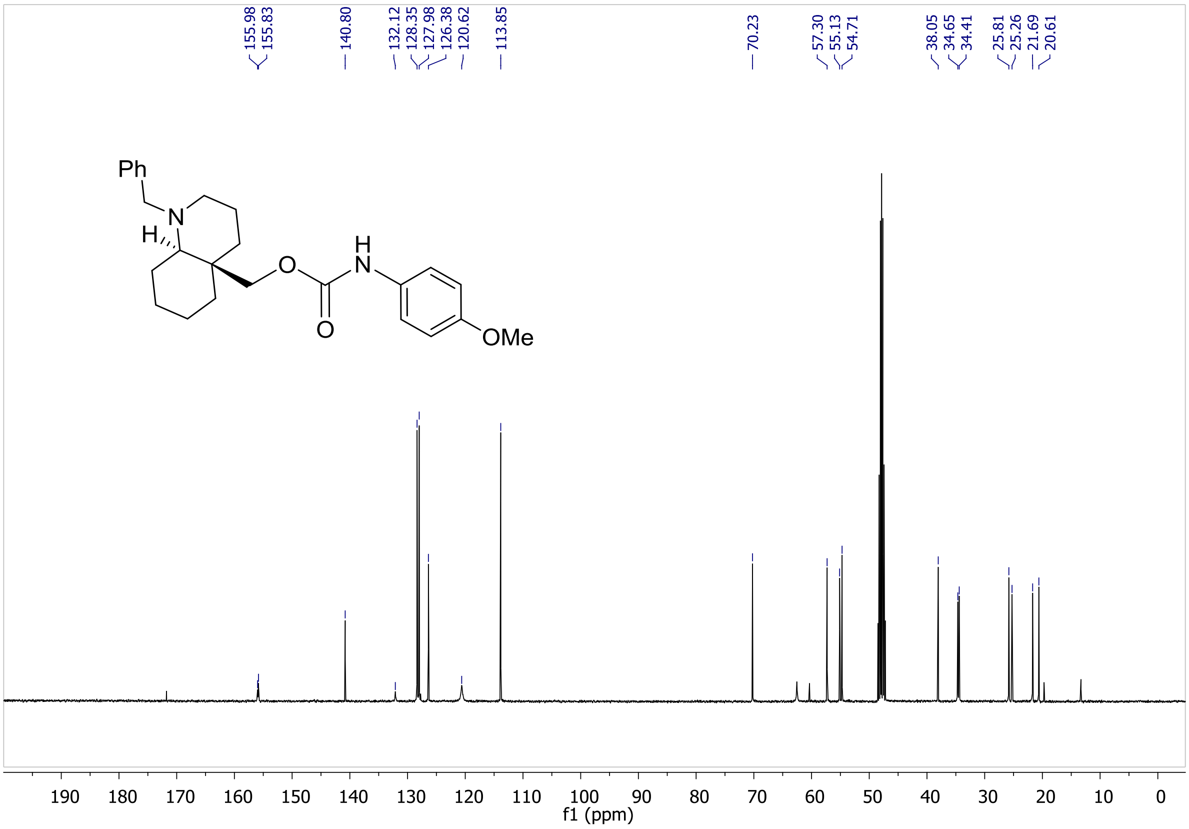
155.98
155.83
140.80
132.12
128.35
127.98
126.38
120.62
113.85
70.23
57.30
55.13
54.71
38.05
34.65
34.41
25.81
25.26
21.69
20.61
Ph
N
H
O
H
N
O
OMe
190
180
170
160
150
140
130
120
110
100
90
80
70
60
50
40
30
20
10
0
f1 (ppm)

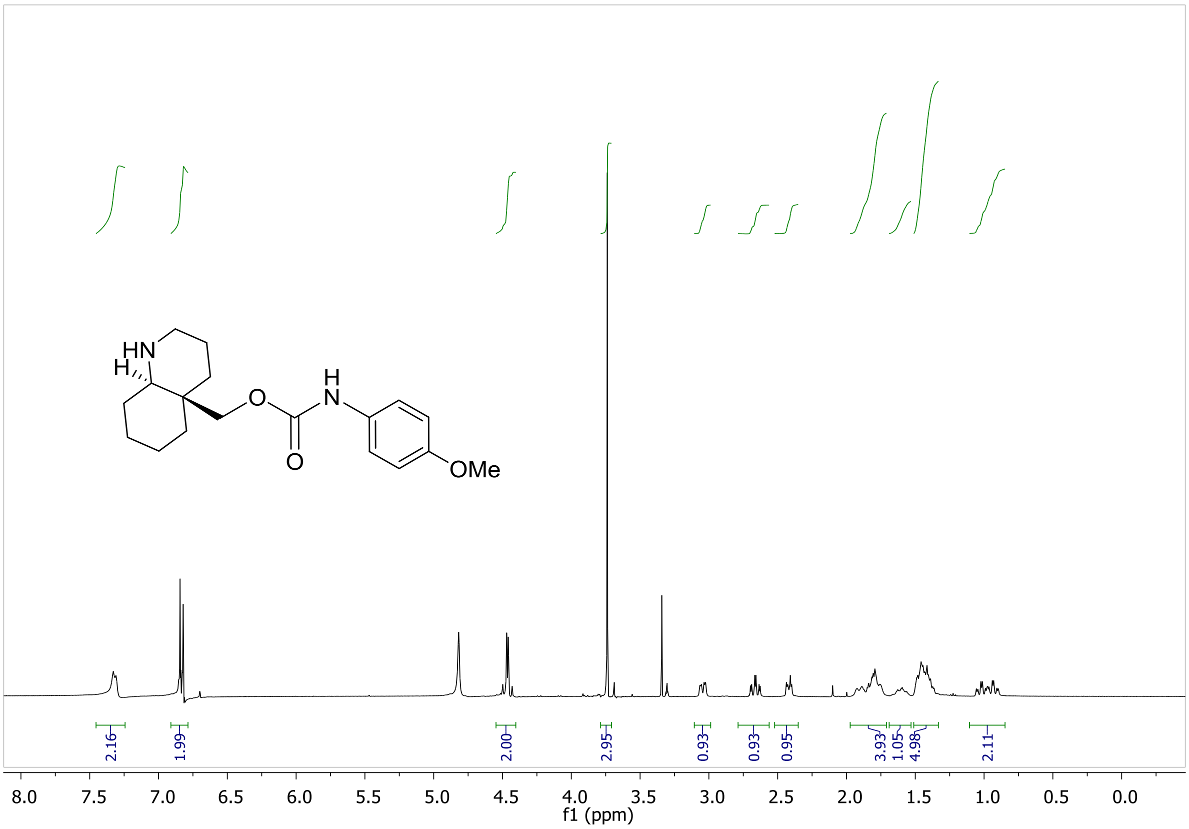
HN
H
O
H
N
O
OMe
2.16
1.99
2.00
2.95
0.93
0.93
0.95
3.93
1.05
4.98
2.11
8.0 7.5 7.0 6.5 6.0 5.5 5.0 4.5 4.0 3.5 3.0 2.5 2.0 1.5 1.0 0.5 0.0
f1 (ppm)

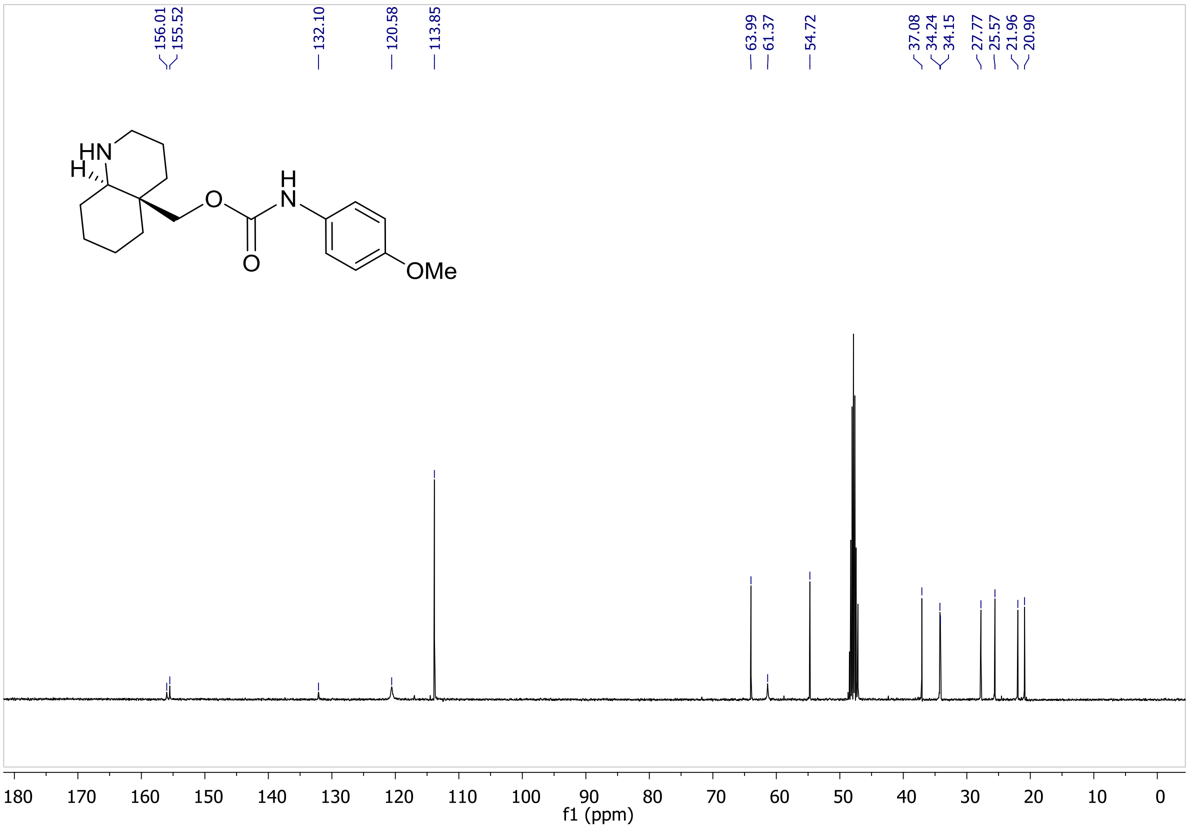
156.01
155.52
132.10
120.58
113.85
63.99
61.37
54.72
37.08
34.24
34.15
27.77
25.57
21.96
20.90
HN
H
O
H
N
O
OMe
180 170 160 150 140 130 120 110 100 90 80 70 60 50 40 30 20 10 0
f1 (ppm)

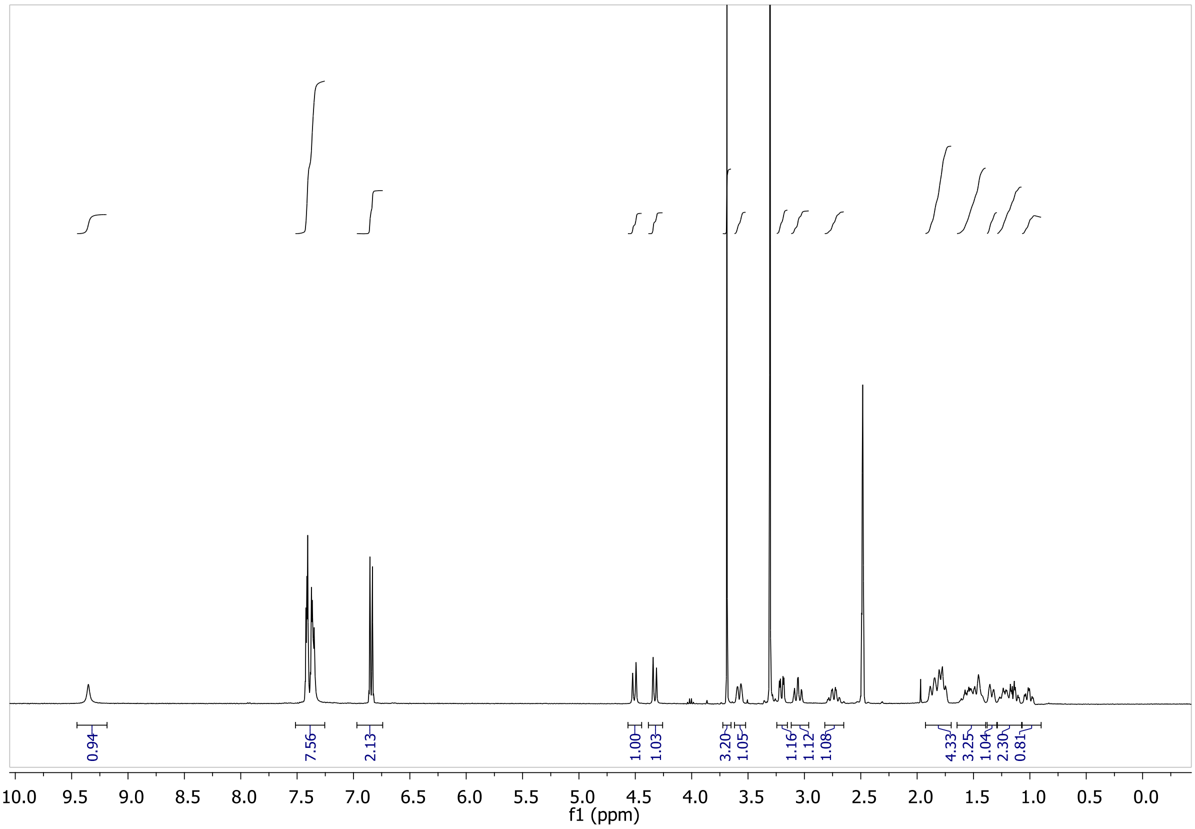
0.94
7.56
2.13
1.00
1.03
3.20
1.05
1.16
1.12
1.08
4.33
3.25
1.04
2.30
0.81
10.0 9.5 9.0 8.5 8.0 7.5 7.0 6.5 6.0 5.5 5.0 4.5 4.0 3.5 3.0 2.5 2.0 1.5 1.0 0.5 0.0
f1 (ppm)

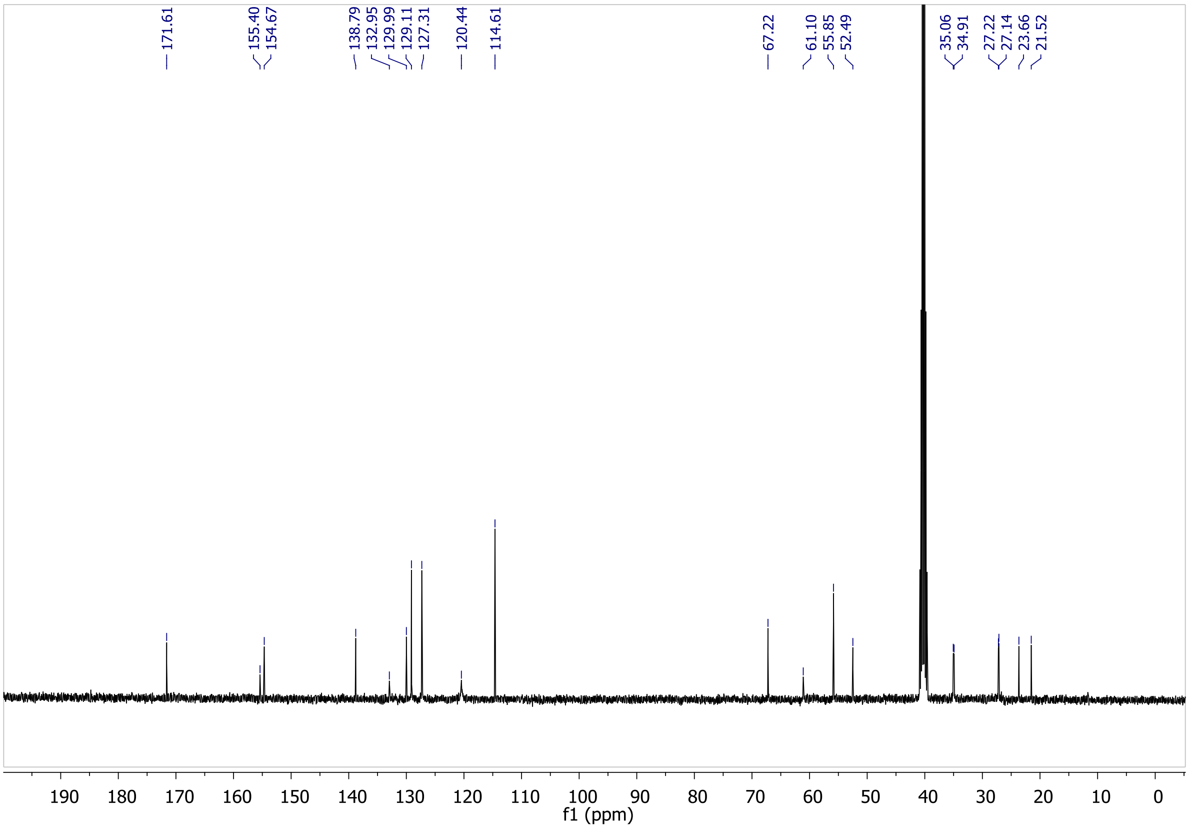
171.61
155.40
154.67
138.79
132.95
129.99
129.11
127.31
120.44
114.61
67.22
61.10
55.85
52.49
35.06
34.91
27.22
27.14
23.66
21.52
190 180 170 160 150 140 130 120 110 100 90 80 70 60 50 40 30 20 10 0
f1 (ppm)

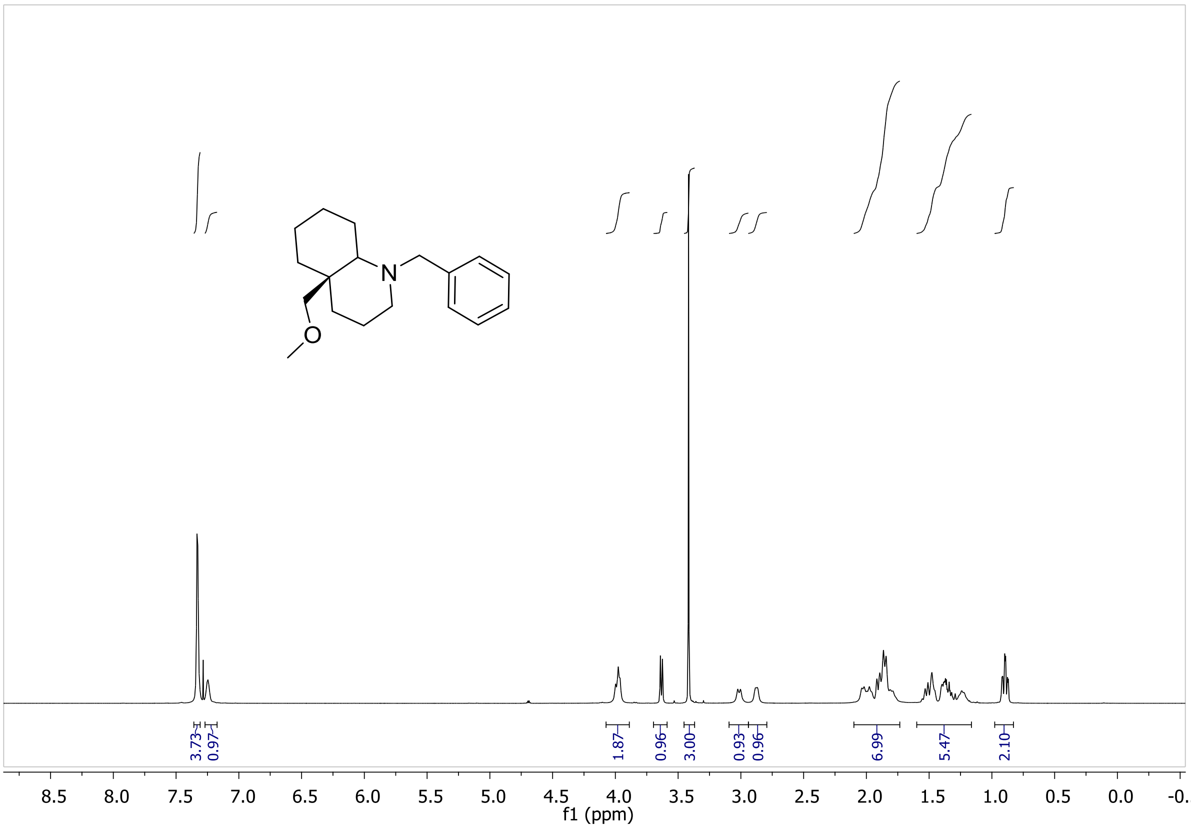

3.73
0.97
1.87
0.96
3.00
0.93
0.96
6.99
5.47
2.10
8.5 8.0 7.5 7.0 6.5 6.0 5.5 5.0 4.5 4.0 3.5 3.0 2.5 2.0 1.5 1.0 0.5 0.0 -0.5
f1 (ppm)
N
O

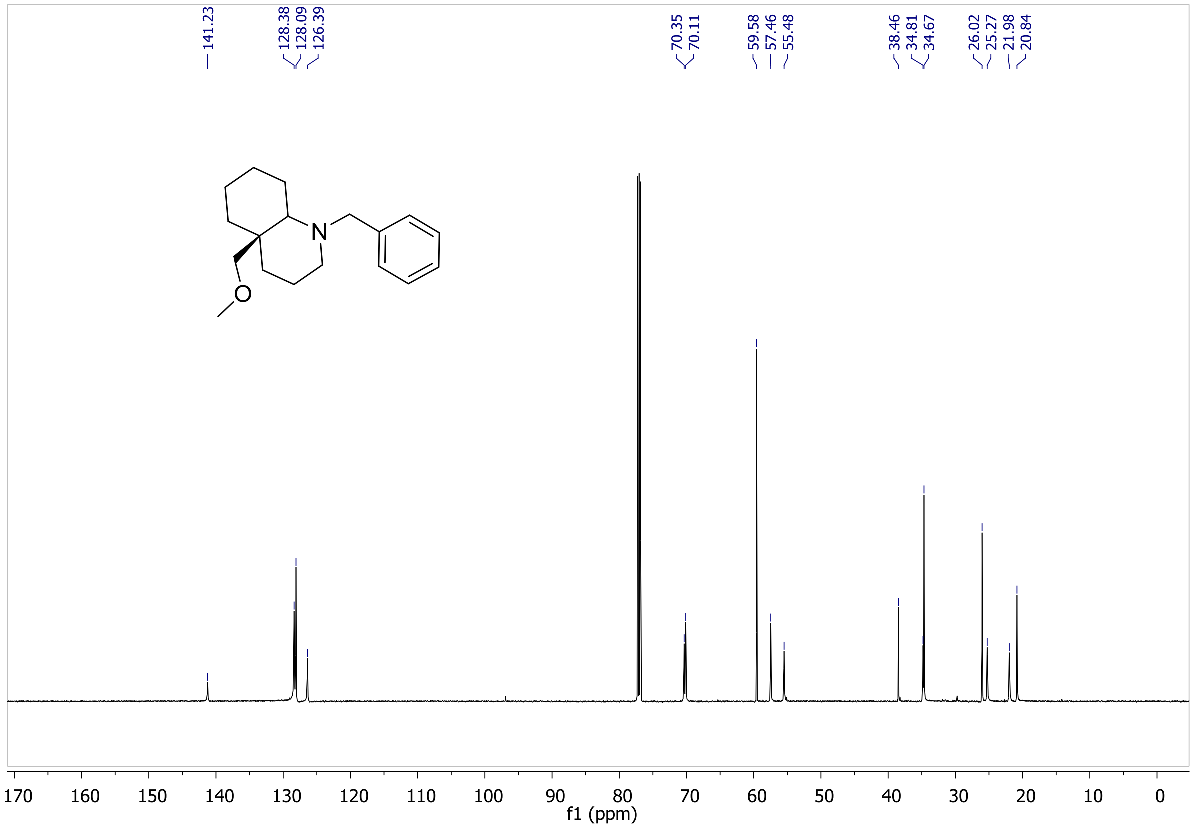

141.23
128.38
128.09
126.39
70.35
70.11
59.58
57.46
55.48
38.46
34.81
34.67
26.02
25.27
21.98
20.84
170 160 150 140 130 120 110 100 90 80 70 60 50 40 30 20 10 0
f1 (ppm)
N
O

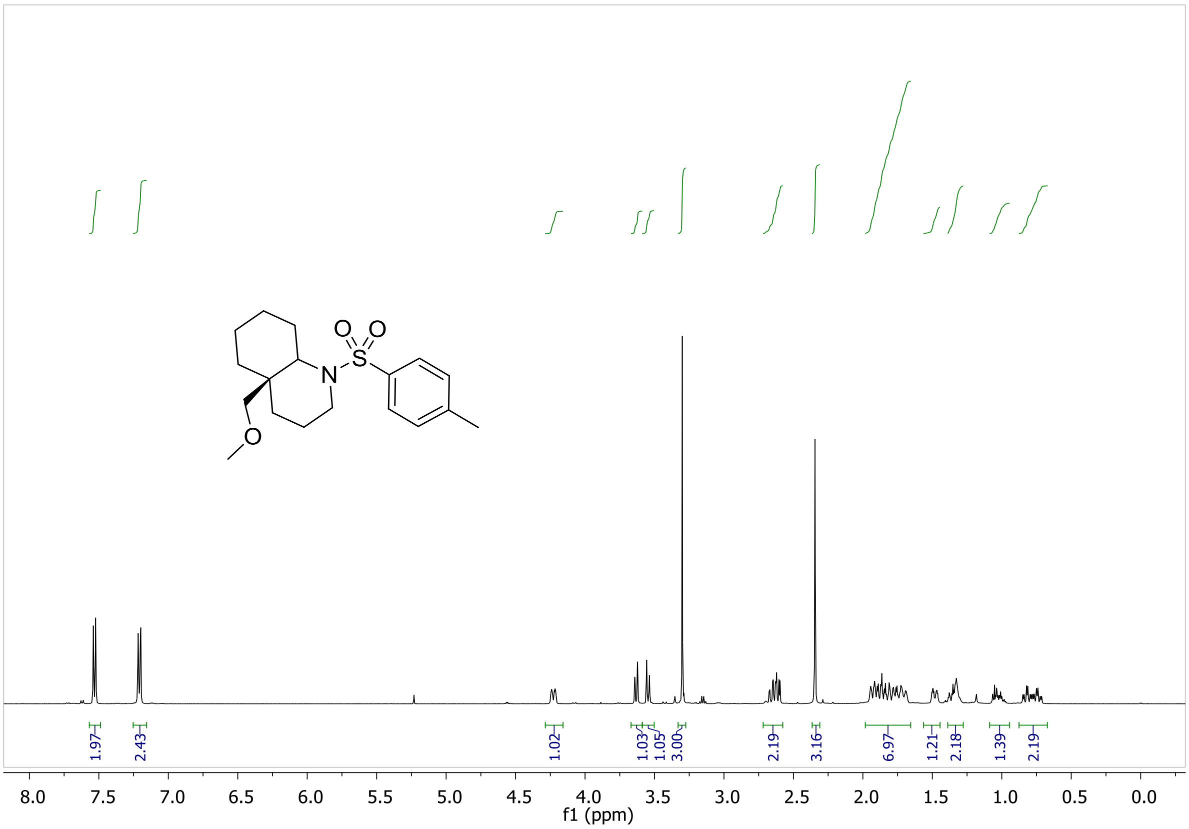
O
O
S
N
O
1.97
2.43
1.02
1.03
1.05
3.00
2.19
3.16
6.97
1.21
2.18
1.39
2.19
8.0 7.5 7.0 6.5 6.0 5.5 5.0 4.5 4.0 3.5 3.0 2.5 2.0 1.5 1.0 0.5 0.0
f1 (ppm)

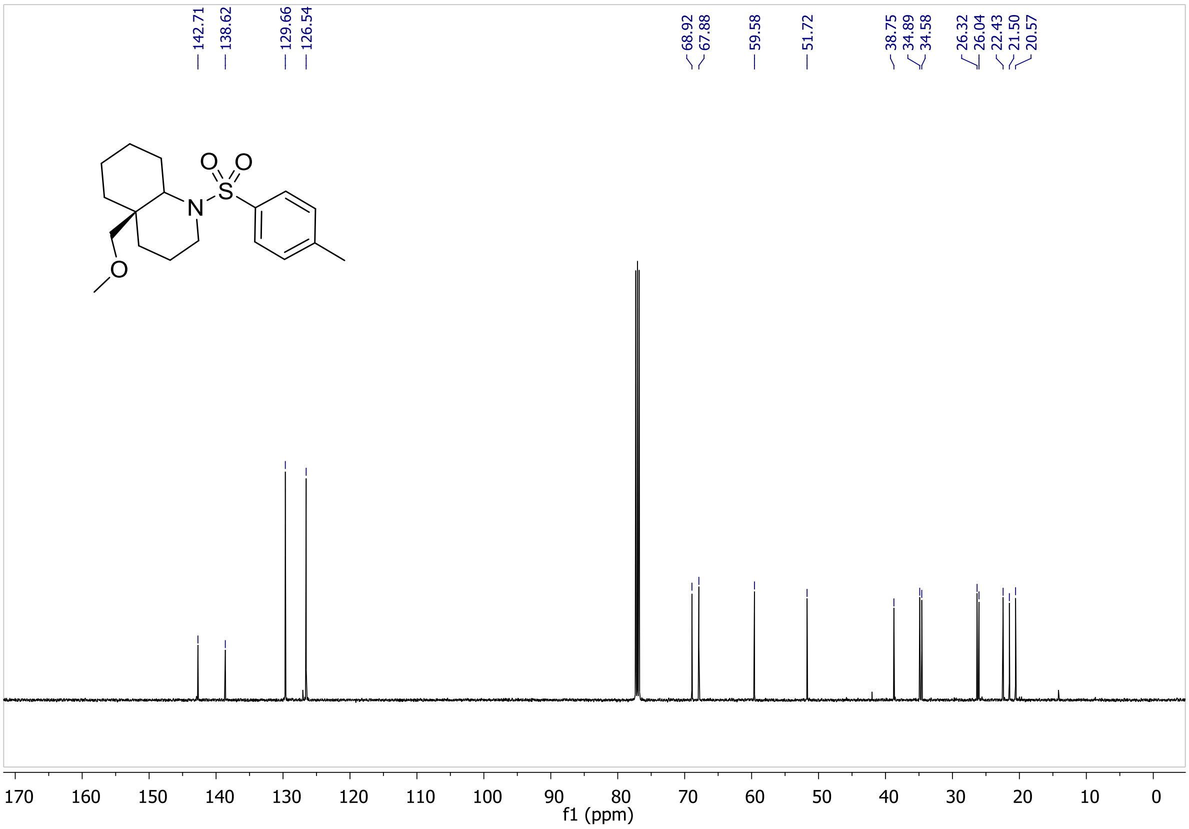
142.71
138.62
129.66
126.54
68.92
67.88
59.58
51.72
38.75
34.89
34.58
26.32
26.04
22.43
21.50
20.57
O
O
S
N
O
170 160 150 140 130 120 110 100 90 80 70 60 50 40 30 20 10 0
f1 (ppm)

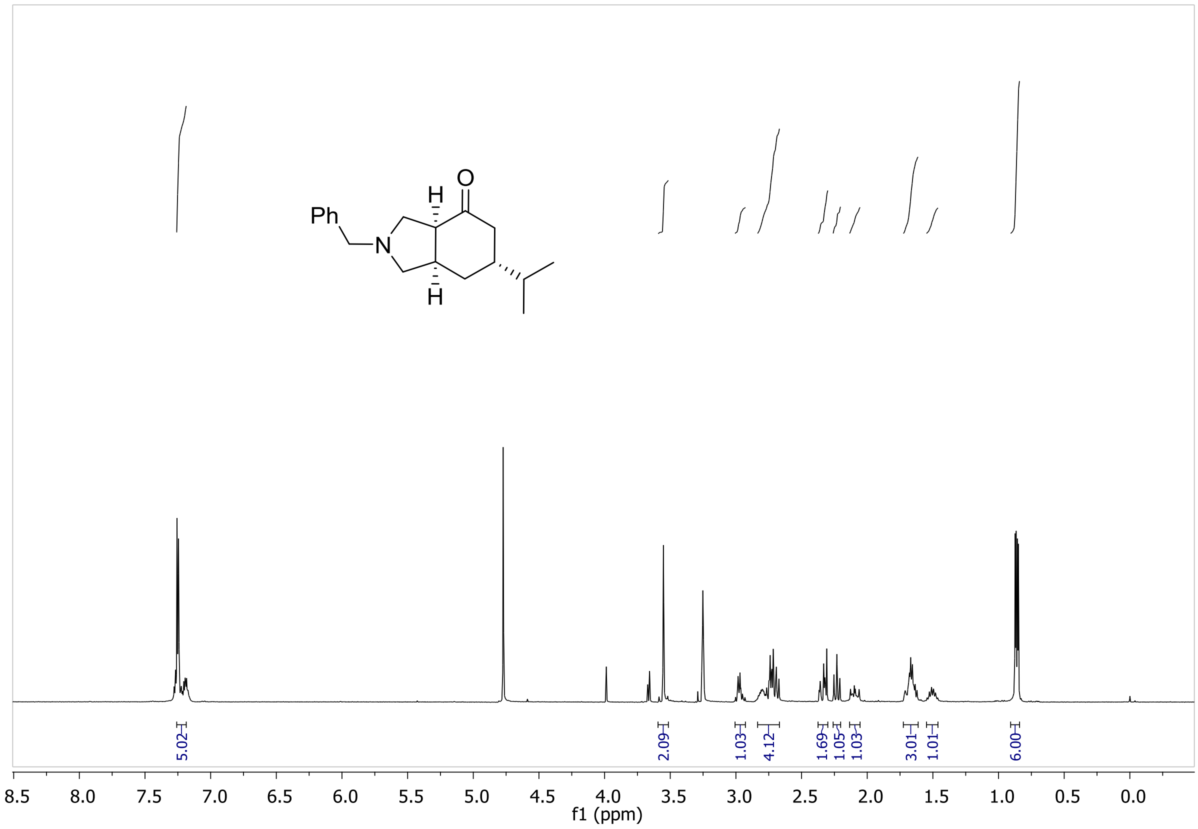
Ph
N
H
H
O
5.02
2.09
1.03
4.12
1.69
1.05
1.03
3.01
1.01
6.00
8.5 8.0 7.5 7.0 6.5 6.0 5.5 5.0 4.5 4.0 3.5 3.0 2.5 2.0 1.5 1.0 0.5 0.0
f1 (ppm)

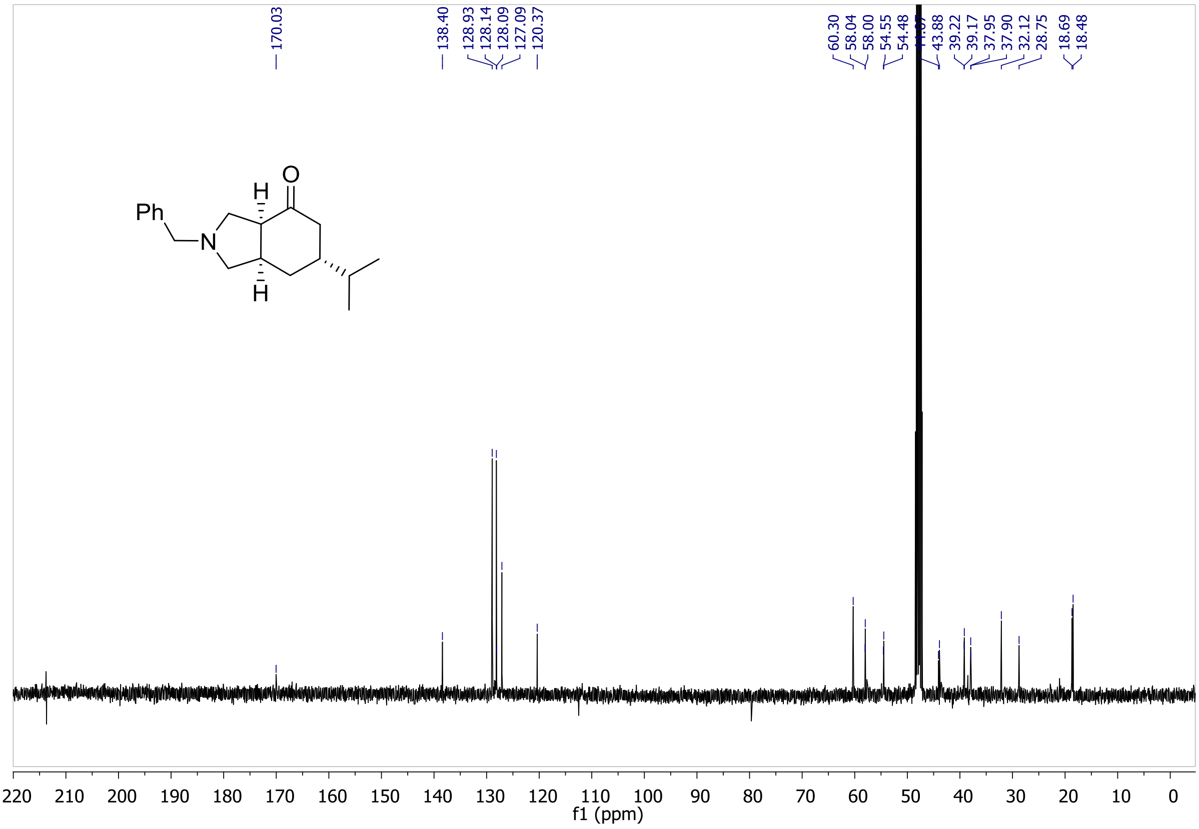
170.03
138.40
128.93
128.14
128.09
127.09
120.37
60.30
58.04
58.00
54.55
54.48
43.88
39.22
39.17
37.95
37.90
32.12
28.75
18.69
18.48
Ph
N
H
H
O
220 210 200 190 180 170 160 150 140 130 120 110 100 90 80 70 60 50 40 30 20 10 0
f1 (ppm)

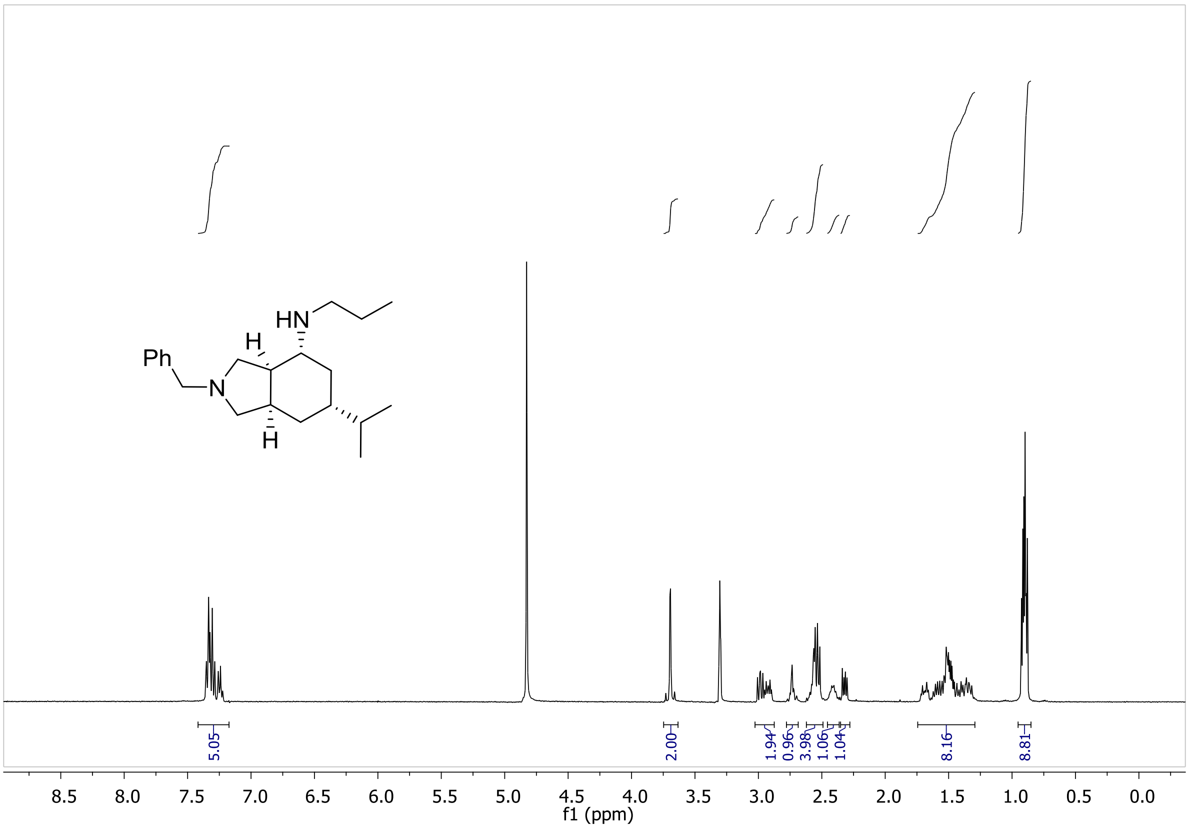
HN
H
Ph
N
H
5.05
2.00
1.94
0.96
3.98
1.06
1.04
8.16
8.81
8.5
8.0
7.5
7.0
6.5
6.0
5.5
5.0
4.5
4.0
3.5
3.0
2.5
2.0
1.5
1.0
0.5
0.0
f1 (ppm)

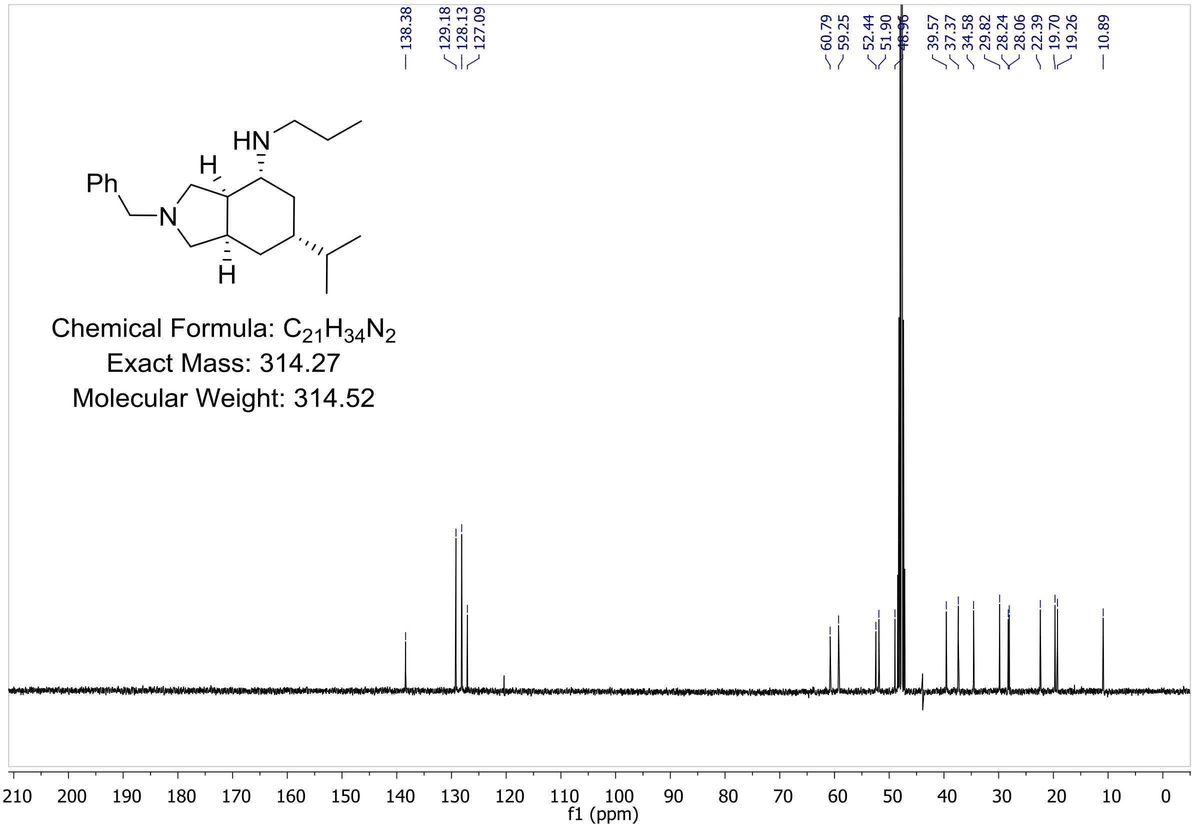
138.38
129.18
128.13
127.09
60.79
59.25
52.44
51.90
48.96
39.57
37.37
34.58
29.82
28.24
28.06
22.39
19.70
19.26
10.89
HN
H
Ph
N
H
Chemical Formula: $C_{21}H_{34}N_2$
Exact Mass: 314.27
Molecular Weight: 314.52
210
200
190
180
170
160
150
140
130
120
110
100
90
80
70
60
50
40
30
20
10
0
f1 (ppm)

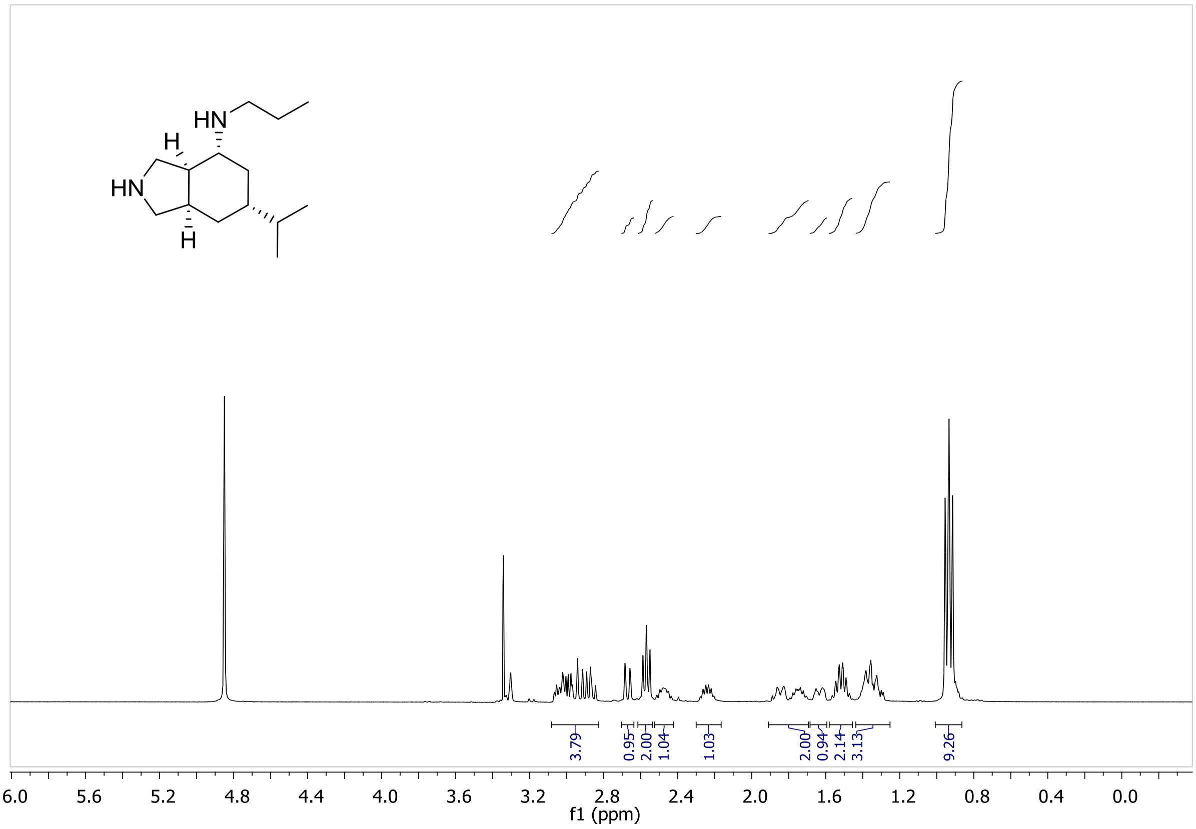
HN
H
HN
H
3.79
0.95
2.00
1.04
1.03
2.00
0.94
2.14
3.13
9.26
6.0 5.6 5.2 4.8 4.4 4.0 3.6 3.2 2.8 2.4 2.0 1.6 1.2 0.8 0.4 0.0
f1 (ppm)

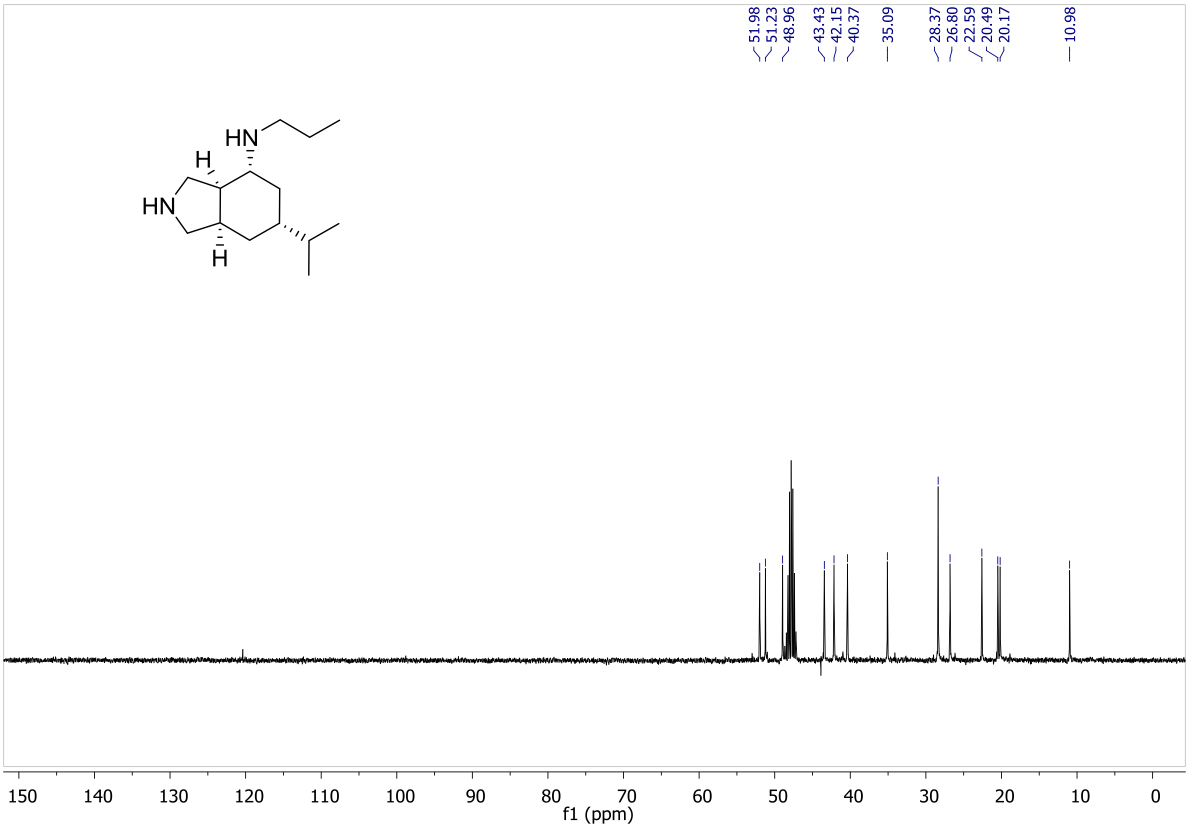
51.98
51.23
48.96
43.43
42.15
40.37
35.09
28.37
26.80
22.59
20.49
20.17
10.98
HN
H
HN
H
150 140 130 120 110 100 90 80 70 60 50 40 30 20 10 0
f1 (ppm)

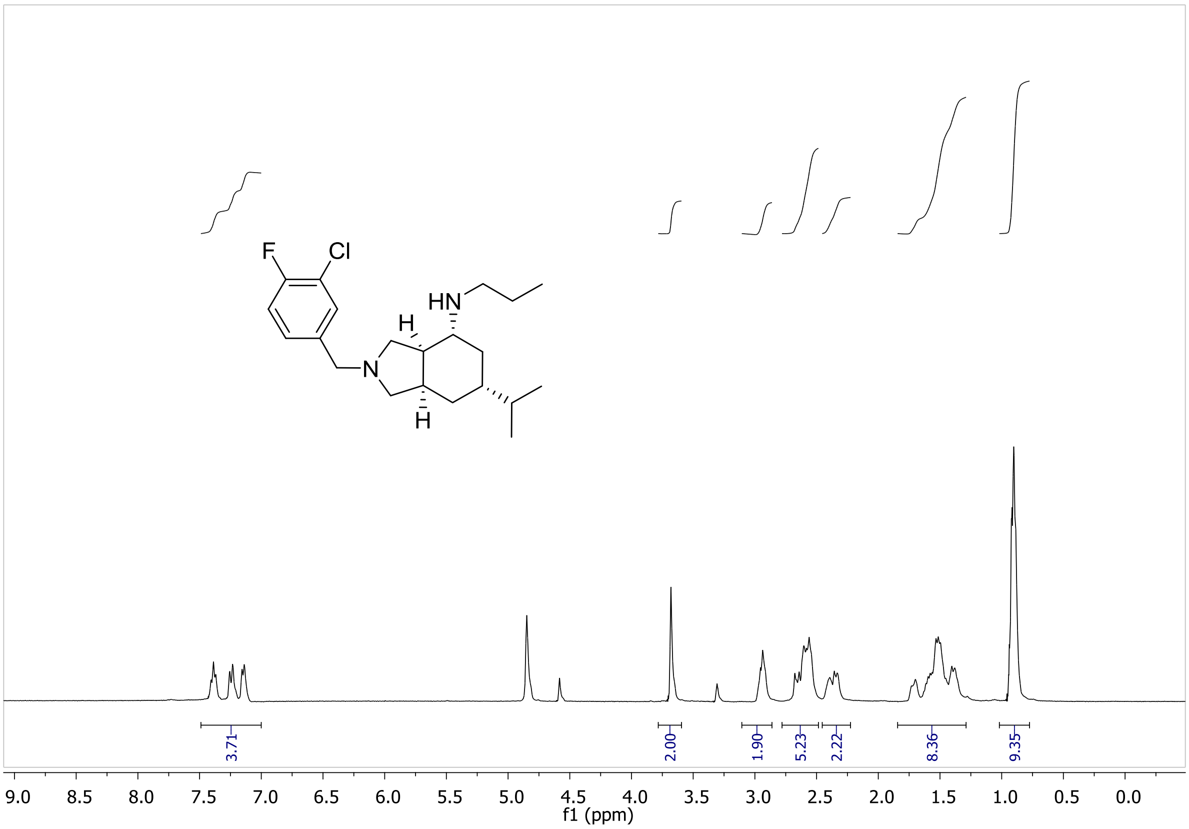
F
Cl
HN
H
N
H
3.71
2.00
1.90
5.23
2.22
8.36
9.35
9.0 8.5 8.0 7.5 7.0 6.5 6.0 5.5 5.0 4.5 4.0 3.5 3.0 2.5 2.0 1.5 1.0 0.5 0.0
f1 (ppm)

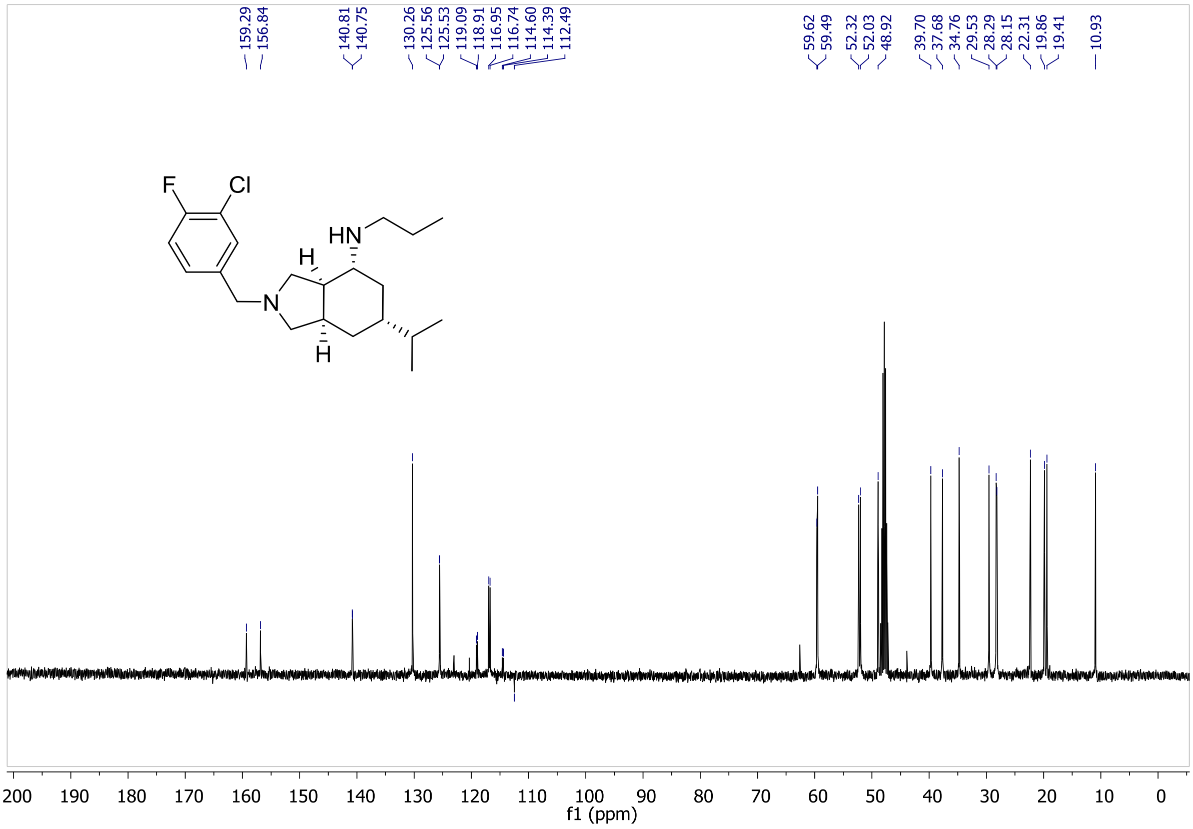
159.29
156.84
140.81
140.75
130.26
125.56
125.53
119.09
118.91
116.95
116.74
114.60
114.39
112.49
59.62
59.49
52.32
52.03
48.92
39.70
37.68
34.76
29.53
28.29
28.15
22.31
19.86
19.41
10.93
F
Cl
HN
H
N
H
200 190 180 170 160 150 140 130 120 110 100 90 80 70 60 50 40 30 20 10 0
f1 (ppm)

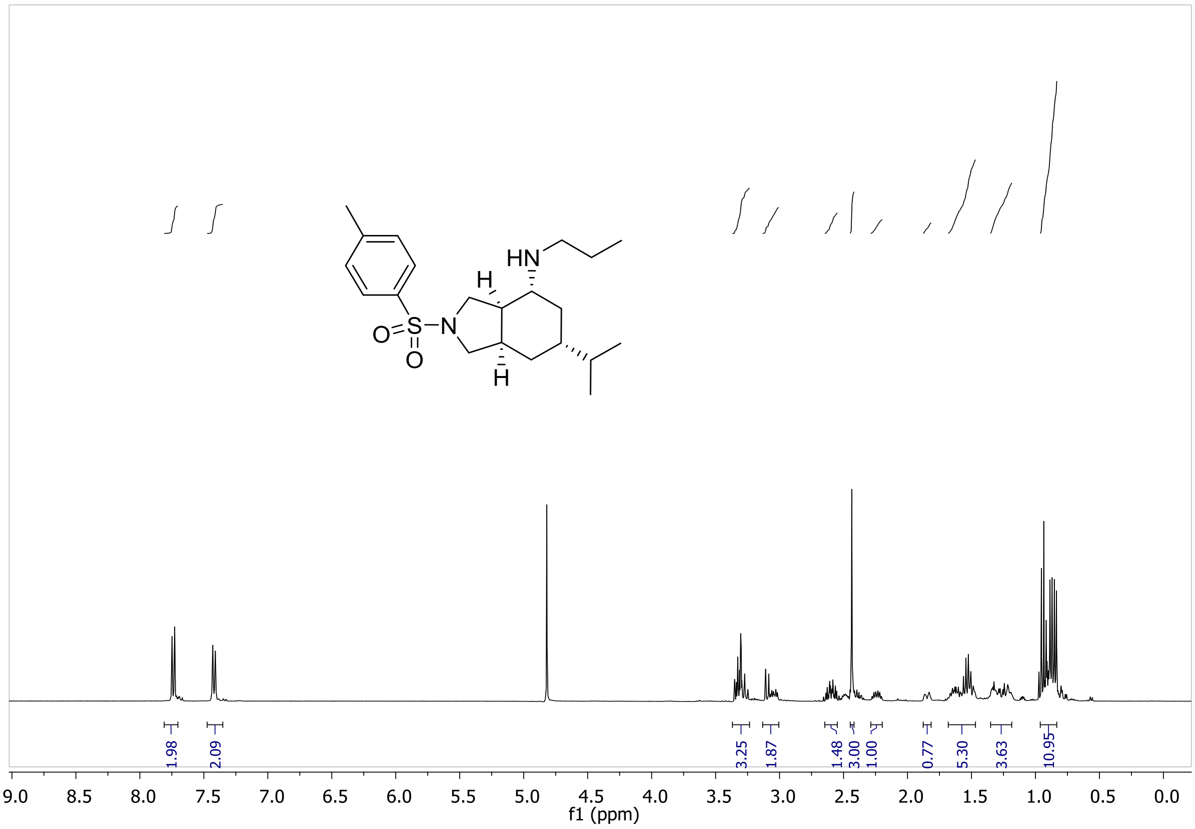
HN
H
O=S−N
O
H
1.98
2.09
3.25
1.87
1.48
3.00
1.00
0.77
5.30
3.63
10.95
9.0 8.5 8.0 7.5 7.0 6.5 6.0 5.5 5.0 4.5 4.0 3.5 3.0 2.5 2.0 1.5 1.0 0.5 0.0
f1 (ppm)

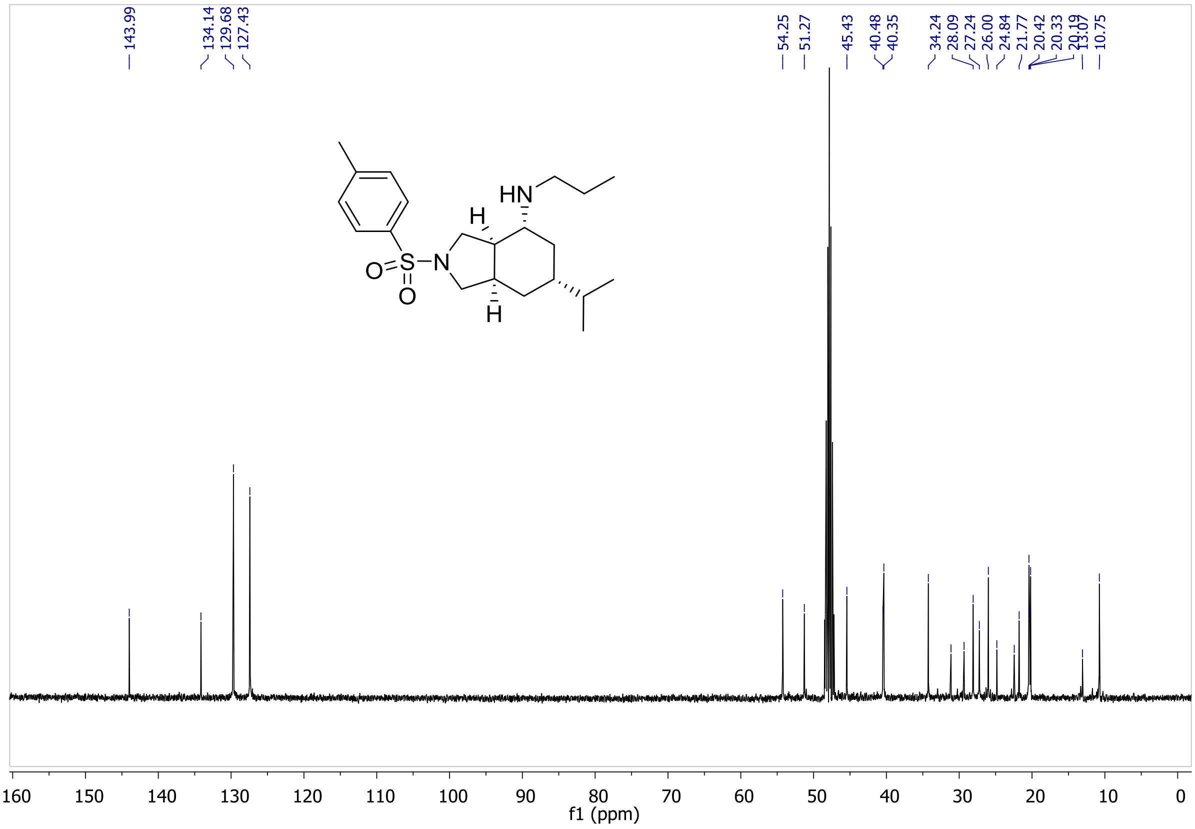
143.99
134.14
129.68
127.43
54.25
51.27
45.43
40.48
40.35
34.24
28.09
27.24
26.00
24.84
21.77
20.42
20.33
20.19
13.07
10.75
HN
H
O=S−N
O
H
160 150 140 130 120 110 100 90 80 70 60 50 40 30 20 10 0
f1 (ppm)

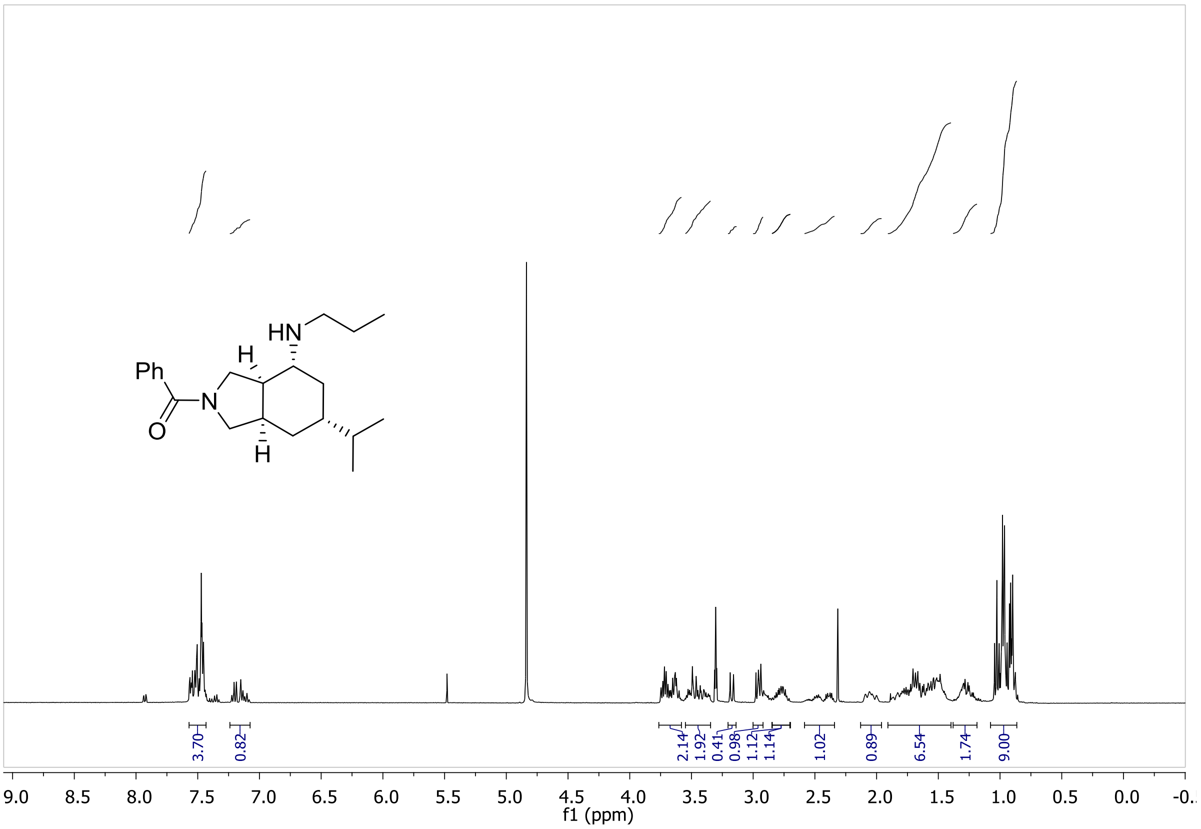
HN
H
Ph
N
O
H
3.70
0.82
2.14
1.92
0.41
0.98
1.12
1.14
1.02
0.89
6.54
1.74
9.00
9.0 8.5 8.0 7.5 7.0 6.5 6.0 5.5 5.0 4.5 4.0 3.5 3.0 2.5 2.0 1.5 1.0 0.5 0.0
f1 (ppm)

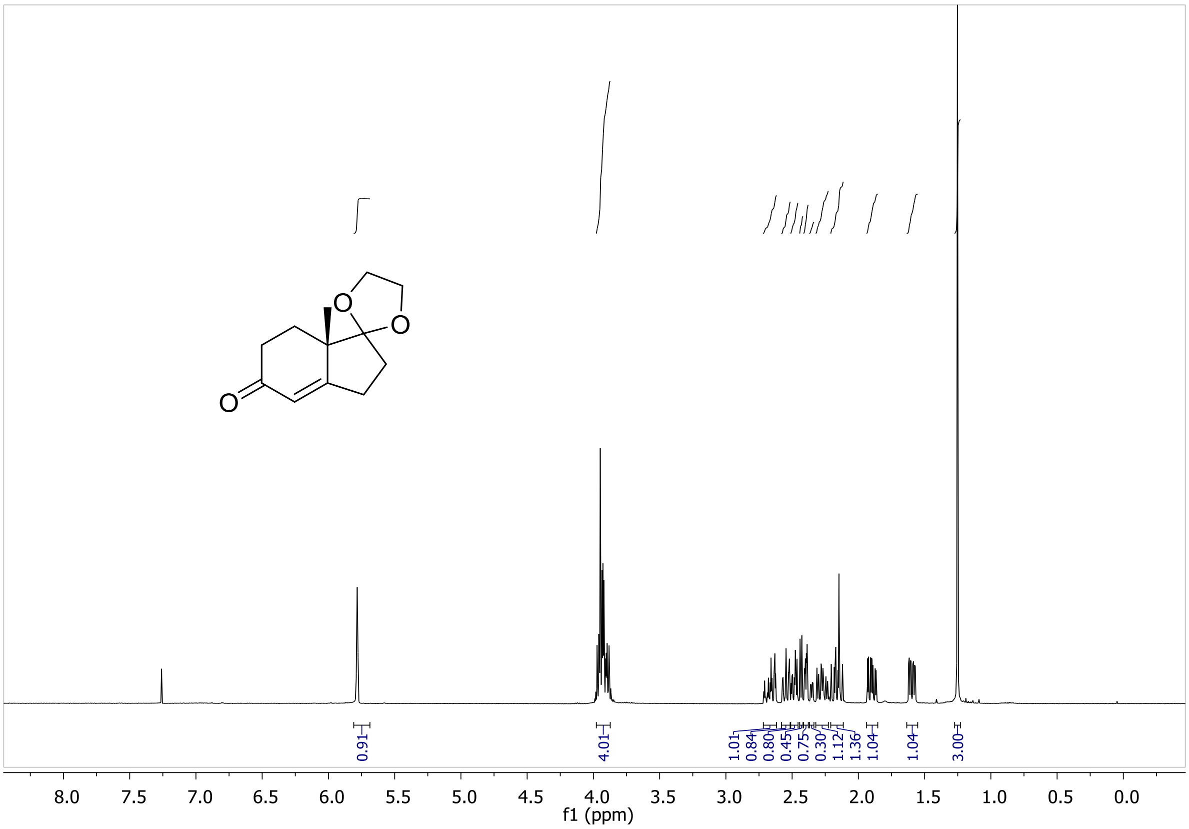
O
O
O
0.91
4.01
1.01
0.84
0.80
0.45
0.75
0.30
1.12
1.36
1.04
1.04
3.00
8.0 7.5 7.0 6.5 6.0 5.5 5.0 4.5 4.0 3.5 3.0 2.5 2.0 1.5 1.0 0.5 0.0
f1 (ppm)

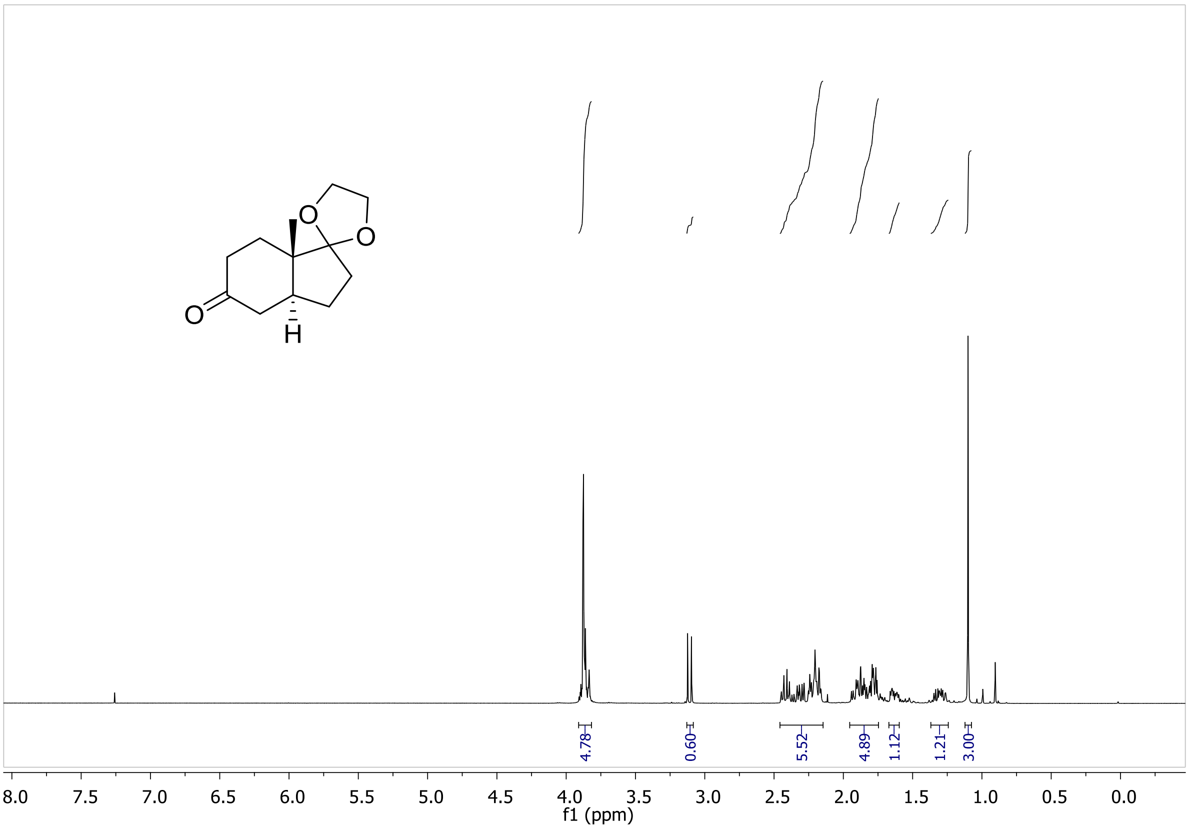
O
O
O
H
4.78
0.60
5.52
4.89
1.12
1.21
3.00
8.0 7.5 7.0 6.5 6.0 5.5 5.0 4.5 4.0 3.5 3.0 2.5 2.0 1.5 1.0 0.5 0.0
f1 (ppm)

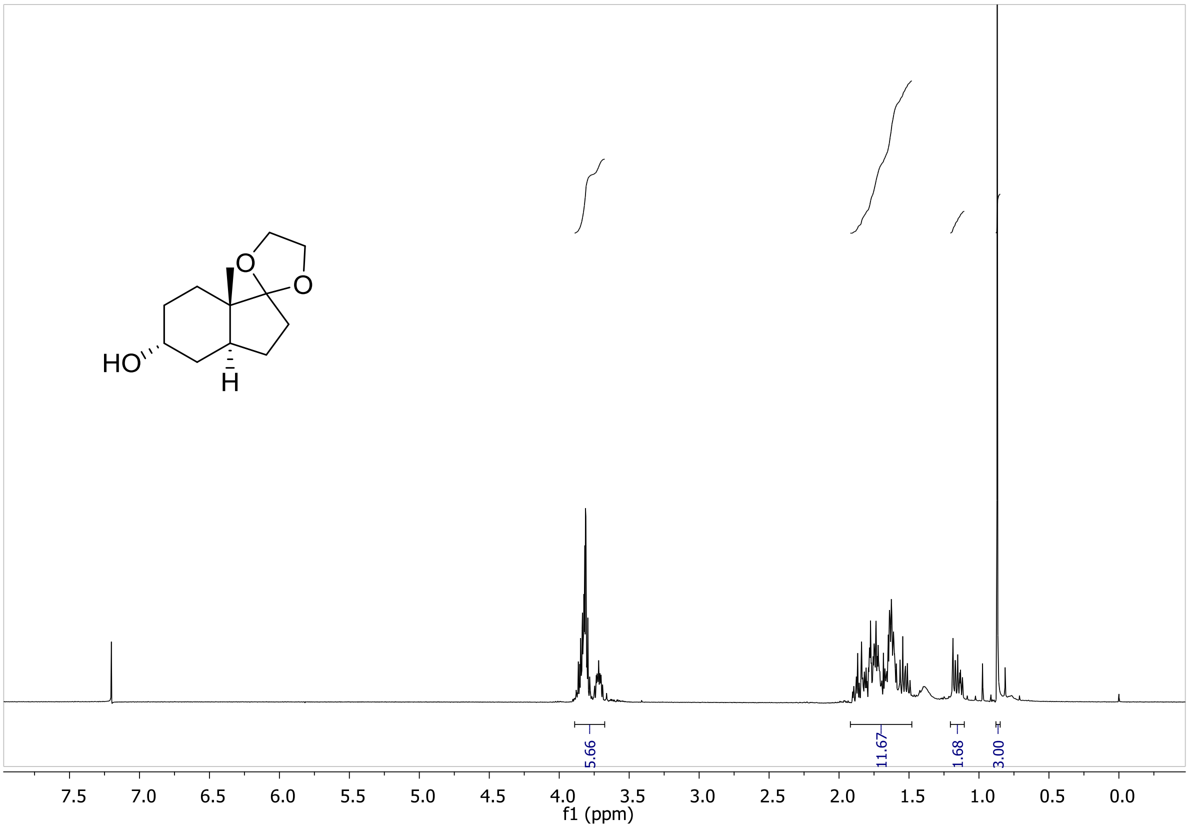
O
O
HO
H
5.66
11.67
1.68
3.00
7.5 7.0 6.5 6.0 5.5 5.0 4.5 4.0 3.5 3.0 2.5 2.0 1.5 1.0 0.5 0.0
f1 (ppm)

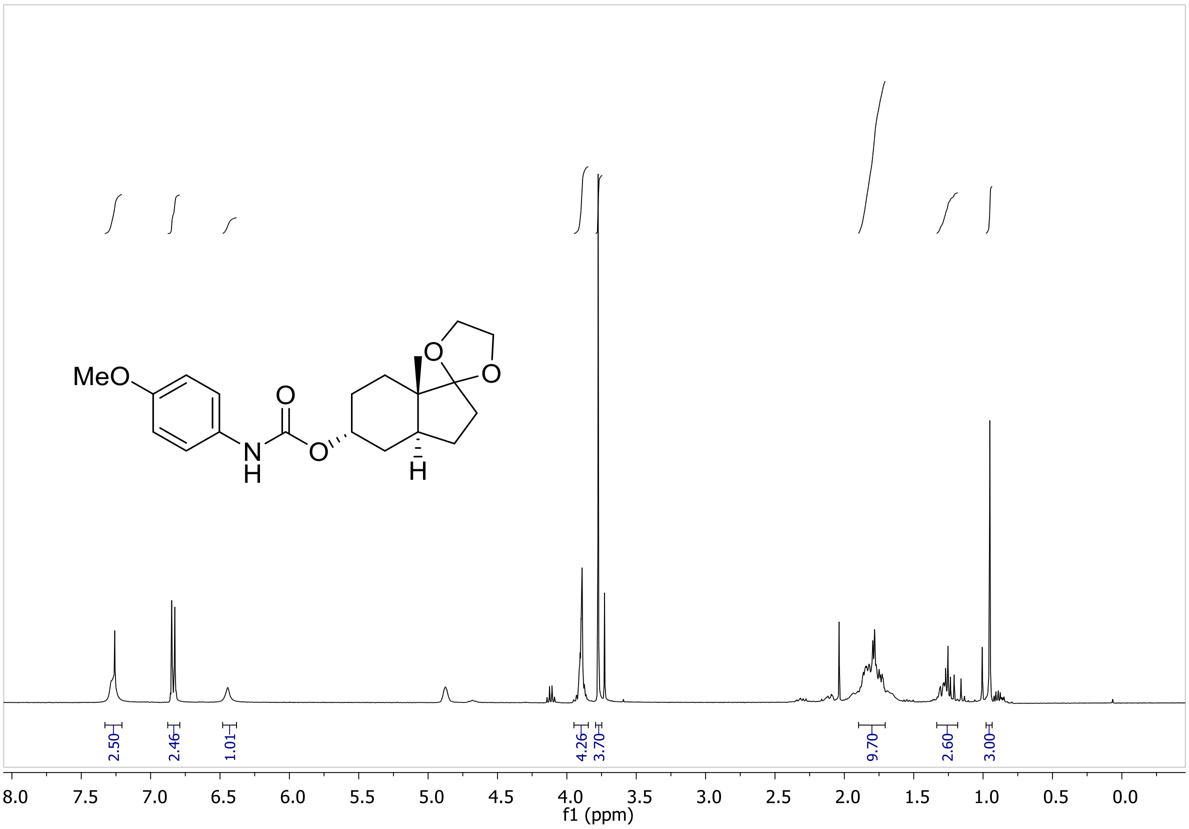
MeO
O
N
H
O
O
O
H
2.50
2.46
1.01
4.26
3.70
9.70
2.60
3.00
8.0 7.5 7.0 6.5 6.0 5.5 5.0 4.5 4.0 3.5 3.0 2.5 2.0 1.5 1.0 0.5 0.0
f1 (ppm)

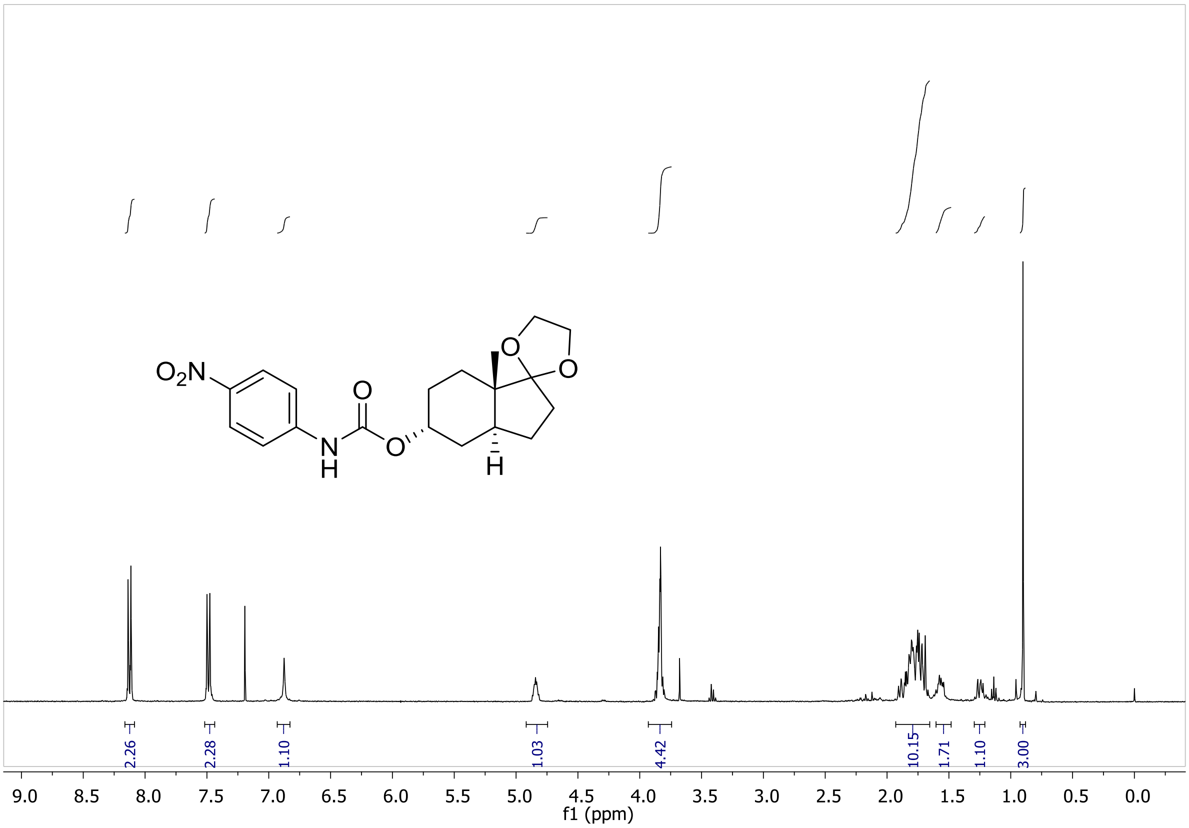
O_2N
O
N
H
O
O
O
H
2.26
2.28
1.10
1.03
4.42
10.15
1.71
1.10
3.00
9.0 8.5 8.0 7.5 7.0 6.5 6.0 5.5 5.0 4.5 4.0 3.5 3.0 2.5 2.0 1.5 1.0 0.5 0.0
f1 (ppm)

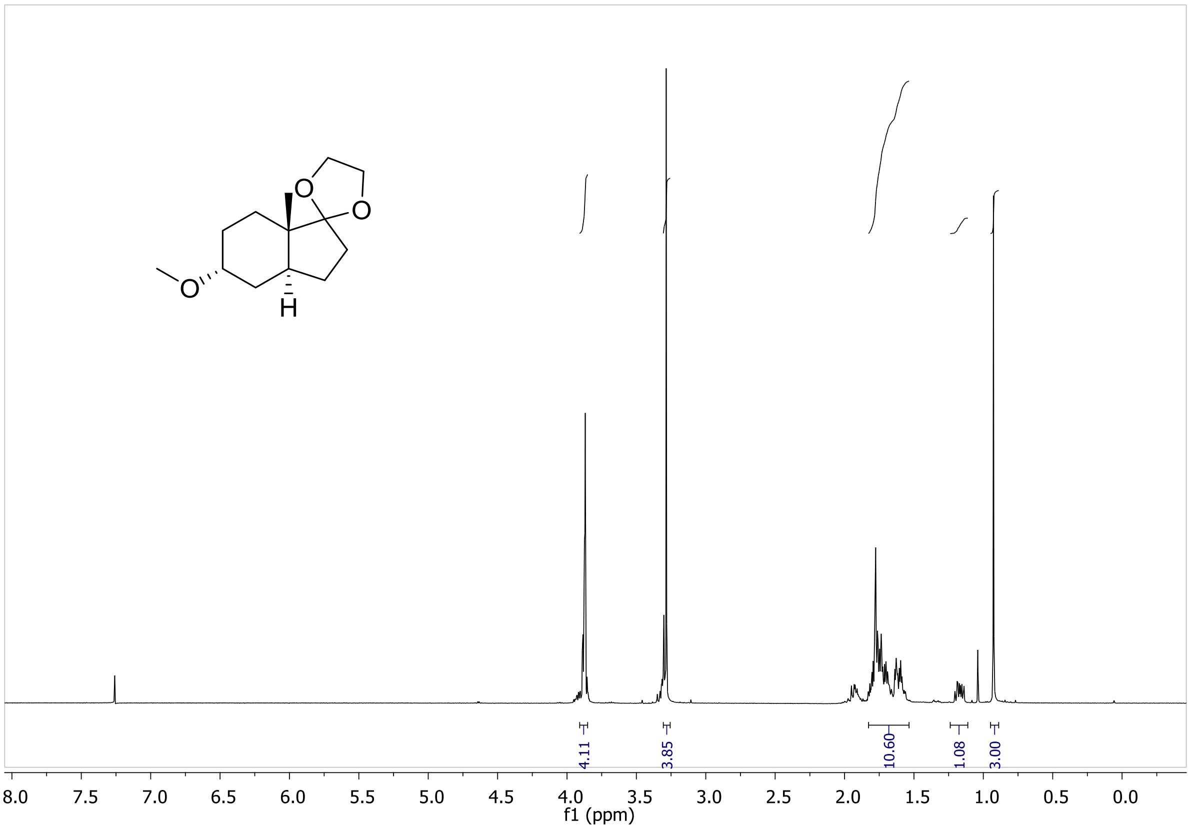
O
O
O
H
4.11
3.85
10.60
1.08
3.00
8.0 7.5 7.0 6.5 6.0 5.5 5.0 4.5 4.0 3.5 3.0 2.5 2.0 1.5 1.0 0.5 0.0
f1 (ppm)

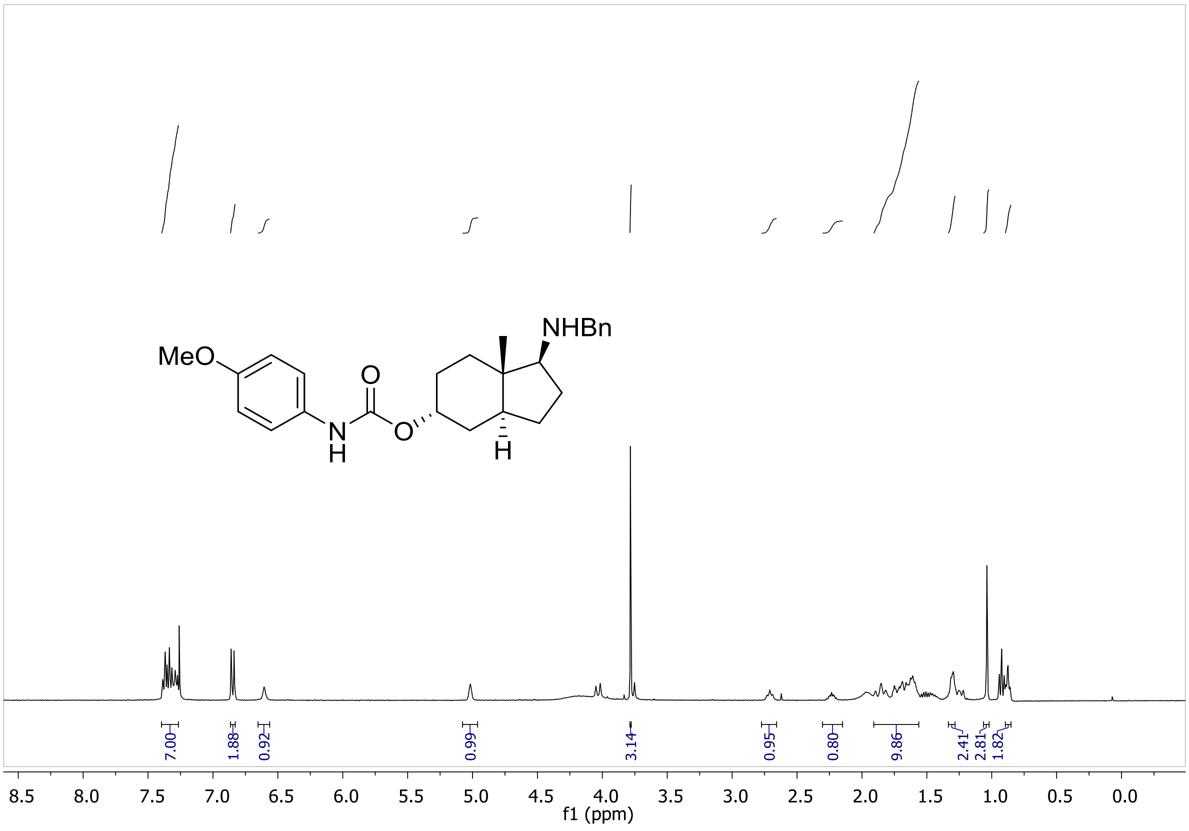
NHBn
MeO
O
N
H
O
H
7.00
1.88
0.92
0.99
3.14
0.95
0.80
9.86
2.41
2.81
1.82
8.5 8.0 7.5 7.0 6.5 6.0 5.5 5.0 4.5 4.0 3.5 3.0 2.5 2.0 1.5 1.0 0.5 0.0
f1 (ppm)

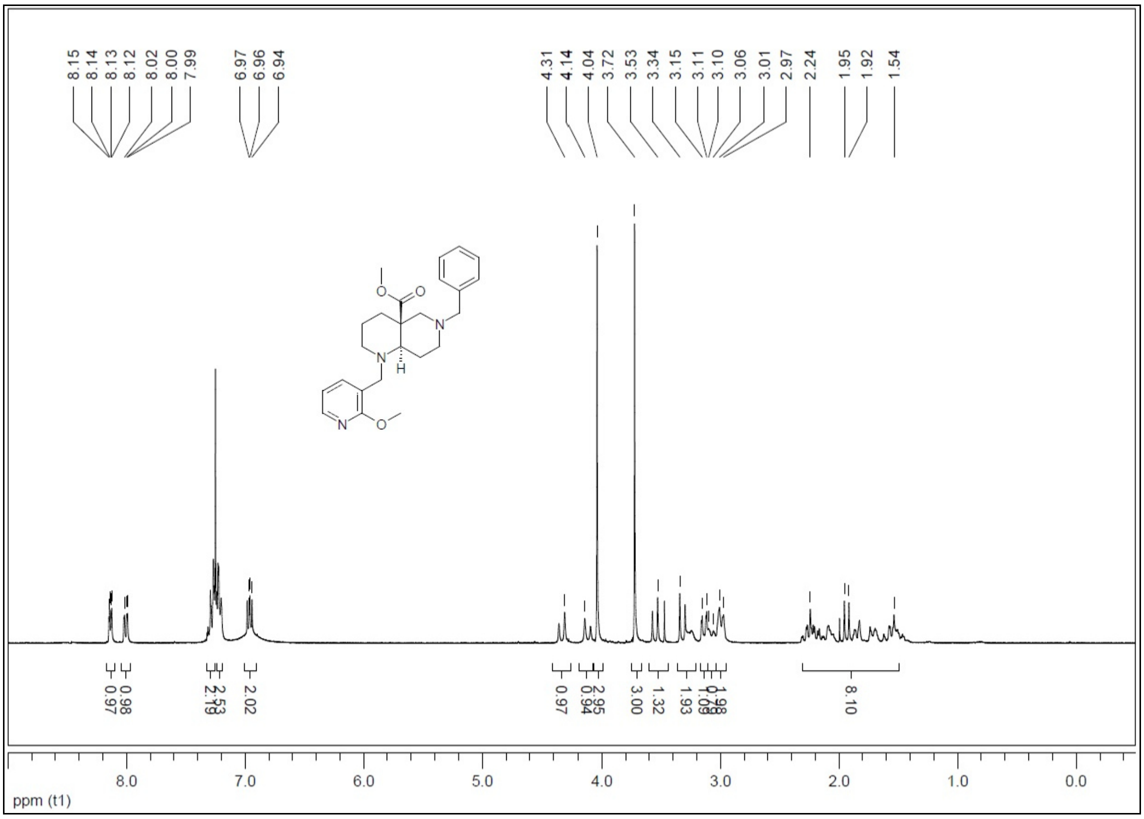

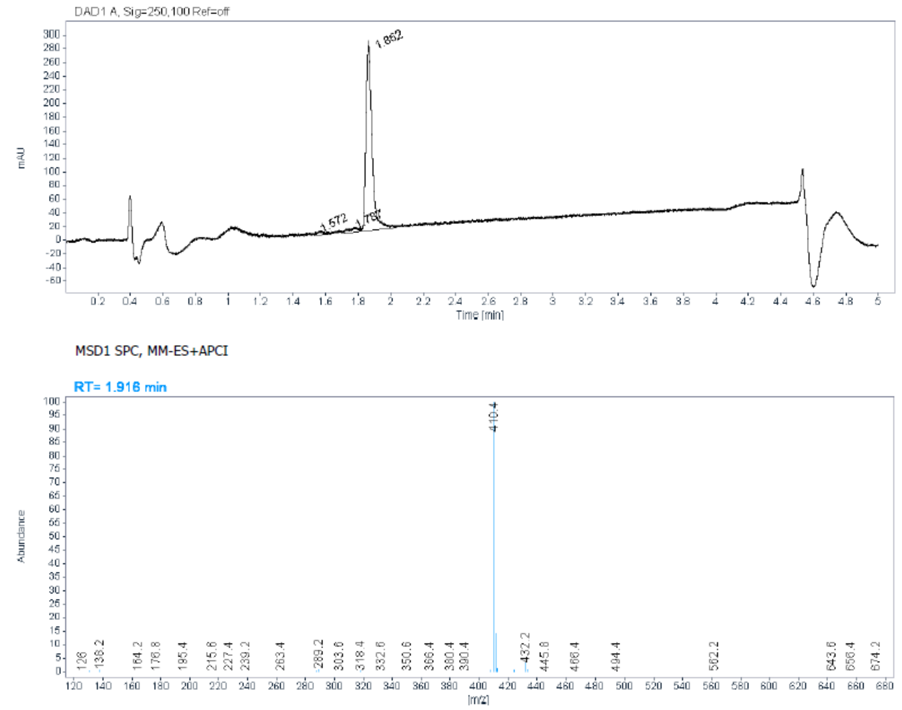

Signal: DAD1 A, Sig=250,100 Ref=off

RT [min]	Peak Area Percent
1.572	2.07
1.787	3.76
1.862	94.17

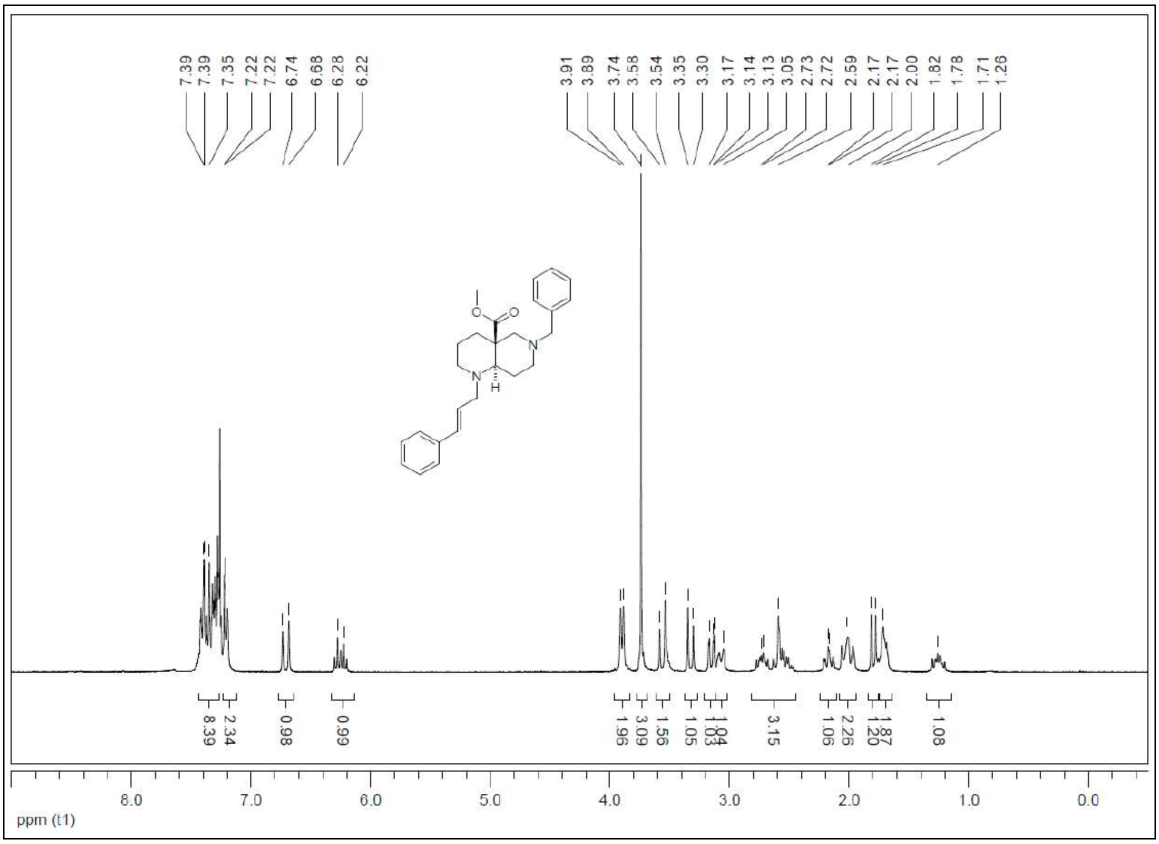

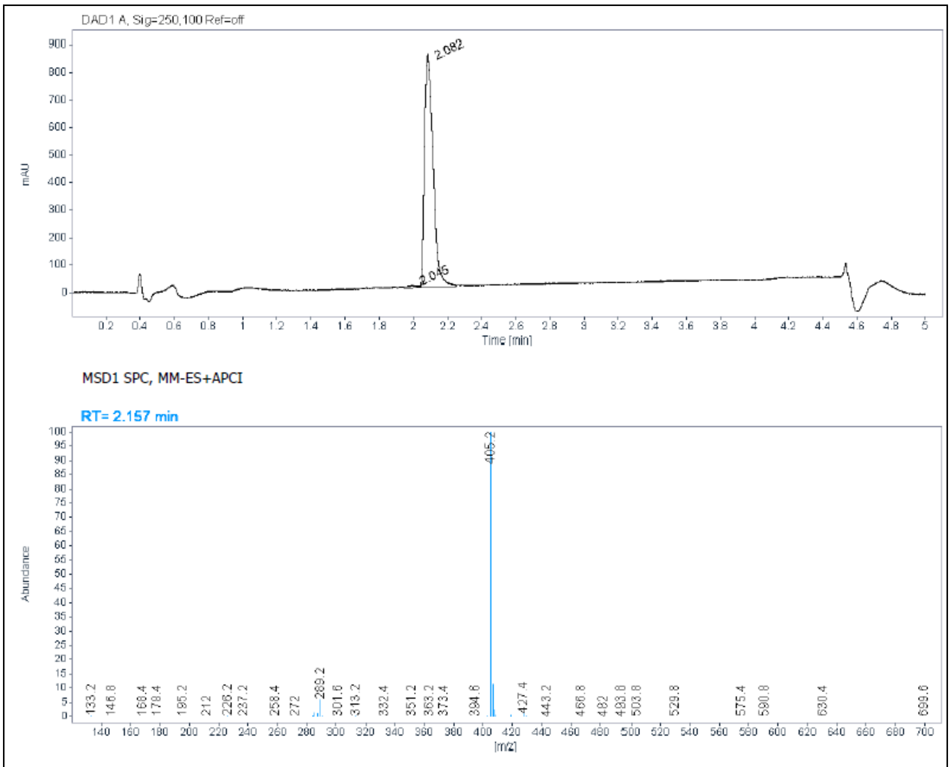

Signal: DAD1 A, Sig=250,100 Ref=off

RT [min]	Peak Area Percent
2.045	1.44
2.082	98.56

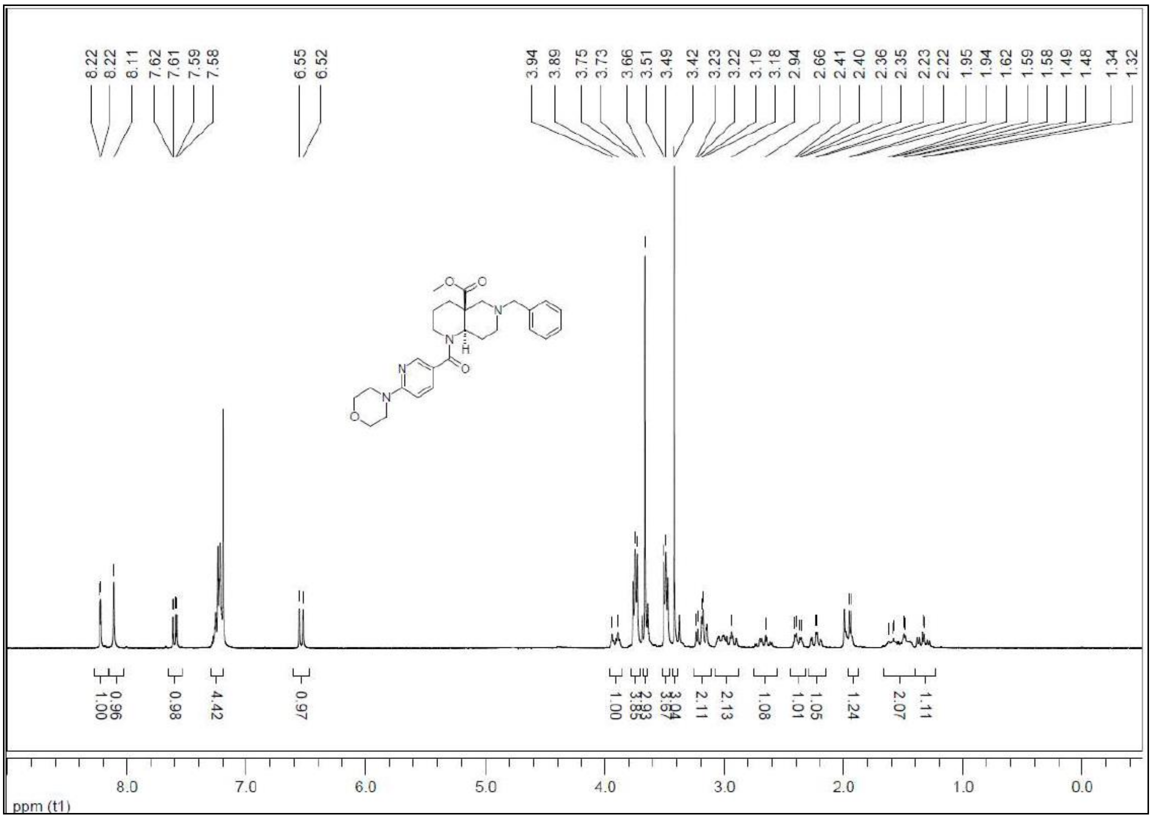

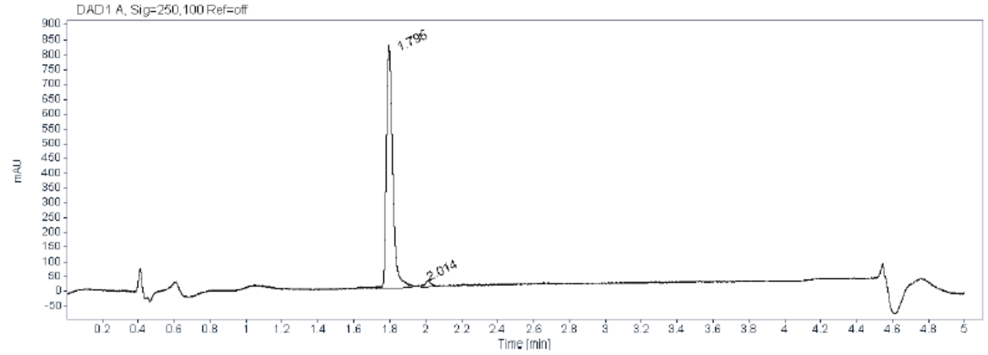

MSD1 SPC, MM-ES+APCI

RT= 1.89 min

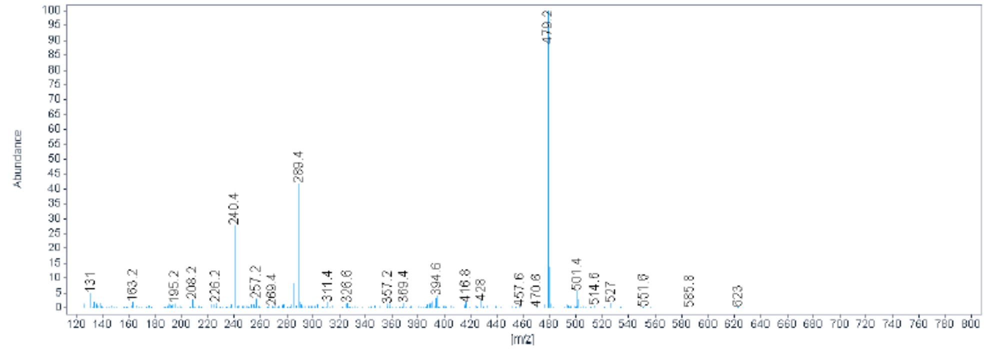

Signal: DAD1 A, Sig=250,100 Ref=off

RT [min]	Peak Area Percent
1.796	97.48
2.014	2.52

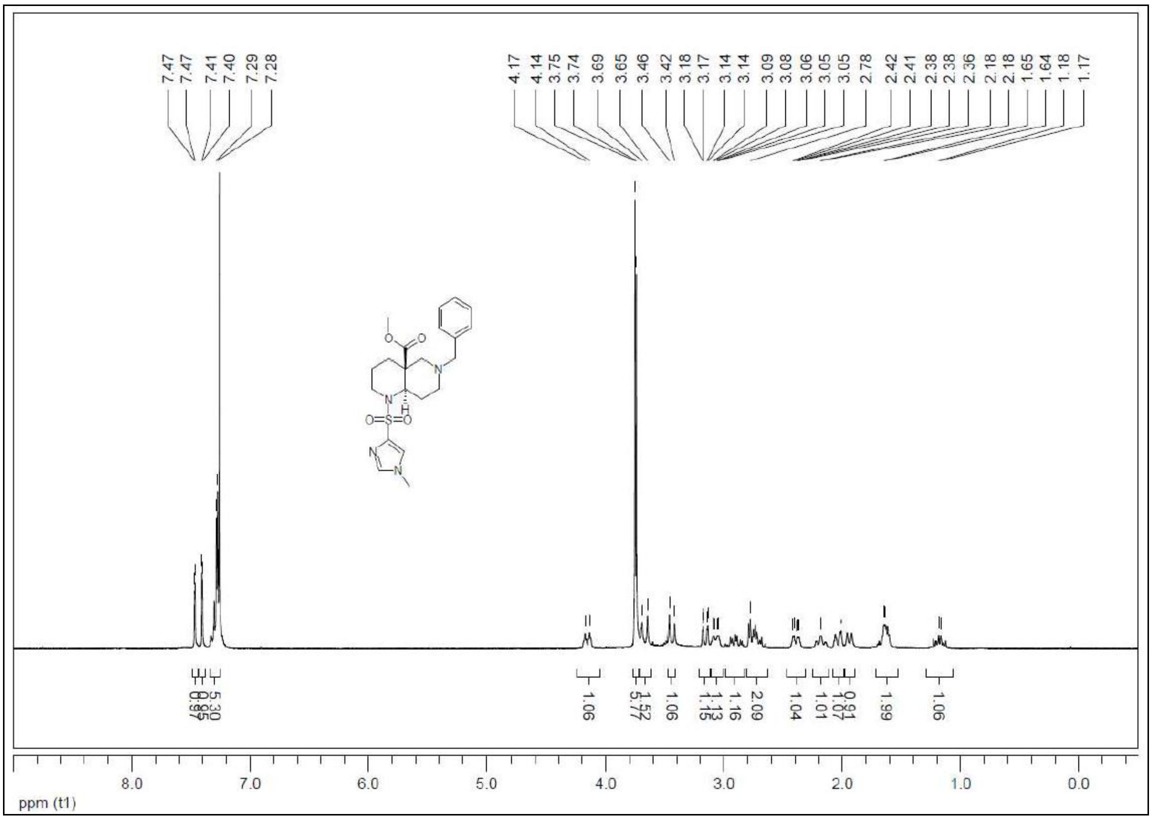

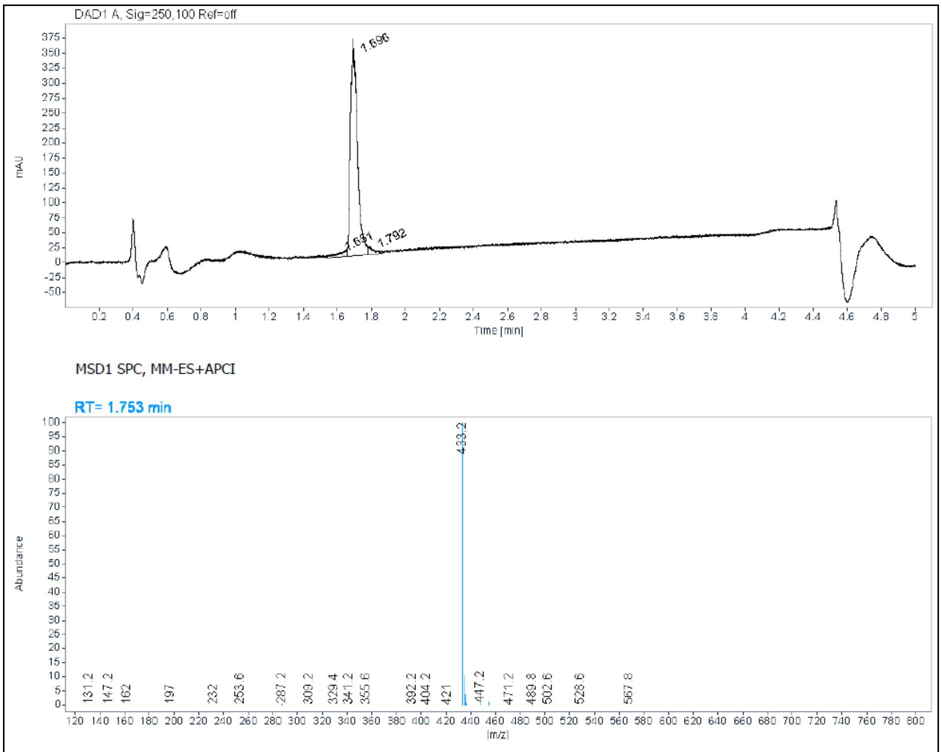

Signal: DAD1 A, Sig=250,100 Ref=off

RT [min]	Peak Area Percent
1.651	3.49
1.696	93.45
1.792	3.07

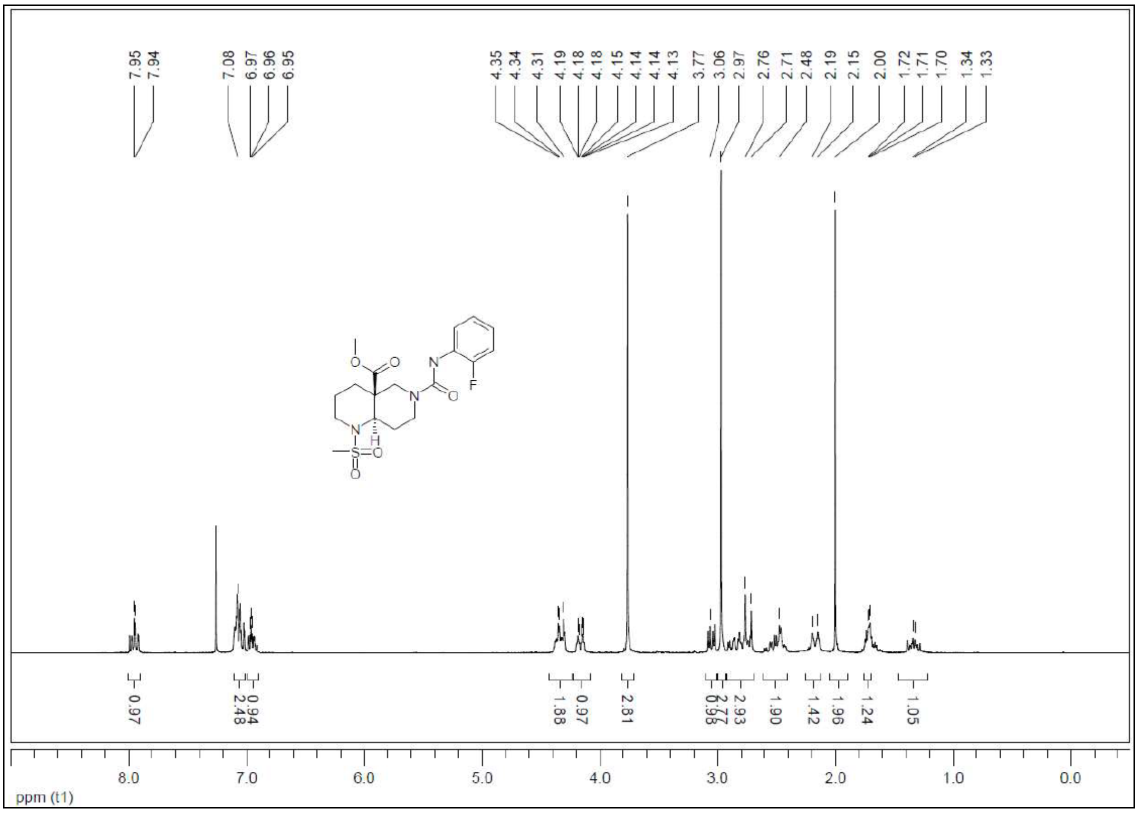

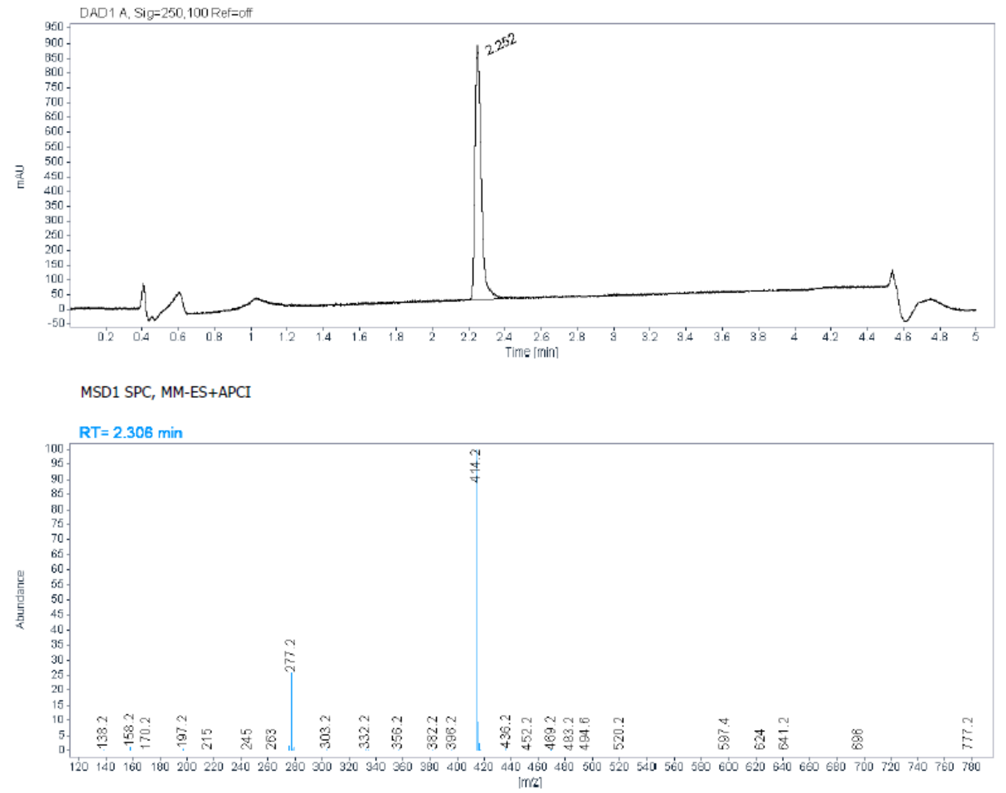

Signal: DAD1 A, Sig=250,100 Ref=off

RT [min]	Peak Area Percent
2.252	100.00

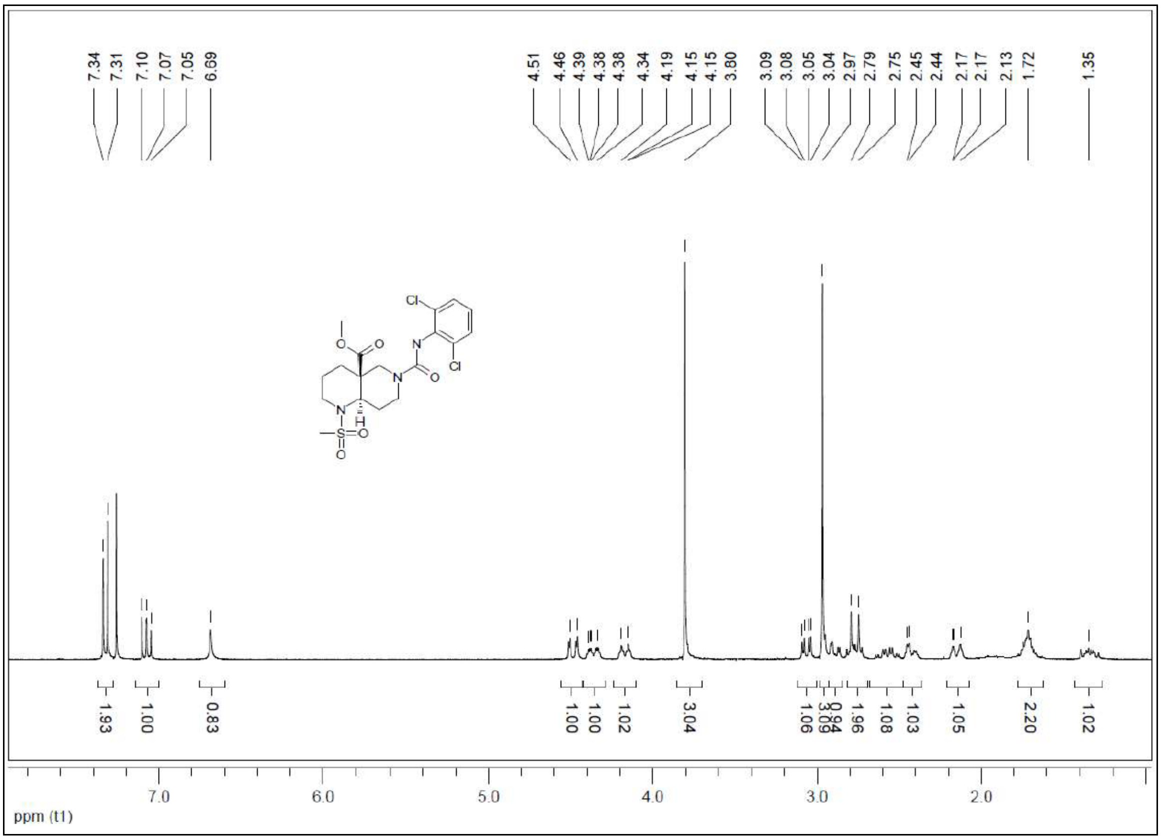

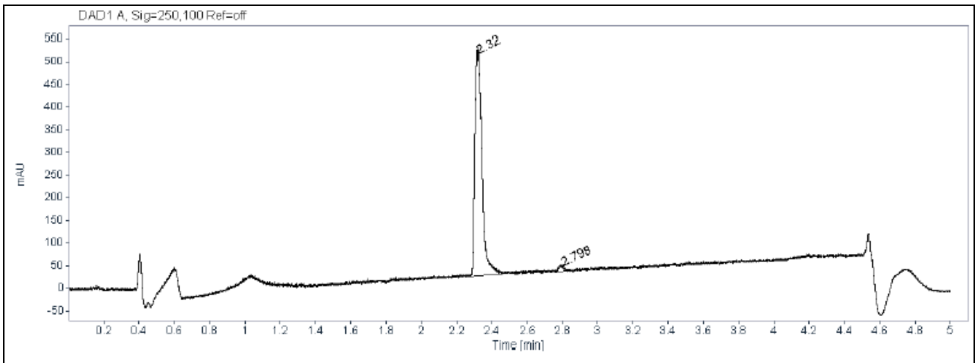

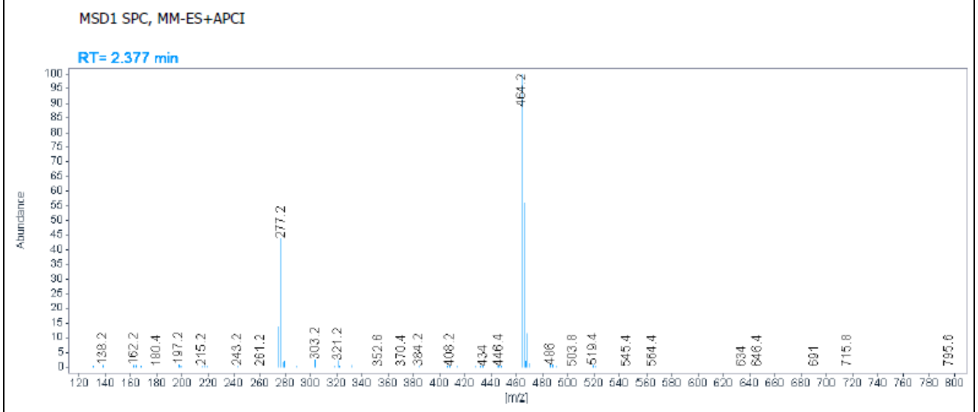

Signal: DAD1 A, Sig=250,100 Ref=off

RT [min]	Peak Area Percent
2.320	97.90
2.798	2.10

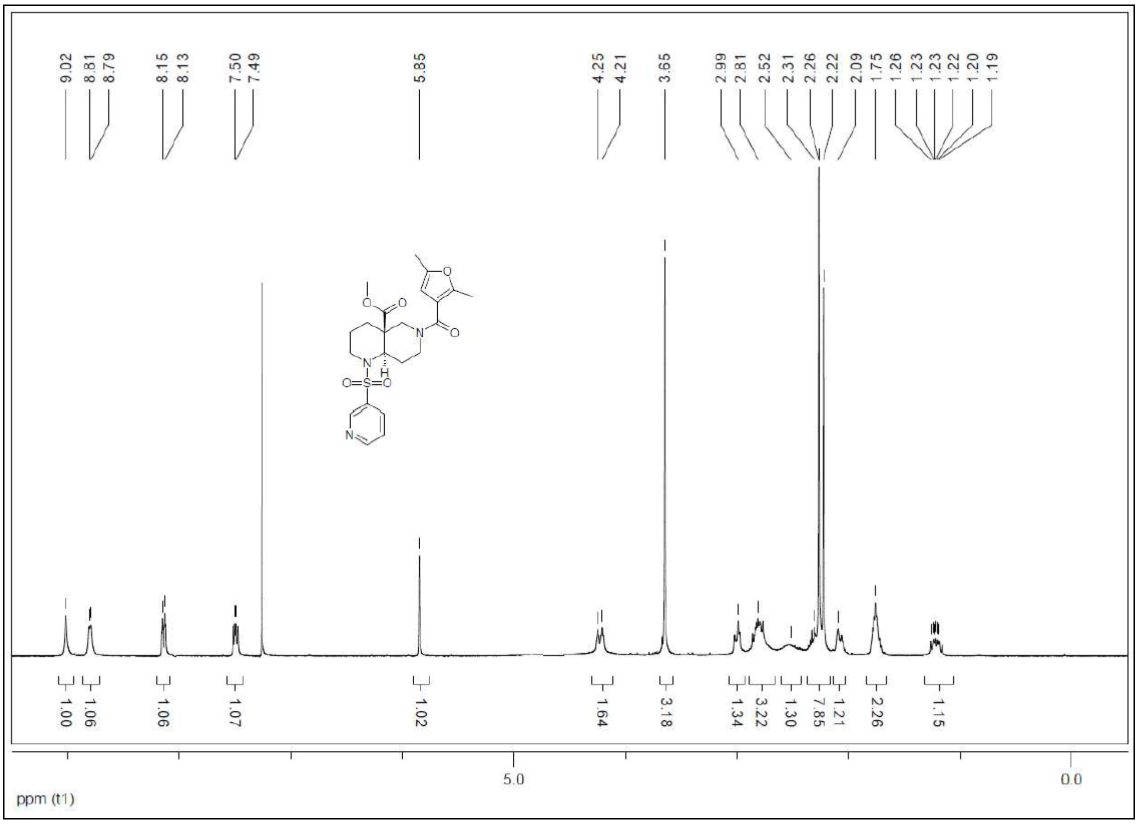

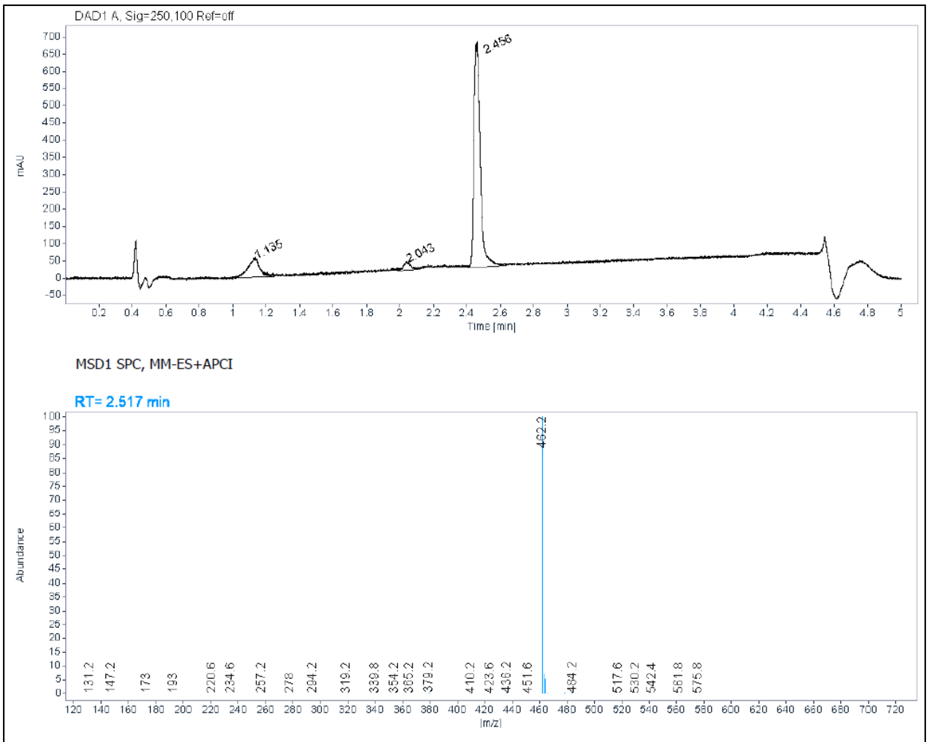

Signal: DAD1 A, Sig=250,100 Ref=off

RT [min]	Peak Area Percent
1.135	13.18
2.043	3.39
2.456	83.43

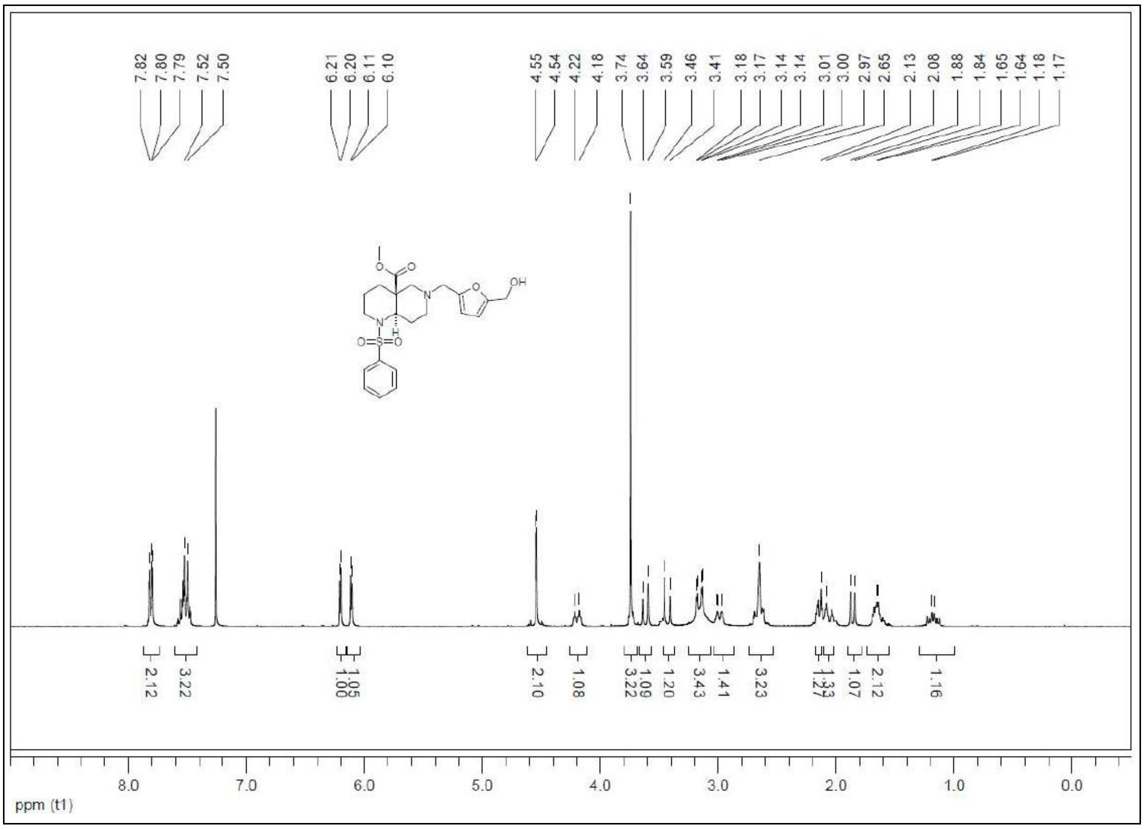

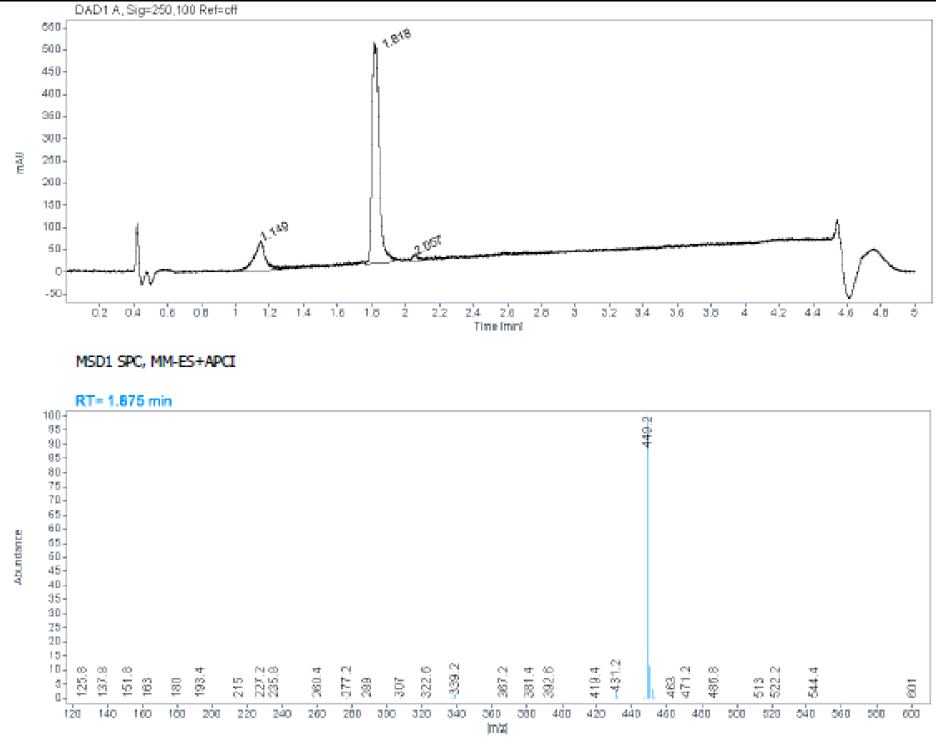

Signal: DAD1 A, Sig=250,100 Ref=off

RT [min]	Peak Area Percent
1.149	16.82
1.818	81.30
2.057	1.88

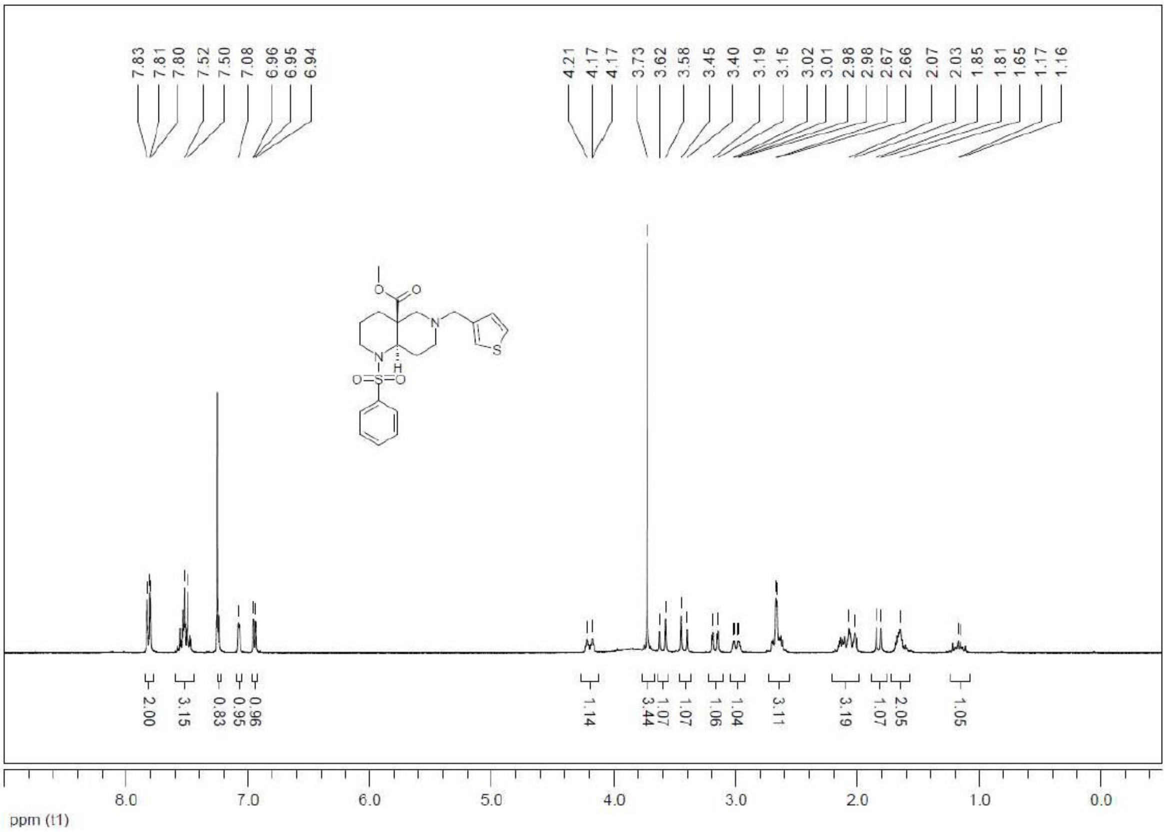

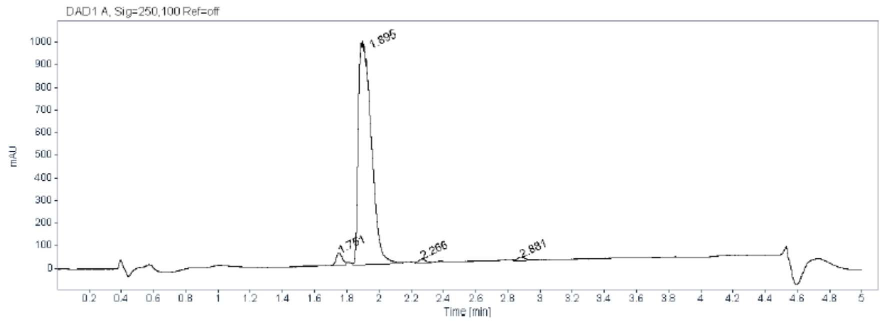

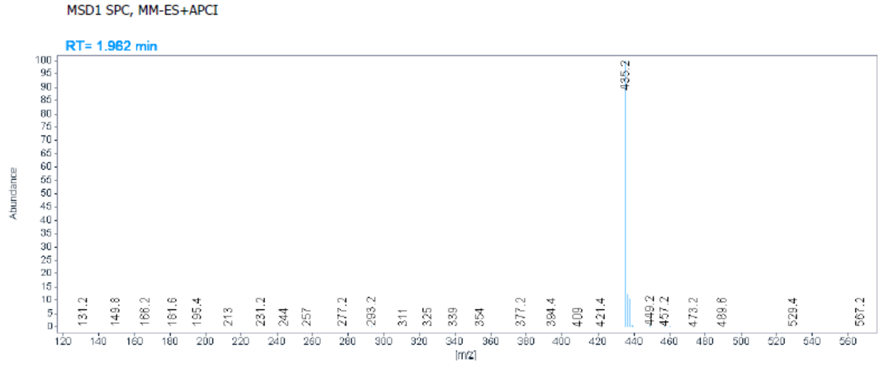

Signal: DAD1 A, Sig=250,100 Ref=off

RT [min]	Peak Area Percent
1.751	2.64
1.895	95.26
2.266	1.11
2.881	0.99

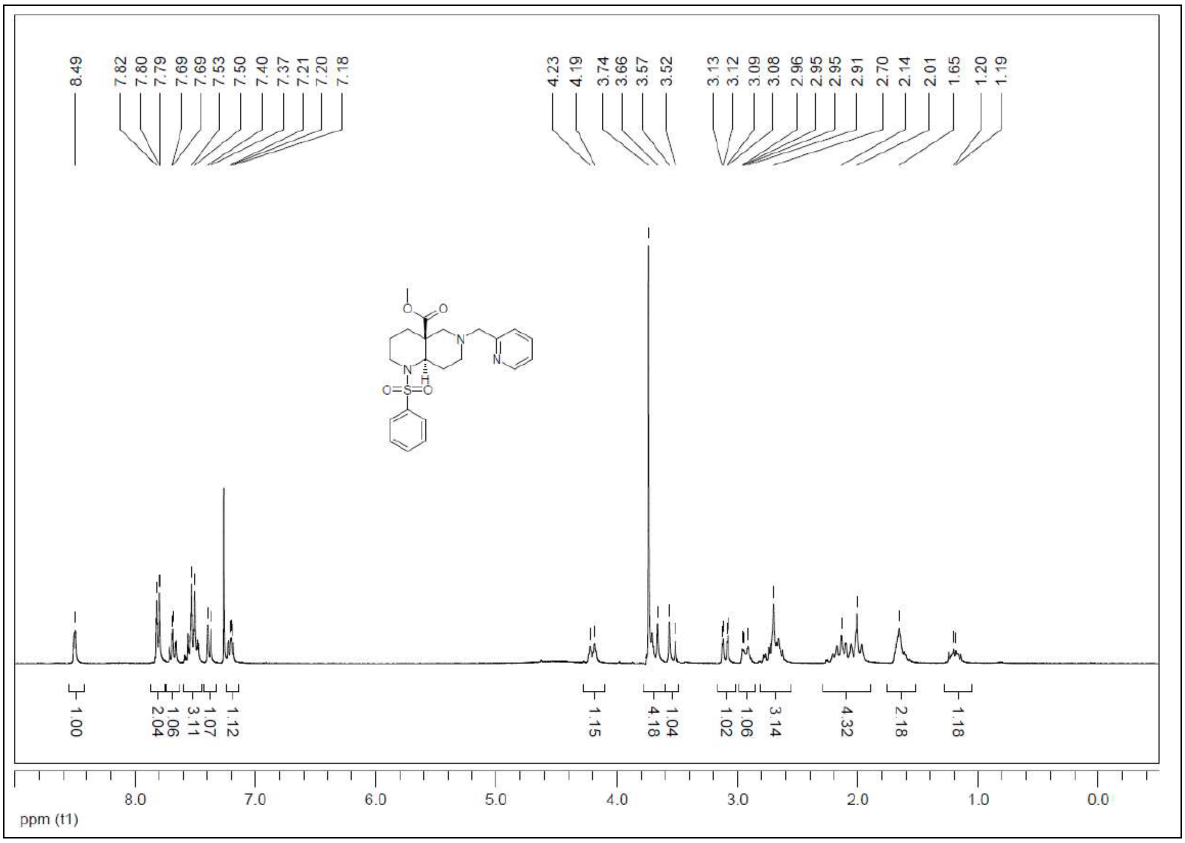

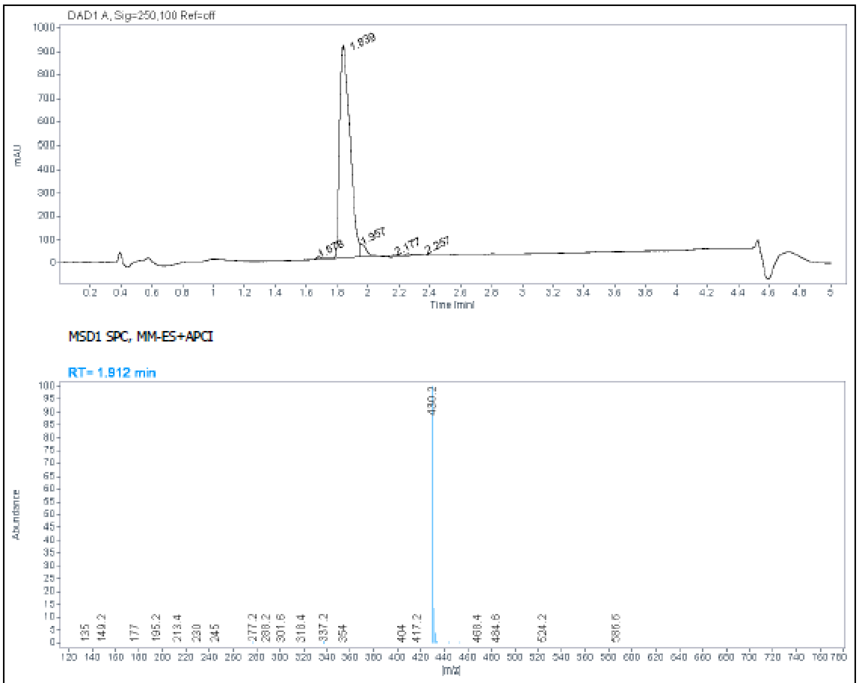

Signal: DAD1 A, Sig=250,100 Ref=off

RT [min]	Peak Area Percent
1.678	0.62
1.839	94.98
1.957	3.15
2.177	0.64
2.257	0.62

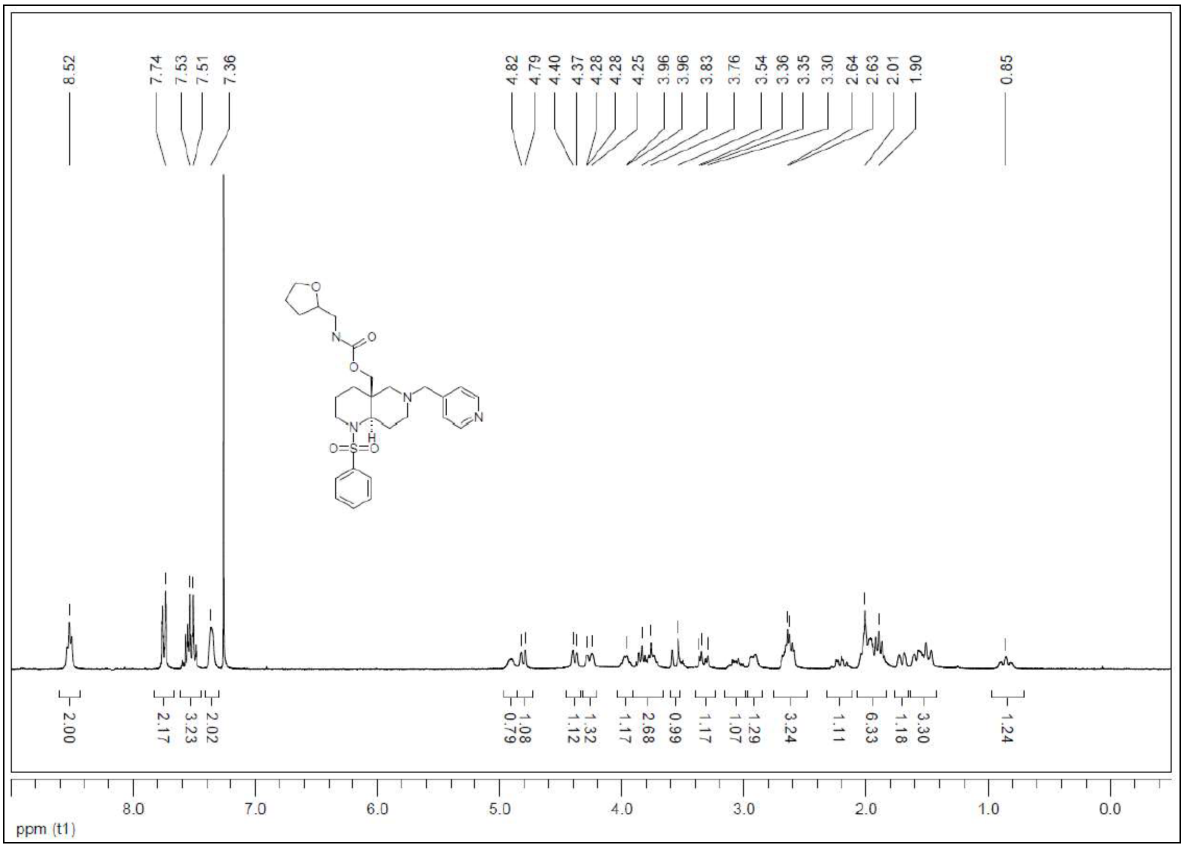

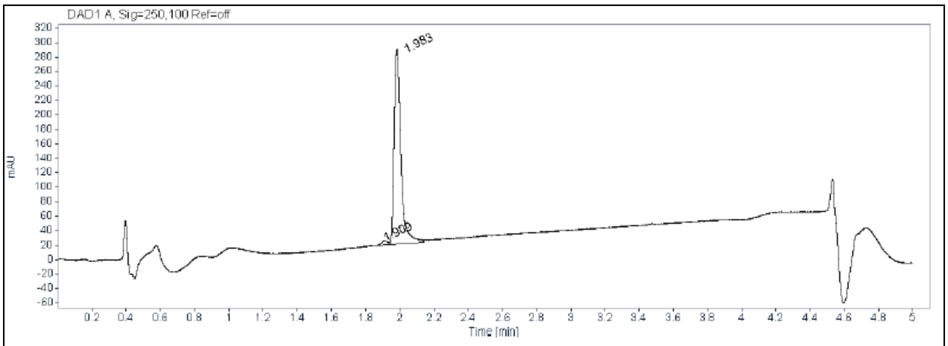

MSD1 SPC, MM-ES+APCI

RT= 2.054 min

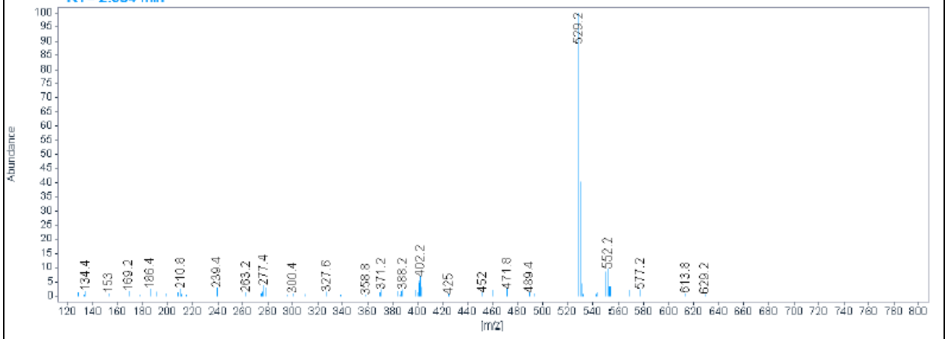

Signal: DAD1 A, Sig=250,100 Ref=off

**RT [min]**	Peak Area Percent
1.909	1.83
1.983	98.17

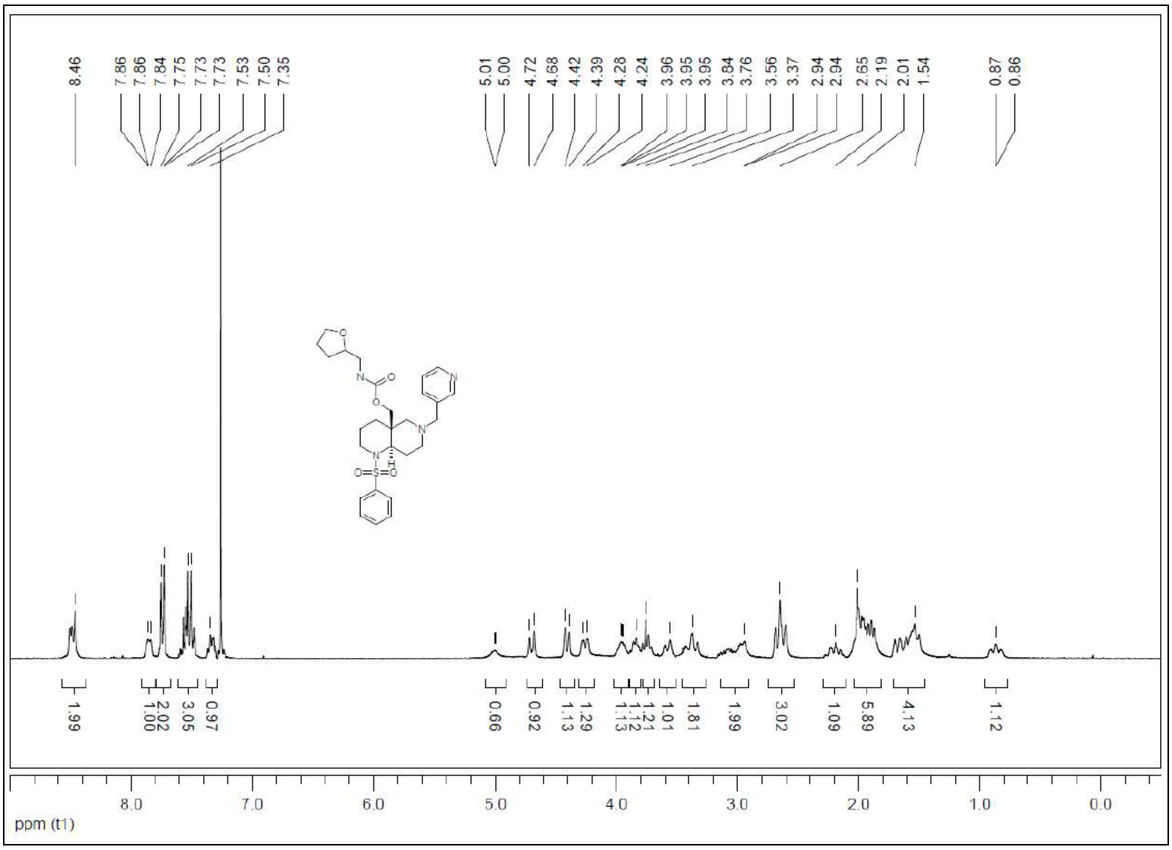

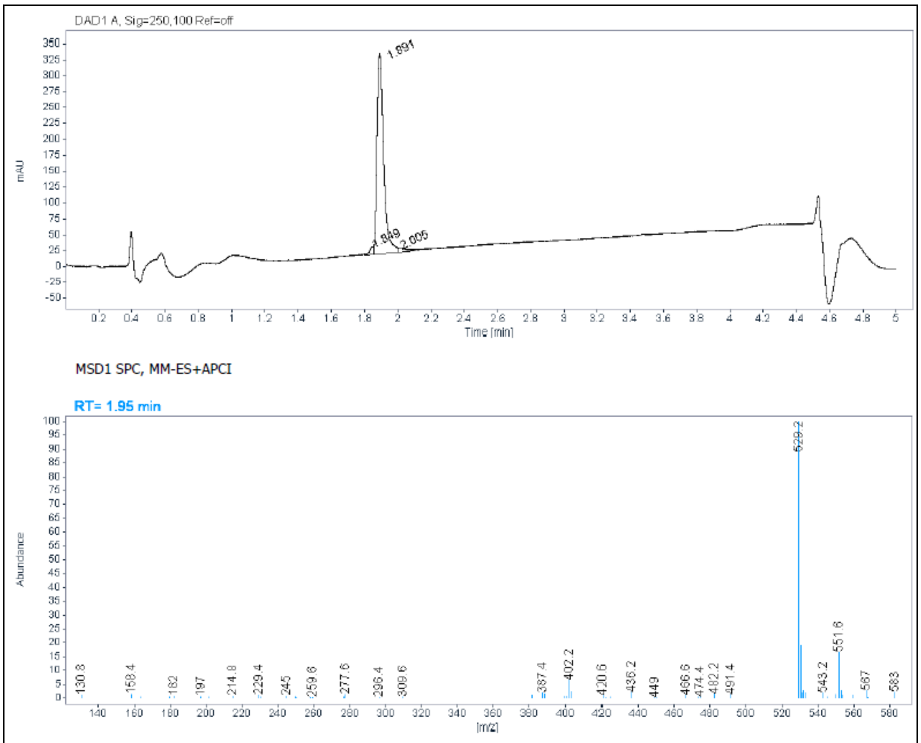

Signal: DAD1 A, Sig=250,100 Ref=off

RT [min]	Peak Area Percent
1.849	2.19
1.891	95.67
2.005	2.14

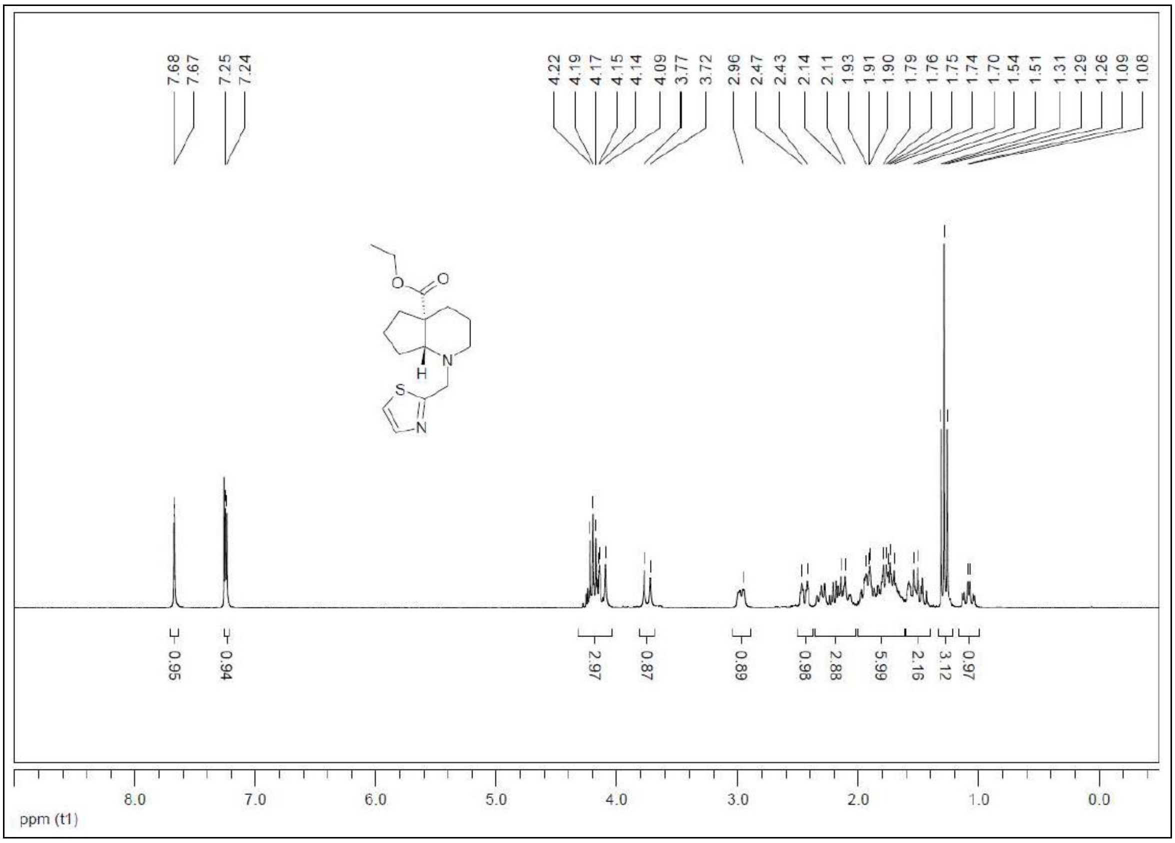

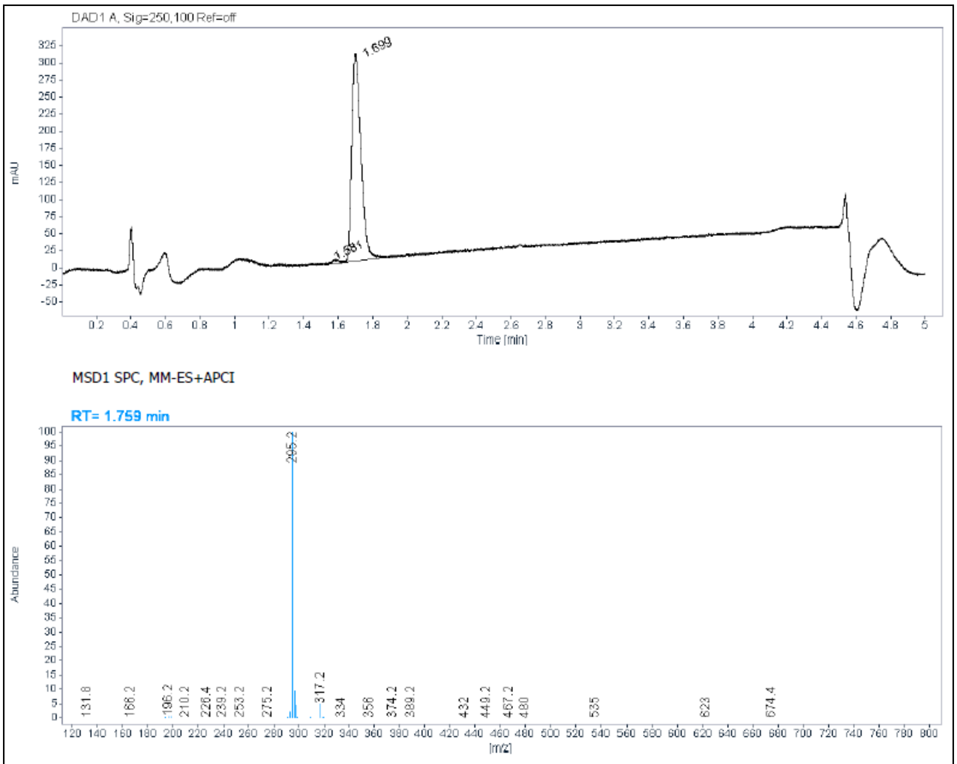

Signal: DAD1 A, Sig=250,100 Ref=off

RT [min]	Peak Area Percent
1.581	1.58
1.699	98.42

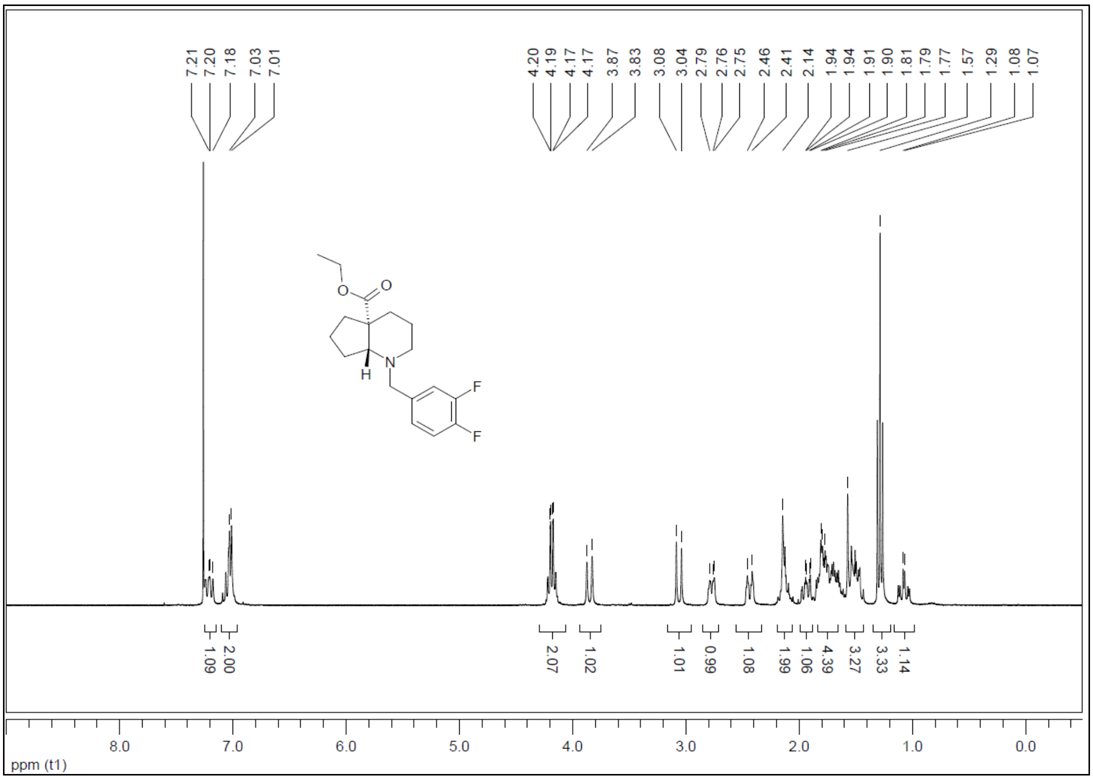

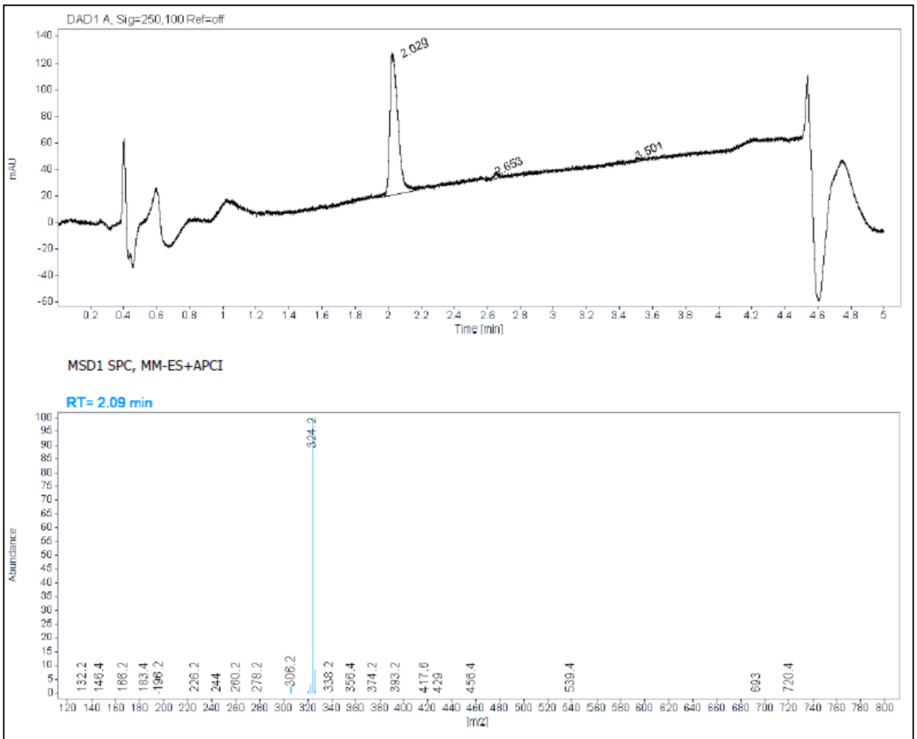

Signal: DAD1 A, Sig=250,100 Ref=off

RT [min]	Peak Area Percent
2.029	96.35
2.653	2.07
3.501	1.58

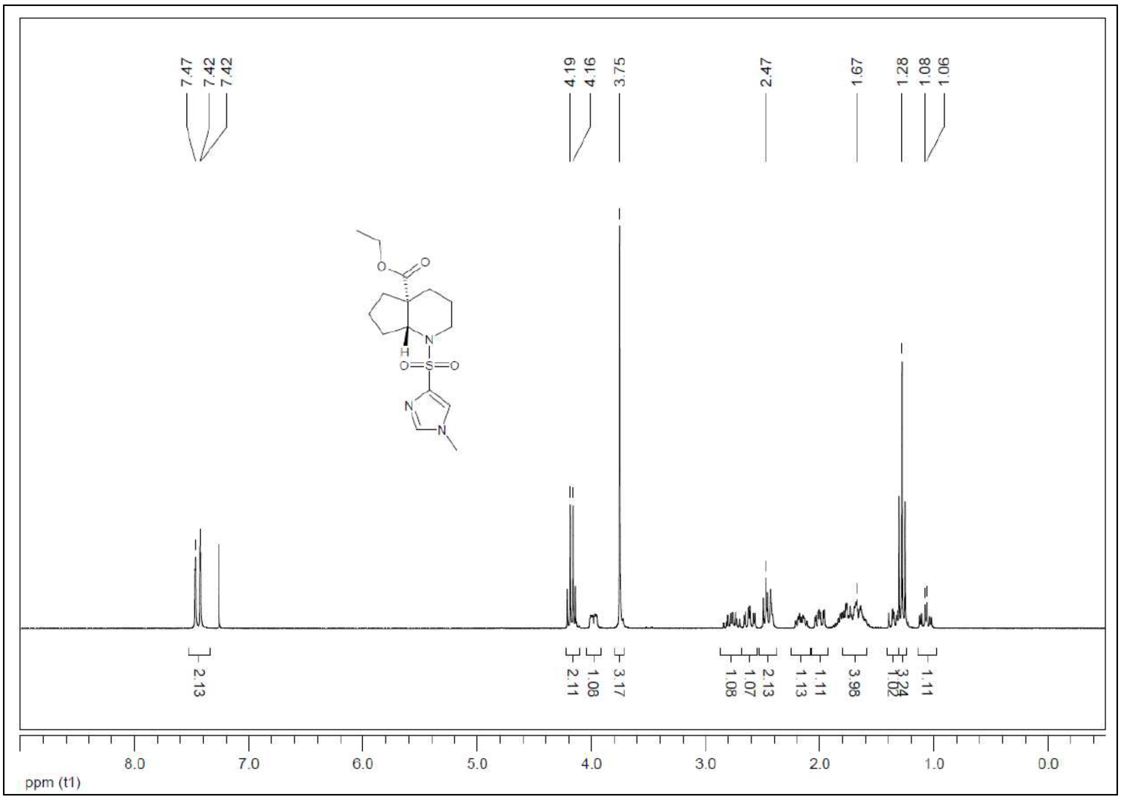

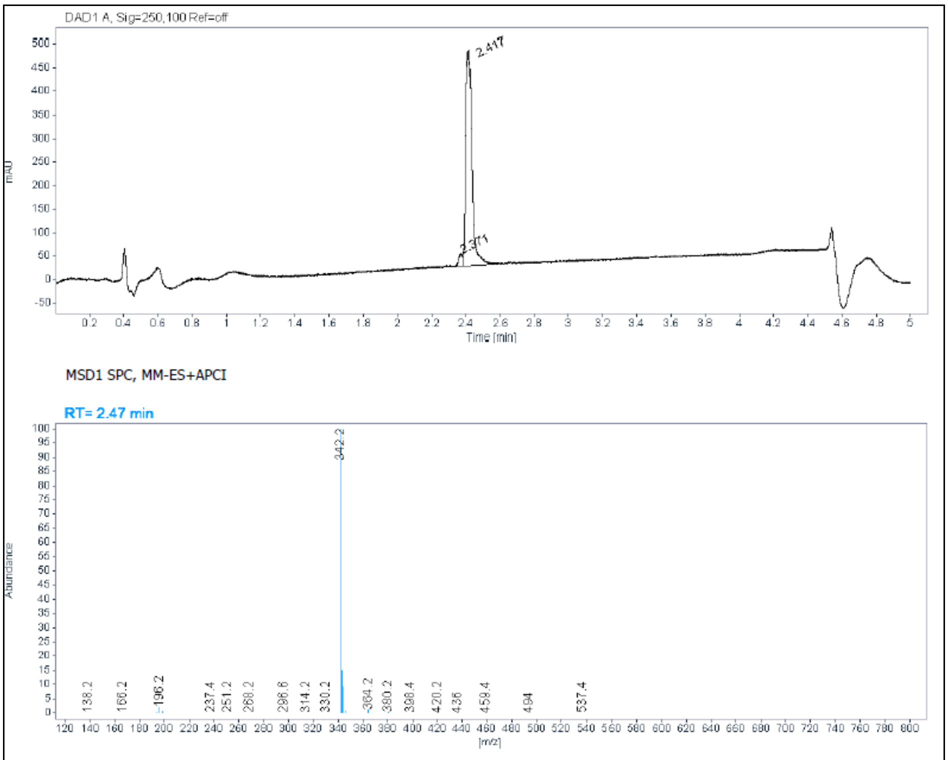

Signal: DAD1 A, Sig=250,100 Ref=off

RT [min]	Peak Area Percent
2.371	3.39
2.417	96.61

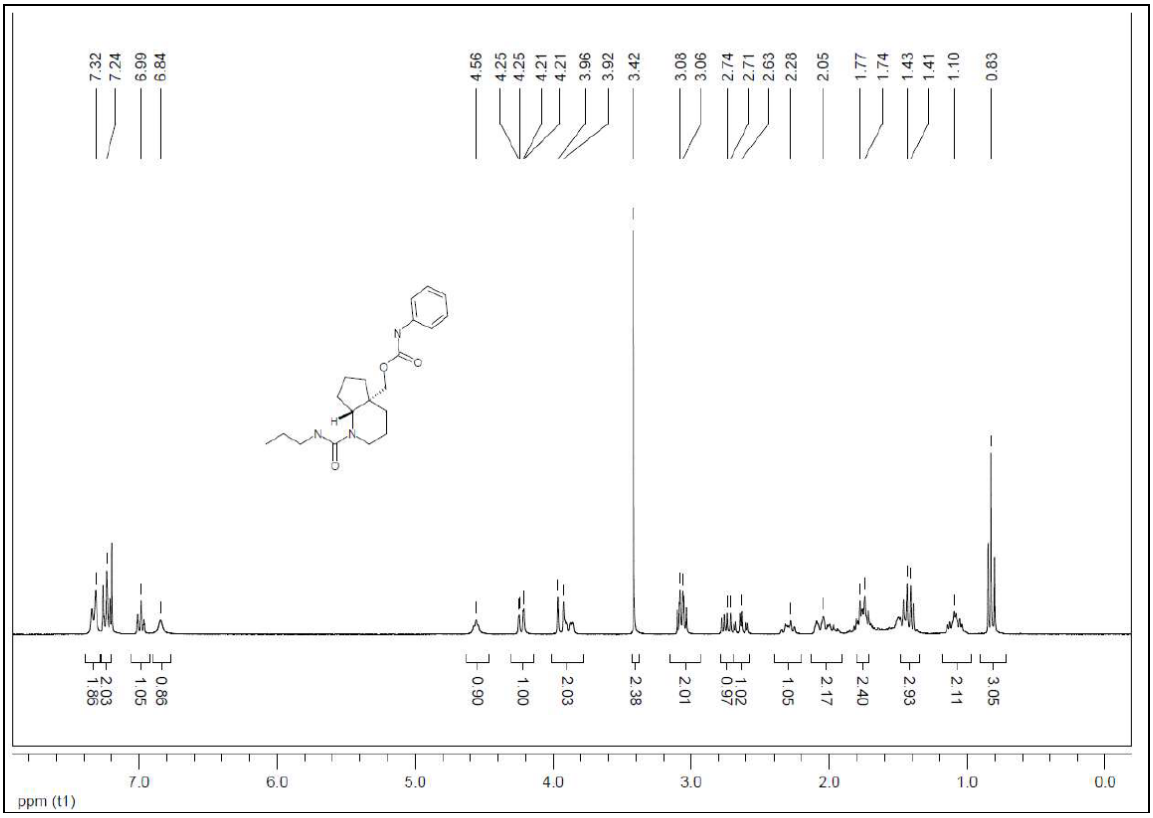

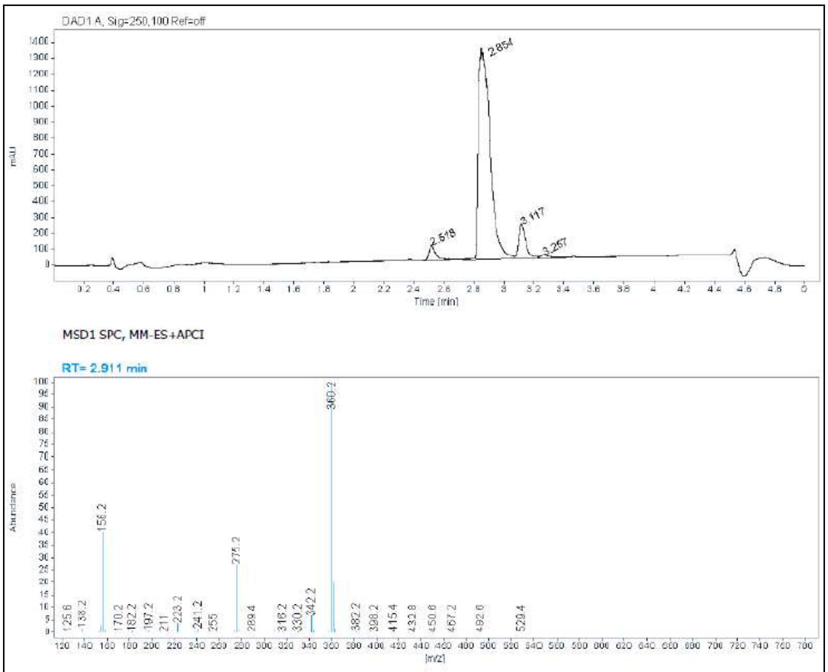

Signal: DAD1 A, Sig=250,100 Ref=off

RT [min]	Peak Area Percent
2.518	3.70
2.854	88.78
3.117	8.32
3.267	1.18

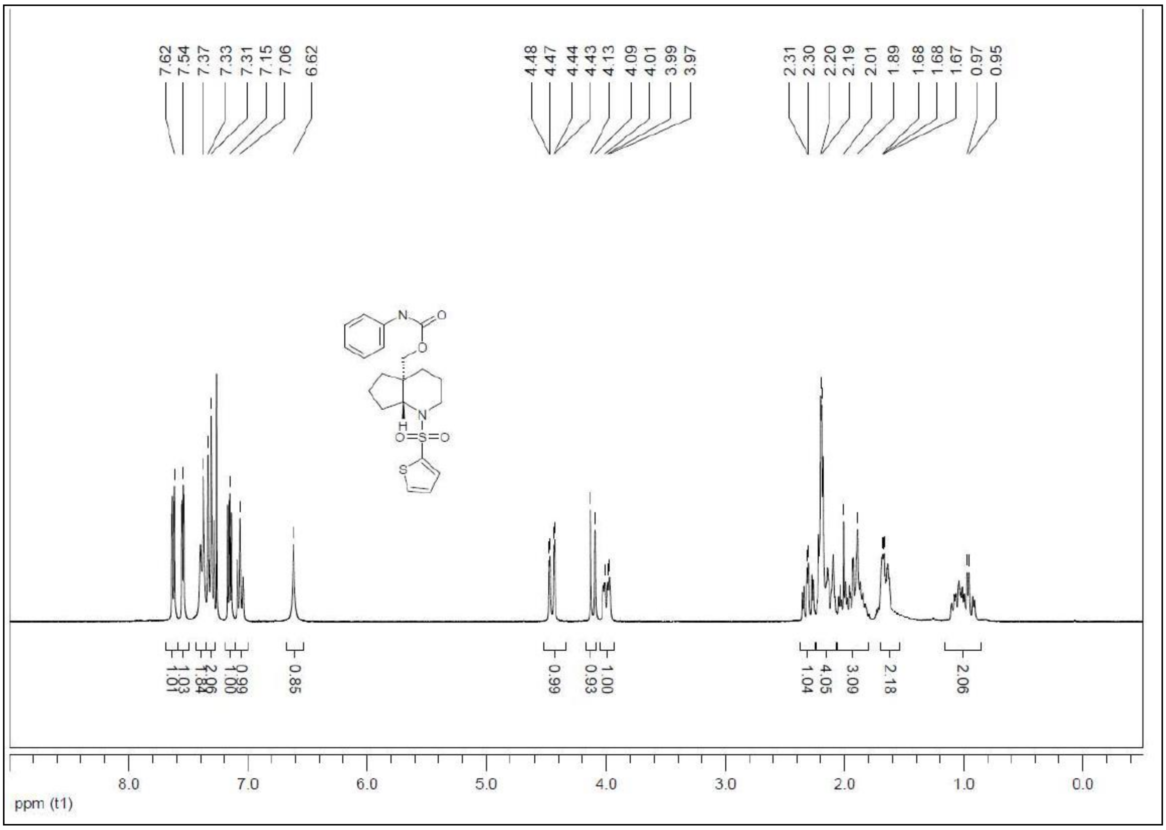

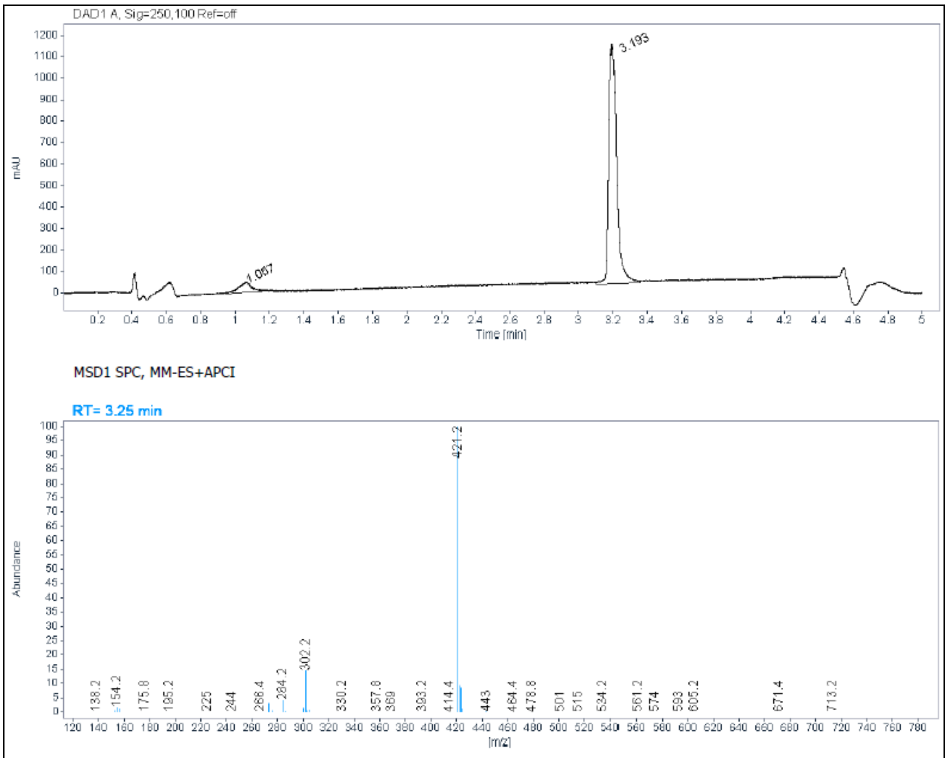

Signal: DAD1 A, Sig=250,100 Ref=off

RT [min]	Peak Area Percent
1.067	6.09
3.193	93.91

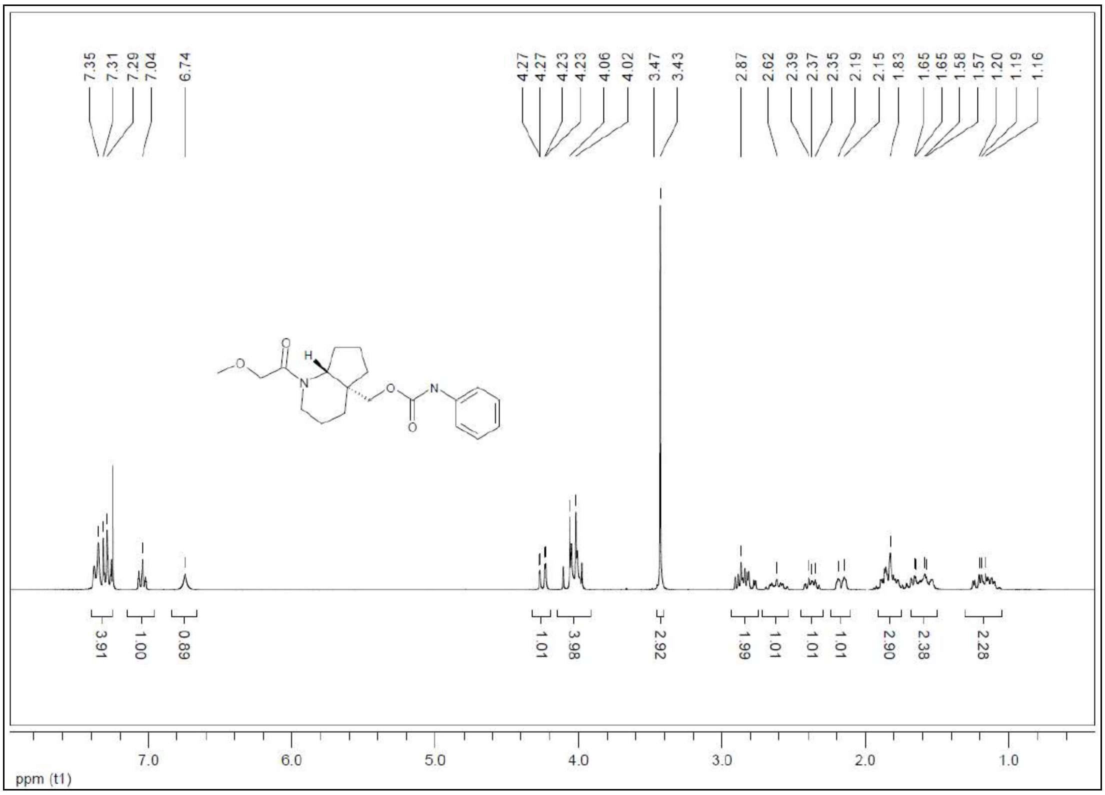

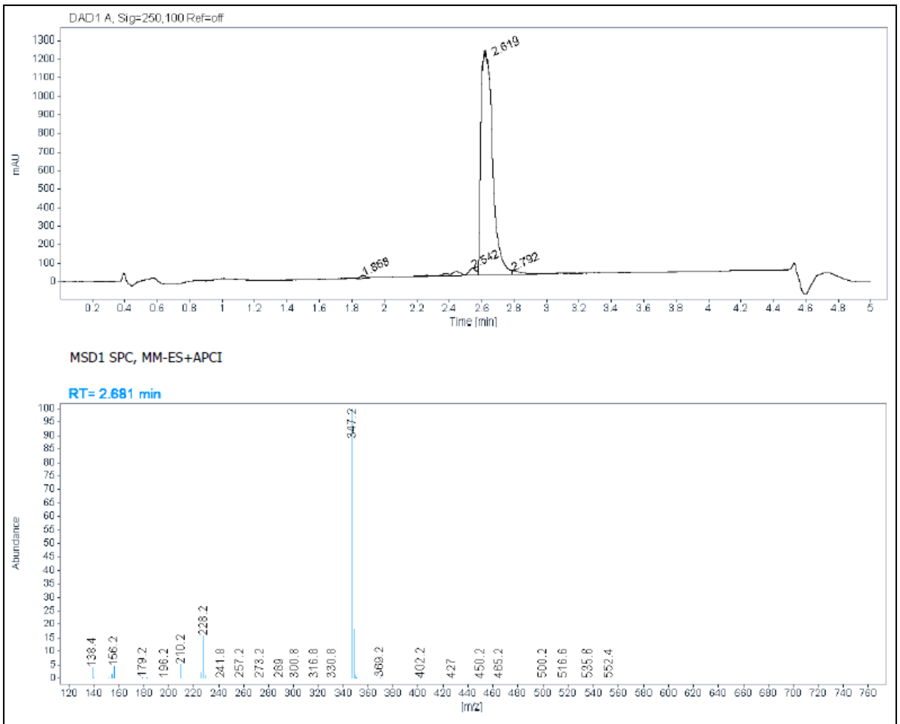

Signal: DAD1 A, Sig=250,100 Ref=off

RT [min]	Peak Area Percent
1.868	0.55
2.542	4.18
2.619	93.92
2.792	1.35

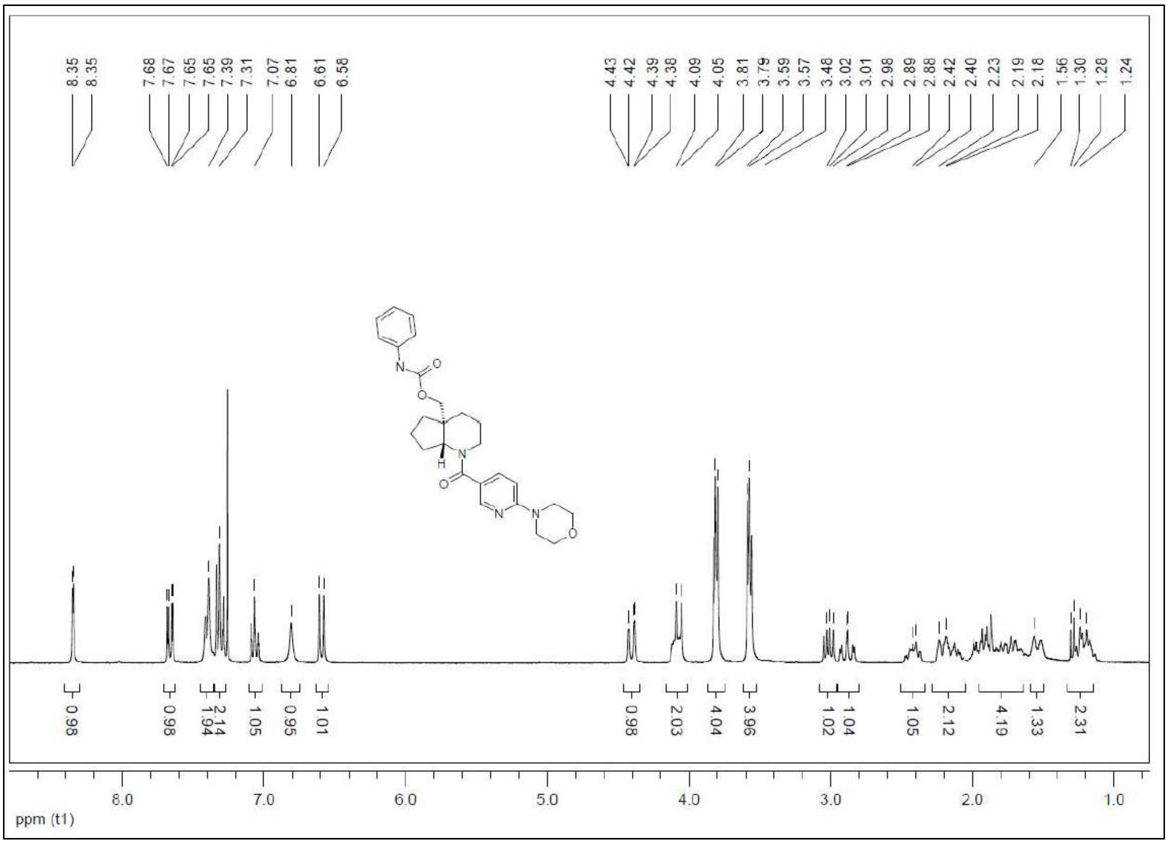

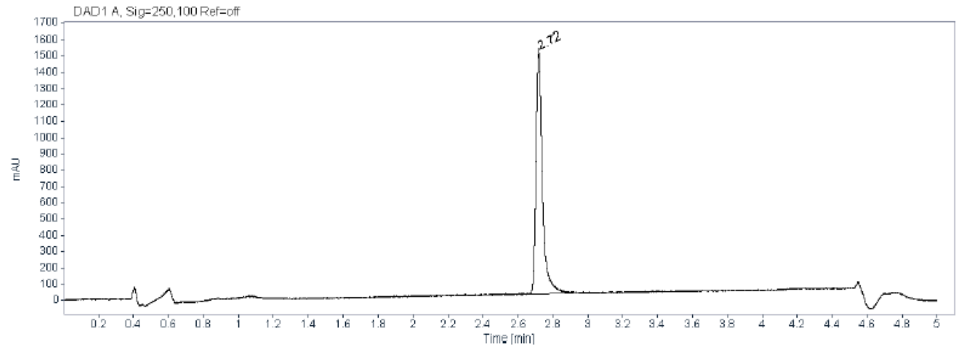

MSD1 SPC, MM-ES+APCI

RT= 2.776 min

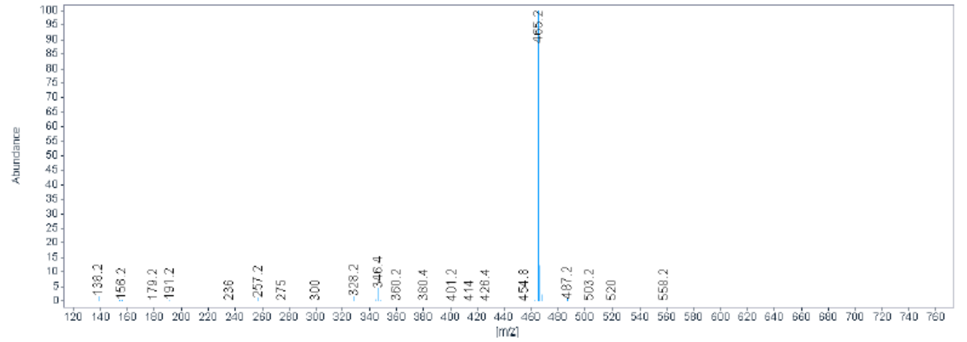

Signal: DAD1 A, Sig=250,100 Ref=off

RT [min]	Peak Area Percent
2.720	100.00

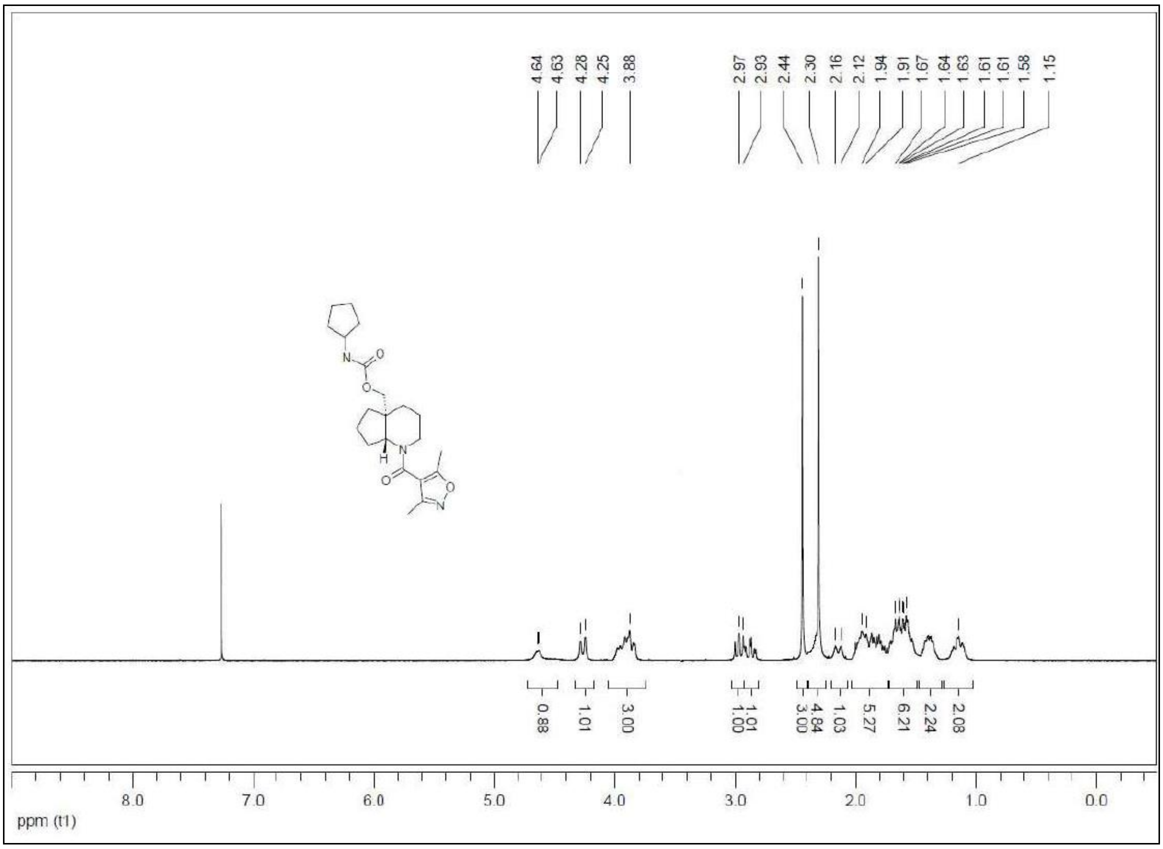

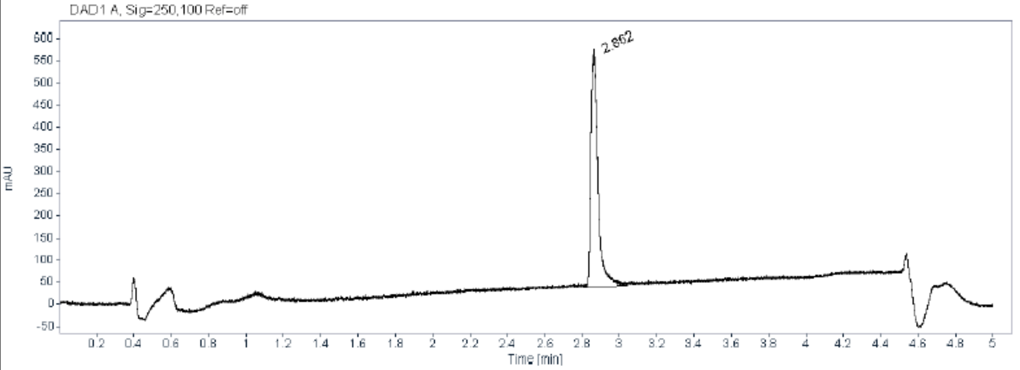

MSD1 SPC, MM-ES+APCI

RT= 2.919 min

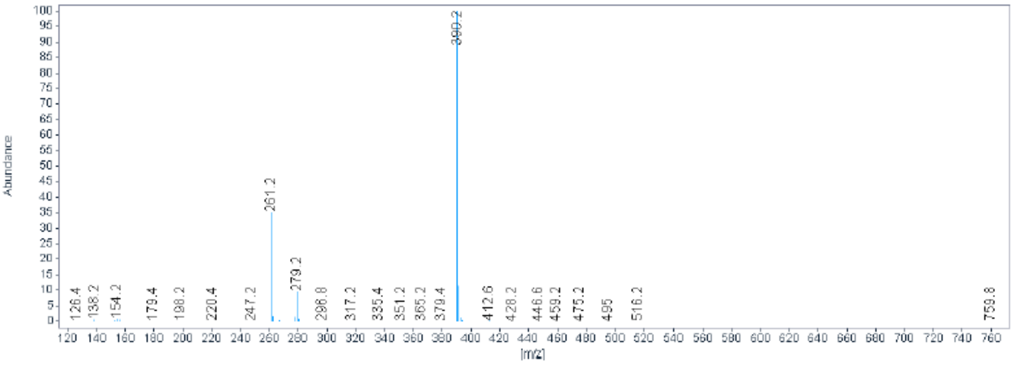

Signal: DAD1 A, Sig=250,100 Ref=off

RT [min]	Peak Area Percent
2.862	100.00

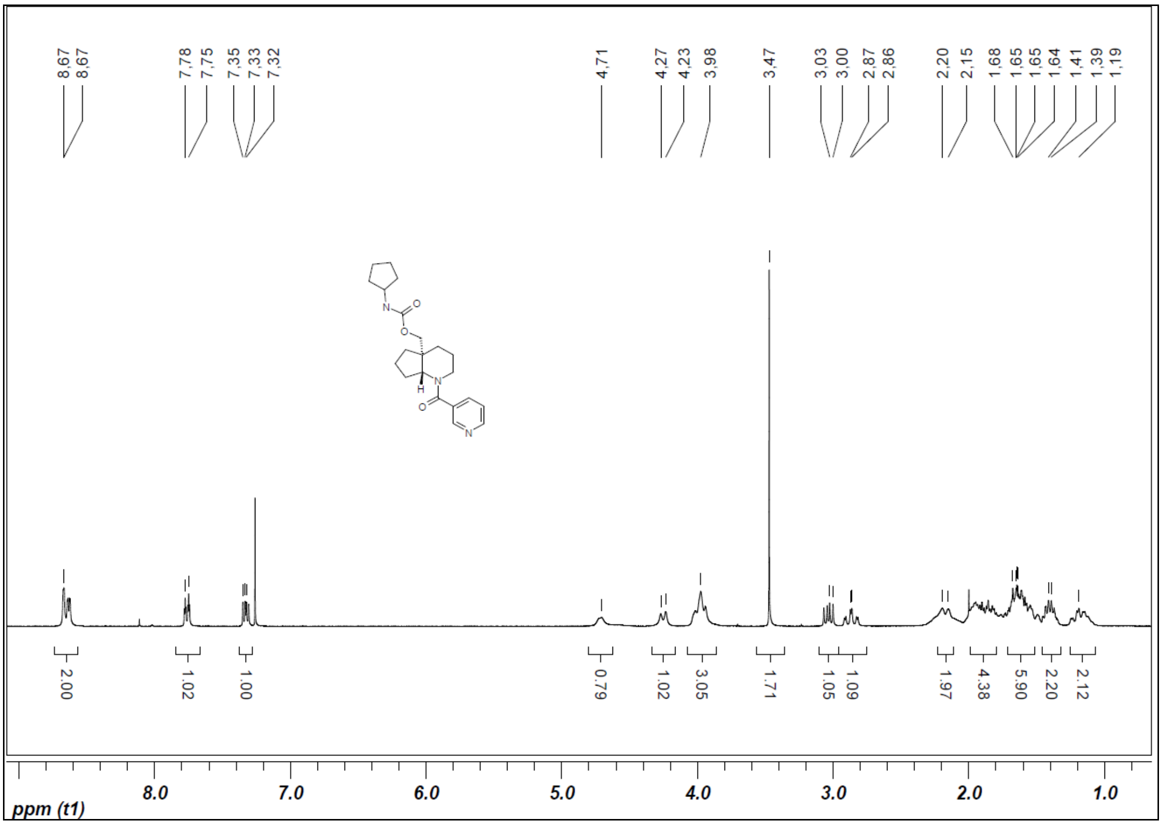

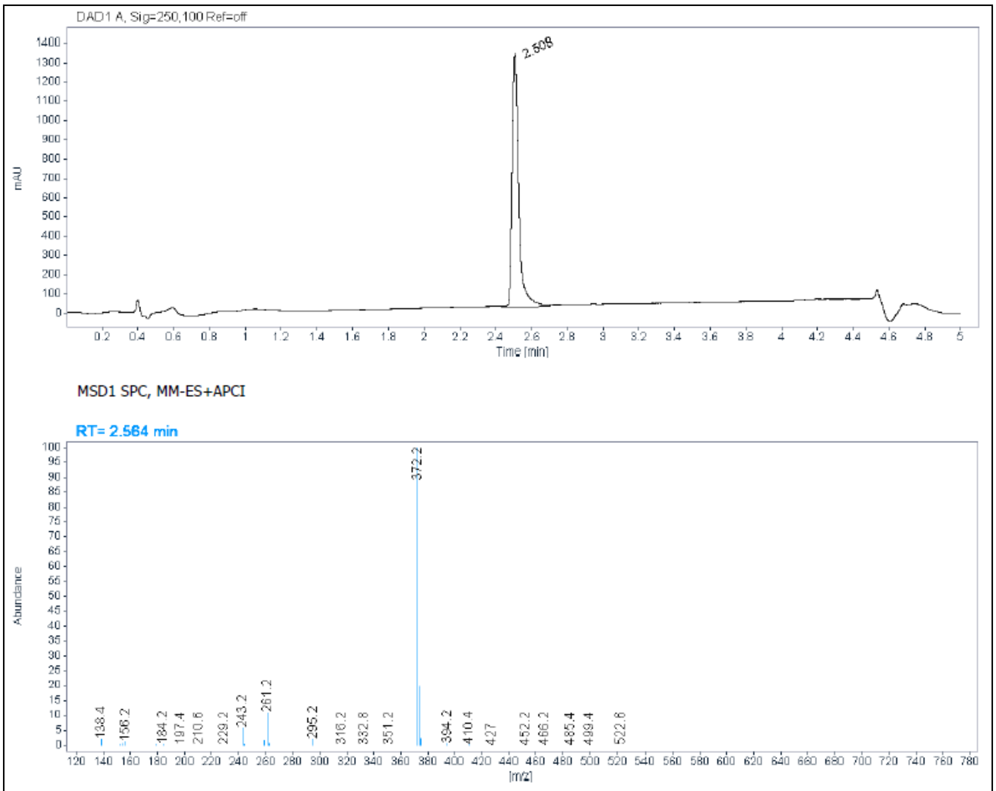

Signal: DAD1 A, Sig=250,100 Ref=off

RT [min]	Peak Area Percent
2.508	100.00

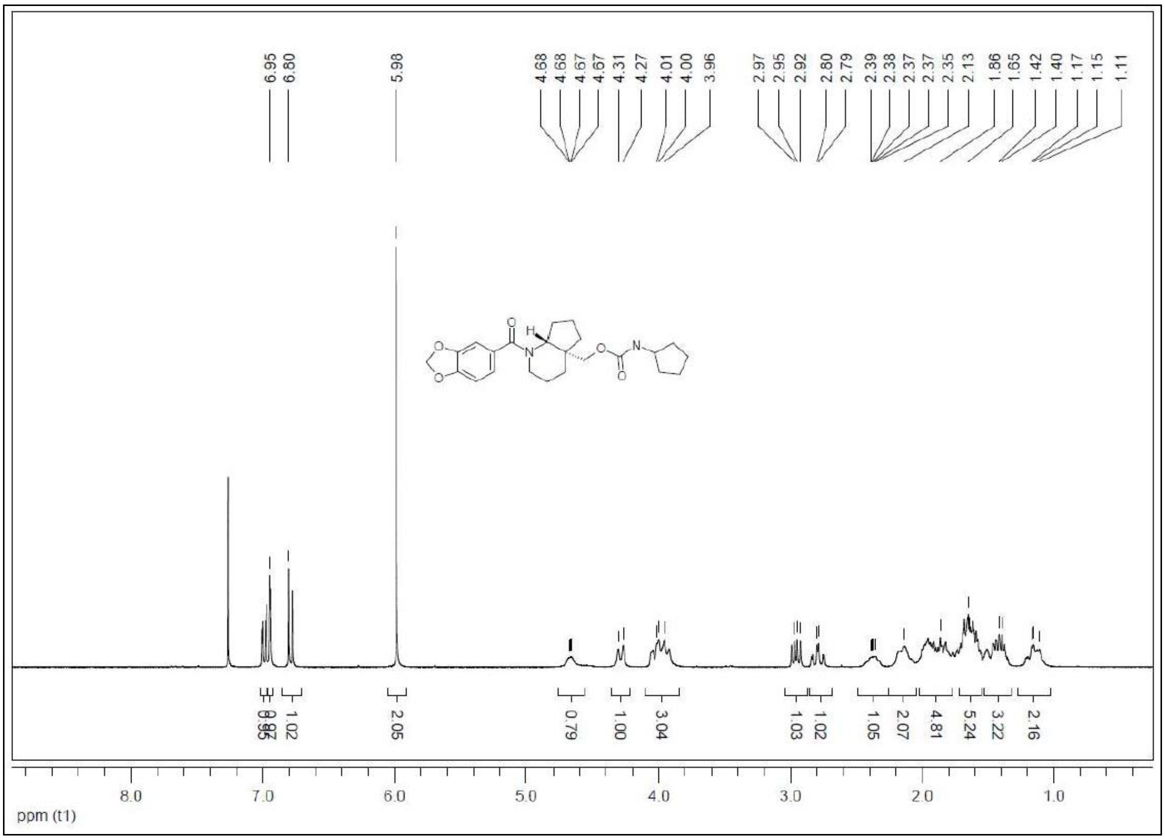

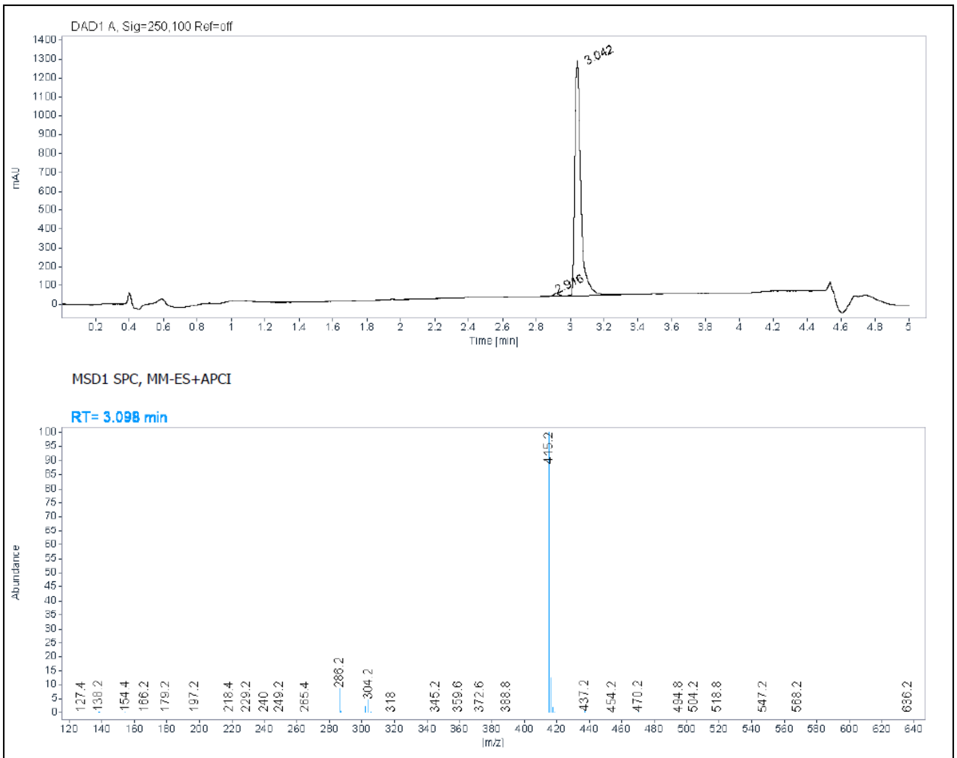

Signal: DAD1 A, Sig=250,100 Ref=off

**RT [min]**	Peak Area Percent
2.916	0.97
3.042	99.03

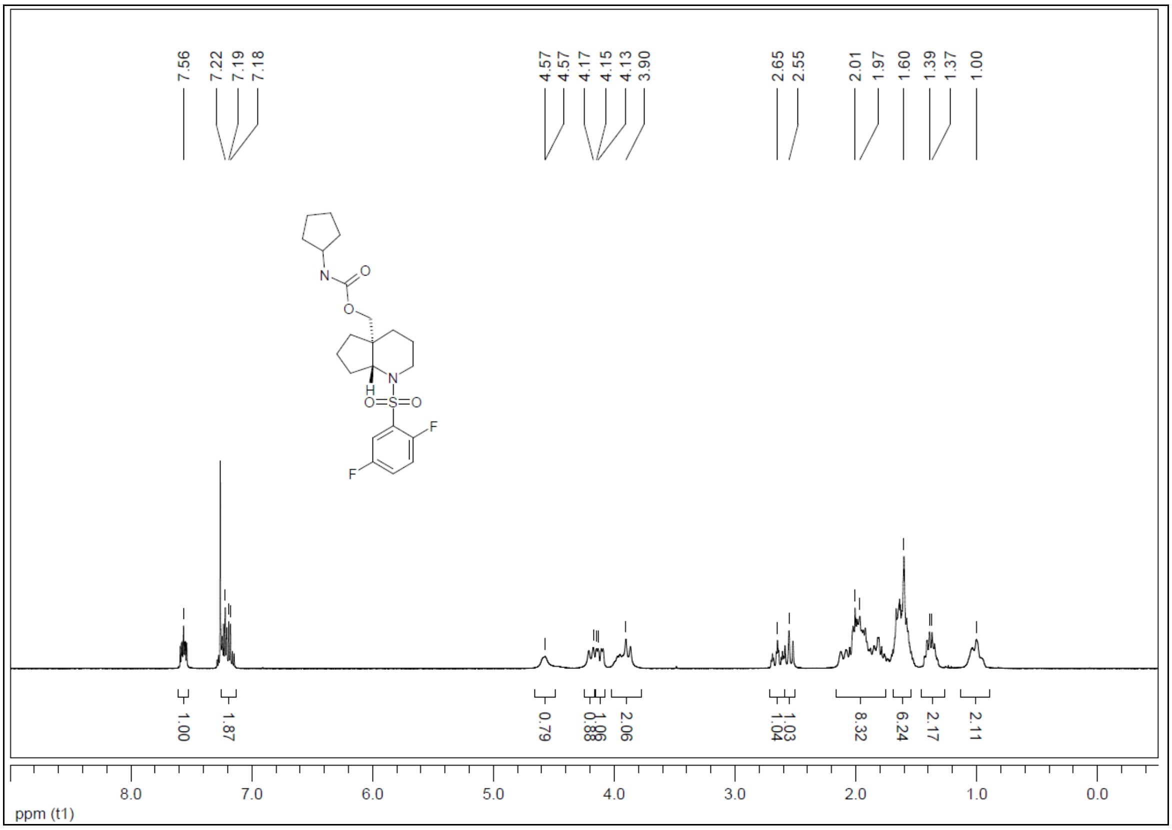

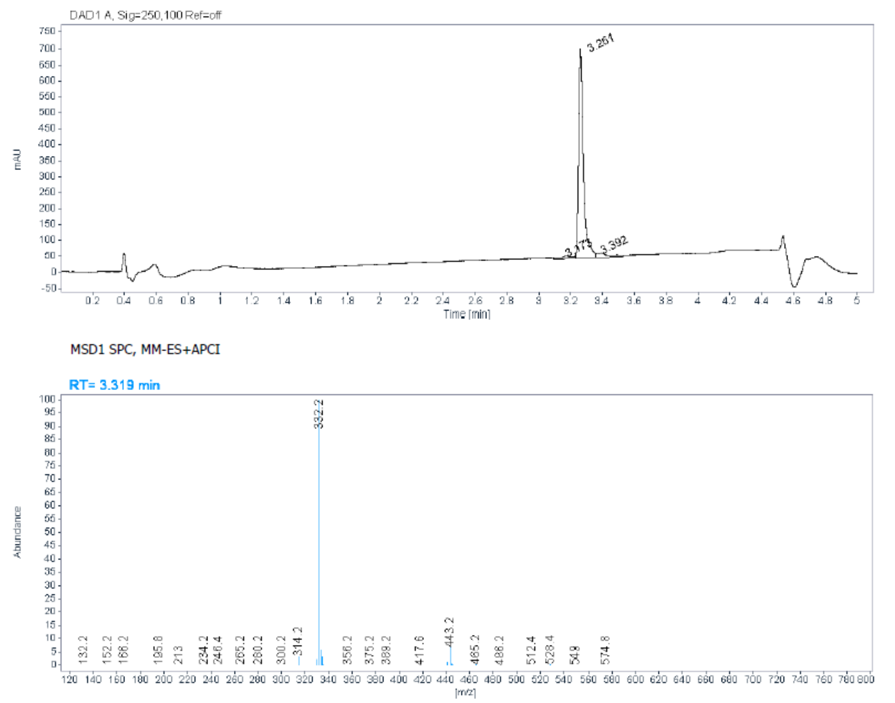

Signal: DAD1 A, Sig=250,100 Ref=off

RT [min]	Peak Area Percent
3.173	2.60
3.261	92.77
3.392	4.63

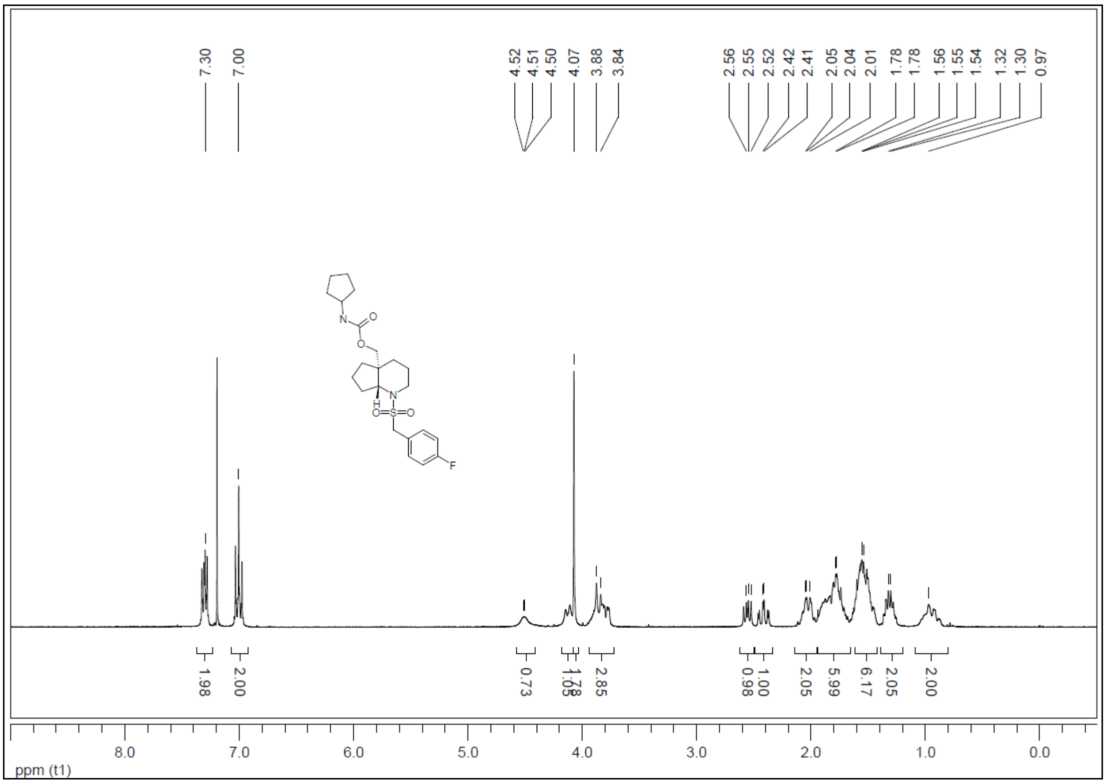

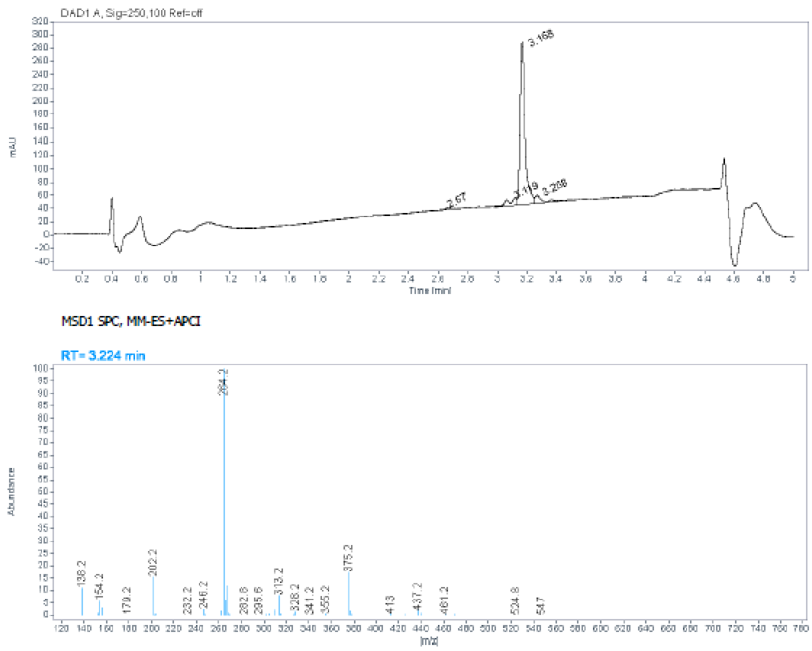

Signal: DAD1 A, Sig=250,100 Ref=off

RT [min]	Peak Area Percent
2.670	1.05
3.119	6.65
3.168	85.12
3.268	7.18

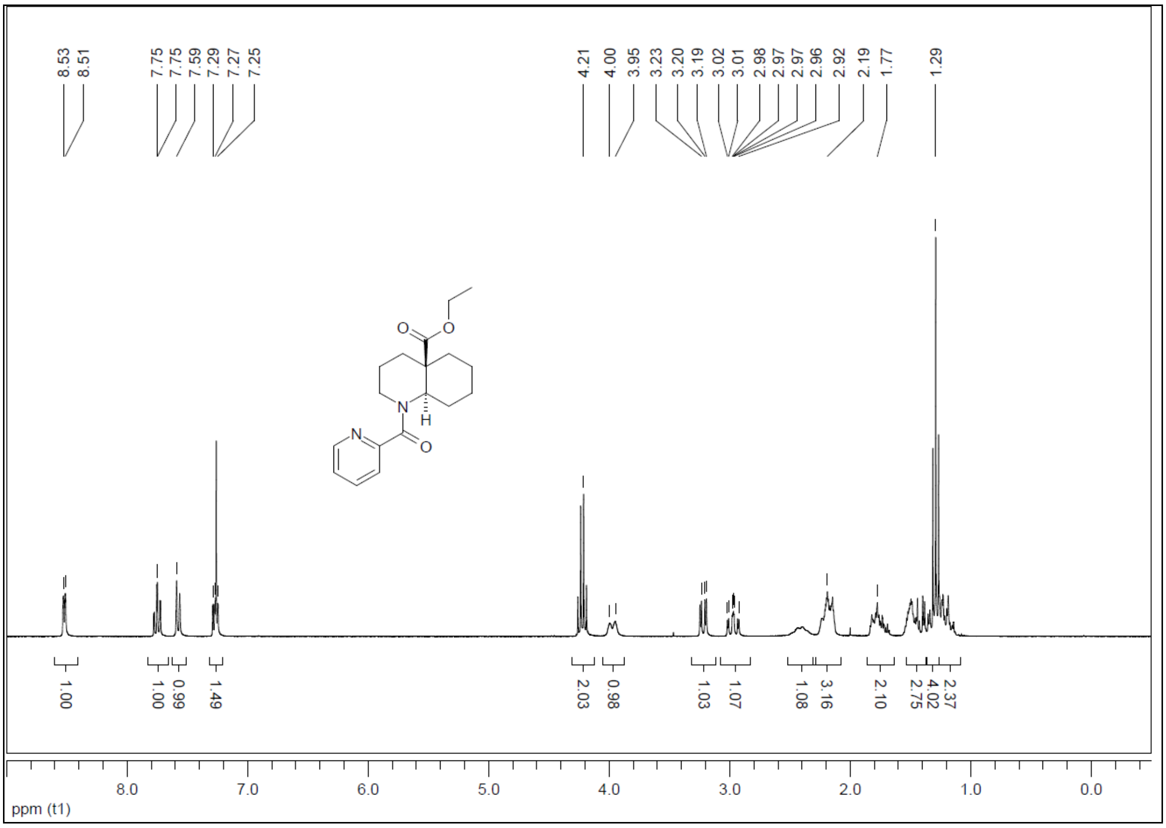

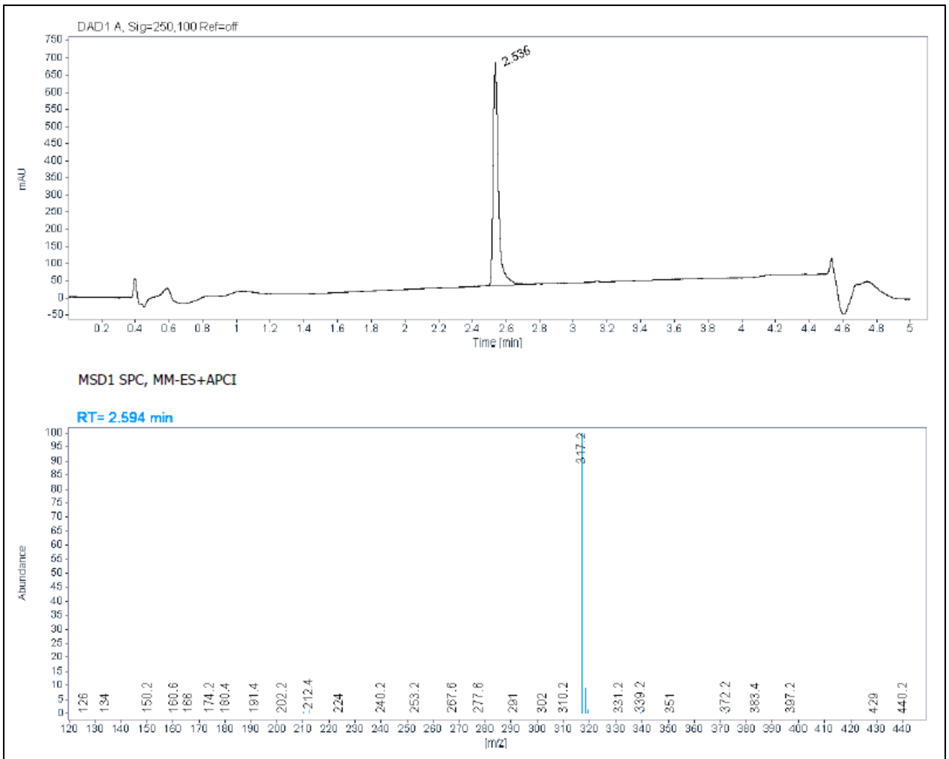

Signal: DAD1 A, Sig=250,100 Ref=off

RT [min]	Peak Area Percent
2.536	100.00

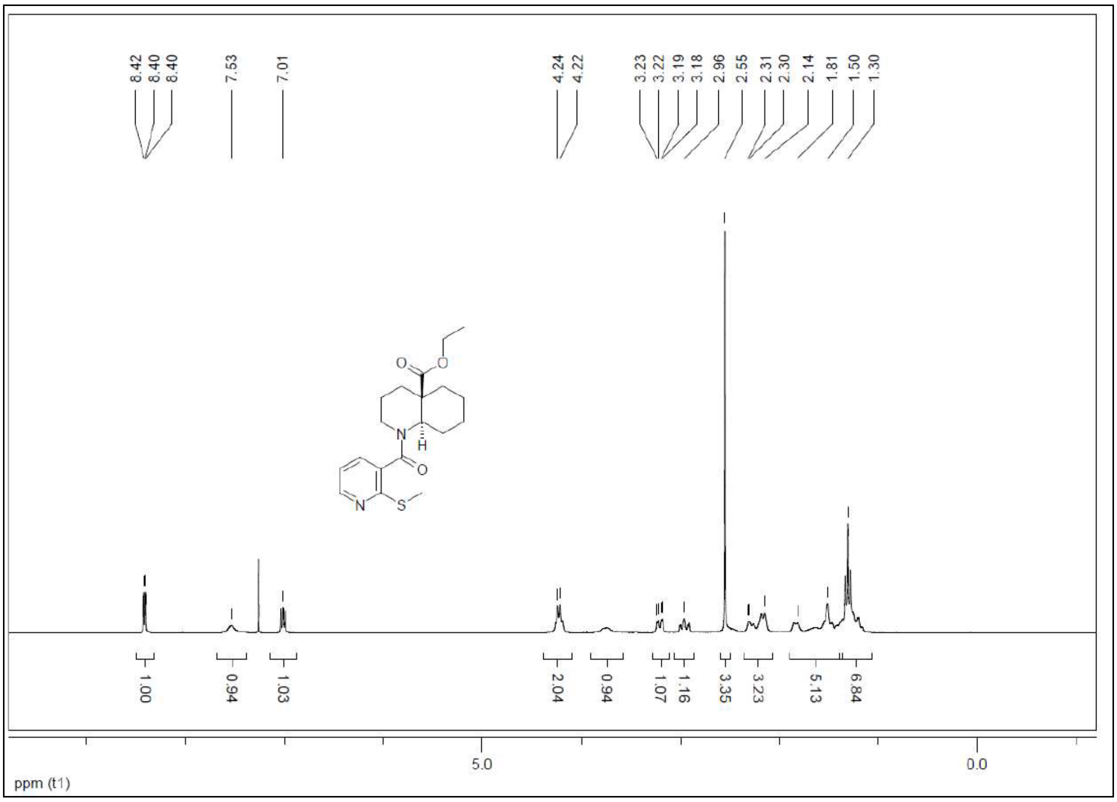

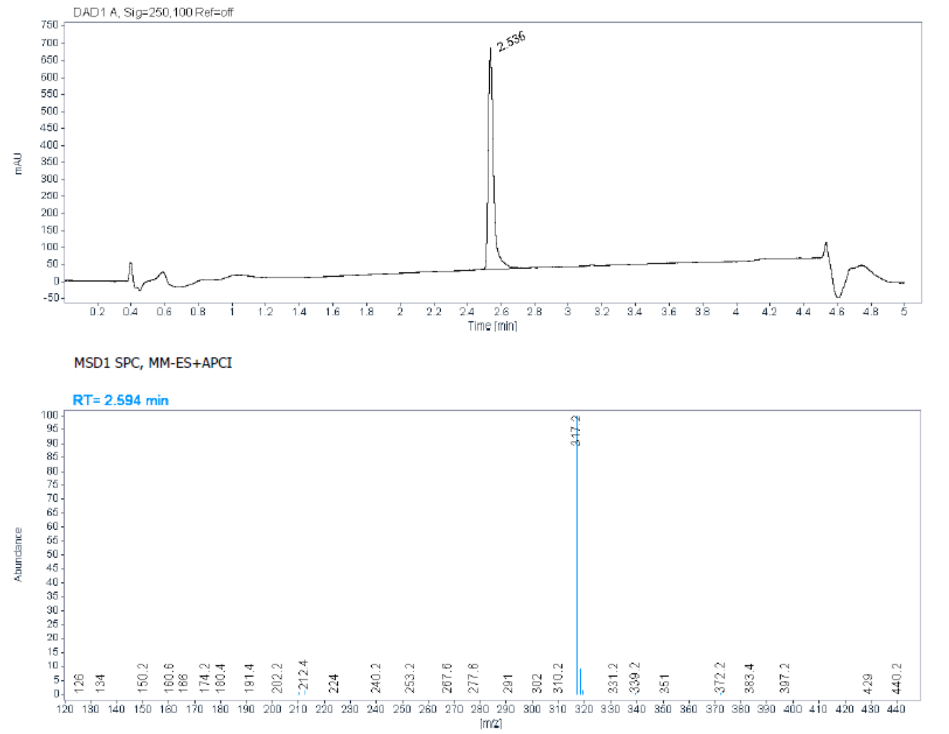

Signal: DAD1 A, Sig=250,100 Ref=off

RT [min]	Peak Area Percent
2.536	100.00
